# SPR‐UPLC‐QTOF/MS‐GNPS‐Guided Discovery of Hypermonol J as a Novel Tumor Necrosis Factor‐Binding Small‐Molecule Lead for Inflammatory Storm Intervention

**DOI:** 10.1002/mco2.70960

**Published:** 2026-08-28

**Authors:** Yongqi Li, Weiguang Sun, Xingpiao Jin, Lanqin Li, Xiaotian Zhang, Pingping Fan, Yahui Huang, Chaohu Xiong, Jinsheng Wang, Jiangchun Wei, Yonghui Zhang, Zhengxi Hu

**Affiliations:** ^1^ Hubei Key Laboratory of Natural Medicinal Chemistry and Resource Evaluation, and Wuhan (China–Romania) Belt and Road Joint Laboratory on Traditional Chinese Medicine, School of Pharmacy, Tongji Medical College Huazhong University of Science and Technology Wuhan China; ^2^ Hubei Shizhen Laboratory Wuhan China; ^3^ Shanxi Provincial Innovation Center for Upper Gastrointestinal Cancer Research and Clinical Translation Heping Hospital Affiliated to Changzhi Medical College Changzhi China; ^4^ Shanxi Provincial Department‐Municipal Key Laboratory Cultivation Base for Quality Enhancement and Utilization of Shangdang Chinese Medicinal Materials, School of Pharmacy Changzhi Medical College Changzhi China; ^5^ Department of Pharmacy, The Central Hospital of Wuhan, Tongji Medical College Huazhong University of Science and Technology Wuhan China; ^6^ College of Pharmacy Hubei University of Medicine Shiyan China; ^7^ School of Pharmaceutical Sciences Zhejiang Chinese Medical University Hangzhou China

**Keywords:** acute lung injury, *Hypericum monogynum*, hypermonol J, psoriasis, surface plasmon resonance‐active ingredient recognition system, tumor necrosis factor

## Abstract

This study developed an integrated strategy combining surface plasmon resonance (SPR) for real‐time biomolecular interaction analysis, ultra‐performance liquid chromatography coupled with quadrupole time‐of‐flight mass spectrometry (UPLC‐QTOF/MS) for compound profiling, and Global Natural Products Social Molecular Networking (GNPS)‐based molecular networking for structural annotation in complex herbal systems. Using this platform, we identified hypermonol J (HPJ), a polycyclic polyprenylated acylphloroglucinol (PPAP), as a high‐affinity small‐molecule binder of tumor necrosis factor (TNF) (*K*_D_ = 25.9 pM). SPR and orthogonal isothermal titration calorimetry (ITC) supported direct TNF binding, whereas mechanistic assays suggested that HPJ may preferentially stabilize TNF dimers, thereby impairing trimer assembly and downstream TNF receptor 1 (TNFR1)/nuclear factor kappa B (NF‐κB) signaling. In a preclinical psoriasis model, topical 1% HPJ alleviated imiquimod (IMQ)‐induced epidermal hyperplasia and suppressed IL‐17/IL‐23 pathways. In an acute lung injury (ALI) model, 10 mg/kg HPJ reduced neutrophil infiltration, lowered serum inflammatory cytokine levels, and restored pulmonary tight junction integrity. Biosafety assessments supported the preliminary tolerability of HPJ under the tested conditions. These findings identify HPJ as a promising small‐molecule lead for further pharmacological optimization and underscore the value of SPR‐guided natural product discovery for inflammatory storm intervention.

## Graphical abstract

SPR‐UPLC‐QTOF/MS‐GNPS‐guided screening identified hypermonol J (HPJ) as a high‐affinity TNF‐binding small molecule. HPJ may stabilize TNF dimers, reduce active trimer formation, and suppress TNFR1/NF‐κB signaling, thereby alleviating inflammatory pathology in psoriasis and acute lung injury models. These findings support HPJ as a promising small‐molecule lead for further optimization (created by BioRender.com).

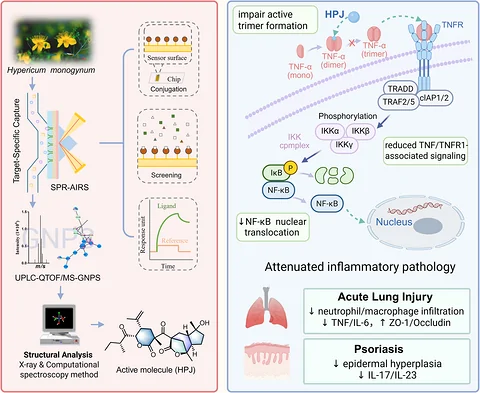

## 1 Introduction

Tumor necrosis factor (TNF) is a central cytokine that regulates immune homeostasis and contributes to inflammatory and autoimmune disorders, including rheumatoid arthritis, psoriasis, and Crohn's disease [1]. TNF functions primarily as a homotrimer that engages TNFR1 and TNFR2, thereby activating proinflammatory signaling pathways [2, 3, 4, 5]. Dysregulated TNF production drives tissue damage and chronic inflammation in these diseases, establishing TNF inhibition as a cornerstone therapeutic strategy [6, 7, 8, 9]. Although biologic TNF inhibitors are clinically effective, their high production costs, immunogenicity, parenteral administration, and limited tissue penetration underscore the need for small‐molecule TNF‐binding leads with improved tissue distribution, structural tunability, and potential utility in combination therapy [10, 11, 12, 13].

Traditional herbal medicines, including *Hypericum monogynum*, represent a rich but underexplored reservoir of bioactive compounds [14]. Their clinical use in autoimmune conditions suggests that some constituents may modulate inflammatory pathways [15, 16, 17]. However, the chemical complexity of these natural product libraries makes target‐guided identification of active constituents challenging.

Natural products have long contributed to anti‐inflammatory drug discovery [18], but many early discoveries relied on empirical observations and labor‐intensive purification [19]. By contrast, modern target‐based screening can accelerate lead identification; however, synthetic and semi‐synthetic libraries often lack the chemical diversity and three‐dimensional complexity of natural products [20]. Efficient strategies that connect complex natural product mixtures with defined molecular targets are therefore needed.

To address this challenge, we integrated surface plasmon resonance (SPR)‌, ‌ultra‐performance liquid chromatography coupled with quadrupole time‐of‐flight mass spectrometry (UPLC‐QTOF/MS)‌, and ‌Global Natural Products Social Molecular Networking (GNPS) into an SPR‐active ingredient recognition system (SPR‐AIRS)‌ [21, 22]. This workflow enables label‐free capture of target binders from crude extracts, followed by UPLC‐QTOF/MS characterization and GNPS‐based molecular‐network annotation [23, 24].

In this study, we applied an integrated SPR‐UPLC‐QTOF/MS‐GNPS strategy to identify bioactive constituents from *H. monogynum* with potential relevance to autoimmune and inflammatory diseases. Using TNF as a model target for inflammatory storm intervention, we successfully isolated hypermonol J (HPJ) as a high‐affinity TNF‐binding small molecule. SPR analysis revealed a dissociation constant of 25.9 pM, suggesting a strong binding between HPJ and TNF. Mechanistic studies supported a working model in which HPJ binds TNF dimers, impairs their assembly into biologically active trimers, and thereby suppresses TNF‐mediated signaling. Finally, in vivo experiments using the acute lung injury (ALI) model showed that HPJ attenuated inflammatory cytokine storms and helped restore immune balance. Our work illustrates the potential of this integrated approach to modernize traditional medicine discovery, providing a streamlined path from complex herbal systems to functionally validated lead compounds with therapeutic promise.

## 2 Results

### 2.1 The SPR‐UPLC‐QTOF/MS‐GNPS‐Guided Separation and Identification

**FIGURE 1:**
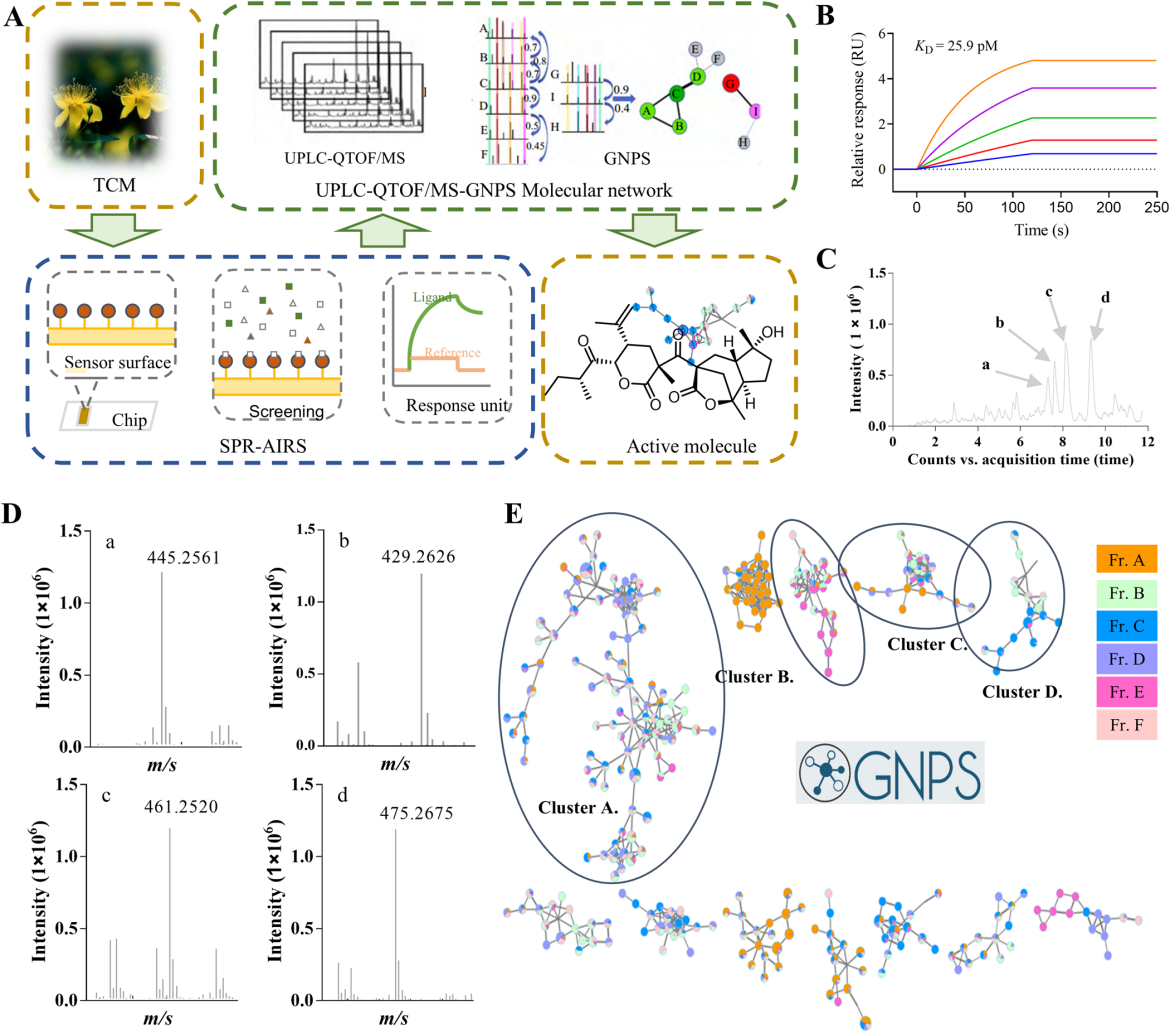
Recovery and identification of TNF‐bound ingredients. (A) SPR‐UPLC‐QTOF/MS‐GNPS workflow. (B) SPR‐derived HPJ affinity. (C and D) TIC and MS profiles of recovered samples. (E) MS/MS‐based molecular network of extract fractions with an expanded protonated cluster. Molecular ions are depicted as pie charts, with node sizes reflecting the degree of correlation between compounds.

**FIGURE 2:**
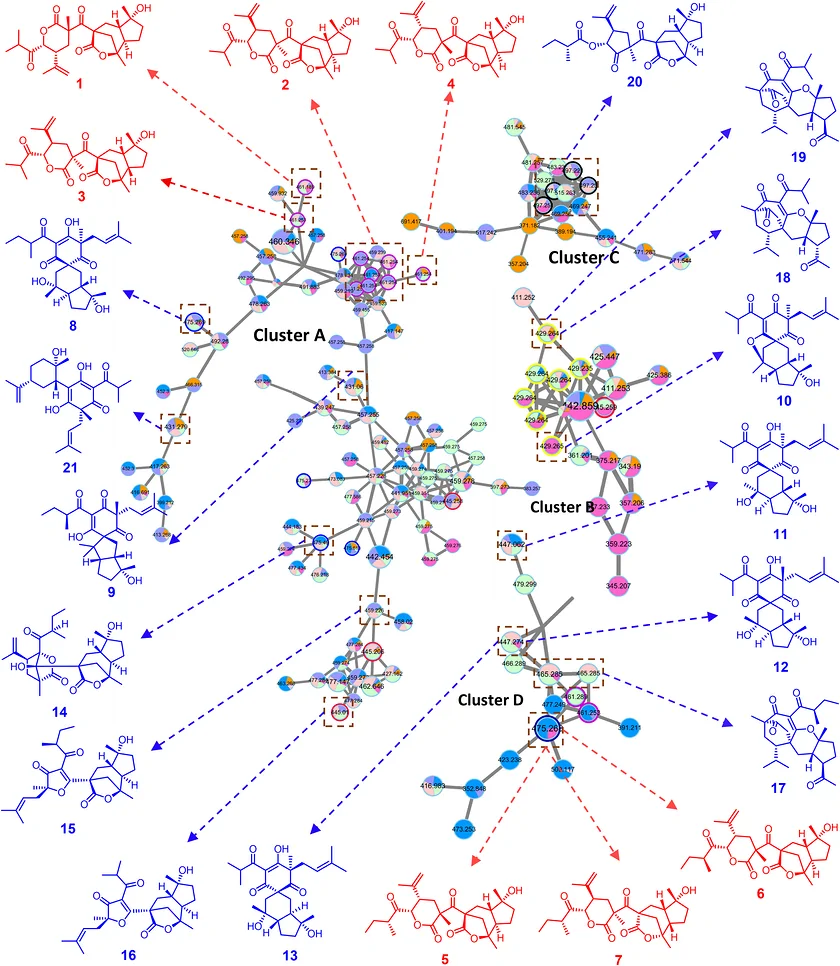
Molecular networking of compounds **1–21**. Nodes are shown as pie charts, with colors indicating spectral‐count contributions from each group.

Our previous studies showed that extract fractions (A–F) of *H. monogynum* exhibited potential TNF inhibitory activity, with Fraction C showing the strongest effect. To identify the active constituents responsible for this activity, Fraction C was subjected to an SPR‐based injection‐recovery assay using the Biacore T200 system. TNF‐bound components were recovered, concentrated under a nitrogen stream, and subsequently characterized by UPLC‐QTOF/MS (Figure 1A). The resulting total ion chromatogram and representative precursor ions are presented in Figure 1C,D. To facilitate structural characterization, the MS/MS data were analyzed through the GNPS platform, which groups compounds with similar fragmentation patterns into molecular families [25]. Molecular networking identified several candidate TNF‐binding ions (*m*/*z* 475.268, 461.254, 429.264, and 445.258), represented as distinct molecular clusters (Figure 1E). Fractions corresponding to these clusters were prioritized for bioactivity‐guided purification, leading to the isolation of putative TNF‐binding compounds. Their direct interactions with TNF were subsequently evaluated by SPR affinity analysis. Among the isolated compounds, HPJ displayed the strongest binding affinity, with a dissociation constant of 25.9 pM (Figure 1B). Guided by SPR results, further bioactivity‐directed isolation afforded seven previously undescribed polycyclic polyprenylated acylphloroglucinols (PPAPs, **1–7**), together with 14 structural analogs (**8–21**) (Figure S1). Expansion of the molecular network cluster revealed that these compounds were organized into distinct nodes annotated by their parent *m*/*z* values, providing additional support for their structural relatedness (Figure 2).

### 2.2 Structural Elucidation of Compounds 1–7

Hypermonol F (**1**) was isolated as colorless crystals. Its molecular formula was established as C_26_H_36_O_7_ based on the ^13^C NMR and HRESIMS data (*m*/*z* 483.2352, [M + Na]^+^, calcd 483.2359), corresponding to nine indices of hydrogen deficiency (IHDs). The planar structure was elucidated by comprehensive analysis of the 2D NMR data (Figure S2). The ^1^H–^1^H COSY spectrum revealed three spin systems: H_3_‐13/H‐12/H_3_‐14, H_2_‐3/H‐4/H‐5, and H_2_‐18/H‐19/H‐23/H_2_‐22/H_2_‐21. Further interpretation of the HMBC correlations established two structural fragments. Unit A was defined by the correlations from H_3_‐7 to C‐1/C‐2/C‐3, from H_3_‐10 to C‐4/C‐8/C‐9, from H‐5 to C‐1/C‐3/C‐8/C‐11/C‐12, and from H_3_‐13/H_3_‐14 to C‐11/C‐12. Unit B was constructed from the correlations from H_3_‐26 to C‐23/C‐24/C‐25, from H_3_‐27 to C‐19/C‐20/C‐21, and from H_2_‐25 to C‐16/C‐17/C‐18/C‐23/C‐24/C‐26. These two units were connected through a carbonyl carbon (C‐6), as supported by the HMBC correlations from H_3_‐7 and H_2_‐25 to C‐6 (Figure S2).

**FIGURE 3:**
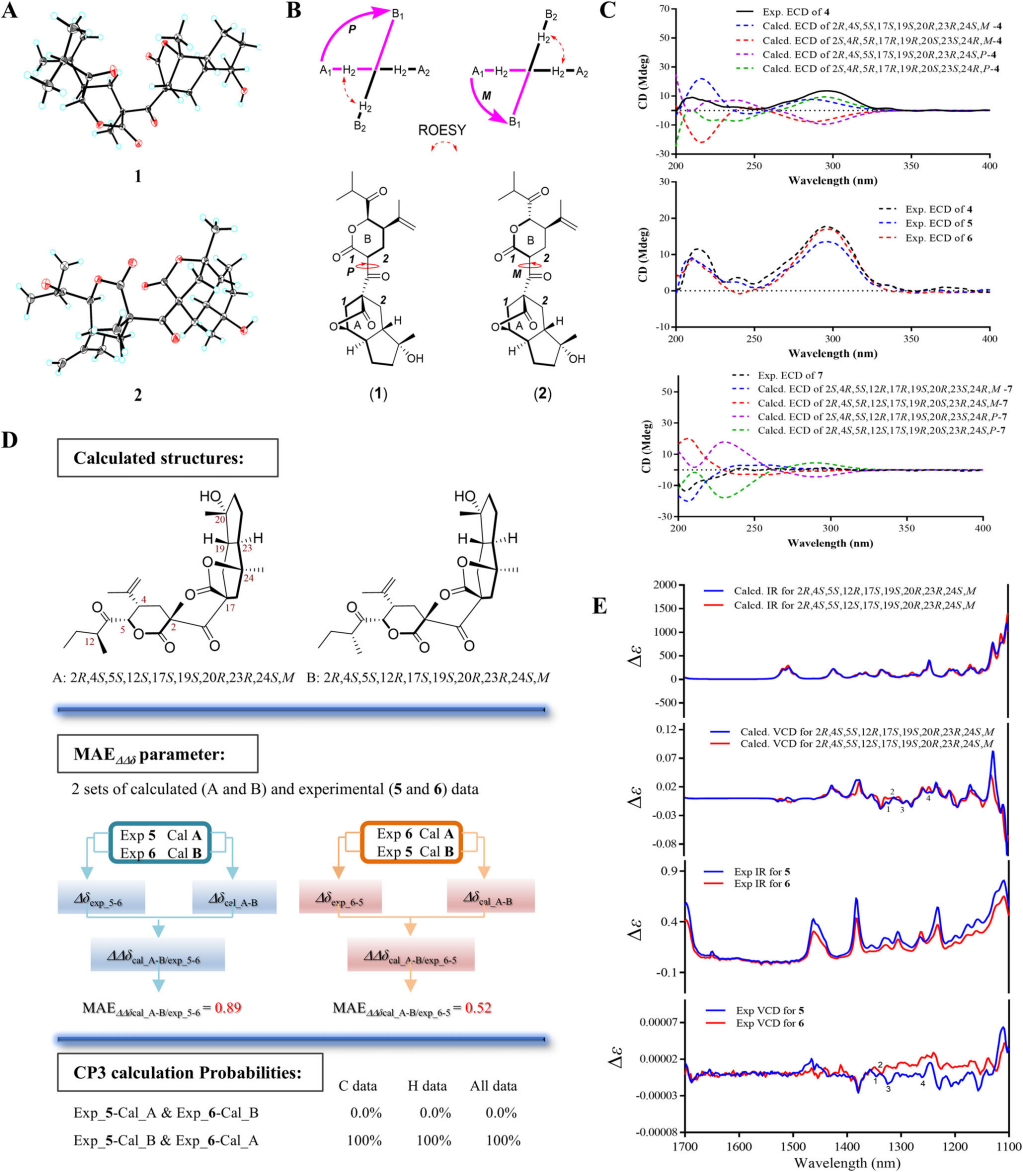
Configuration determination. (A) X‐ray crystal structures of compounds **1** and **2**. (B) Diagnostic ROESY correlations of hypermonols F (**1**) (2*S*,4*R*,5*R*,17*S*,19*S*,20*R*,23*R*,24*S*,*P*) and G (**2**) (2*S*,4*R*,5*S*,17*S*,19*S*,20*R*,23*R*,24*S*,*M*). (C) Comparison of experimental and calculated ECD spectra of compounds **4–7**. (D) CP3 analysis and MAE_ΔΔ_ *_δ_* parameters of compounds **5** and **6** based on experimental and calculated NMR chemical shifts. (E) Experimental and calculated VCD/IR spectra of compounds **5** and **6**.

The relative configuration of **1** was determined by ROESY analysis (Figure S3). The correlations of H‐5/H‐4, H‐4/H_3_‐7, and H‐5/H_3_‐7 indicated that H‐5, H‐4, and H_3_‐7 were located on the same face of the molecule and were therefore assigned as *β*‐oriented, placing the isobutyryl group at C‐5 and the 2‐propenyl group at C‐4 on the opposite (*α*) face. In unit B, the ROESY correlations of H‐19/H‐18b, H‐18b/H_3_‐27, H‐23/H‐18a, and H‐23/H_3_‐26, together with the large coupling constant between H‐19 and H‐23 (*J* = 12.0 Hz), established that H‐19 and H_3_‐27 were *β*‐oriented, whereas H‐23 and H_3_‐26 were *α*‐oriented. The planar structure and absolute configuration of **1** were unequivocally confirmed by single‐crystal X‐ray diffraction analysis using Cu K*α* radiation (CCDC 2340837, Figure 3A) [26].

The structures of compounds **2–7** were unambiguously confirmed by comprehensive spectroscopic analysis; detailed data are presented in the Supporting Information [27, 28, 29, 30, 31].

By comparison with previous reports, the known compounds were identified as chipericumin D (**8**) [32], longisglucinol B (**9**) [33], hyperpatulol I (**10**) [34], hyperpatulol C (**11**) [34], chipericumin E (**12**) [32], hyperpatulol D (**13**) [34], biyoulactone B (**14**) [35], furanmonogone A (**15**) [36], furanmonogone B (**16**) [36], hypermoin C (**17**) [37], hypermoin B (**18**) [37], hypermoin A (**19**) [37], hybeanone A (**20**) [38], and hyperhenone E (**21**) [39].

### 2.3 Identification of HPJ as a TNF Inhibitor

**FIGURE 4:**
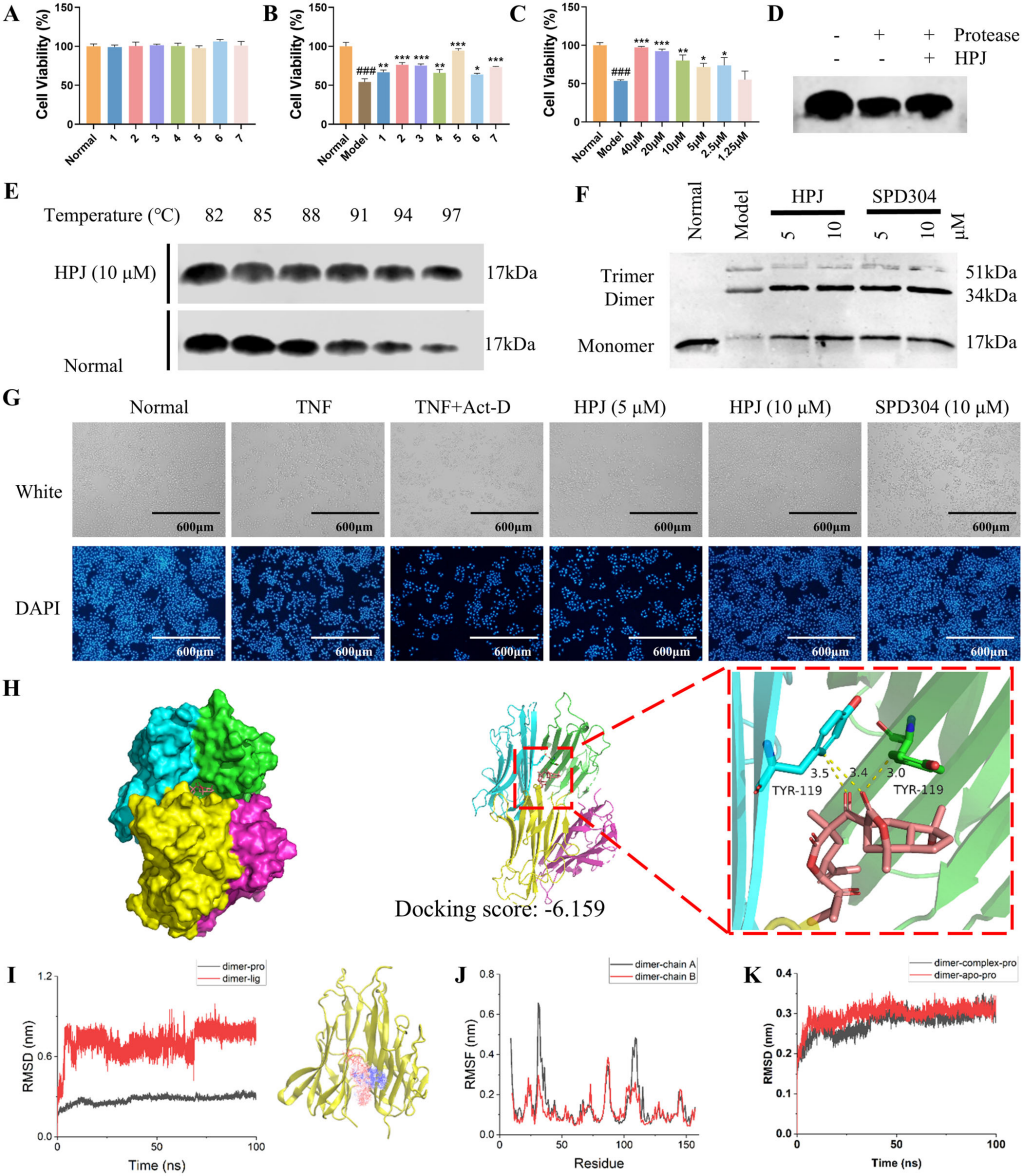
Targeted inhibition of HPJ against TNF. (A) Cytotoxicity of compounds **1–7** in L929 cells. (B) Cell viability of compounds **1–7** against TNF‐induced cytotoxicity in L929 cells. (C) Concentration‐dependent inhibition of TNF by HPJ. (D and E) DARTS and CETSA validation of HPJ‐induced TNF stabilization. (F) Cross‐linking analysis of TNF in the presence of HPJ and SPD304. (G) Morphological changes and DAPI staining of L929 cells treated with or without TNF and actinomycin D. (H) Molecular docking of HPJ with the TNF dimer. (I) RMSD plot of the TNF dimer and trajectory of HPJ after protein alignment. (J) RMSF plot of two chains of the TNF dimer. (K) RMSD comparison of the TNF dimer in the apo and HPJ‐bound states. Data are presented as mean ± SD. ^###^ *p* < 0.001 versus control; ^*^ *p* < 0.05, ^**^ *p* < 0.01, ^***^ *p* < 0.001 versus model.

Guided by SPR‐UPLC‐QTOF/MS analysis, compounds **1–7** were prioritized as potential TNF inhibitors for further validation. Their cytotoxicity (40 µM) was first evaluated in L929 cells using the Cell Counting Kit‐8 (CCK‐8) assay after 24 h of treatment, and no significant cell death was observed (Figure 4A). Given the high sensitivity of L929 cells to TNF, a TNF‐induced L929 cell model was used to assess inhibitory activity. Actinomycin D, which sensitizes L929 cells to TNF‐mediated cytotoxicity, was added at a low concentration to improve assay responsiveness. Among the tested compounds, compound **5** (HPJ) exhibited the strongest protective effect against TNF‐induced cytotoxicity in L929 cells (Figure 4B,C). HPJ also dose‐dependently reversed apoptotic morphology, including cell shrinkage, nuclear fragmentation, and chromatin condensation (Figure 4G). Based on these findings, HPJ was selected for further mechanistic evaluation.

Although compounds **1–7** were structurally related PPAP analogs, HPJ displayed the strongest cytoprotective activity, suggesting that subtle differences in stereochemistry and side‐chain architecture can substantially influence TNF recognition. Compared with the other analogs, HPJ possesses a C‐12*R* 2‐methylbutanoyl side chain together with a favorable caged‐core configuration, which may enhance hydrophobic complementarity and optimize its binding orientation at the TNF dimer interface. Therefore, the superior activity of HPJ is likely attributable to the cooperative effects of side‐chain stereochemistry, local hydrophobic interactions, and the overall three‐dimensional molecular architecture, rather than to any single structural feature.

Drug affinity responsive target stability (DARTS) is a small‐molecule screening and target‐identification technology [40, 41]. In the presence of HPJ, TNF exhibited increased stability (Figure 4D). To further validate this stabilization effect, fluorescence‐based thermal shift assays and cellular thermal shift assays were used to assess protein thermal stability [42]. In a native state, proteins maintain folded conformations; heating induces denaturation, exposing hydrophobic regions that bind the fluorescent dye SYPRO Orange, thereby amplifying fluorescence emission [43]. Compared with the DMSO control, HPJ attenuated the fluorescence response and increased TNF thermal stability (Figure S5). Drug binding typically stabilizes target proteins and delays thermal denaturation. As shown in Figure 4E, HPJ markedly reduced TNF degradation at 88°C, supporting HPJ‐induced stabilization of TNF. TNF exerts its biological functions by engaging TNFRs as a trimer, and disruption of trimer assembly can abolish cytokine activity [44]. Given the inhibitory effect of HPJ on TNF signaling, we hypothesized that HPJ blocks TNF activity by inhibiting functional trimer formation. To test this hypothesis, cross‐linking protein interaction assays were conducted. The results demonstrated that HPJ dose‐dependently promoted the accumulation of TNF dimer while concomitantly inhibiting the assembly of functional TNF homotrimers compared with the cross‐linked control group (Figure 4F).

To further investigate the binding mode of HPJ and reduce reliance on a single docking model, molecular docking was performed using the TNF dimer structure (PDB ID: 2AZ5). This template was selected because it is co‐crystallized with SPD304 [45], a reported small‐molecule TNF inhibitor used as the positive control in this study. HPJ was predicted to bind at the TNF dimer interface with a score of –6.159 kcal/mol, whereas SPD304 yielded a docking score of –4.402 kcal/mol under the same docking protocol. By comparison, docking of HPJ to the TNF trimer (PDB ID: 5YOY) and monomer (PDB ID: 3WD5) yielded less favorable docking scores of –3.382 and –3.115 kcal/mol, respectively. Consistent with these results, binding free energy calculations predicted a substantially more stable HPJ‐TNF dimer complex (Δ*G*_binding_ = –40.04 kcal/mol) than the corresponding trimer complex (Δ*G*_binding_ = –21.87 kcal/mol), suggesting that HPJ may preferentially stabilize the TNF dimer rather than the trimer. The predicted docking complexes were subsequently subjected to molecular dynamics (MD) simulations [46]. During the simulation, the TNF trimer rapidly stabilized after about 5 ns and remained compact, while HPJ consistently occupied the predicted dimer‐interface pocket throughout the trajectory (Figure 4I). Root mean square fluctuation (RMSF) analysis showed that Chain B of the complex was more stable than Chain A (Figure 4J). Root mean square deviation (RMSD) comparisons revealed that HPJ reduced conformational fluctuations of the dimer, but increased those of the trimer (Figure 4K and Figure S6). Collectively, these results support a model in which HPJ may preferentially stabilize the TNF dimer and interfere with formation of the biologically active trimer, thereby potentially attenuating TNF signaling [47].

To evaluate thefunctional selectivity, HPJ was evaluated in an LT‐α/actinomycin D‐induced WEHI164 cytotoxicity model. In contrast to its protective effect against TNF‐induced cytotoxicity in L929 cells, HPJ failed to rescue WEHI164 cells from LT‐α‐induced cell death, as demonstrated by both CCK‐8 viability assays and bright‐field/DAPI imaging (Figure S7). These data suggest that HPJ does not broadly suppress the cytotoxicity of TNF‐family cytokines and instead exhibits functional selectivity toward TNF over LT‐α under the experimental conditions examined.

To further substantiate the SPR‐derived interaction, ITC was performed as an orthogonal solution‐phase biophysical assay (Figure S8). ITC demonstrated that HPJ bound TNF with a *K*_D_ of 4.06 nM (*n* = 0.897, Δ*H* = –244.87, and *R* ^2^ = 0.996), whereas SPD304 showed a *K*_D_ of 30.3 nM (*n* = 0.986, Δ*H* = –46.21, and *R* ^2^ = 0.997). Although the absolute binding affinities measured by ITC were weaker than those obtained by SPR, both techniques consistently identified HPJ as the stronger TNF binder relative to SPD304. The *K*_D_ discrepancies may arise from methodological differences, including immobilized versus solution‐phase protein states, mass‐transport and rebinding effects in SPR, buffer and temperature variations, and fitting sensitivity limits for ultrahigh‐affinity interactions. Thus, ITC data provided orthogonal support for direct binding and relative potency without replacing SPR‐derived kinetic conclusions.

### 2.4 Functional and Pathway Enrichment of Differentially Expressed Genes

**FIGURE 5:**
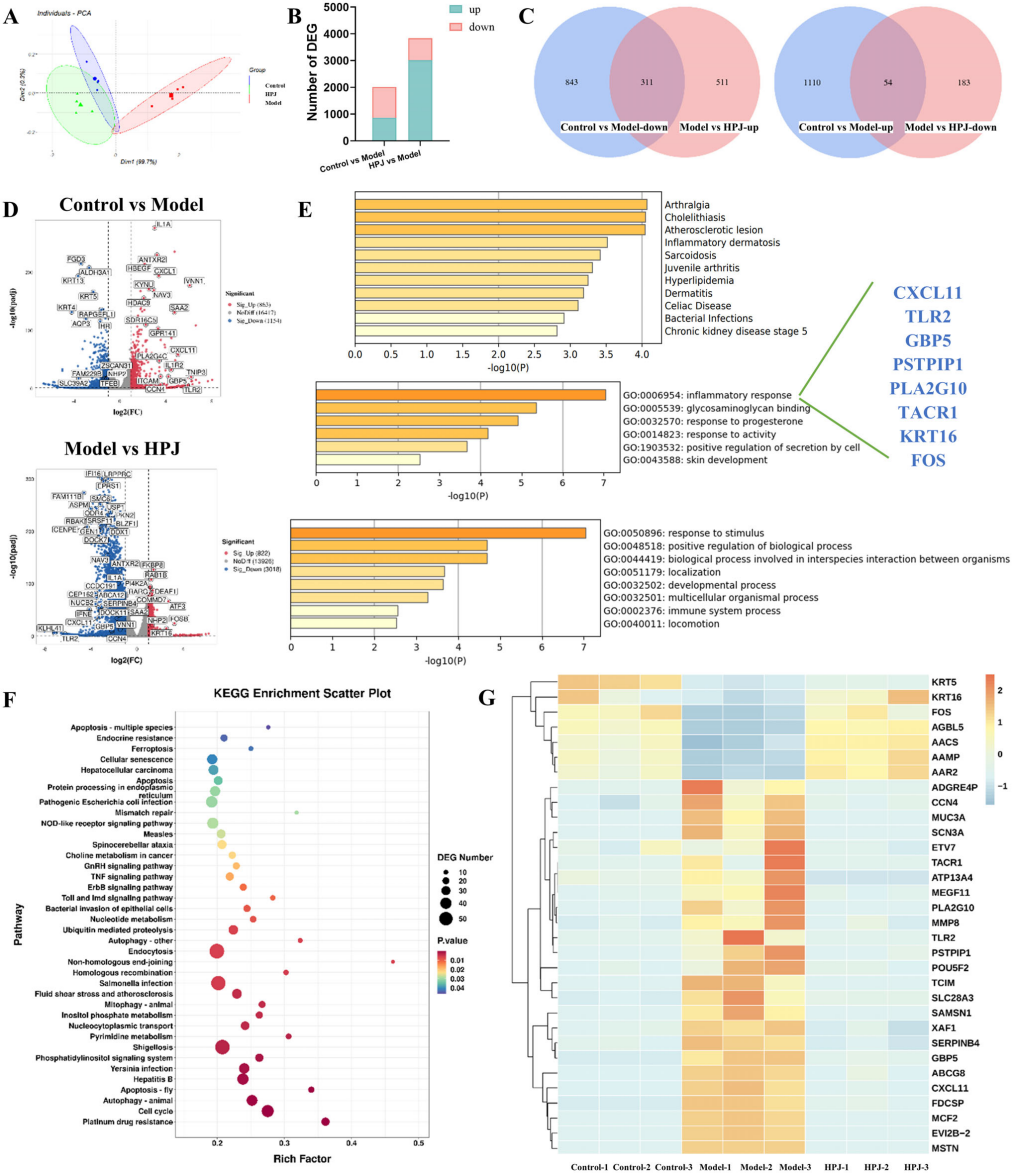
Differential gene expression and enrichment analysis. (A) PCA of control, model, and HPJ groups. (B and C) Differentially expressed genes and overlap analysis. (D) Volcano plots showing significantly differentially expressed genes (DEGs) between groups (*n* = 3). Red and blue dots indicate upregulated and downregulated DEGs, respectively. (E and F) GO and KEGG pathway enrichment analysis. (G) Heatmap of DEGs in control, model, and HPJ groups. In enrichment plots, circle size indicates the enrichment factor, and circle color reflects the *p*‐value.

Transcriptomic analysis of HaCaT cells showed group separation by principal component analysis (Figure 5A). The control versus model comparison identified 2017 differentially expressed genes (DEGs) (863 upregulated and 1154 downregulated), whereas the HPJ versus model comparison identified 3840 DEGs (3018 upregulated and 822 downregulated; Figure 5B). Overlap analysis identified 311 genes that were downregulated in the model and upregulated by HPJ, and 54 genes that were upregulated in the model and suppressed by HPJ (Figure 5C). The volcano plot highlighted IL1A, ANTXR2, CXCL11, TLR2, and CCN4, suggesting that HPJ treatment primarily influences anti‐inflammatory and immune‐related pathways (Figure 5D). GO enrichment analysis further supported this interpretation, with significant enrichment in inflammatory response, glycosaminoglycan binding, response to progesterone, cellular activity, positive regulation of secretion, and skin development (Figure 5E). KEGG analysis highlighted the TNF signaling pathway (Figure 5F), and heatmap analysis showed correlations among inflammation‐ and endothelial‐function‐related genes, including KRT5, KRT16, AAMP, CXCL11, and TLR2 (Figure 5G). Collectively, these results indicate that HPJ modulates transcriptional networks related to inflammation and immune regulation in HaCaT cells.

### 2.5 HPJ Attenuates TNF‐Induced Inflammatory Amplification In Vitro

**FIGURE 6:**
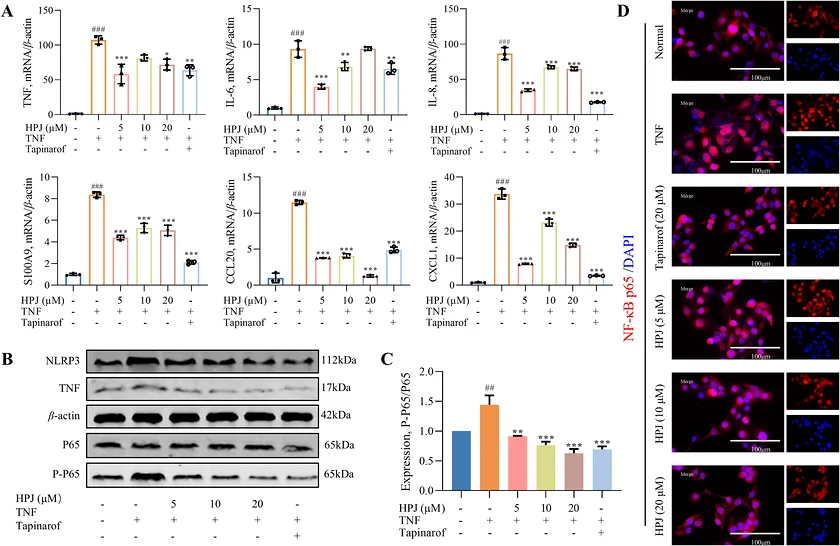
HPJ inhibited TNF‐induced inflammation in vitro. (A) Transcriptional changes of inflammatory cytokines in HaCaT cells stimulated with TNF. (B and C) Protein expression levels of NF‐κB p65, phosphorylated p65 (p‐p65), iNOS, NLRP3, and β‐actin in TNF‐stimulated HaCaT cells. (D) Immunofluorescence analysis of NF‐κB p65 nuclear translocation in TNF‐induced HaCaT cells. NF‐κB p65 is stained with an anti‐p65 antibody (red), and nuclei are counterstained with DAPI (blue). Data are presented as mean ± SD. ^#^ *p* < 0.05, ^##^ *p* < 0.01, ^###^ *p* < 0.001 versus control; ^*^ *p* < 0.05, ^**^ *p* < 0.01, ^***^ *p* < 0.001 versus model, *n* = 3.

To further evaluate the anti‐inflammatory effects of HPJ, RT‐qPCR and Western blot analyses were performed to examine changes in gene transcription and protein expression [48]. Excessive release of proinflammatory cytokines drives inflammation; therefore, suppressing cytokine overproduction is a key strategy for mitigating inflammatory responses [49]. As shown in Figure 6A, HPJ markedly reduced TNF‐induced expression of multiple inflammatory mediators, including TNF, interleukin‐6 (IL‐6), interleukin‐8 (IL‐8), C–C motif chemokine ligand 20 (CCL20), C–X–C motif chemokine ligand 1 (CXCL1), and S100 calcium‐binding protein A9 (S100A9). Consistently, protein levels of inducible nitric oxide synthase (iNOS), NOD‐like receptor thermal protein domain‐associated protein 3 (NLRP3), and the phosphorylation ratio of p‐p65/p65 were also decreased after HPJ treatment (Figure 6B,C). In addition, HPJ reduced TNFR1 and cleaved caspase‐3 levels compared with the TNF‐stimulated model group (Figure S9). These results provide cellular evidence that HPJ suppresses TNF‐driven TNFR1 signaling and apoptosis‐associated inflammatory amplification. NF‐κB is a pleiotropic transcription factor that regulates multiple biological processes, including inflammation and immunity, and p65 is a critical subunit capable of nuclear translocation during inflammatory activation [46, 50, 51]. Immunofluorescence analysis further showed that HPJ inhibited nuclear translocation of NF‐κB p65 (Figure 6D). Collectively, these results indicate that HPJ exerts anti‐inflammatory activity in vitro.

### 2.6 Pharmacokinetic and Safety Evaluation of HPJ

To assess systemic exposure of HPJ, a pharmacokinetic study was performed in mice following intravenous (i.v.) (5 mg/kg), intraperitoneal (i.p.) (10 mg/kg), and intragastric (i.g.) (20 mg/kg) administration. As shown in Table S4 and Figure S10, HPJ was rapidly absorbed via all three routes, reaching its maximum plasma concentration (*C*_max_) within 0.25–2 h. Following i.p. administration at the efficacious dose (10 mg/kg), the *C*_max_ was 5939.768 µg/L (∼12 µM) at 1 h, exceeding the in vitro active concentration. Absolute bioavailability was 84.9% for i.p. and 56.2% for i.g. administration, indicating encouraging systemic exposure.

### 2.7 HPJ Ameliorates Epidermal Hyperplasia and Other Symptoms of IMQ‐Induced Psoriasis in Mice

**FIGURE 7:**
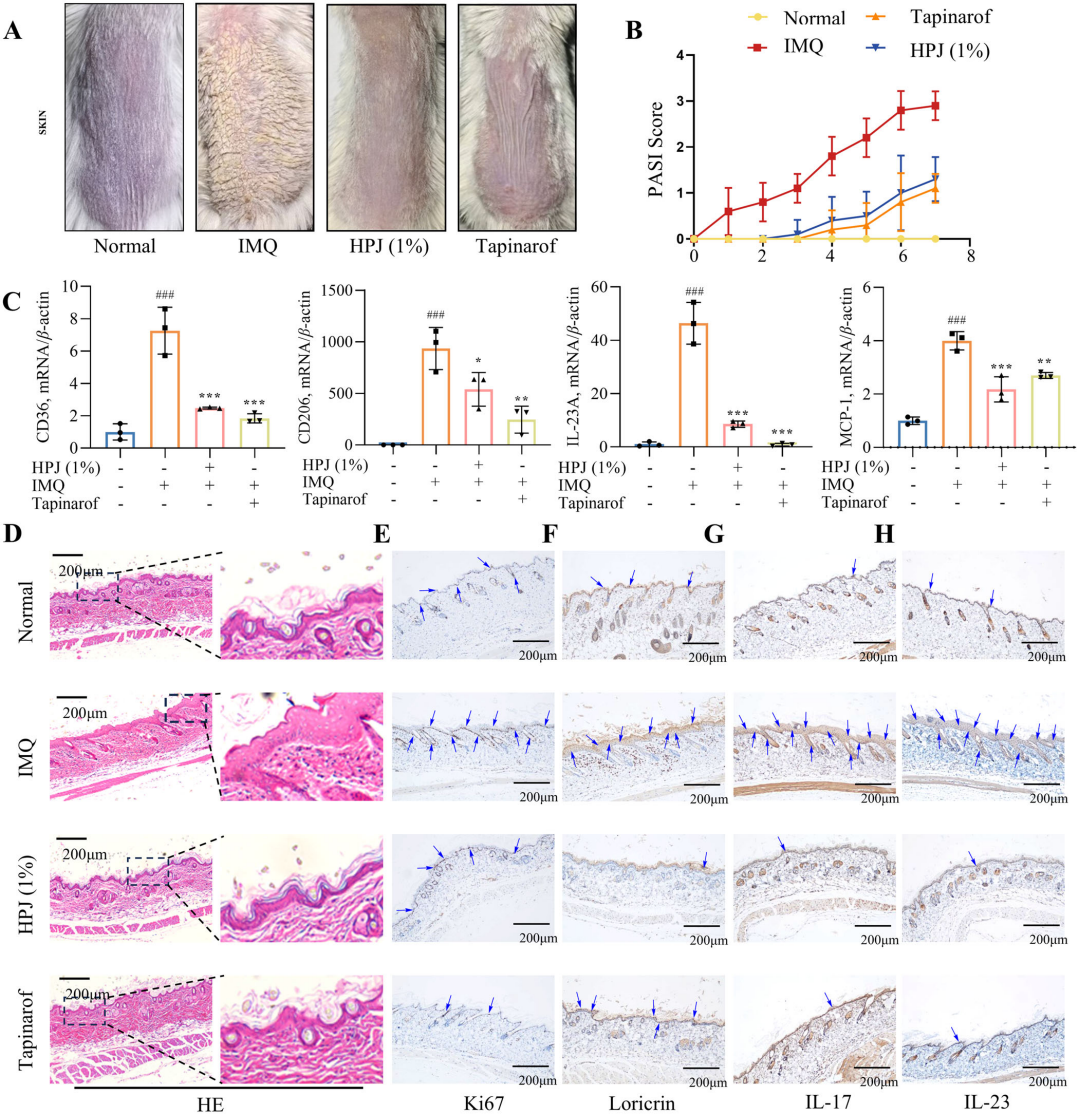
Therapeutic effects of HPJ in IMQ‐induced psoriatic mice. (A) Representative phenotypes of dorsal skin from mice after 7 days of IMQ application. (B) Quantification of PASI scores in mice for each group. (C) RT‐qPCR analysis of mRNA levels of CD36, CD206, IL‐23A, and MCP‐1 in skin biopsies. (D) H&E staining of cross‐sections of dorsal skin on Day 7. (E–H) Representative immunohistochemical analysis of key markers in skin lesions across groups. Data are presented as mean ± SD. ^#^ *p* < 0.05, ^##^ *p* < 0.01, ^###^ *p* < 0.001 versus control; ^*^ *p* < 0.05, ^**^ *p* < 0.01, ^***^ *p* < 0.001 versus model, *n* = 3. Tapinarof is used as a positive control, a small‐molecule aryl hydrocarbon receptor agonist with anti‐inflammatory effects. In an IMQ‐induced psoriasis‐like mouse model, topical tapinarof reduces epidermal thickening and inflammatory cytokine levels.

We next evaluated the clinical and histopathological manifestations of IMQ‐induced psoriasis in BALB/c mice. Disease progression was monitored daily using the Psoriasis Area and Severity Index (PASI) [52]. HPJ markedly delayed disease onset and alleviated the severity of IMQ‐induced psoriasis lesions on the dorsal skin, as reflected primarily by decreased erythema and scaling (Figure 7A). In the model group, mild psoriasis symptoms appeared on Day 1 and progressively worsened throughout the observation period, whereas HPJ‐treated mice developed only mild symptoms beginning on Day 3 (Figure 7B). In addition, HPJ significantly suppressed the expression of inflammatory mediators, including cluster of differentiation 36 (CD36), cluster of differentiation 206 (CD206), interleukin‐23 (IL‐23), and monocyte chemotactic protein‐1 (MCP‐1), without evidence of overt toxicity under the tested conditions (Figure 7C). Hematoxylin–eosin (H&E) staining on Day 7 further confirmed the protective effect of HPJ, revealing reduced thickness of both the stratum corneum and stratum spinosum, consistent with alleviation of epidermal hyperplasia (Figure 7D).

To rule out the possibility that the observed therapeutic effects were confounded by overt toxicity, a preliminary biosafety assessment was conducted. In the immune‐related cell lines THP‐1 and RAW264.7, HPJ caused neither a significant reduction in cell viability, as determined by the CCK‐8 assay, nor obvious morphological abnormalities in bright‐field or DAPI‐stained images within the tested concentration range (Figure S11A,B). Consistent with these in vitro findings, H&E staining of heart and spleen tissues from HPJ‐treated mice revealed no evident histopathological abnormalities compared with the control group (Figure S11C). These observations support the preliminary tolerability of HPJ under the conditions used in the efficacy studies.

Ki67 is a well‐established marker of cellular proliferation, whereas loricrin, a major structural component of the cornified envelope, is essential for epidermal barrier integrity [53, 54]. Immunohistochemical staining revealed that HPJ decreased the expression of both Ki67 and loricrin in the epidermis compared with the IMQ‐treated group (Figure 7E,F), suggesting that HPJ mitigates epidermal hyperplasia by suppressing excessive keratinocyte proliferation and abnormal epidermal differentiation.

IL‐17 is a pleiotropic cytokine primarily produced by CD4^+^ T cells that plays a central role in driving inflammatory responses [55]. During psoriatic inflammation, the heterodimeric cytokine IL‐23 sustains Th17 responses and amplifies IL‐17‐mediated inflammation by promoting the production of IL‐1 family cytokines and other proinflammatory mediators [56, 57]. Dysregulated activation of the IL‐23/IL‐17 axis initiates a self‐amplifying inflammatory cascade that is critical for psoriasis pathogenesis [58, 59]. Consistent with this mechanism, HPJ treatment significantly reduced the expression of both IL‐17 and IL‐23 (Figure 7G,H), indicating effective suppression of the IL‐23/IL‐17‐driven inflammatory axis associated with psoriasis.

### 2.8 HPJ Attenuates Inflammatory Storms in ALI Mouse Model

**FIGURE 8:**
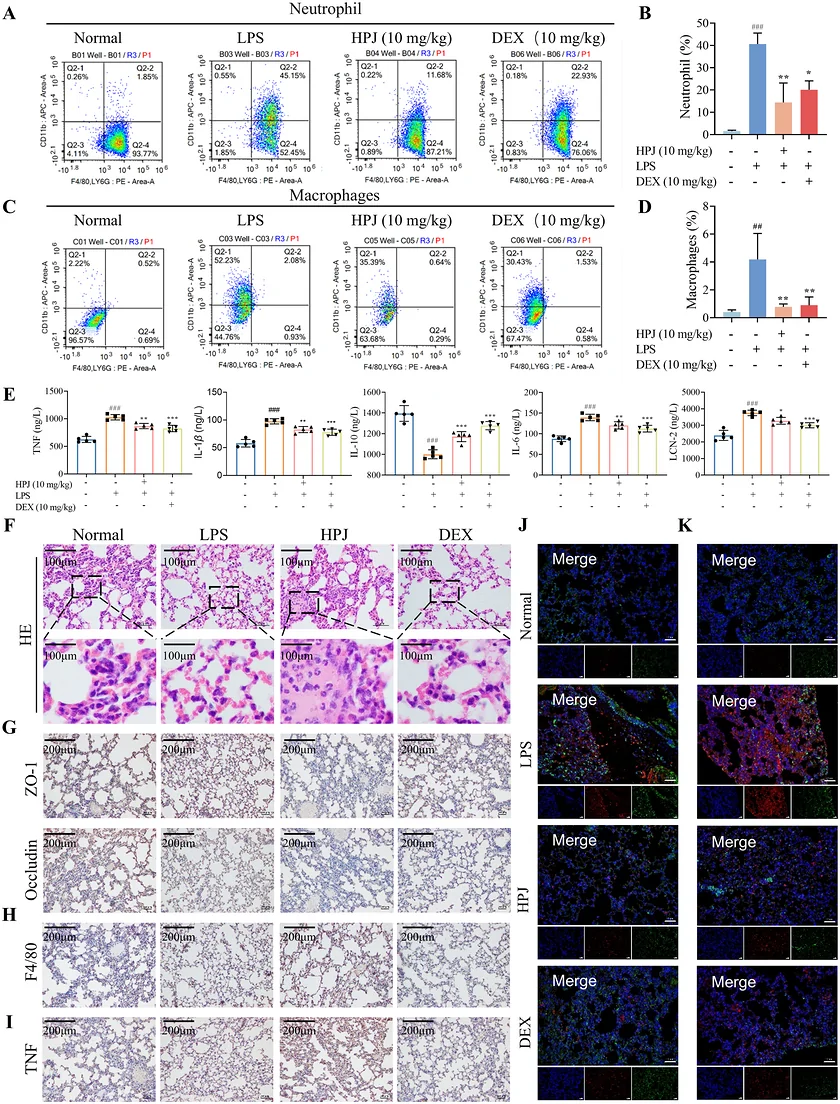
HPJ protects mice from LPS‐induced ALI. (A–D) Percentages of neutrophils and macrophages in bronchoalveolar lavage fluid. (E) Serum levels of TNF, IL‐1β, IL‐6, IL‐10, and LCN‐2 in mice treated with or without LPS and HPJ. (F) Representative H&E‐stained images of mouse lung sections. (G–I) Representative immunohistochemical images showing the expression of ZO‐1, Occludin, F4/80, and TNF in mouse lungs. Data are presented as mean ± SD. ^#^ *p* < 0.05, ^##^ *p* < 0.01, ^###^ *p* < 0.001 compared with control. (J) Immunofluorescence analysis of TNF (red), TNFR1 (green), and nuclei (DAPI, blue). (K) Immunofluorescence analysis of TNF (red), p65 (green), and nuclei (DAPI, blue). ^*^ *p* < 0.05, ^**^ *p* < 0.01, ^***^ *p* < 0.001, compared with model, *n* = 5. Dexamethasone (DEX) is a potent synthetic glucocorticoid that suppresses inflammatory responses by inhibiting NF‐κB and reducing proinflammatory cytokines. In an LPS‐induced ALI model, DEX attenuates pulmonary edema and pathological injury.

To evaluate the in vivo anti‐inflammatory efficacy of HPJ, an LPS‐induced ALI mouse model was established. Because neutrophils and macrophages play pivotal roles in amplifying cytokine secretion and driving inflammatory progression [47, 60], immune cell populations in bronchoalveolar lavage fluid (BALF), a clinically relevant indicator in lung disease, were analyzed. HPJ treatment significantly reduced the proportions of both neutrophils and macrophages in BALF (Figure 8A–D).

Proinflammatory cytokines, including TNF, IL‐6, and IL‐1β, are key mediators of inflammatory cascades. Accordingly, HPJ significantly decreased the serum levels of TNF, IL‐1β, IL‐6, and LCN‐2, while increasing the level of the anti‐inflammatory cytokine IL‐10, indicating effective suppression of excessive inflammatory responses (Figure 8E). H&E staining showed that HPJ‐treated mice exhibited alleviated pulmonary edema and leukocyte infiltration compared with LPS‐treated mice (Figure 8F). Consistent with these findings, immunostaining showed that HPJ restored the expression of the tight junction proteins ZO‐1 and occludin, both of which were markedly downregulated in LPS‐treated mice (Figure 8G). ZO‐1 is indispensable for maintaining paracellular barrier integrity, regulating signal transduction, and coordinating cell regulation [61, 62], whereas occludin is essential for tight junction assembly, stability, and epithelial barrier function [63]. Collectively, these findings suggest that HPJ may attenuate LPS‐induced ALI not only by suppressing excessive inflammatory responses but also by preserving pulmonary epithelial barrier integrity.

F4/80 immunostaining showed that LPS induced substantial macrophage infiltration into lung tissue [64], which was significantly reduced by HPJ treatment (Figure 8H). HPJ also markedly decreased TNF expression in lung tissue, supporting its potential to modulate TNF‐related inflammatory responses in the LPS‐induced ALI model (Figure 8I). To link HPJ‐mediated TNF stabilization to TNFR signaling, TNF/TNFR1 interaction and NF‐κB activation were examined by immunofluorescence and Western blotting. In TNF‐stimulated cells, TNF (red) colocalized with TNFR1 (green), and p65 translocated to the nucleus. HPJ treatment reduced the TNF/TNFR1‐associated fluorescence and inhibited p65 nuclear accumulation, consistent with impaired receptor engagement and downstream NF‐κB activation (Figure 8J,K).

## 3 Discussion and Future Directions

### 3.1 Summary of Major Findings

This study establishes an integrated SPR‑UPLC‑QTOF/MS‑GNPS platform for target‑directed natural product discovery and identifies hypermonol J (HPJ) from *H. monogynum* as a high‐affinity small‐molecule TNF binder. Mechanistically, HPJ may stabilize the TNF dimer and impair its assembly into the biologically active trimer, thereby attenuating downstream inflammatory signaling. In both IMQ‑induced psoriasis and LPS‑induced ALI models, HPJ exerted significant anti‑inflammatory efficacy, reducing epidermal hyperplasia, cytokine storms, and tissue injury while demonstrating preliminary tolerability under the experimental conditions examined. These results establish HPJ as a promising small‑molecule TNF‐binding lead and provide a foundation for its further pharmacological optimization and preclinical development.

### 3.2 Mechanistic Validation of HPJ‐TNF Interaction

HPJ‐TNF binding was supported by multiple complementary approaches, including SPR, ITC, DARTS/CETSA, cross‐linking assays, and analyses of the TNFR1/NF‐κB signaling pathway. SPR revealed an apparent picomolar‑level binding affinity (*K*_D_ = 25.9 pM), whereas the orthogonal ITC assay independently confirmed direct binding of HPJ to TNF in solution and preserved the same relative affinity ranking between HPJ and SPD304, despite differences in the absolute *K*_D_ values obtained by the two techniques. These discrepancies likely reflect inherent methodological differences, including immobilized versus solution‐phase protein states, mass‐transport and rebinding effects in SPR, variations in buffer composition and experimental temperature, and differences in fitting sensitivity for ultrahigh‐affinity interactions.

Molecular docking and MD simulations predicted that HPJ binds at the TNF dimer interface, whereas cross‐linking assays demonstrated increased dimer formation accompanied by reduced trimer assembly following HPJ treatment. DARTS and CETSA further indicated that HPJ stabilizes TNF, while cellular and tissue‐level analyses showed that HPJ treatment attenuated TNF/TNFR1‐dependent signaling and reduced nuclear accumulation of NF‐κB p65. Because docking scores are empirical and cytokine interfaces are intrinsically conformationally dynamic, the computational analyses were used to generate a plausible binding model rather than to define a definitive binding interface.

Together, these data support a working model in which HPJ favors a less productive TNF dimer‐associated state and thereby weakens trimer‐dependent receptor signaling.

### 3.3 Dimer Stabilization as a Therapeutic Strategy

Current clinically approved TNF inhibitors are predominantly biologics (e.g., adalimumab and infliximab) that neutralize soluble TNF trimers or prevent their interaction with TNF receptors. In contrast, stabilizing an inactive TNF dimer represents a mechanistically distinct and relatively underexplored therapeutic strategy. Cross‑linking assays, thermal shift analyses, and MD simulations collectively support preferential binding of HPJ at the TNF dimer interface and suggest that HPJ increases the energetic barrier to trimer assembly. This interpretation is consistent with the calculated binding free energies, in which the TNF dimer complex exhibited a substantially more favorable Δ*G*_binding_ (–40.04 kcal/mol) than the trimer complex (–21.87 kcal/mol). The predicted binding interface involves TYR119 from both TNF monomers, a residue previously implicated in stabilizing the trimeric assembly.

From a pharmacological perspective, dimer‑stabilizing small molecules may offer several potential advantages over conventional trimer‑neutralizing biologics, including the potential for oral optimization, lower risk of anti‐drug antibody formation, structural tunability, and improved tissue penetration due to their smaller molecular size.

### 3.4 In Vivo Efficacy in Psoriasis and Acute Lung Injury

HPJ demonstrated therapeutic efficacy in two distinct and clinically relevant inflammatory disease models. In the IMQ‑induced psoriasis model, topical application of 1% HPJ markedly reduced epidermal hyperplasia and suppressed the expression of IL‐17/IL‐23 axis‐related inflammatory markers. In the LPS‑induced ALI model, systemic administration of HPJ (10 mg/kg) significantly decreased immune cell infiltration, inflammatory cytokine production, and TNF expression in lung tissue while restoring the expression of the tight junction proteins ZO‐1 and occludin.

### 3.5 Selectivity, Pharmacokinetics, and Considerations Relative to Biologics

HPJ protected cells from TNF‑induced cytotoxicity but showed no comparable cytoprotective effect in the LT‐α‑induced WEHI164 assay. These findings support a functional preference for HPJ toward TNF‐mediated cellular responses under the tested experimental conditions.

Following intragastric administration, HPJ reached its *C*_max_ at 2 h and showed an oral absolute bioavailability of 56.2%, indicating favorable systemic exposure after oral dosing. Together with its therapeutic efficacy in the ALI model, these findings highlight the promising pharmacokinetic and in vivo pharmacological properties of HPJ.

### 3.6 Platform Value: SPR‑UPLC‑QTOF/MS‑GNPS as a Generalizable Strategy

A key methodological contribution of this work is the integration of SPR‐AIRS with UPLC‑QTOF/MS and GNPS molecular networking. Traditional bioassay‑guided fractionation is indirect because fractions are tested for activity without prior knowledge of the target‐binding constituents. In contrast, this platform directly retrieves TNF‑bound compounds from a complex crude extract, thereby reducing purification time and the likelihood of false‑positive hits. GNPS molecular networking further enables rapid dereplication and structural annotation, allowing prioritization of previously undescribed PPAPs over known compounds.

This strategy may be adaptable to other soluble protein targets and provides a practical route for linking botanical chemical diversity to molecular mechanisms.

### 3.7 Limitations and Future Directions

Several limitations and future directions should be considered together. First, the HPJ‐TNF dimer‐interface model remains experimentally supported, but not yet structurally definitive; docking scores are empirical estimates, and cytokine interfaces are conformationally flexible. High‐resolution crystallography, cryo‐EM, HDX‐MS, mutagenesis, or TNF‐TNFR1 competition assays will be required to define the precise binding interface. Second, the absolute KD values differed between SPR and ITC, likely because of immobilized versus solution‐phase protein states, mass‐transport or rebinding effects in SPR, buffer and temperature variation, and fitting sensitivity for ultrahigh‐affinity interactions. Third, the LT‐α/WEHI164 assay supports assay‐context‐dependent functional selectivity rather than absolute TNF specificity. Fourth, the animal experiments show anti‐inflammatory efficacy and reduced TNF/TNFR1‐associated signaling, but do not provide direct biochemical proof of in vivo HPJ‐TNF engagement. Finally, the proposed translational advantages of HPJ as a small‐molecule TNF binder remain to be validated by studies of formulation, tissue distribution, metabolic stability, repeated‐dose toxicity, chronic safety, additional autoimmune models, and direct pharmacodynamic comparisons with established TNF inhibitors.

## 4 Conclusion

This study identifies and validates HPJ as a small‐molecule TNF‐binding lead discovered through an integrated SPR‐UPLC‐QTOF/MS‐GNPS‐guided screening strategy. SPR and ITC results demonstrated high‐affinity binding of HPJ to TNF, while complementary biochemical and cellular assays support a working model in which HPJ stabilizes the TNF dimer and interferes with assembly of the biologically active trimer required for efficient receptor engagement. Multilayered functional characterization further demonstrated that HPJ suppresses NF‐κB signaling in vitro and attenuates cytokine‐driven inflammatory responses in vivo, leading to significant therapeutic benefits in both IMQ‐induced psoriasis and LPS‐induced ALI models. Collectively, these findings establish HPJ as a promising small‐molecule TNF‐binding lead with functional preference toward TNF under the experimental conditions examined.

Beyond HPJ identification, this work demonstrates the utility of a target‐oriented natural product discovery platform that connects complex botanical extracts with disease‐relevant molecular targets and may facilitate broader discovery of bioactive natural products from herbal resources.

## 5 Materials and Methods

Unless otherwise specified, all chemical reagents and solvents were obtained from commercial suppliers and used without further purification. Key experimental procedures included SPR‐based recovery of TNF‐bound compounds; UPLC‐QTOF/MS and GNPS‐based molecular networking; structural elucidation by NMR, ECD, VCD, and X‐ray crystallography; SPR, ITC, DARTS, CETSA, and cross‐linking assays for TNF interaction validation, cell‐based TNF pathway assays; pharmacokinetic analysis; and IMQ‐induced psoriasis and LPS‐induced ALI animal models. Comprehensive experimental protocols are provided in the Supporting Information.

For the in vivo efficacy studies, the IMQ‐induced psoriasis model was performed using *n* = 3 mice per group, whereas the LPS‐induced ALI model was performed using *n* = 5 mice per group, unless otherwise specified in the corresponding figure legends. Mice were randomly assigned to treatment groups. PASI scoring, histological analysis, and immunostaining were performed by investigators blinded to group allocation where applicable. No animals were excluded. No formal a priori power calculation was performed; sample sizes were selected based on preliminary pharmacodynamic observations, expected effect sizes, and ethical considerations aimed at minimizing animal use.

## Author Contributions

Study conception and design were collaboratively developed by Zhengxi Hu, Yonghui Zhang, Jiangchun Wei, Xiaotian Zhang, Weiguang Sun, and Yongqi Li. Data acquisition was carried out by Yongqi Li, Xingpiao Jin, Pingping Fan, Yahui Huang, Lanqin Li, Chaohu Xiong, and Jiangchun Wei. Jinsheng Wang extracted the data and performed statistical analysis. Xingpiao Jin accessed and validated the data. Yongqi Li and Jiangchun Wei completed the writing of the initial draft. Zhengxi Hu and Jinsheng Wang revised and polished the manuscript. All authors contributed to data interpretation, writing, and manuscript review and are fully accountable for data integrity and the decision to publish. All authors have read and approved the final manuscript.

## Funding

This work was supported by the Hubei Provincial Natural Science Foundation of China (No. 2024AFA028), the Wuhan International Science and Technology Cooperation Project (No. 2025071204030392), the National Natural Science Foundation of China (Nos. 82404822, 82273811, and 22577033), the National Key R&D Program of China (No. 2021YFA0910500), the Fundamental Research Funds for the Central Universities (No. 2025BRA015), the Program for Higher Education Young Teachers' Scientific Research Innovation Capability Support of Higher Education Institutions in Shanxi (No. 2025Q030), the Traditional Chinese Medicine Scientific Research Project of Shanxi Provincial Health Commission (No. 2025ZYYA036), and the Key Project of the 1+1 Scientific Research Cooperation Program of Changzhi Medical College (No. HZZD202406).

## Disclosure

Author Yonghui Zhang is an editorial board member of MedComm. Yonghui Zhang was not involved in the journal's review of, or decisions related to, this manuscript.

## Ethics Statement

This study was approved by the Ethics Committee for Laboratory Animals of Huazhong University of Science and Technology (20233407).

## Conflicts of Interest

The authors declare no conflicts of interest.

## Supporting information




**Supporting File**: mco270960‐sup‐0001‐SuppMat.pdf.

## Data Availability

The raw NGS data generated in this study have been deposited in the Genome Sequence Archive for Human (GSA‐Human) of the China National Center for Bioinformation/Beijing Institute of Genomics, Chinese Academy of Sciences (CNCB‐NGDC), under the accession number HRA019030. The associated BioProject accession number is PRJCA066335. The dataset has been released and is publicly available at: https://ngdc.cncb.ac.cn/gsa‐human/browse/HRA019030.
