# Supplementary material for: SPR‐UPLC‐QTOF/MS‐GNPS‐Guided Discovery of Hypermonol J as a Novel Tumor Necrosis Factor‐Binding Small‐Molecule Lead for Inflammatory Storm Intervention
Source: MedComm (2020). 2026 Aug 28;7(9):e70960. doi: 10.1002/mco2.70960 (PMC13522848; doi:10.1002/mco2.70960)
Supplement: Supplementary file 1 [file MCO2-7-e70960-s001.pdf]

Supplementary Materials

SPR-UPLC-QTOF/MS-GNPS-Guided Discovery of Hypermonol J as a Novel Tumor Necrosis Factor-Binding Small-Molecule Lead for Inflammatory Storm Intervention

Yongqi Li *et al.*

*Corresponding author. Emails: hzx616@126.com (Zhengxi Hu); zhangyh@mails.tjmu.edu.cn (Yonghui Zhang); jiangchun_w@126.com (Jiangchun Wei); zhangxt997@126.com (Xiaotian Zhang)

This PDF file includes:

Supplementary Text S1 to S34
Figs. S1 to S88
Tables S1 to S13
Supplementary Source Data S1 to S11
References (1 to 3)

Supplementary results section

S1. Structural elucidation of compounds 2–7

Hypermonol G (**2**) was also isolated as colorless crystals and possessed the same molecular formula, $C_{26}H_{36}O_7$, as compound **1**. Detailed comparison of the 1D and 2D NMR spectroscopic data (Table S1, Figure S2) revealed that **1** and **2** shared the same planar structure. The nearly identical chemical shifts observed for unit B, together with the noticeable differences in unit A, suggested that the two compounds differed only in the relative configuration of unit A. ROESY correlations of H-4/H_3-7 indicated that these protons were *β*-oriented, whereas the correlations of H-5/H_3-10 and H-5/H_2-9 (Figure S3), in combination with the large coupling constant between H-4 and H-5 (J = 10.8 Hz), established the *α*-orientation of H-5 and the 2-propenyl substituent at C-4.

After repeated crystallization attempts, suitable single crystals of **2** were finally obtained from a MeOH/H_2O (10:1, v/v) solution. The absolute configuration was readily assigned based on the X-ray diffraction data, which gave a Flack parameter of 0.08(18) (CCDC 2340838, Figure 3A). Notably, both the single-crystal X-ray diffraction results and the ROESY correlations (H-3/H-18 and H-3/H-25) observed for **1** and **2** suggested restricted rotation around the carbonyl bridge, indicating that both compounds exist as single atropisomers (Figure 3B). Accordingly, the absolute configurations of **1** and **2** were determined as 2*S*,4*R*,5*R*,17*S*,19*S*,20*R*,23*R*,24*S*,*P* and 2*S*,4*R*,5*S*,17*S*,19*S*,20*R*,23*R*,24*S*,*M*, respectively.

Hypermonol H (**3**) was obtained as a white amorphous powder. Its molecular formula and planar structure were found to be identical to those of **2**. However, unlike **2**, which exhibited a large coupling constant between H-19 and H-23 (J = 12.0 Hz), compound **3** showed a much smaller coupling constant for these protons, suggesting a different relative configuration in unit B. This assignment was further supported by ROESY correlations of H-18b/H-19, H-19/H-23, H-23/H_3-27, H_2-25/H_3-26, and H-3b/H-18b (Figure S3), which established **3** as the C-23 epimer of **2**.

Hypermonol I (**4**) possessed the same molecular formula as **3**. Its 1D NMR spectroscopic data (Table S2) closely resembled those of **3**, except for noticeable differences in the resonances of H_2-3/C-3, H-4/C-4, H-5/C-5, and H_3-7/C-7, suggesting that **4** differed from **3** in the stereochemistry of unit A. This hypothesis was supported by the ROESY correlations of H_3-7/H-4, H_3-7/H-5, and H-4/H-5 (Figure S3), together

with the relatively small coupling constant between H-4 and H-5 (J = 6.0 Hz), which established the relative configuration of unit A. The absolute configuration of **4** was subsequently determined by comparison of its experimental and calculated ECD spectra (Figure 3C), leading to the assignment of the stereochemistry as 2*R*,4*S*,5*S*,17*S*,19*S*,20*R*,23*R*,24*S*,*M*.

Hypermonols J (**5**) and K (**6**) were assigned the molecular formula $C_{27}H_{38}O_7$ based on their HRESIMS and ^{13}C NMR data, corresponding to an increase of 14 mass units relative to compound **4**. Comparison of their NMR spectroscopic data (Table S2) with those of **4** revealed that the 2-methylpropanoyl substituent at C-5 in **4** was replaced by a 2-methylbutanoyl group in **5** and **6**. Detailed analysis of the 2D NMR data established that **5** and **6** possessed the same planar structure and differed only in the configuration at C-12, indicating that they are C-12 epimers.

The relative configurations of **5** and **6** were found to be identical to that of **4**, as supported by their ROESY correlations and highly similar NMR spectroscopic features. In addition, their experimental ECD spectra closely matched the calculated curves (Figure 3C), supporting the same absolute configuration for the common stereogenic centers. Because ECD calculations were unable to discriminate between the two possible configurations at C-12, VCD calculations were subsequently performed. Geometry optimizations and VCD spectral simulations were carried out at the B3LYP/6-311+G(d) level for the two candidate stereoisomers. Comparison of the experimental and calculated VCD spectra (Figure 3E) enabled assignment of the absolute configurations of **5** and **6** as 2*R*,4*S*,5*S*,12*R*,17*S*,19*S*,20*R*,23*R*,24*S*,*M* and 2*R*,4*S*,5*S*,12*S*,17*S*,19*S*,20*R*,23*R*,24*S*,*M*, respectively.

To further validate the configurational assignment of the C-12 epimers, ^{1}H and ^{13}C NMR chemical shifts were calculated for two candidate structures (A and B) at the mPW1PW91/6-311+G(d,p)//ωB97XD/6-31G(d) level in chloroform. Statistical analyses, including correlation coefficient (R^2) evaluation (Figure S4), $MAE_{\Delta\Delta\delta}$ analysis, and CP3 probability calculations, showed the best agreement between structure B and compound **5**, and between structure A and compound **6** (Figure 3D). These results independently confirmed the stereochemical assignments of **5** and **6**.

Hypermonol L (**7**) was found to possess the same planar structure as **5** and **6**, as established by comprehensive analysis of its HRESIMS, 1D NMR, and 2D NMR spectroscopic data (Table S3). Its relative configuration was determined from key

ROESY correlations and coupling constant analysis. ROESY cross-peaks of H_3-7 (δ_H 1.58)/H-3a (δ_H 1.55)/H-4 (δ_H 2.85), H-5 (δ_H 5.38)/H-3b (δ_H 3.00)/H-18a (δ_H 2.08), H-23 (δ_H 2.29)/ H_3-26 (δ_H 1.43), and H-18b (δ_H 2.37)/H-25b (δ_H 1.95)/H-23 (δ_H 2.29)/H-21b(δ_H 1.58)/H-19(δ_H 2.19)/ H_3-27 (δ_H 1.21), together with the large coupling constants between H-4 and H-5 (J = 12.0 Hz), established its relative stereochemistry. The absolute configuration of **7** was subsequently assigned by comparison of its experimental and calculated ECD spectra (Figure 3C), leading to the stereochemical assignment of 2*S*,4*R*,5*S*,12*R*,17*R*,19*S*,20*R*,23*S*,24*R*,*M*.

S2. Physical data of 1–7

Hypermonol F (**1**): colorless crystals; mp 153–155 °C; $[\alpha]_D^{25}$: –48.3 (MeOH, *c* 0.1); UV (MeOH) λ_{max} (log ε): 201 (4.29) nm; ECD (MeOH) λ_{max} ($\Delta\varepsilon$): 201 (+9.36), 209 (+8.12), 220 (+12.56), 297 (–12.04) nm; IR (KBr) ν_{max}: 3408, 1968, 2921, 2851, 1762, 1734, 1466, 1382, 1104, 914 cm^{-1}; For ^{1}H and ^{13}C NMR data, see Table 1; HRESIMS *m*/*z* 483.2352 ($[M + Na]^+$, calcd for $C_{26}H_{36}O_7Na^+$, 483.2359).

Hypermonol G (**2**): colorless crystals; mp 126–127 °C; $[\alpha]_D^{25}$: +41.5 (MeOH, *c* 0.1); UV (MeOH) λ_{max} (log ε): 202 (4.36) nm; ECD (MeOH) λ_{max} ($\Delta\varepsilon$): 201 (+24.42) nm, 287 (–5.58) nm; IR (KBr) ν_{max}: 3365, 2920, 2850, 1759, 1726, 1958, 1468, 1381, 1237, 1125, 917 cm^{-1}; For ^{1}H and ^{13}C NMR data, see Table 1; HRESIMS *m*/*z* 483.2350 ($[M + Na]^+$, calcd for $C_{26}H_{36}O_7Na^+$, 483.2359).

Hypermonol H (**3**): colorless oil; $[\alpha]_D^{25}$: +124.7 (MeOH, *c* 0.1); UV (MeOH) λ_{max} (log ε): 201 (4.43) nm; ECD (MeOH) λ_{max} ($\Delta\varepsilon$): 202 (+6.72), 283 (–3.91) nm; IR (KBr) ν_{max}: 3433, 1968, 2923, 2852, 1761, 1725, 1459, 1383, 1123, 1015, 899 cm^{-1}; For ^{1}H and ^{13}C NMR data, see Table 2; HRESIMS *m*/*z* 483.2358 ($[M + Na]^+$, calcd for $C_{26}H_{36}O_7Na^+$, 483.2359).

Hypermonol I (**4**): colorless oil; $[\alpha]_D^{25}$: +134.4 (MeOH, *c* 0.1); UV (MeOH) λ_{max} (log ε): 202 (4.27) nm; ECD (MeOH) λ_{max} ($\Delta\varepsilon$): 214 (+11.67), 246 (+2.48), 296 (+17.67) nm; IR (KBr) ν_{max}: 3443, 2971, 2391, 1765, 1725 cm^{-1}; For ^{1}H and ^{13}C NMR data, see Table 2; HRESIMS *m*/*z* 483.2352 ($[M + Na]^+$, calcd for $C_{26}H_{36}O_7Na^+$, 483.2359).

Hypermonol J (**5**): colorless oil; $[\alpha]_D^{25}$: +42.5 (MeOH, *c* 0.1); UV (MeOH) λ_{max} (log ε): 202 (4.38) nm; ECD (MeOH) λ_{max} ($\Delta\varepsilon$): 210 (+8.99), 245 (+1.15), 296 (+13.58) nm; IR (KBr) ν_{max}: 3431, 2921, 2851, 1764, 1737, 1697, 1658, 1467, 1381, 1109, 913 cm^{-1}; For ^{1}H and ^{13}C NMR data, see Table 3; HRESIMS *m*/*z* 497.2511 ($[M + Na]^+$, calcd for $C_{27}H_{38}O_7Na^+$, 497.2515).

Hypermonol K (**6**): colorless oil; $[\alpha]_D^{25}$: +119.2 (MeOH, *c* 0.1); UV (MeOH) λ_{max} (log ε): 201 (4.55) nm; ECD (MeOH) λ_{max} ($\Delta\varepsilon$): 212 (+8.25), 241 (−0.68), 97 (+17.06) nm; IR (KBr) ν_{max}: 3444, 2967, 2921, 1765, 1738, 1647, 1465, 1381, 1232, 1109, 913 cm^{-1}; For ^{1}H and ^{13}C NMR data, see Table 3; HRESIMS *m/z* 497.2517 ($[M + Na]^+$, calcd for $C_{27}H_{38}O_7Na^+$, 497.2515).

Hypermonol L (**7**): colorless oil; $[\alpha]_D^{25}$: +6.4 (MeOH, *c* 0.1); UV (MeOH) λ_{max} (log ε): 201 (4.36) nm; ECD (MeOH) λ_{max} ($\Delta\varepsilon$): 205 (–13.52) nm; IR (KBr) ν_{max}: 3444, 2965, 2928, 2854, 1762, 1726, 1704, 1458, 1208, 1137 cm^{-1}; For ^{1}H and ^{13}C NMR data, see Table 3; HRESIMS *m/z* 497.2510 ($[M + Na]^+$, calcd for $C_{27}H_{38}O_7Na^+$, 497.2515).

S3. Single-crystal X-ray diffraction analysis

Crystal data for compound **1** (CCDC No. 2340837): M = $C_{26}H_{36}O_7$, M = 460.55, a = 9.8213(4) Å, b = 11.0665(4) Å, c = 22.3002(8) Å, α = 90°, β = 90°, γ = 90°, V = 2423.75(16) $Å^3$, T = 110.0(2) K, space group $P2_12_12_1$, Z = 4, μ(Cu Kα) = 0.741 mm^{-1}, 24864 reflections measured, 4757 independent reflections (R_{int} = 0.0454). The final R_1 values were 0.0322 ($I > 2\sigma(I)$). The final $wR(F^2)$ values were 0.0843 ($I > 2\sigma(I)$). The final R_1 values were 0.0323 (all data). The final $wR(F^2)$ values were 0.0844 (all data). The goodness of fit on F^2 was 1.073. Flack parameter = 0.05(3).

Crystal data for compound **2** (CCDC No. 2340838): $C_{26}H_{36}O_7$, M = 460.55, a = 15.6186(19) Å, b = 8.2171(9) Å, c = 20.348(3) Å, α = 90°, β = 106.211(7)°, γ = 90°, V = 2507.6(5) $Å^3$, T = 150.0(2) K, space group P1211, Z = 4, μ(Cu Kα) = 0.716 mm^{-1}, 58479 reflections measured, 9193 independent reflections (R_{int} = 0.2273). The final R_1 values were 0.0776 ($I > 2\sigma(I)$). The final $wR(F^2)$ values were 0.1954 ($I > 2\sigma(I)$). The final R_1 values were 0.1853 (all data). The final $wR(F^2)$ values were 0.2455 (all data). The goodness of fit on F^2 was 0.978. Flack parameter = 0.08(18).

Supplementary Experimental Section

S4. General experimental procedures

Optical rotations and UV spectra were recorded on a PerkinElmer 341 polarimeter (PerkinElmer Inc., Fremont, California, USA) and a Lambda 35 instrument (PerkinElmer Inc., Fremont, California, USA), respectively. IR spectra were acquired using a Bruker Vertex 70 FT-IR spectrophotometer (Bruker, Karlsruhe, Germany). Experimental ECD data were collected on a JASCO J-810 spectrometer (JASCO, Tokyo, Japan). NMR spectra were recorded on a Bruker AM-600 or AM-400 spectrometers using TMS as the internal standard. All chemical shifts (δ) were expressed in ppm relative to the solvent signal (chloroform-*d*: δ_H 7.26, δ_C 77.16). HRESIMS data were acquired in the positive-ion mode on a Bruker micrOTOF-Q II spectrometer (Bruker, Karlsruhe, Germany). Column chromatography (CC) was performed using silica gel (200–300 mesh, Qingdao Marine Chemical, Inc., Qingdao, China), Sephadex LH-20 (40–70 μm, Amersham Pharmacia Biotech AB, Uppsala, Sweden), and octadecylsilyl (ODS, 50 μm, YMC Co., Ltd., Kyoto, Japan). Preparative HPLC was performed on a Sanotac system (Shanghai Sanotac Scientific Instruments Co., Ltd., Shanghai, China) equipped with a UV detector and a YMC C_{18} column (250 × 20 mm, 5 μm, YMC, Kyoto, Japan). Semi-preparative HPLC was conducted on an Agilent 1100 liquid chromatograph equipped with a reversed-phase (RP) C_{18} column (250 × 10 mm, 5 μm, Welch Materials, Inc., Shanghai, China). Thin-layer chromatography (TLC) was carried out using silica gel 60 F_{254} plates (Yantai Chemical Industry Research Institute, Yantai, China). Fractions were monitored by TLC, and spots were visualized by spraying the plates with 10% H_2SO_4 in EtOH followed by heating.

S5. Plant material

The aerial parts of *H. monogynum* were collected from Shennongjia Forestry District, Hubei Province, China, in September 2022. The plant material was authenticated by Professor Jianping Wang of Huazhong University of Science and

Technology. A voucher specimen (No. HB20220930) has been deposited in the herbarium of the School of Pharmacy, Tongji Medical College, Huazhong University of Science and Technology.

S6. Extraction and isolation

The air-dried aerial parts of *H. monogynum* (20 kg) were pulverized and extracted with 95% EtOH (60 L per extraction, 3 days per cycle) at room temperature for three consecutive cycles. The combined EtOH extracts were concentrated under reduced pressure, with the evaporation temperature maintained below 50 °C, to afford a crude residue (1320 g). The residue was suspended in H_2O and partitioned three times with petroleum ether, yielding a petroleum ether extract (310 g). This extract was subjected to silica gel CC and eluted with petroleum ether/EtOAc (10:1, 5:1, 3:1, 2:1, 1:1, and 1:2, v/v) to afford six major fractions (A–F).

Active screening results indicated that extract fractions A–F exhibited potential TNF-inhibitory activity, with Fraction C showing the strongest inhibition and Fraction D showing the next strongest effect. To identify the active components responsible for TNF binding, Fractions C and D were subjected to detailed isolation. Fraction C (113 g) was further chromatographed on an MCI gel column (10 × 100 cm) using stepwise elution with CH_3OH/H_2O (30:70, 50:50, 70:30, and 90:10, v/v), affording four subfractions (C1–C4). Subfraction C3 (18.4 g) was purified using Sephadex LH-20 eluted with CH_3OH/CH_2Cl_2 (1:1, v/v) to yield seven subfractions (C3-1–C3-7). Subfraction C3-2 (7.6 g) was separated by medium-pressure liquid chromatography (MPLC) over octadecylsilane (ODS), using a stepwise gradient of CH_3OH/H_2O (50:50 to 90:10, v/v) over 10 h at a flow rate of 25 mL/min, yielding ten subfractions (C3-2A–C3-2J). Further purification of subfraction C3-2C (817 mg) by preparative HPLC (CH_3CN/H_2O, 56/44, v/v; flow rate, 8 mL/min) yielded **15** (25.6 mg, t_R = 19.6 min), **2** (3.7 mg, t_R = 26.3 min), **19** (25.2 mg, t_R = 28.9 min), and subfraction C3-2C5 (130.5 mg). Subfraction C3-2C5 was further purified by semi-preparative HPLC (CH_3OH/H_2O, 75/25, v/v; flow rate, 3 mL/min) to yield **16** (5.2 mg, t_R = 22.5 min), **7** (5.9 mg, t_R = 28.2 min), and **10** (6.2 mg, t_R = 30.2 min). Similarly, subfraction C3-2F (186.5 mg) was purified by preparative HPLC (CH_3CN/H_2O, 63/37, v/v; flow rate, 8 mL/min) to yield **1** (2.4 mg, t_R = 23.8 min), **4** (4.3 mg, t_R = 26.1 min), and subfraction C3-2F2 (117.3 mg). Subfraction C3-2F2 was further purified by preparative HPLC

(CH_3OH/H_2O, 80/20, v/v; flow rate, 8 mL/min) to yield **5** (83.7 mg, t_R = 36.5 min), **14** (11.6 mg, t_R = 41.2 min), and **20** (3.2 mg, t_R= 43.3 min). Additionally, subfraction C3-2G (53 mg) was purified by preparative HPLC (CH_3CN/H_2O, 63/37, v/v; flow rate, 8 mL/min) to yield **11** (8.3 mg, t_R = 25.5 min) and **6** (36.5 mg, t_R = 42.6 min).

Fraction D (37 g) was chromatographed on an MCI gel column (6 × 80 cm) and eluted with CH_3OH/H_2O (30:70, 50:50, 70:30, and 90:10, v/v), affording four subfractions (D1–D4). Subfraction D3 (7.9 g) was further purified using Sephadex LH-20 eluted with CH_3OH/CH_2Cl_2 (1:1, v/v), yielding five subfractions (D3-1–D3-5). Subfraction D3-3 (1.4 g) was subjected to MPLC on an ODS column using a stepwise gradient of CH_3OH/H_2O (50:50 to 90:10, v/v) over 6 h at a flow rate of 25 mL/min, resulting in eight subfractions (D3-3A–D3-3H). Further purification of D3-3D (142 mg) by preparative HPLC (CH_3CN/H_2O, 55/45, v/v; flow rate, 8 mL/min) yielded **9** (12.4 mg, t_R = 26.5 min), **3** (5.8 mg, t_R = 33.7 min), and subfraction D3-3D2 (102.3 mg). Subfraction D3-3D2 was further purified by preparative HPLC (CH_3OH/H_2O, 70/30, v/v; flow rate, 8 mL/min) to yield **8** (13.5 mg, t_R = 38.4 min), **12** (5.4 mg, t_R = 42.2 min), and **13** (7.2 mg, t_R = 43.9 min). Similarly, subfraction D3-3E (312 mg) was purified by preparative HPLC (CH_3CN/H_2O, 60/40, v/v; flow rate, 8 mL/min) to yield **17** (3.1 mg, t_R = 28.4 min), **21** (2.8 mg, t_R = 33.1 min), and subfraction D3-3E2 (245.4 mg). Subfraction D3-3E2 was further purified by preparative HPLC (CH_3OH/H_2O, 78/22, v/v; flow rate, 8 mL/min) to yield **18** (7.5 mg, t_R = 38.7 min).

S7. ECD calculation details

Conformational analyses were carried out by random searching in Sybyl-X 2.0 using the MMFF94S force field with an energy cutoff of 5 kcal/mol (1). Geometry optimizations and frequency analyses were then performed at the B3LYP-D3(BJ)/6-31G* level in CPCM methanol using ORCA 5.0.1 (*2*). All conformers used for property calculations were confirmed as stable points on the potential energy surface (PES), with no imaginary frequencies. The excitation energies, oscillator strengths, and rotational strengths (velocity form) of the first 60 excited states were calculated using TD-DFT at the PBE0/def2-TZVP level in CPCM methanol using ORCA 5.0.1 (*2*). The ECD spectra were simulated using overlapping Gaussian functions (half bandwidth at 1/e peak height, σ = 0.30 for all conformers) (3). Gibbs free energies of the conformers were

obtained from thermal corrections at the B3LYP-D3(BJ)/6-31G* level and electronic energies evaluated at the ωB97M-V/def2-TZVP level in CPCM methanol using ORCA 5.0.1 (2). To obtain the final spectra, the simulated spectra of the conformers were averaged according to Boltzmann distributions based on their relative Gibbs free energies (ΔG). The absolute configurations of the stereocenters were assigned by comparing the experimental spectra with the calculated spectra of the model molecules.

S8. Thermal shift assay (TSA)

Purified protein was diluted to 0.5 mg/mL in buffer (20 mM Tris-HCl, pH 8.5, 250 mM NaCl) and incubated with compound **5** or DMSO for 30 min at room temperature. Each reaction (10 μL per well) contained 1 μL SYPRO™ Orange Protein Gel Stain (Invitrogen) and 9 μL of the protein-compound mixture. The melting curve was recorded using an Applied Biosystems® QuantStudio™ 6 Pro Real-Time PCR System (Thermo Fisher Scientific), with an initial incubation at 25 °C for 10 min, followed by a temperature gradient from 25 °C to 95 °C at 0.075 °C/s. Melting temperature (T_m) values were fitted and calculated using QuantStudio™ Design & Analysis Software v1.5.2.

S9. Cellular thermal shift assay (CETSA)

Purified TNF protein was incubated with compound **5** at 4 °C for 3 h. Samples were then heated at different temperatures for 10 min, mixed with 5× loading buffer, and 4 ng of total protein was loaded per lane. Proteins were separated by SDS-PAGE and transferred onto nitrocellulose membranes (Pall Corporation, Cat: 66485). Membranes were blocked with 5% (w/v) nonfat dry milk in TBST for 1 h and incubated overnight at 4 °C with an anti-TNF primary antibody (Elabscience, Cat: E-AB-33121). After three washes with TBST, membranes were incubated for 1 h with DyLight™ 800-conjugated secondary antibody (Cell Signaling Technology, Cat: 5151S). Protein bands were visualized using the Odyssey® CLx Imaging System (LI-COR).

S10. Molecular dynamics simulation

The protein-ligand complex obtained from molecular docking was used as the initial coordinate file. Molecular dynamics (MD) simulations were performed using GROMACS 2022.2 with the AMBER99SB-ILDN force field for proteins and the

GAFF force field for ligands (topology files generated via Acpype). The complex was solvated in a cubic periodic box with the TIP3P water model, and the net charge of the system was neutralized with sodium and chloride ions to a final ionic strength of 0.15 M. Energy minimization was conducted for 5000 steps using the steepest descent method to remove unfavorable contacts. Equilibration was performed under canonical (NVT) and isothermal-isobaric (NPT) ensembles while maintaining the temperature at 310 K and pressure at 1 bar. A 100 ns production MD simulation was then carried out. Post-simulation analyses were performed using built-in GROMACS modules, and the results were visualized and processed with Origin, PyMOL, and VMD.

S11. Isothermal titration calorimetry (ITC)

ITC experiments were performed to orthogonally quantify the interaction of TNF with HPJ or SPD304 in solution. Purified TNF and compounds were prepared in matched buffer to minimize the heat of dilution. Compounds were titrated into TNF at a constant temperature, and the heat released or absorbed after each injection was recorded. Binding parameters, including K_D, stoichiometry (n), enthalpy change (ΔH), and fitting R^2, were obtained by fitting the integrated heat data to a one-site binding model.

S12. Protein production and purification

The gene encoding human ULP1 (UniProt: P02724) was synthesized by GENEWIZ (Azenta Life Sciences) and cloned into the pET-28a vector (NdeI/XhoI) using standard pET-28a cloning and expression procedures. The recombinant plasmid was transformed into *E. coli* BL21 (DE3) cells and cultured in Luria Broth (LB)containing appropriate antibiotics at 37 °C until OD_{600} reached 0.6–0.8. Expression was induced with 0.4 mM IPTG, and cultures were incubated for an additional 16–20 h at 18 °C. Cells were harvested by centrifugation and resuspended in lysis buffer (50 mM Tris-HCl pH 8.0, 300 mM NaCl, 5 mM *β*-mercaptoethanol, 10% glycerol, 1% Triton X-100, 10 mM imidazole). Soluble C-terminal hexahistidine-tagged ULP1 was bound to Ni-agarose affinity resin (Qiagen, Cat: 172018714), washed with buffer containing 20 mM Tris-HCl pH 8.0, 300 mM NaCl, 20 mM imidazole, and eluted with buffer containing 20 mM Tris-HCl pH 8.0, 300 mM NaCl, 300 mM imidazole. Purified ULP1 was flash-frozen and stored at −80 °C for subsequent

experiments.

The gene encoding human TNF (UniProt: P01375) was synthesized by GENEWIZ (Azenta Life Sciences) and cloned into the pET-28a-SUMO vector (BamHI/XhoI) as previously reported. The plasmid was transformed into *E. coli* BL21 (DE3) cells, which were cultured in LB broth containing appropriate antibiotics at 37 °C until OD_{600} reached 0.6–0.8. Expression was induced with 0.4 mM IPTG, followed by incubation for 16–20 h at 18 °C. Cells were collected by centrifugation and resuspended in lysis buffer (20 mM Tris-HCl, pH 8.5, 200 mM NaCl, 5 mM *β*-mercaptoethanol, 5% glycerol, 0.1% Triton X-100), lysed by sonication 3–5 times, and clarified by centrifugation at 10,000 g, 4 °C for 1 h. The supernatant was loaded onto Ni-agarose resin pre-equilibrated with buffer A (20 mM Tris-HCl pH 8.5, 250 mM NaCl, 10 mM imidazole) and eluted with buffer B (20 mM Tris-HCl pH 8.5, 250 mM NaCl, 150 mM imidazole). For tag removal, TNF and ULP1 were mixed at a 10:1 mass ratio and incubated overnight at 4 °C on a rotary shaker. The mixture was applied to a gravity column, and the flow-through fraction was collected. The reconstituted supernatant was filtered and further purified by size-exclusion chromatography (HiLoad™ 16/60 Superdex 75, Cytiva). The purified TNF protein was flash-frozen and stored at –80 °C for subsequent experiments.

S13. L929 cell viability analysis

L929 cells were cultured in high-glucose DMEM supplemented with 10% heat-inactivated FBS and 100 U/mL penicillin/streptomycin at 37 °C in a humidified atmosphere containing 5% CO_2. Cells were seeded in 96-well plates at a density of 1.5 × 10^5 cells/mL in 50 µL per well and allowed to adhere for 24 h. Subsequently, cells were treated with 40 µM of compounds 1–7 for 24 h. Cell viability was determined using the CCK-8 assay, and absorbance was measured at 450 nm. Cell viability ratio (%) = $OD450_{treated}/OD450_{blank} \times 100\%$.

S14. TNF-induced L929 cells cytotoxicity protection assay

L929 cells in the logarithmic growth phase were seeded in 96-well plates at 1.5 × 10^5 cells/mL in 50 µL per well and preincubated for 24 h. Cells were then treated with varying concentrations of compounds **1**–**7** for 24 h, followed by stimulation with TNF

(2 ng/mL) and actinomycin D (1 μg/mL) for an additional 24 h. Cell viability was assessed using the CCK-8 assay as described above and calculated using the same formula.

S15. RNA extraction

HaCaT cells were cultured in MEM supplemented with 15% heat-inactivated FBS and 100 U/mL penicillin/streptomycin at 37 °C in a humidified 5% CO_2 atmosphere. Cells were seeded at 2 × 10^5 cells/mL in 100 mm culture dishes (8 mL per dish). After 24 h of preincubation, cells were treated with 5 μM compound **5** for 24 h, followed by TNF stimulation (10 ng/mL) for an additional 24 h. Total RNA was extracted using Total RNA Isolation Reagent (Biosharp, Cat: BS258A), and genomic DNA was removed with DNase I (TaKaRa). RNA quality was assessed using an Agilent 2100 Bioanalyzer and quantified using a NanoDrop ND-2000. Only high-quality RNA samples ($OD_{260/280}$ = 1.8–2.2, $OD_{260/230}$ ≥ 2.0, RIN ≥ 6.5, 28S:18S ≥ 1.0, >10 μg) were used for library construction.

S16. Disruption of TNF trimerization assembly

Compound **5** was incubated with 100 ng of TNF at 37 °C for 1 h, followed by cross-linking with 5 mM BS^3 (Yuanye, Cat: T25103) for 30 min at room temperature. The reaction was quenched by adding Tris-HCl to a final concentration of 1 M (Biosharp, CAS: 1185-53-1). Samples were separated by SDS-PAGE and transferred onto nitrocellulose membranes. Membranes were blocked with 5% (w/v) nonfat dry milk in TBST for 1 h and incubated overnight at 4 °C with anti-TNF primary antibodies. After three washes with TBST, membranes were incubated for 1 h with DyLight™ 800-conjugated secondary antibody and visualized using the Odyssey® CLx Imaging System (LI-COR).

S17. Drug affinity-responsive target stability assay

Purified TNF protein was incubated with compound **5** at 4 °C for 3 h, followed by incubation with streptavidin (1:1000) for 30 min at room temperature. The samples were separated by SDS-PAGE and then electrophoretically transferred onto nitrocellulose membranes. Membranes were blocked with (5% (w/v) nonfat dry milk in TBST) for 1 h and then incubated overnight at 4 °C with specific anti-TNF primary

antibodies. After three washes with TBST, membranes were incubated for 1 h with DyLight™ 800-conjugated secondary antibody and visualized using by the Odyssey® CLx Imaging System (LICOR).

S18. TNF-induced L929 cell death inhibition assay

L929 cells were seeded in 6-well plates at 1 × 10^4 cells/mL in 2 mL per well and preincubated for 24 h at 37 °C in 5% CO_2. Cells were then treated with 5 or 10 µM of compound **5** for 24 h, followed by stimulation with TNF (2 ng/mL) and actinomycin D (1 µg/mL) for an additional 24 h. Cells were washed with PBS, fixed with 4% paraformaldehyde for 30 min, and stained with DAPI at room temperature for 5 min. Images were acquired using an OLYMPUS IX73 fluorescence microscope (excitation/emission: 360 nm/450 nm). SPD304 was used as the positive control.

S19. Molecular docking

Molecular docking was performed using Schrödinger Maestro (Schrödinger Release 2023-1). Compound **5** was preprocessed using the LigPrep module, and the TNF dimer structure (PDB ID: 2AZ5) was prepared using the Protein Preparation Wizard. Ligand docking was carried out with default parameters, and protein-ligand interactions were visualized using PyMOL.

S20. Library preparation and NGS sequencing

RNA-seq libraries were prepared using the TruSeq™ RNA Sample Preparation Kit (Illumina, San Diego, CA) with 1 µg of total RNA. Messenger RNA was isolated by poly(A) selection using oligo(dT) beads and fragmented. cDNA synthesis, end repair, A-tailing, and ligation of indexed adapters were performed. Libraries were size-selected for 200–300 bp cDNA fragments using 2% Low Range Ultra Agarose and PCR-amplified for 15 cycles with Phusion DNA polymerase (NEB). Libraries were quantified using a TBS380 fluorometer and sequenced as paired-end reads (150 bp × 2) at Shanghai BIOZERON Co., Ltd.

S21. Differential expression analysis and functional enrichment

Gene expression levels were estimated using the fragments per kilobase of exon

per million mapped reads (FPKM) method, and differentially expressed genes (DEGs) between samples were identified using the R package edgeR. DEGs were selected based on a $\log_2$ fold change > 2 and a false discovery rate (FDR) < 0.05. To explore the biological functions of DEGs, Gene Ontology (GO) enrichment and Kyoto Encyclopedia of Genes and Genomes (KEGG) pathway analyses were conducted using Goatools and KOBAS, respectively. GO terms and pathways with a Bonferroni-corrected P-value < 0.05 were considered significantly enriched.

S22. RNA isolation and RT-qPCR

Total RNA from HaCaT cells treated with compound **5** and stimulated with TNF (10 ng/mL) was extracted using Total RNA Isolation Reagent following the manufacturer's instructions. cDNA was synthesized using Hifair® III 1st Strand cDNA Synthesis SuperMix for qPCR (YEASEN, Cat: 11141ES60). RT-qPCR was performed using Hieff® qPCR SYBR Green Master Mix (YEASEN, Cat: 11202ES60) with 0.2 μM of each primer in a 10 μL reaction volume on an ABI QuantStudio 5 system. The thermal cycling protocol included an initial denaturation at 95 °C for 3 min, followed by 40 cycles of 95 °C for 10 s, 60 °C for 30 s, and 72 °C for 30 s. Relative expression levels were normalized to β-actin. Primer sequences are listed in Table S5.

S23. Pharmacokinetic study

Male Sprague–Dawley (SD) mice (n = 3 per time point per route) received a single dose of HPJ *via* intravenous (*i.v.*, 5 mg/kg), intraperitoneal (*i.p.*, 10 mg/kg), or intragastric (*i.g.*, 20 mg/kg) administration. Blood samples were collected at 0.083, 0.25, 0.5, 1, 2, 4, 8, 12, and 24 h post-dose. Plasma concentrations of HPJ were determined by LC-MS/MS. Pharmacokinetic parameters were calculated using non-compartmental analysis (Phoenix WinNonlin).

S24. Western blotting assay

Total protein was extracted from HaCaT cells treated with compound **5** and stimulated with TNF (10 ng/mL) using RIPA buffer (Beyotime, Cat: P0013B). Protein concentrations were measured with a BCA assay kit (Beyotime, Cat: P0012S). Samples were mixed with loading buffer, denatured at 95 °C for 10 min, separated by SDS-PAGE, and transferred onto nitrocellulose membranes. Membranes were blocked with 5% (w/v) nonfat dry milk in TBST for 1 h, incubated overnight at 4 °C with specific

primary antibodies, washed three times with TBST, and then incubated for 1 h with DyLight™ 800-conjugated secondary antibodies. Protein bands were visualized using the Odyssey® CLx Imaging System (LI-COR).

S25. Immunofluorescence

After treatment with compound **5** as described above, cells cultured on glass coverslips were fixed with 4% cold paraformaldehyde for 15 min and permeabilized with 0.1% Triton X-100 for 30 min. Coverslips were washed three times with PBS and blocked with 3% BSA for 1 h. Cells were incubated overnight at 4 °C with a primary antibody against the NF-*κ*B p65 subunit, followed by incubation with Alexa Fluor-594-conjugated secondary antibody for 1 h at room temperature in the dark. Nuclei were stained with DAPI for 30 min at 37 °C, and coverslips were washed and mounted. Images were captured using an OLYMPUS IX73 fluorescence microscope (excitation/emission: 590/617 nm for Alexa Fluor-594; 360/450 nm for DAPI). For TNFR signaling validation, cells were fixed, permeabilized, blocked, and incubated with primary antibodies against TNF, TNFR1, or NF-*κ*B p65, followed by fluorophore-conjugated secondary antibodies. TNF was visualized in red, TNFR1 or p65 in green, and nuclei were counterstained with DAPI. Images were acquired using an OLYMPUS IX73 fluorescence microscope under identical exposure settings for each channel.

S26. Psoriasis model and drug administration

Seven- to eight-week-old BALB/c mice were randomly assigned to five groups (n = 5 per group): blank control (vaseline), IMQ model, vehicle control (60% anhydrous ethanol/40% glycerol under IMQ), positive control (Tapinarof 1%), and compound **5** (1%). Compound **5** and Tapinarof were prepared as 1% (w/v) solutions in 60% anhydrous ethanol and 40% glycerol and applied topically to a hair-free dorsal skin area of approximately 2 cm × 3 cm once daily for 7 consecutive days. Each application used the same volume per unit area across treatment groups. Except for the blank control, IMQ (62.5 mg per mouse) was applied to the same dorsal area to induce psoriasis. PASI scoring and histological evaluation were performed by investigators blinded to group allocation. No animals were excluded from the final analysis. No formal a priori power calculation was performed; the sample size was selected based on previous preliminary pharmacodynamic studies and ethical considerations to minimize animal use. For histological and immunostaining quantification, n refers to the number of animals per

group, and multiple representative fields were analyzed for each animal where applicable.

S27. Psoriasis Area and Severity Index (PASI)

Dorsal skin changes were observed daily, and erythema, scaling, and thickness were scored on a scale of 0–4 (0 = none, 1 = mild, 2 = moderate, 3 = severe, 4 = very severe) to calculate PASI for 7 consecutive days.

S28. ALI model and drug administration

Seven- to eight-week-old BALB/c mice were randomly divided into four groups (n = 5 per group): blank control (normal), positive control (DEX 10 mg/kg), compound **5** (10 mg/kg), and LPS model. Compound **5** and DEX were administered intraperitoneally once daily for 3 days. LPS (12.5 mg/kg) was then administered intraperitoneally to induce ALI. This regimen was designed as a prophylactic intervention to evaluate whether HPJ could prevent the initiation and amplification of the LPS-induced inflammatory response. After 24 h, bronchoalveolar lavage fluid (BALF) was collected by rinsing the lungs with PBS for flow cytometry and cytokine analysis. Animals were randomly allocated to treatment groups, and histological evaluation was performed by investigators blinded to group allocation. No animals were excluded from the final analysis. No formal a priori power calculation was performed; the sample size was selected based on previous preliminary pharmacodynamic studies and ethical considerations to minimize animal use. For flow cytometry, cytokine analysis, and histological quantification, n refers to the number of animals per group unless otherwise specified.

S29. Flow cytometry assay

Cells were collected from bronchoalveolar lavage fluid by centrifugation. After blocking with anti-CD16/CD32 antibody (Elabscience, Cat: E-AB-F0997A), cells were incubated with appropriate dilutions of PE-F4/80 (Elabscience, Cat: E-AB-F0995D), PE-Ly6G (Elabscience, Cat: E-AB-F1108D), APC-CD11b (Elabscience, Cat: E-AB-F1081E), and FITC-CD45 (Elabscience, Cat: E-AB-F1136C) to determine the proportions of neutrophils and macrophages. Data were acquired using a Sony ID7000 Flow Analyzer (5-laser configuration) and analyzed with FlowJo.

S30. ELISA assay

Serum cytokine levels were measured using commercial ELISA kits: Mouse TNF (Elabscience, Cat: E-EL-M3063), IL-1β (Elabscience, Cat: E-EL-M0037), IL-6 (Elabscience, Cat: E-EL-M0044), IL-10 (Elabscience, Cat: E-EL-M0046), and LCN-2 (Meimian, Cat: MM-44688M1).

S31. Hematoxylin-eosin staining

Mouse skin samples were fixed in 4% paraformaldehyde for 48 h, dehydrated through graded ethanol, embedded in paraffin, and sectioned at 5 μm. Sections were deparaffinized, rehydrated, stained with hematoxylin and eosin, dehydrated, cleared with dimethylbenzene, and examined microscopically.

S32. Immunohistochemistry

Paraffin sections were treated with 3% hydrogen peroxide to block endogenous peroxidase activity. Sections were sequentially incubated with primary and secondary antibodies, stained with DAB, counterstained, dehydrated, and mounted for microscopic observation.

S33. Surface plasmon resonance

Purified TNF protein was immobilized on a CM5 chip (Cytiva, Cat: 29104988) by amine coupling using the Biacore 1K Molecular Interaction Analysis System. Binding enrichment and kinetic analyses of compound 5 were performed using the LMW multi-cycle kinetics/affinity program in Biacore 1K Control Software to determine the optimal coupling concentration and pH.

S34. LT-α-induced WEHI164 cell death inhibition assay

WEHI164 cells were seeded in 6-well plates at 1×10^4 cells/mL in 2 mL per well and preincubated for 24 h at 37 °C in 5% CO_2. Cells were then treated with 5 or 10 μM of compound **5** for 24 h, followed by stimulation with LT-α (2 ng/mL) and actinomycin D (1 μg/mL) for an additional 24 h. Cells were washed with PBS, fixed with 4% paraformaldehyde for 30 min, and stained with DAPI at room temperature for 5 min. Images were acquired using an OLYMPUS IX73 fluorescence microscope (excitation/emission: 360 nm/450 nm).

Supplementary Figures

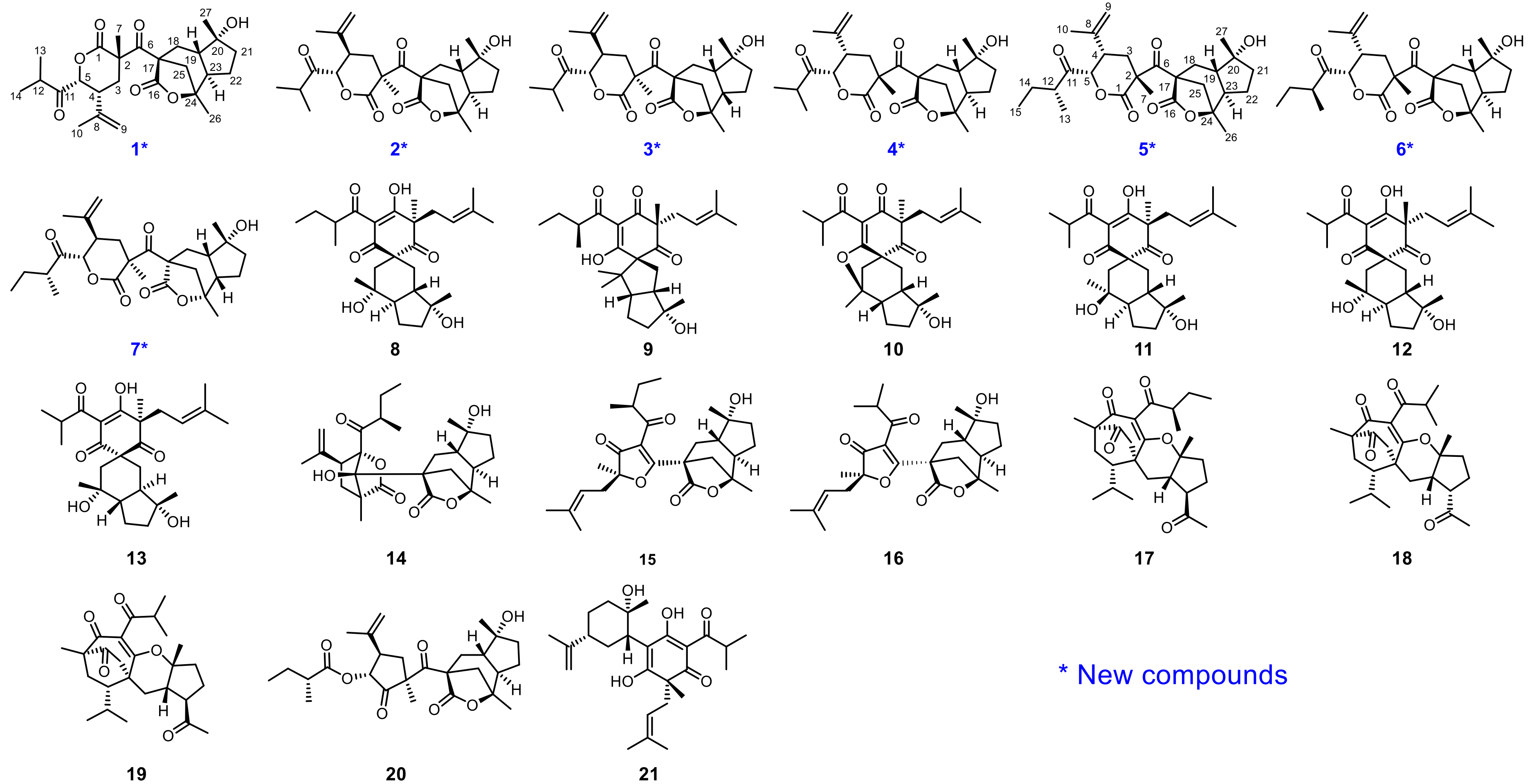

Figure S1. Chemical structures of compounds **1–21**.

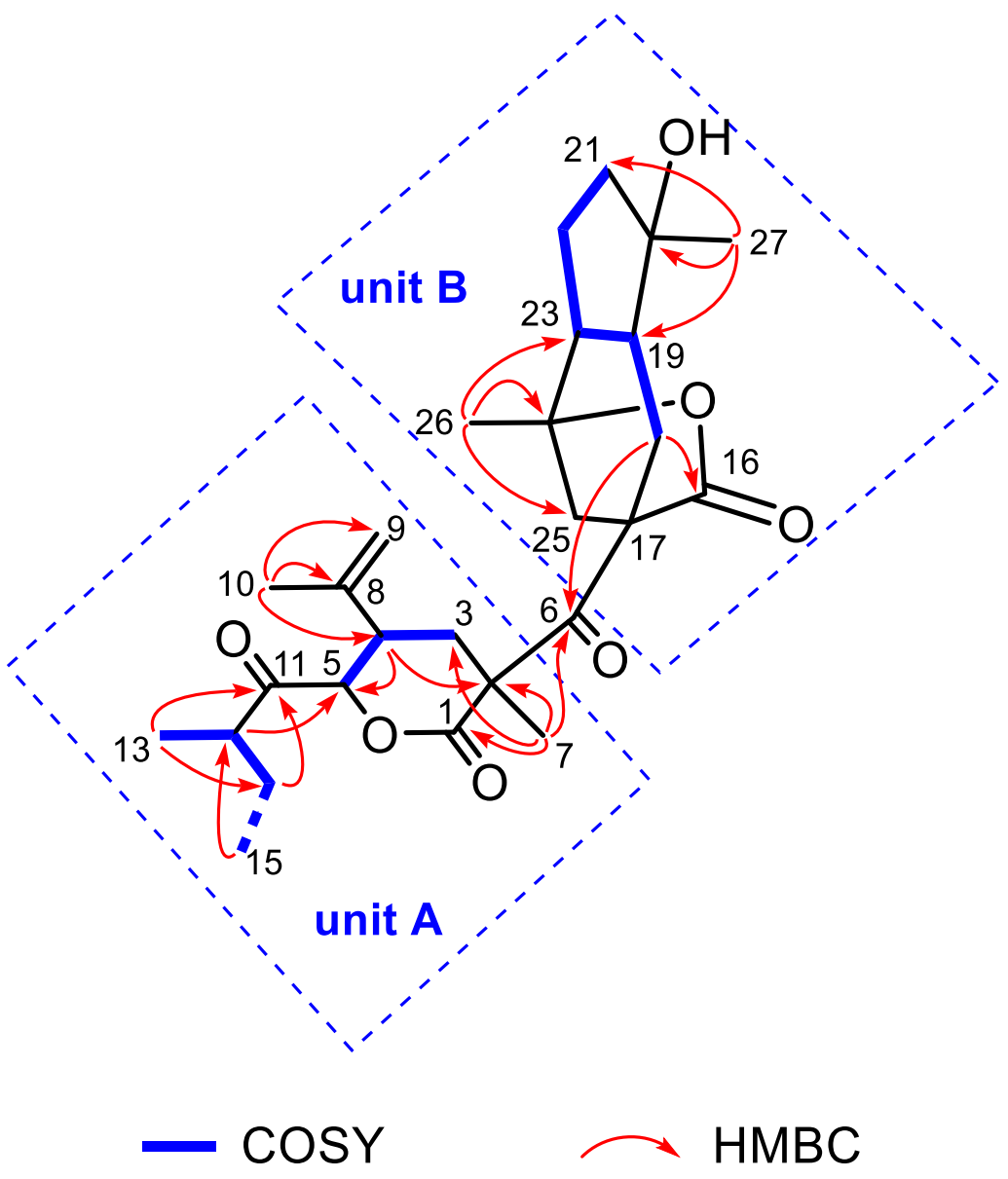

Figure S2. Key ^1H–^1H COSY and HMBC correlations of **1**–**7**.

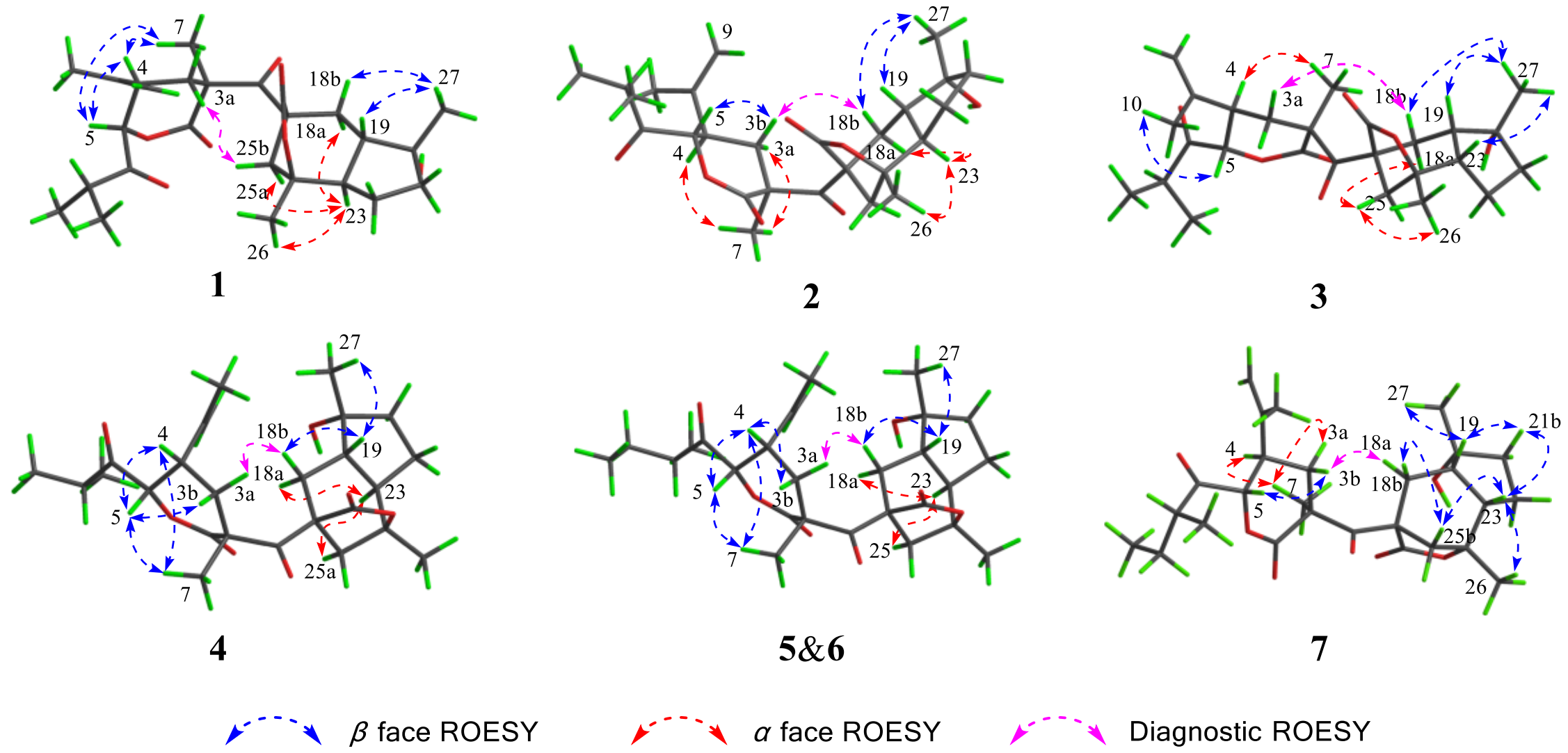

Figure S3. Key ROESY correlations of **1**–**7**.

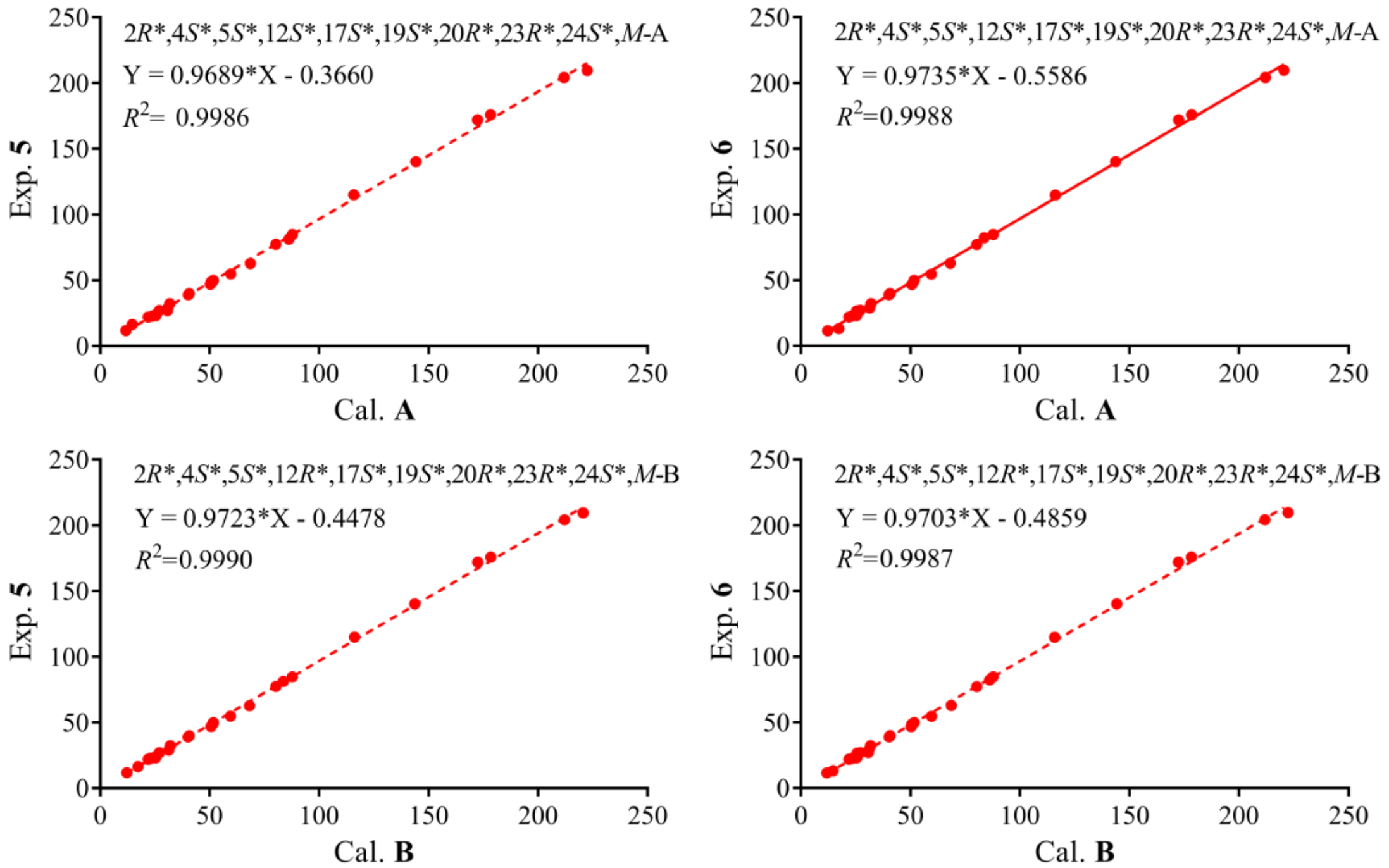

Figure S4. R^2 value analysis of ^{13}C NMR chemical shifts of **5** and **6** based on calculated and experimental values.

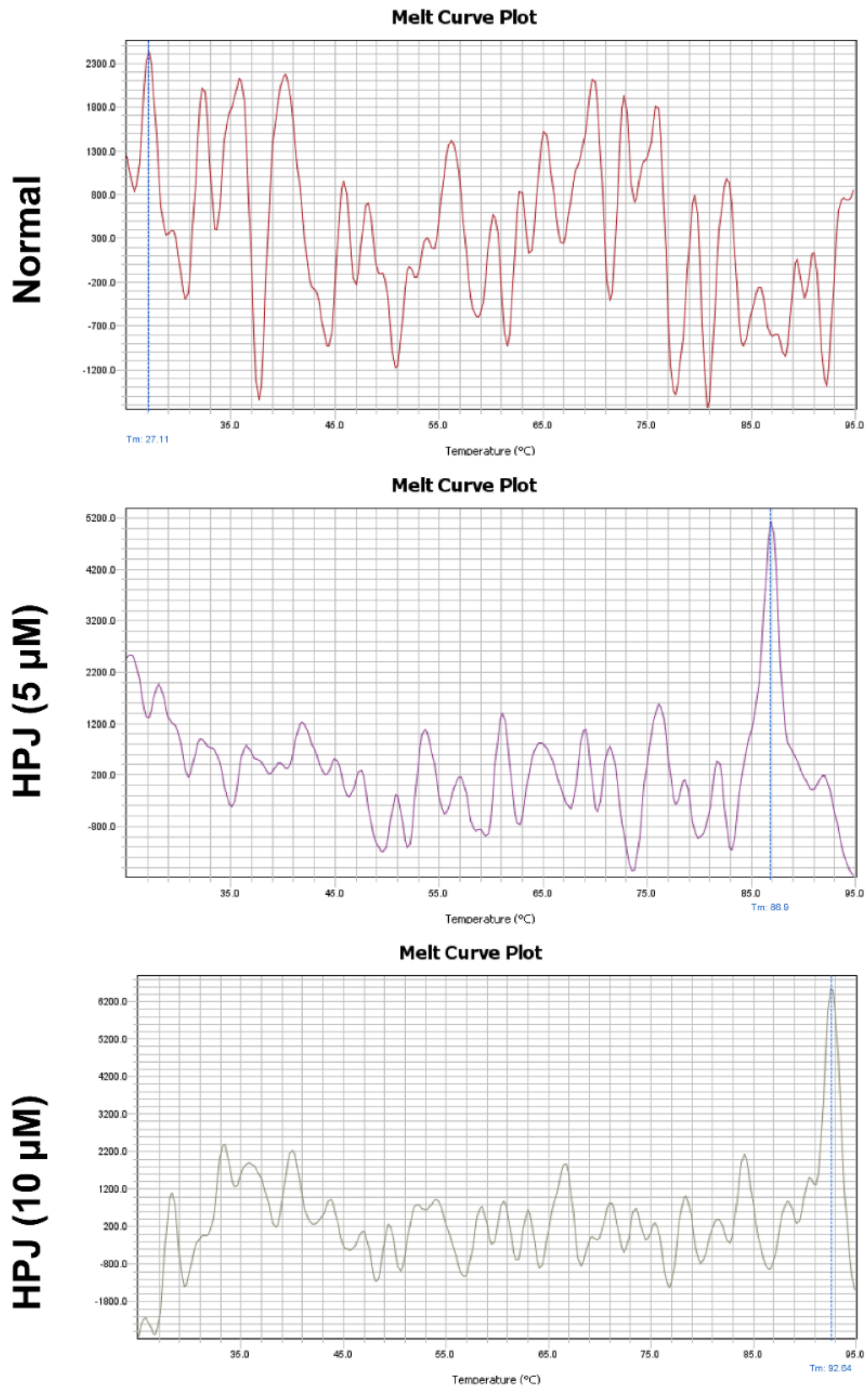

Figure S5. TSA assay to detect the thermal stability of purified TNF protein.

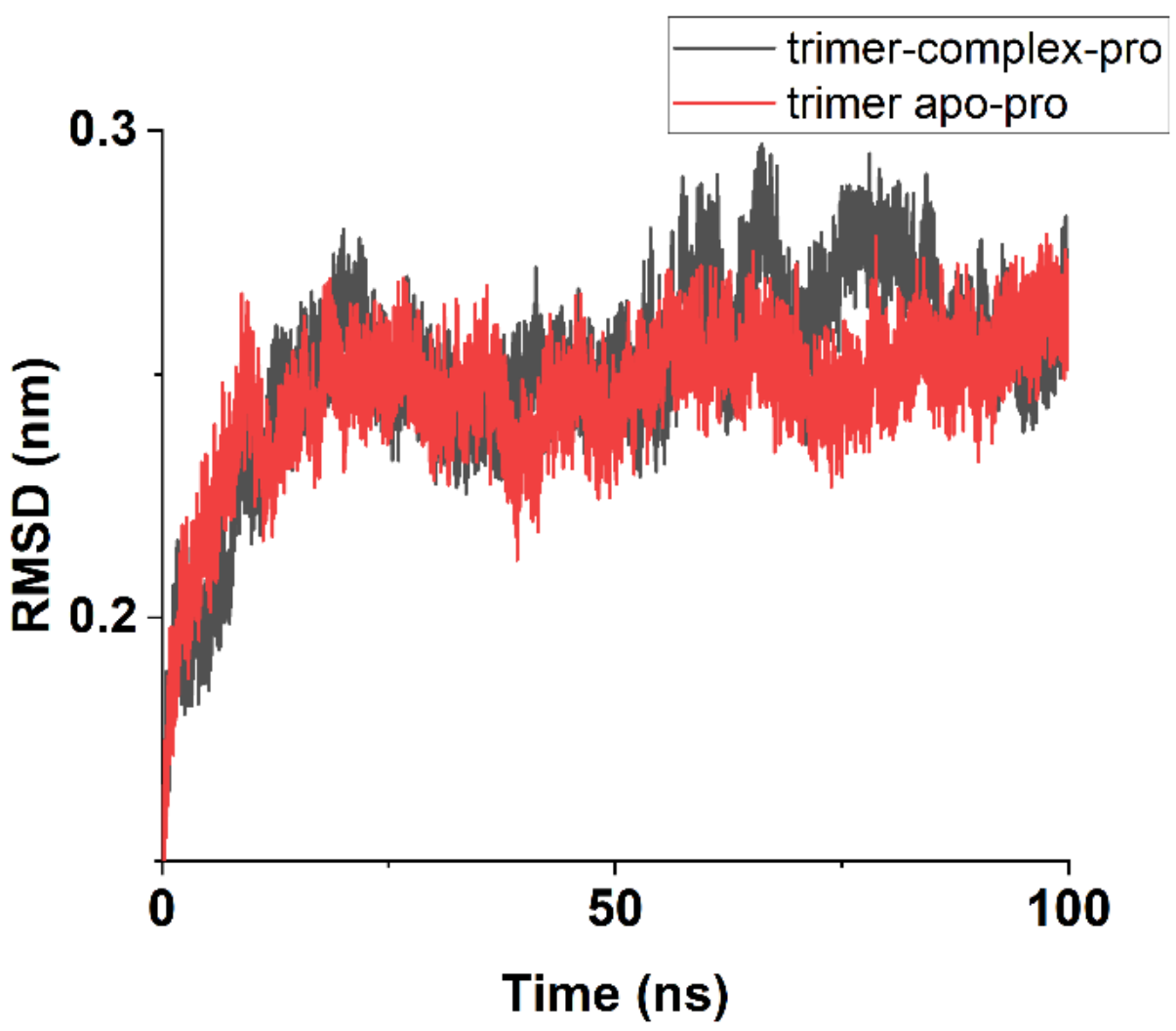

Figure S6. RMSD comparison of TNF trimer in the apo and complex.

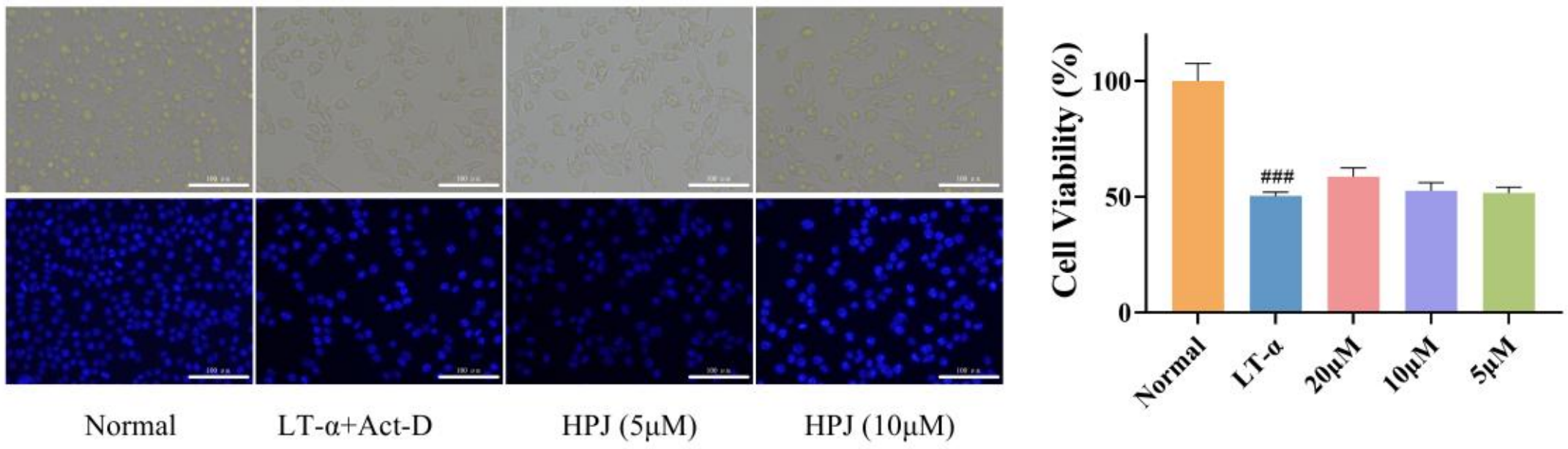

Figure S7. Cell viability of compound **5** against LT-*α*-induced cytotoxicity in WEHI164 cells.

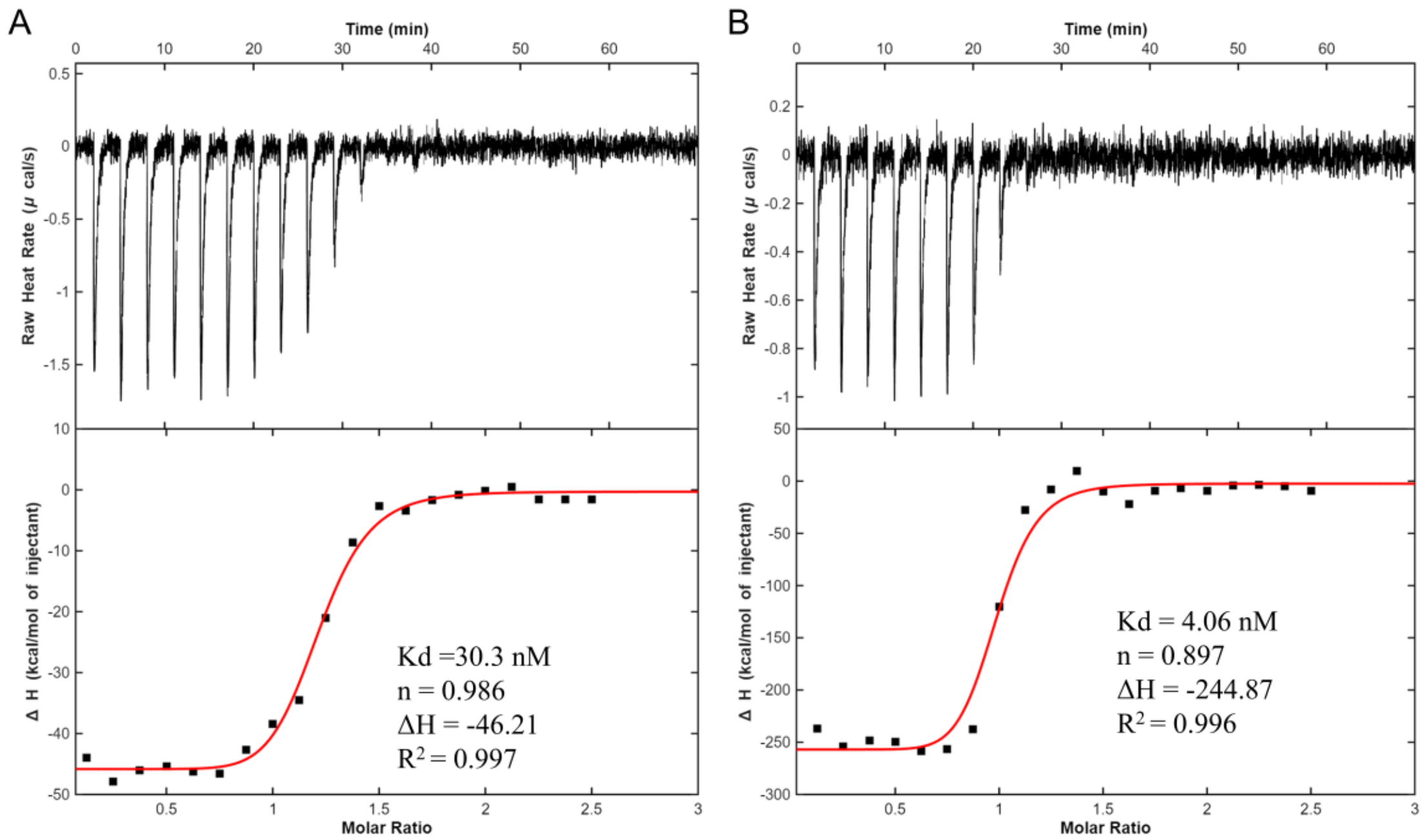

Figure S8. The results of isothermal titration calorimetry (ITC) assays for the binding of HPJ and SPD304 to TNF. (A) SPD304 binding to TNF: The upper panel displays the raw heat flow curve, and the lower panel shows the thermodynamic fitting curve. (B) HPJ binding to TNF: The upper panel presents the raw heat flow curve, and the lower panel shows the thermodynamic fitting curve.

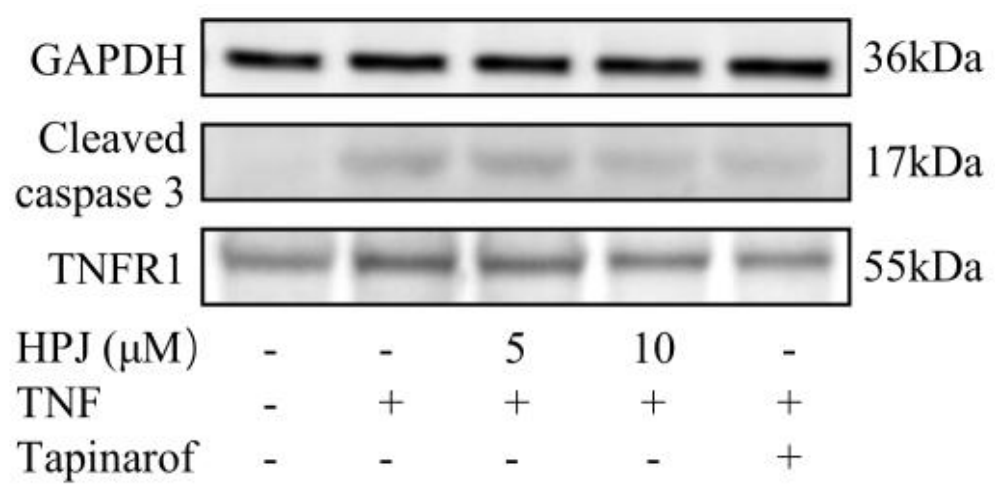

Figure S9. Western blot analysis of cleaved caspase-3 and TNFR1.

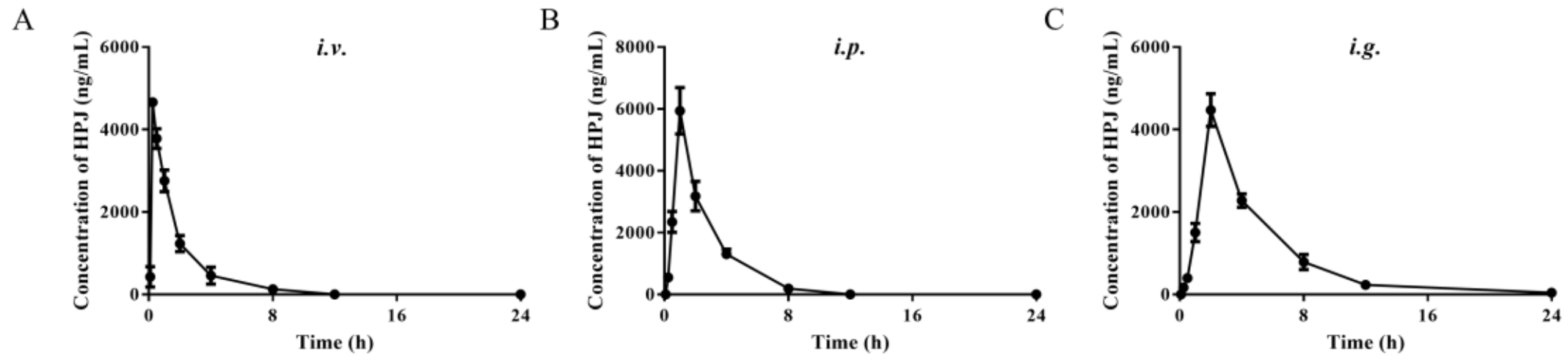

Figure S10. *In vivo* pharmacokinetic properties of HPJ.

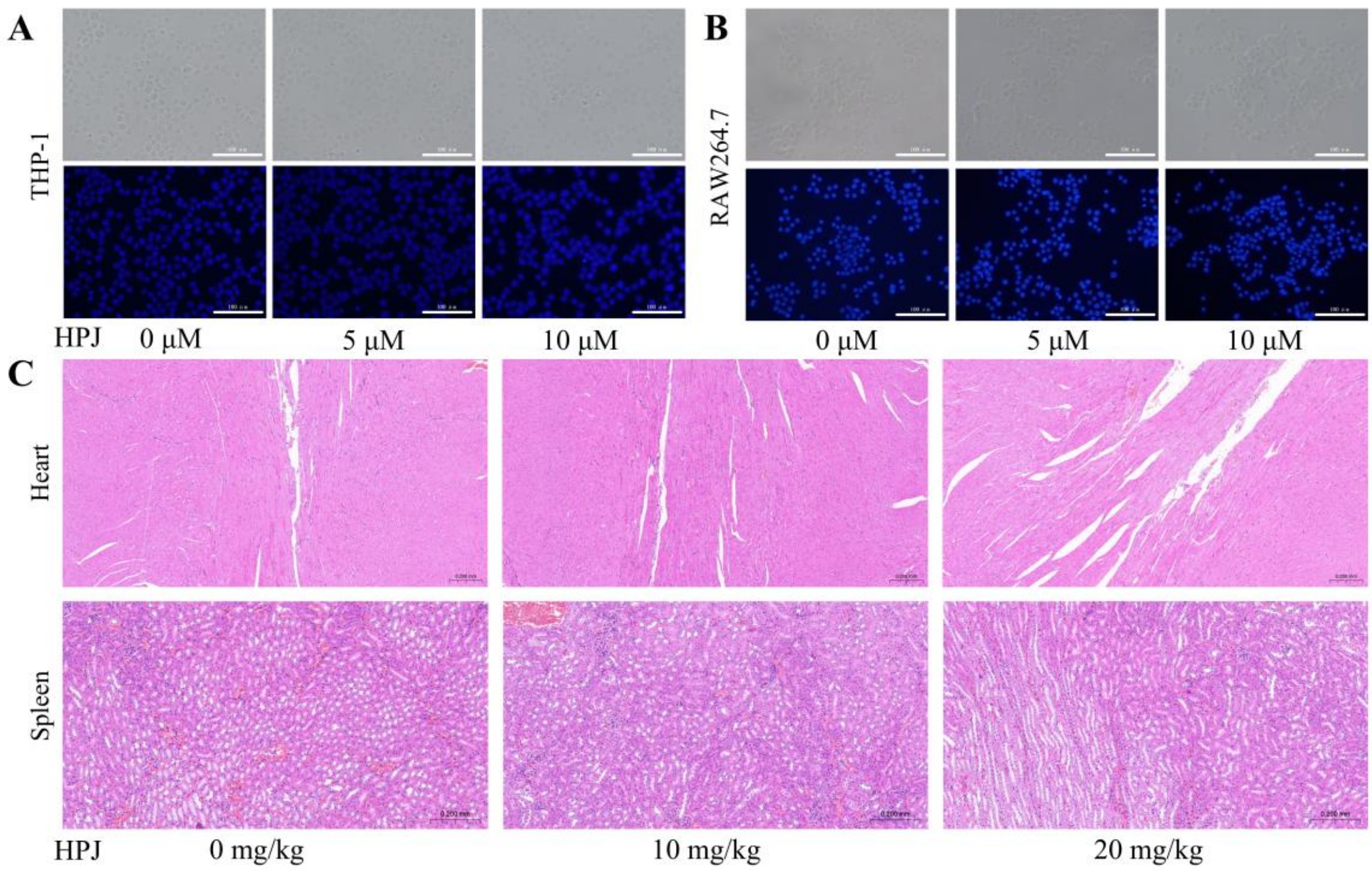

Figure S11. (A) Cytotoxicity evaluation of HPJ in THP-1 cells. (B) Cytotoxicity evaluation of HPJ in RAW264.7 cells. (C) *In vivo* safety assessment of HPJ by H&E staining.

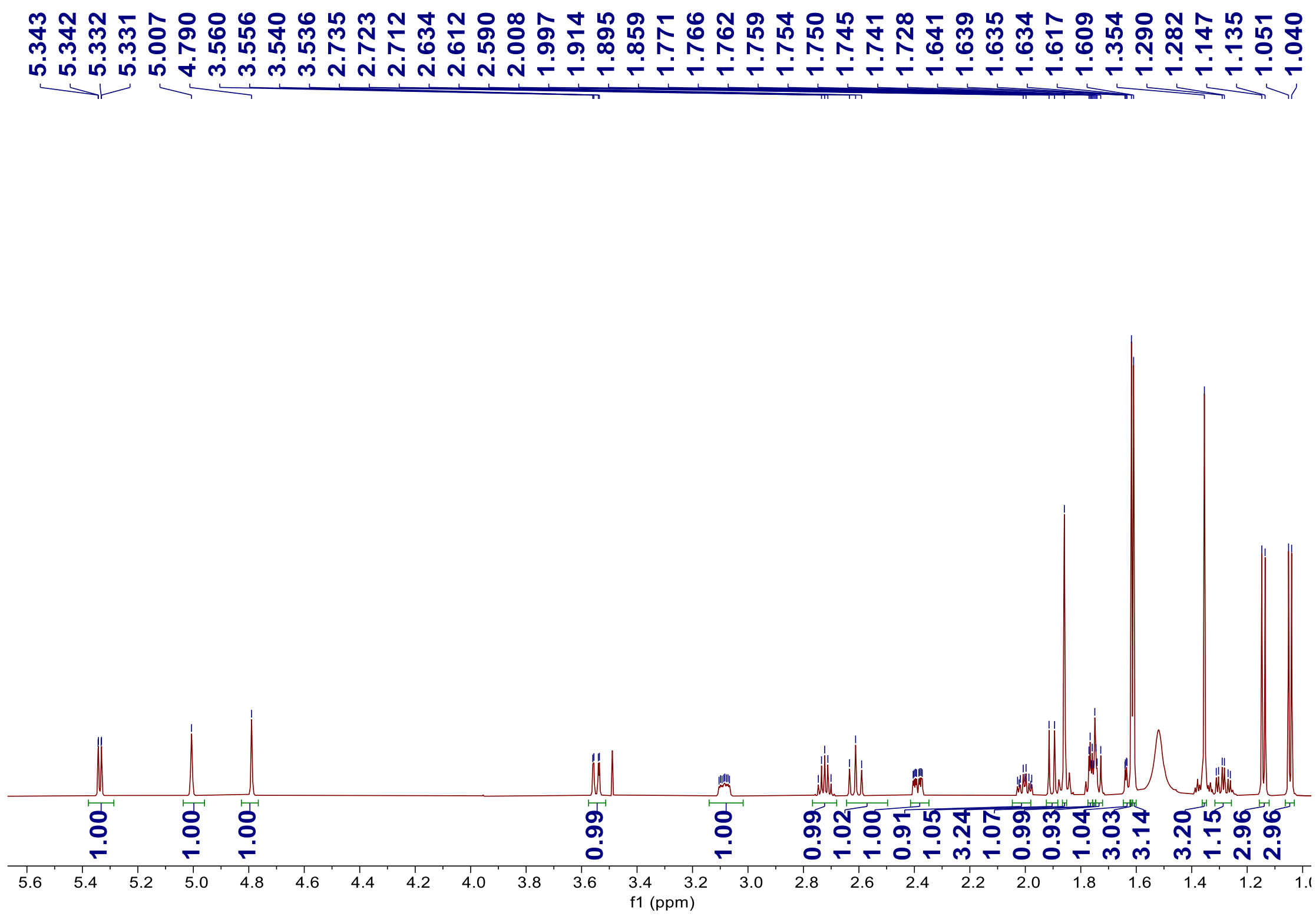

Figure S12. ^{1}H NMR spectrum of compound **1** (recorded in $CDCl_3$)

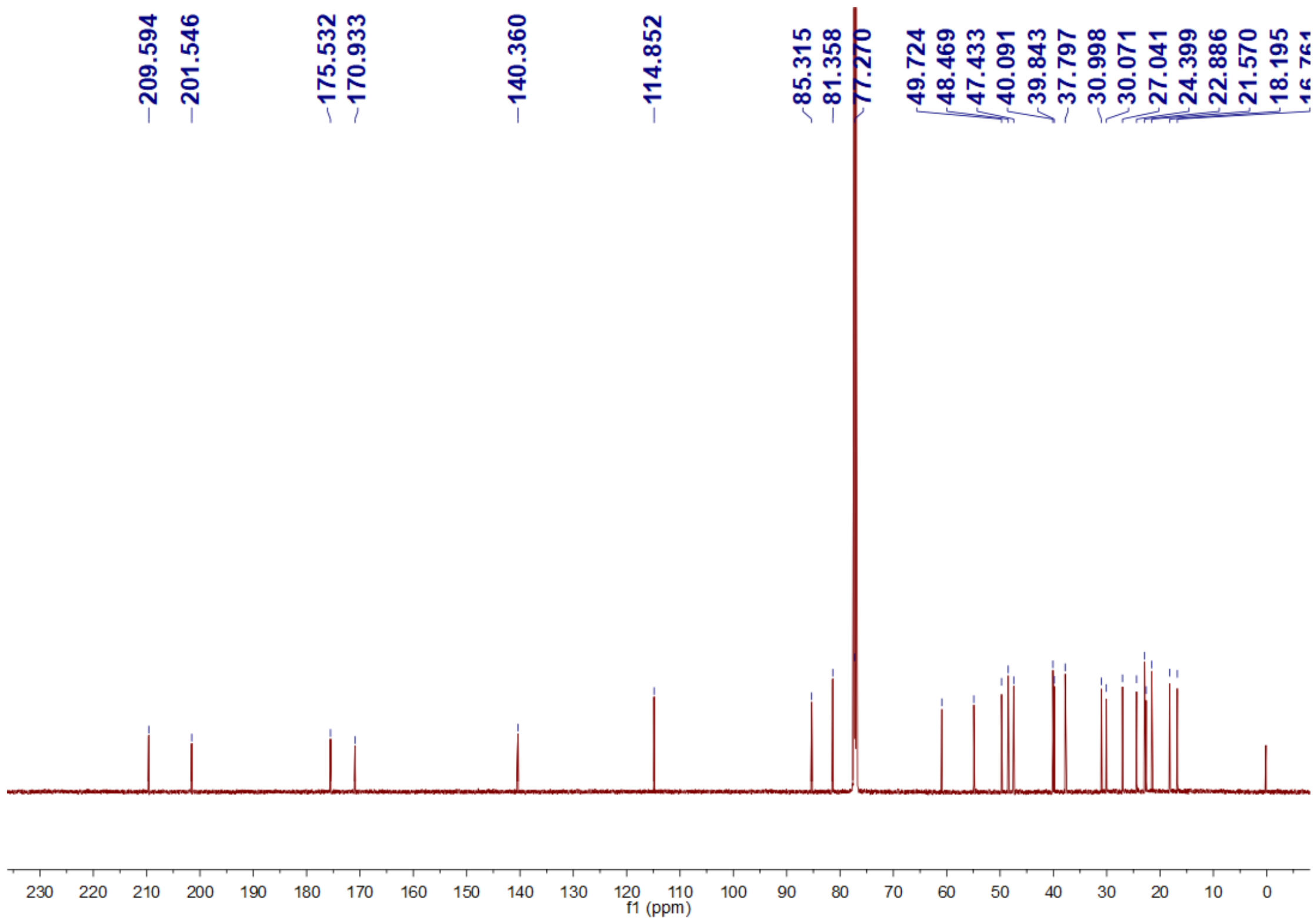

Figure S13. ^{13}C NMR spectrum of compound **1** (recorded in $CDCl_3$).

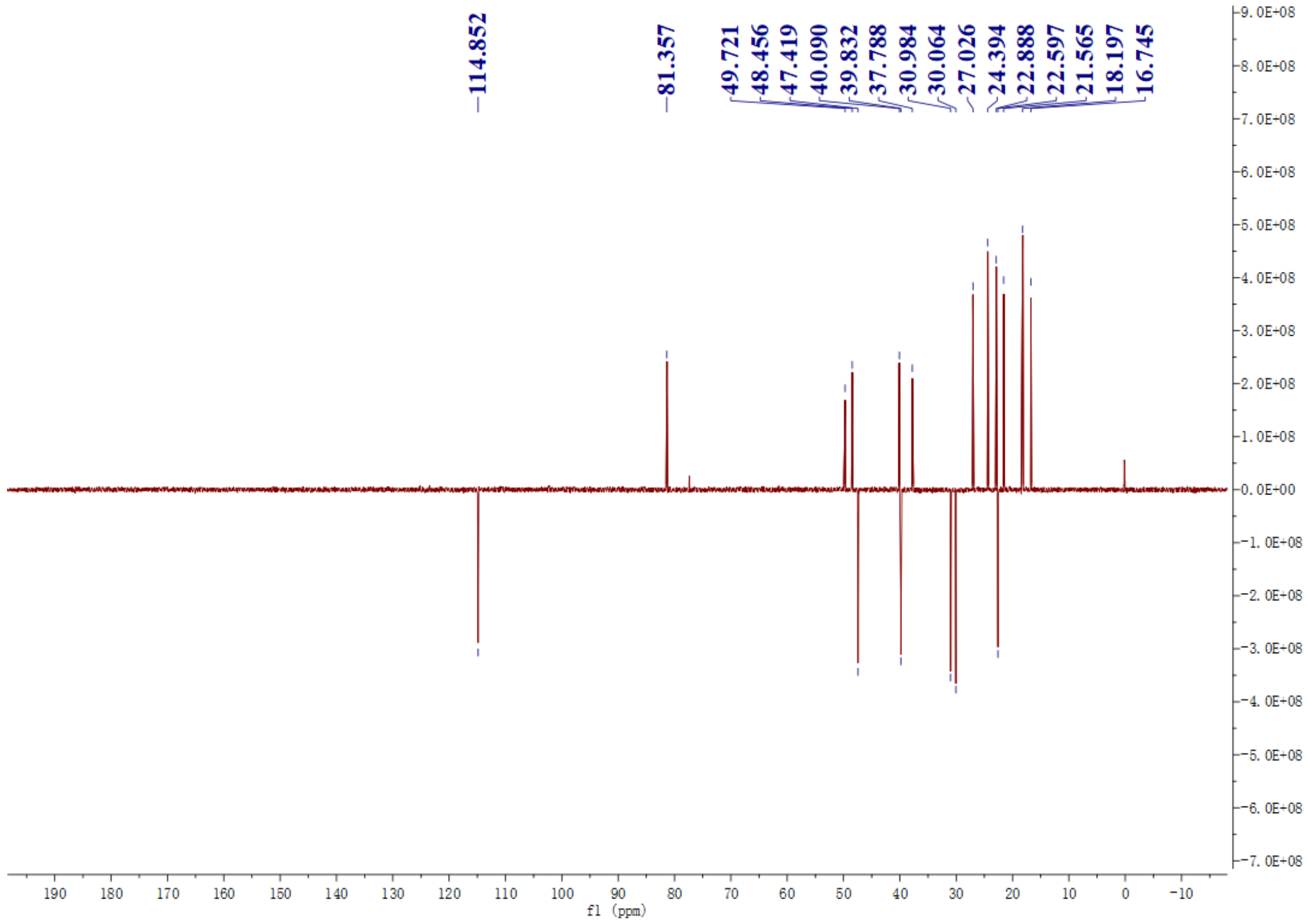

Figure S14. DEPT spectrum of compound **1** (recorded in $CDCl_3$).

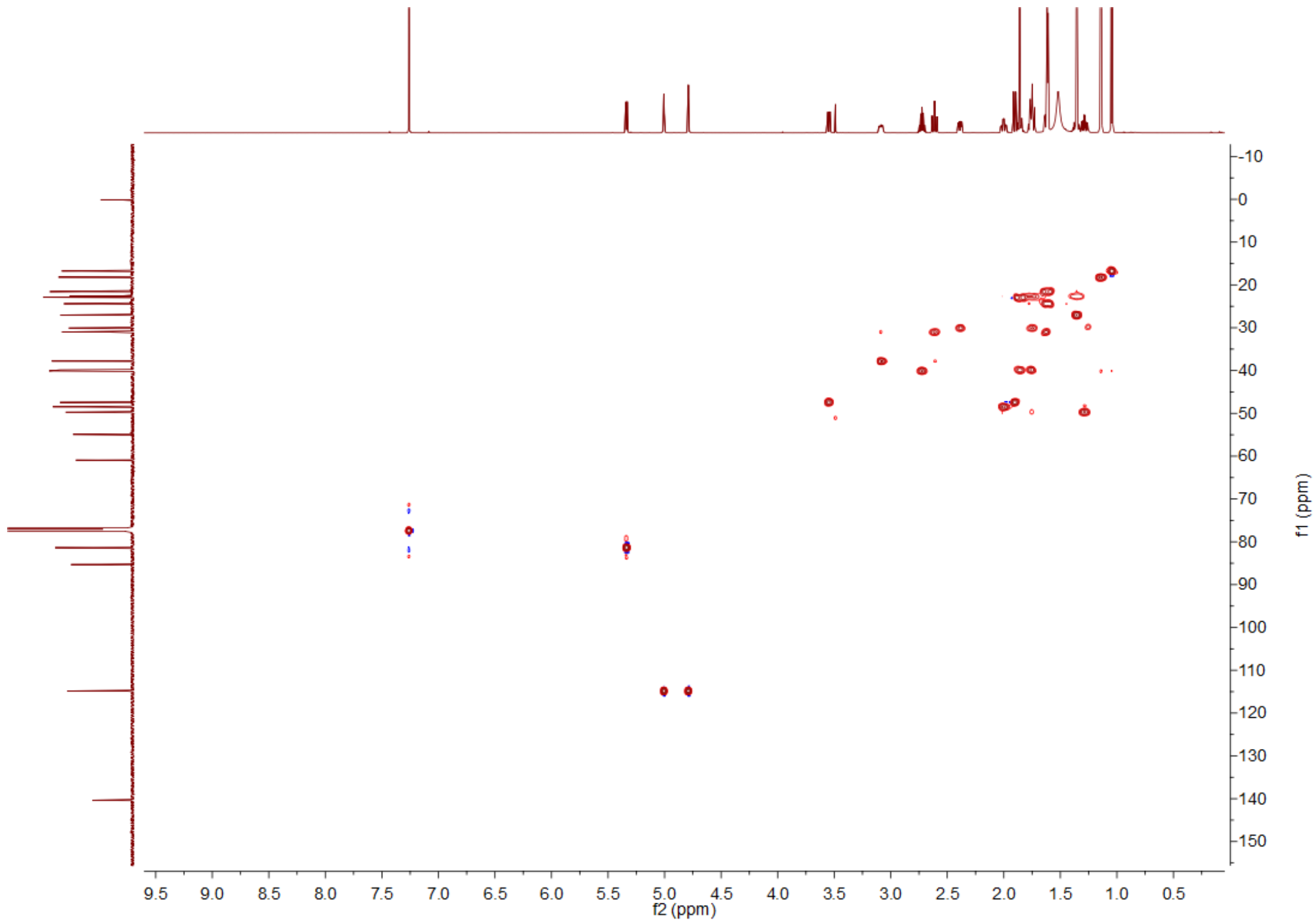

Figure S15. HSQC spectrum of compound **1** (recorded in $CDCl_3$).

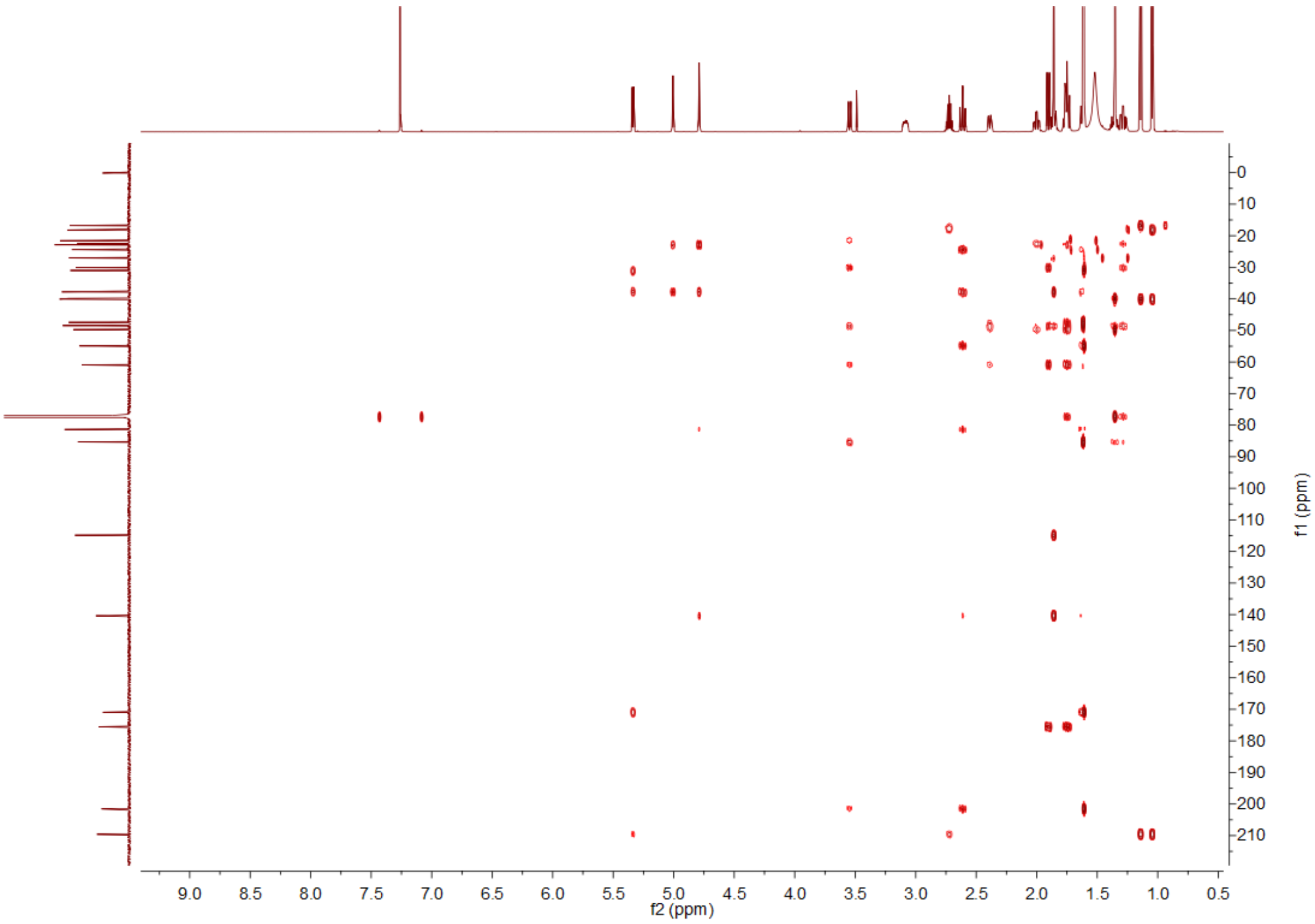

Figure S16. HMBC spectrum of compound **1** (recorded in $CDCl_3$).

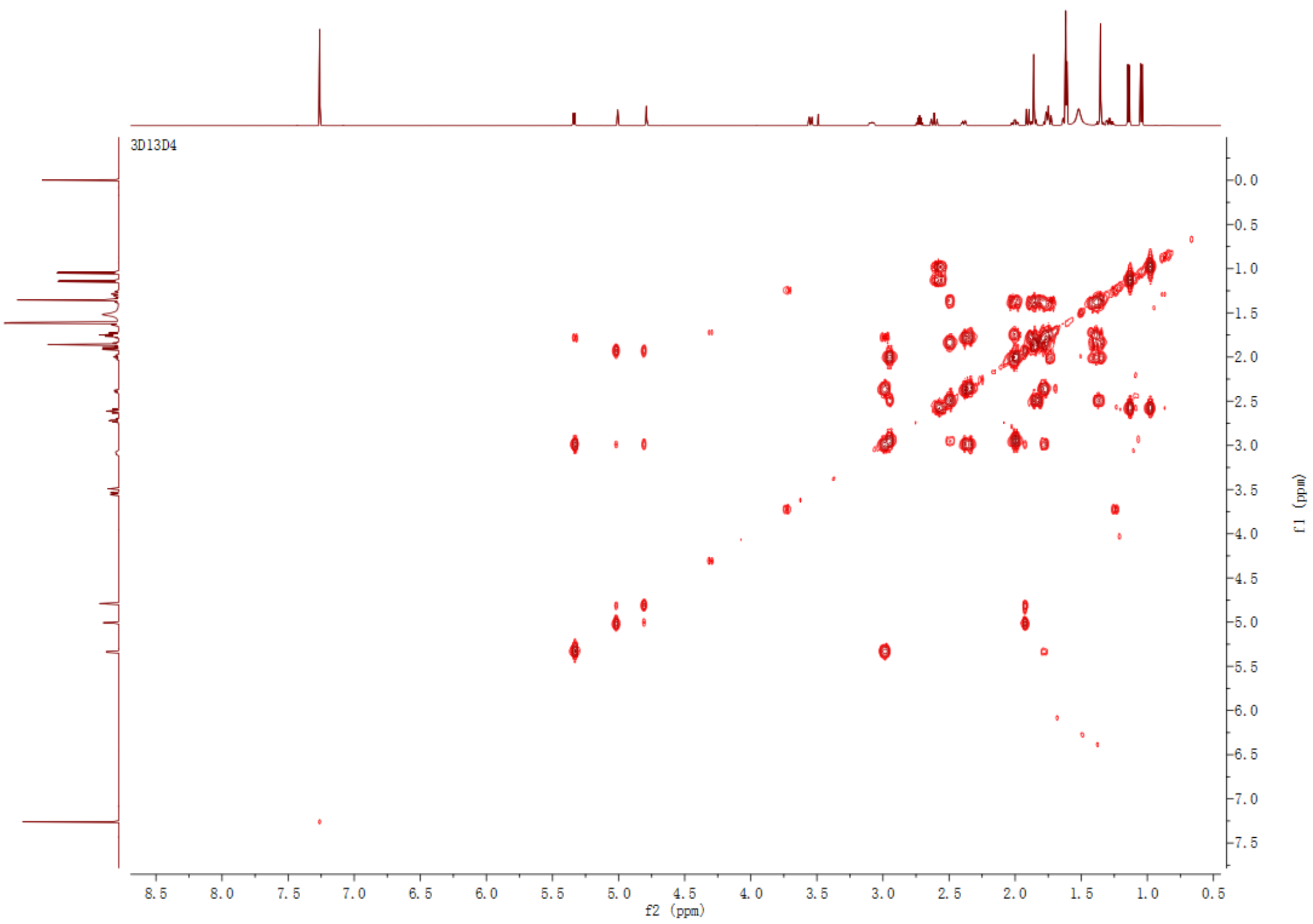

Figure S17. $^1H-^1H$ COSY spectrum of compound **1** (recorded in $CDCl_3$).

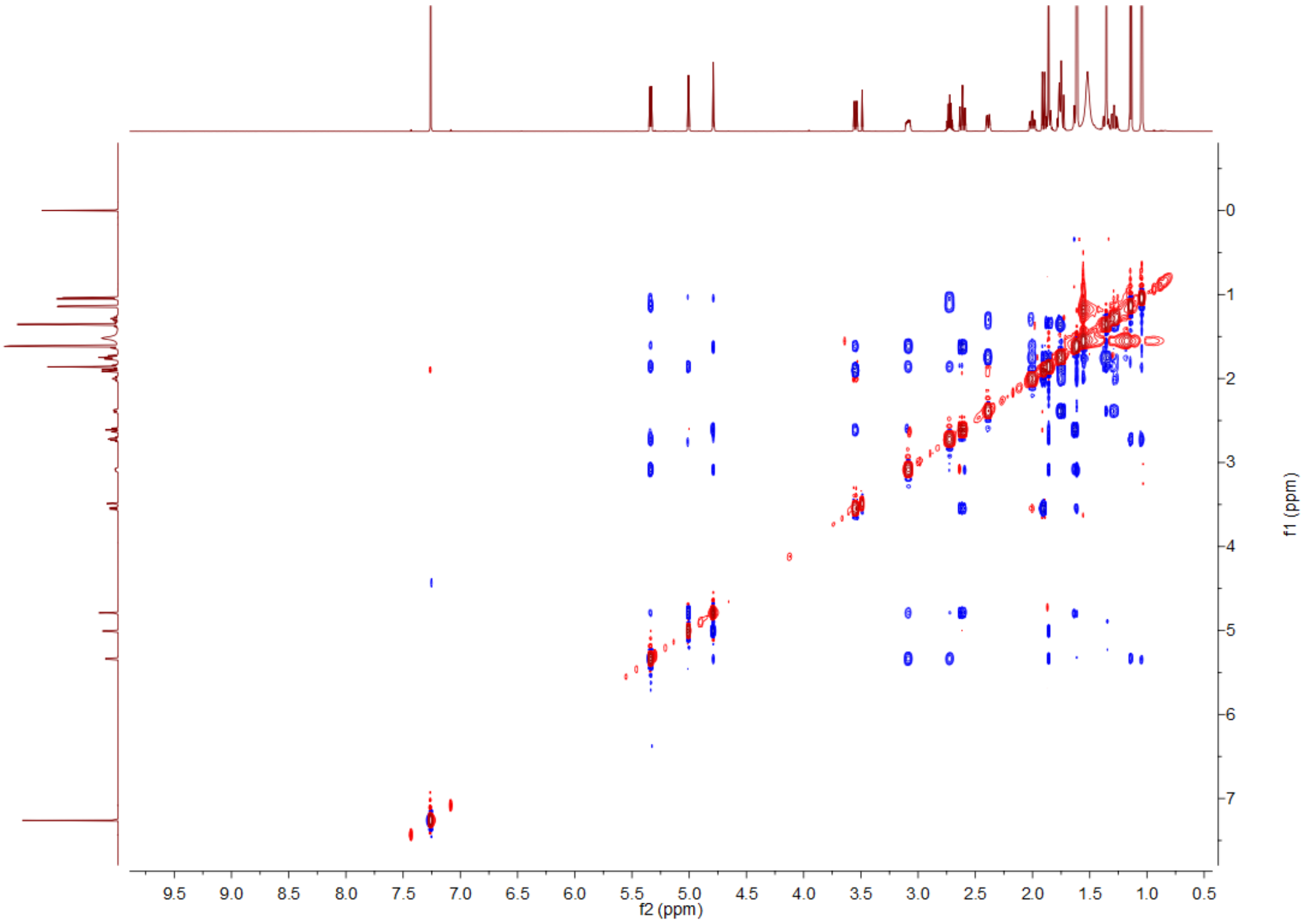

Figure S18. ROESY spectrum of compound **1** (recorded in $CDCl_3$).

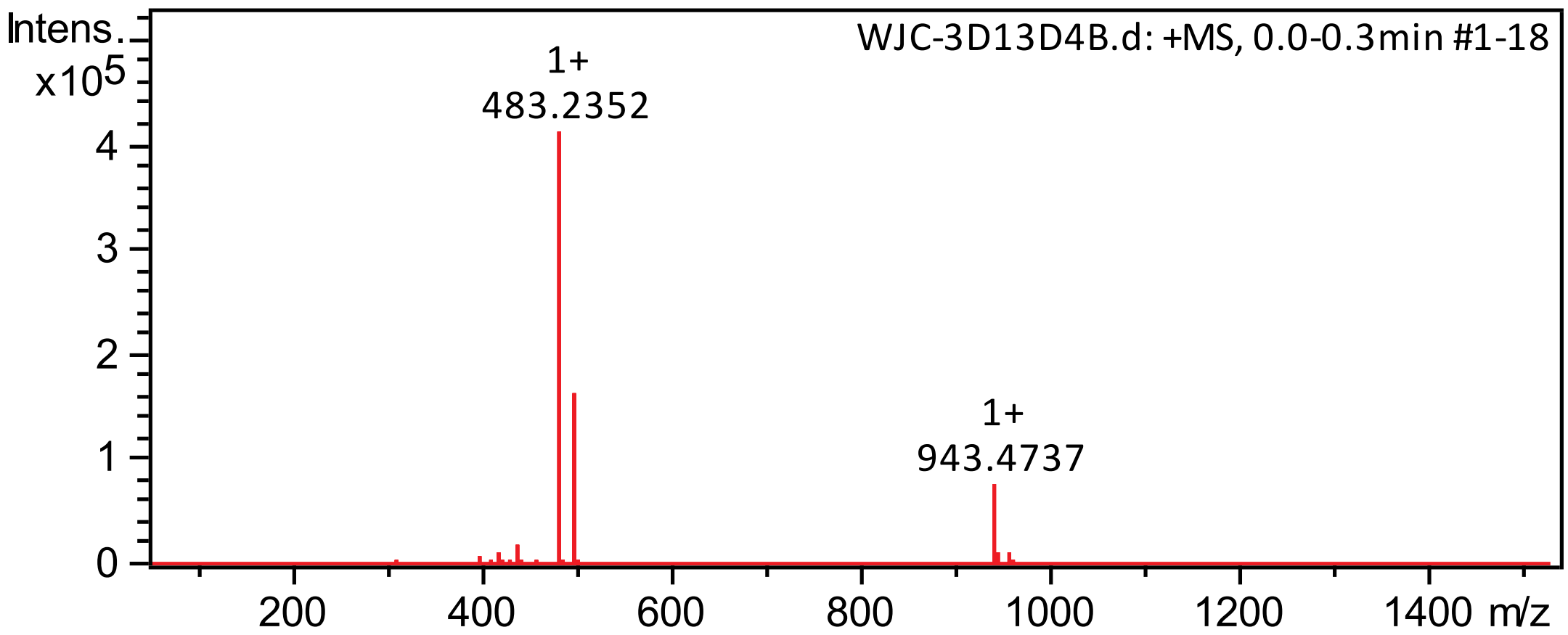

Figure S19. HRESIMS spectrum of compound **1**.

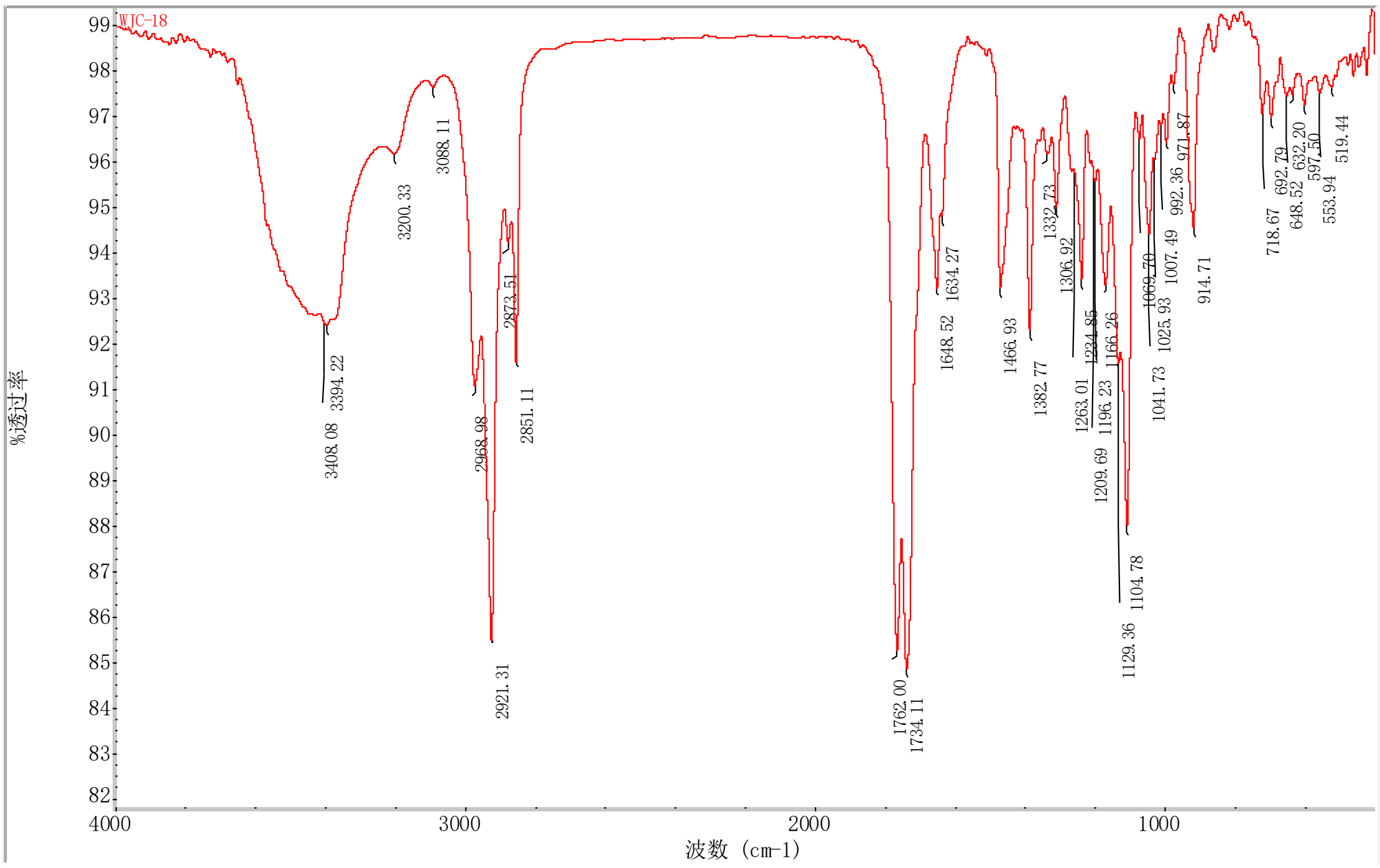

Figure S20. IR spectrum of compound **1**.

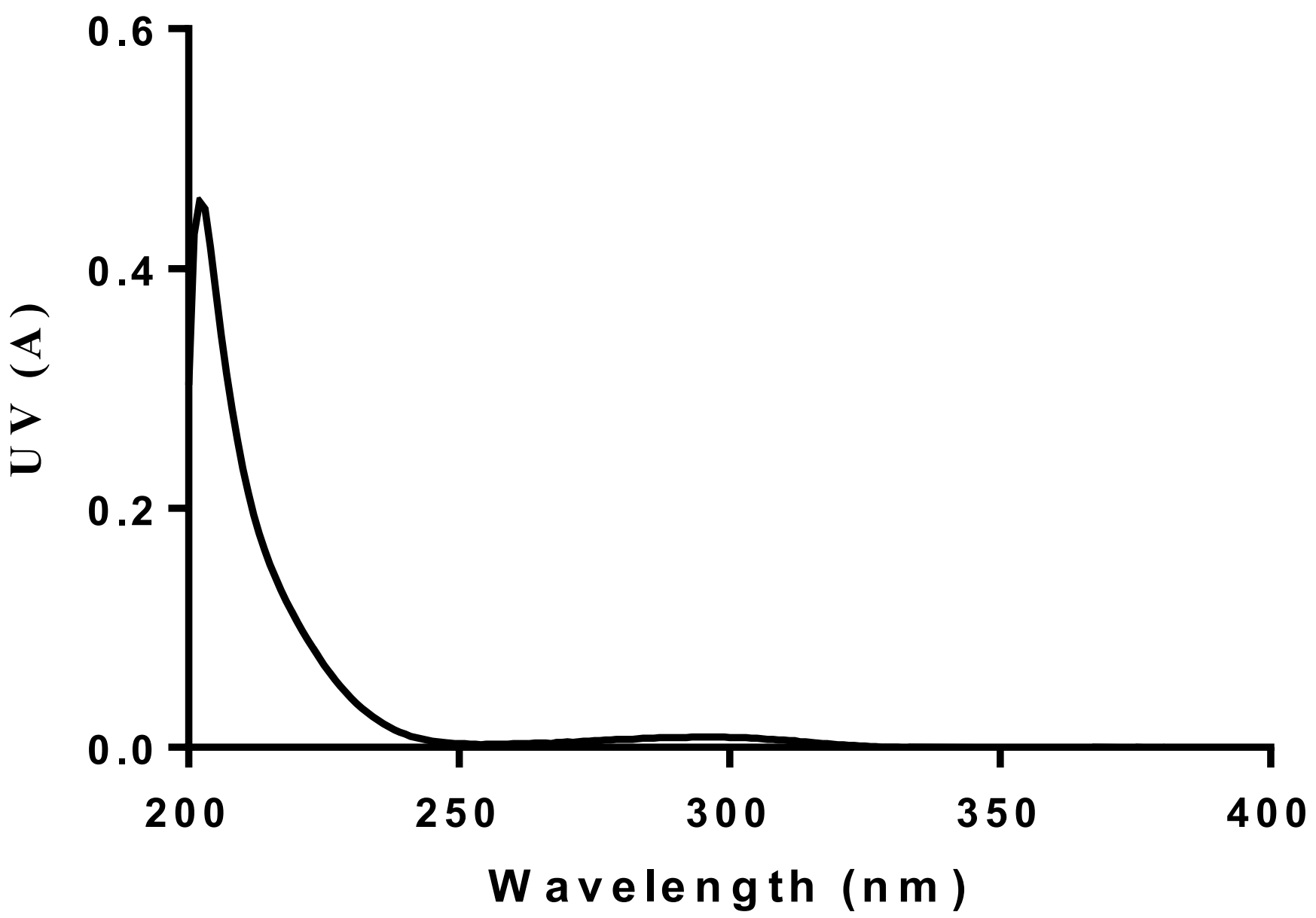

Figure S21. UV spectrum of compound **1**.

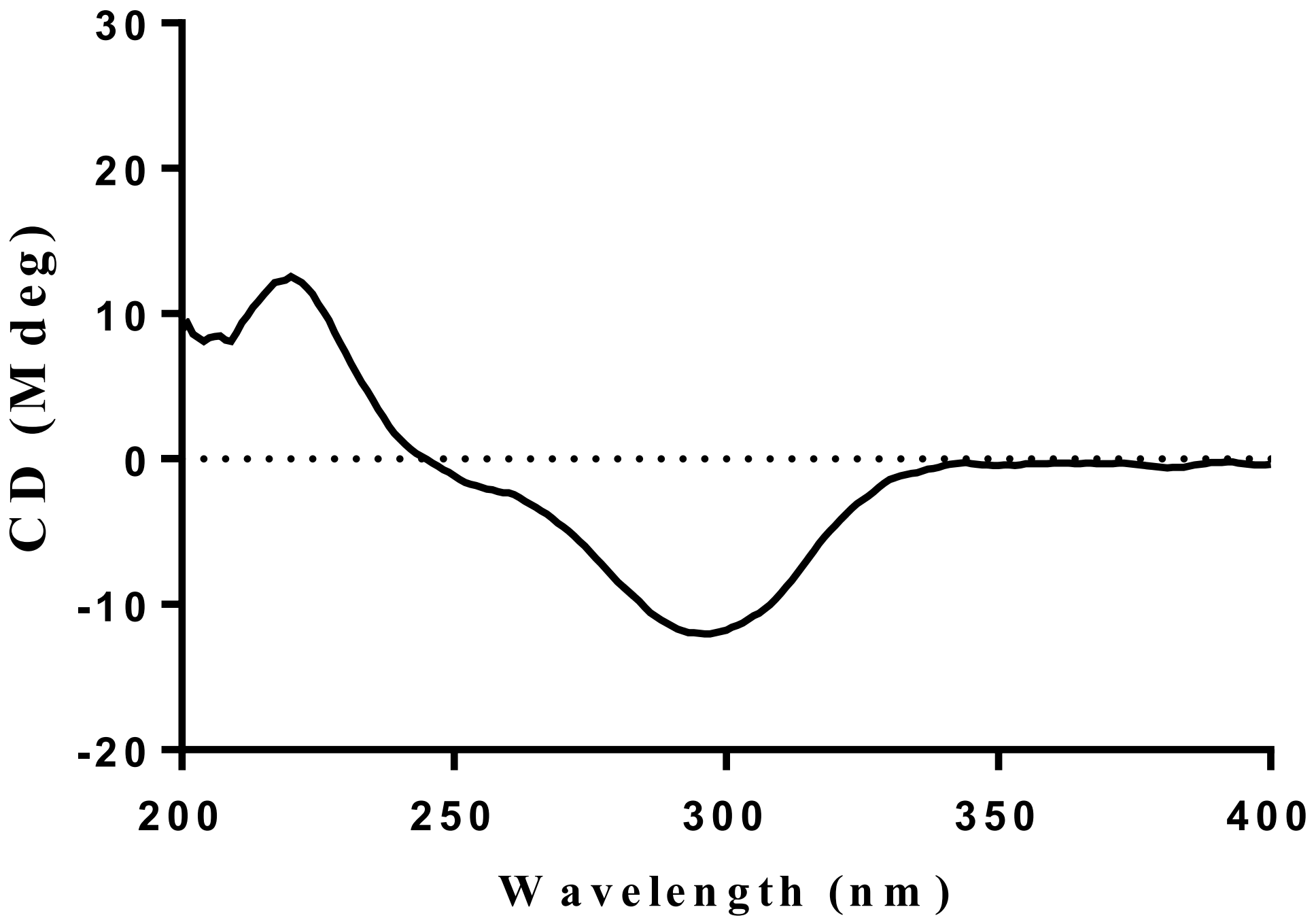

Figure S22. CD spectrum of **1** in MeOH.

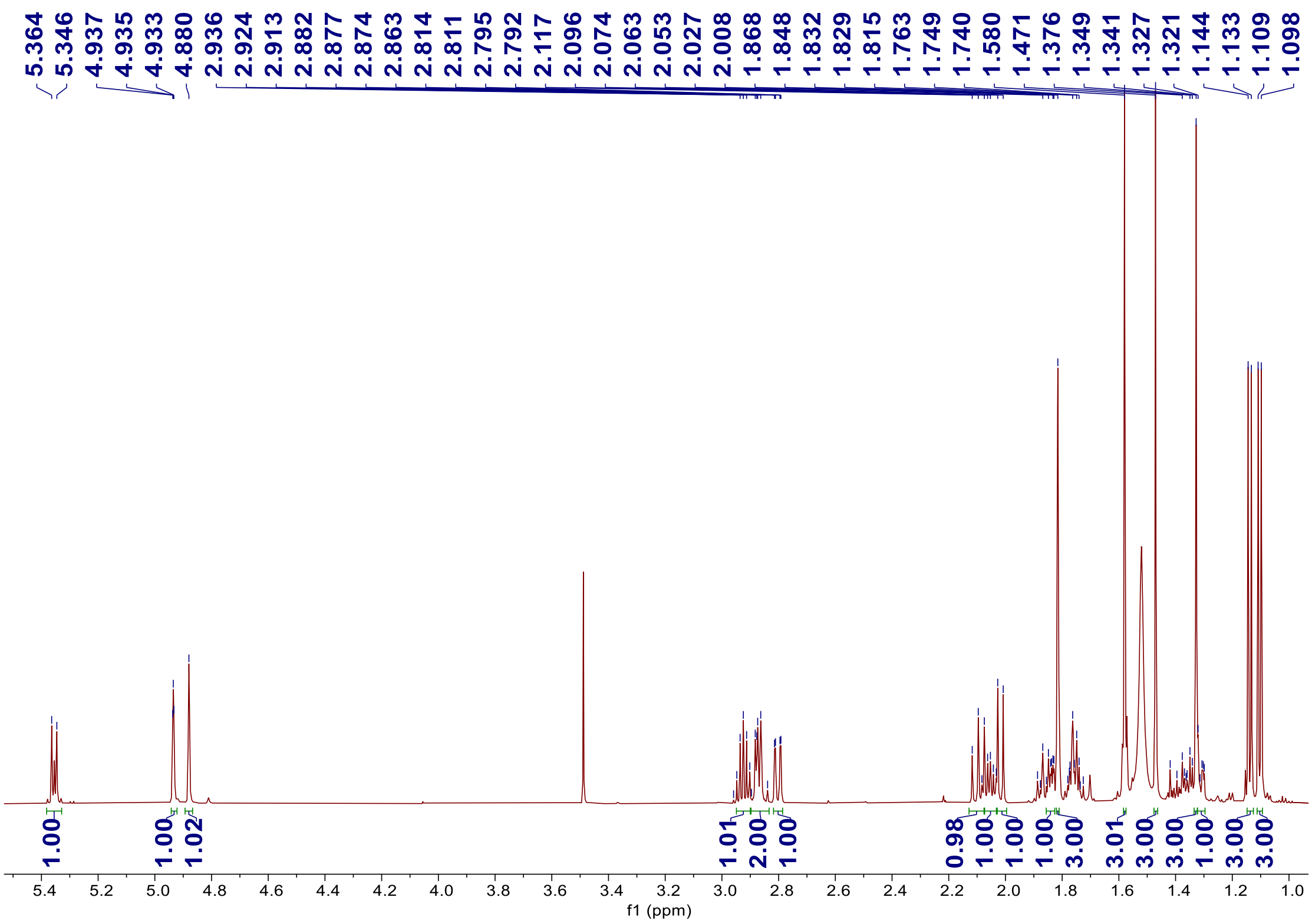

Figure S23. ^{1}H NMR spectrum of compound **2** (recorded in $CDCl_3$).

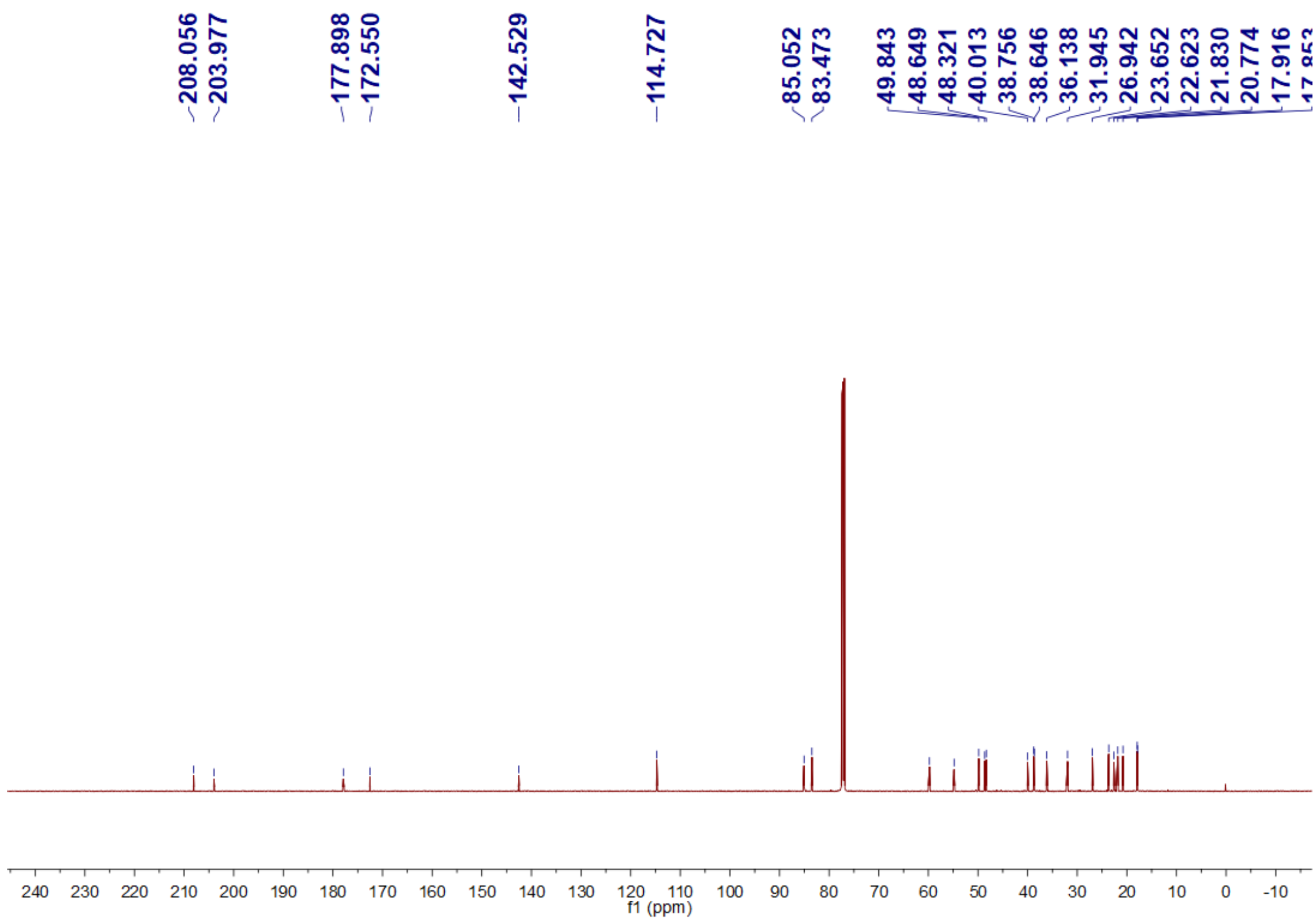

Figure S24. ^{13}C NMR spectrum of compound **2** (recorded in $CDCl_3$).

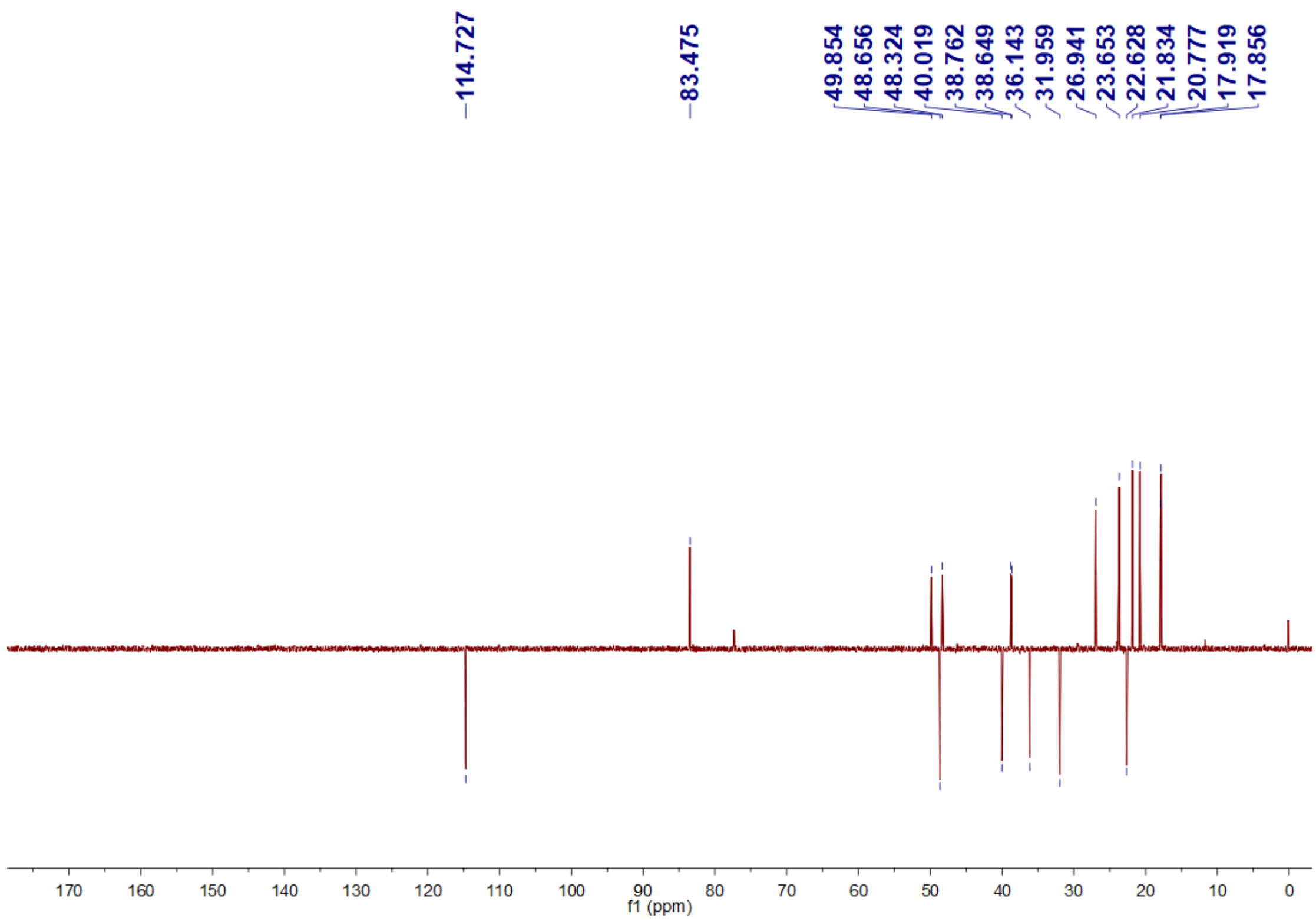

Figure S25. DEPT spectrum of compound **2** (recorded in $CDCl_3$).

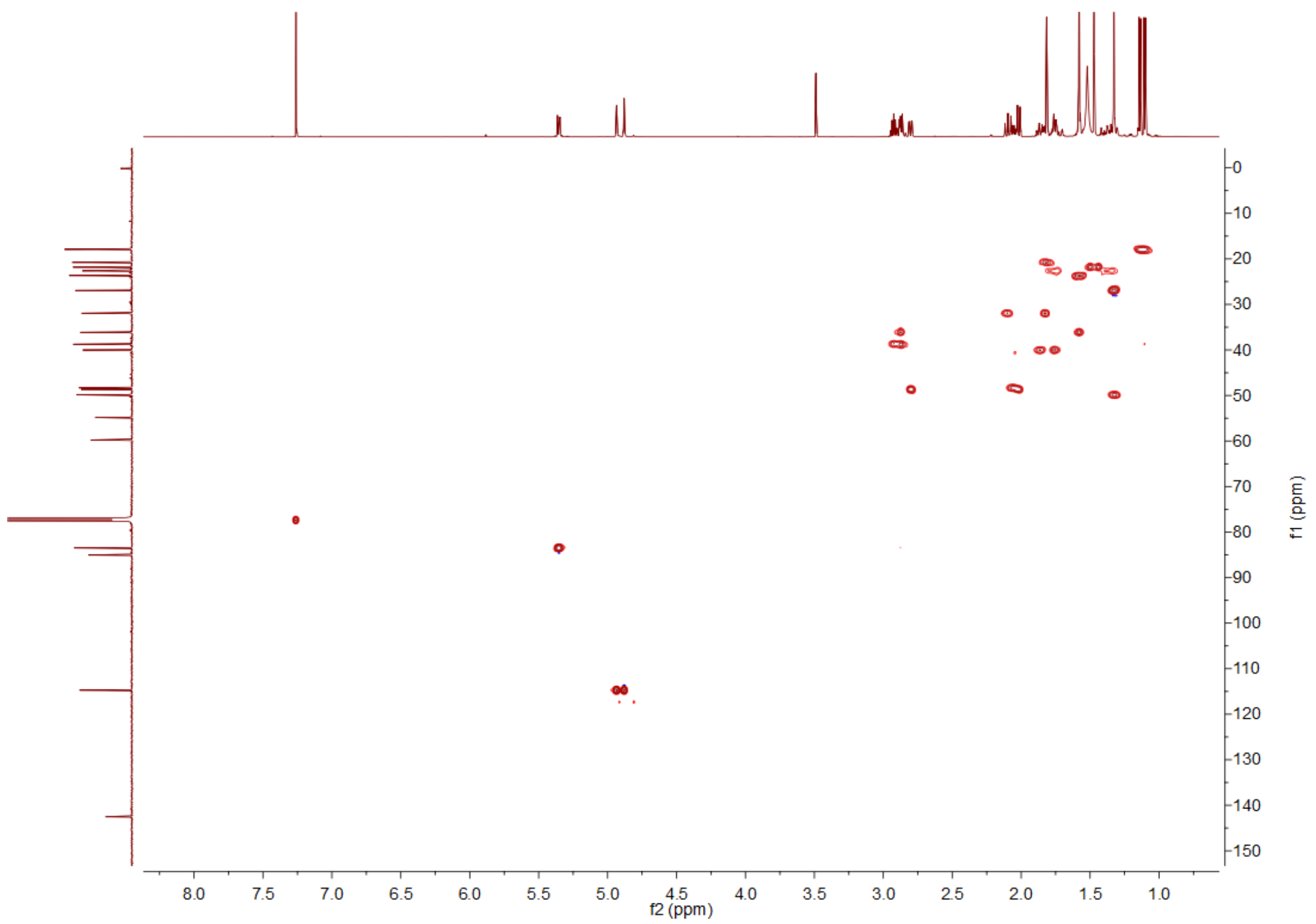

Figure S26. HSQC spectrum of compound **2** (recorded in $CDCl_3$).

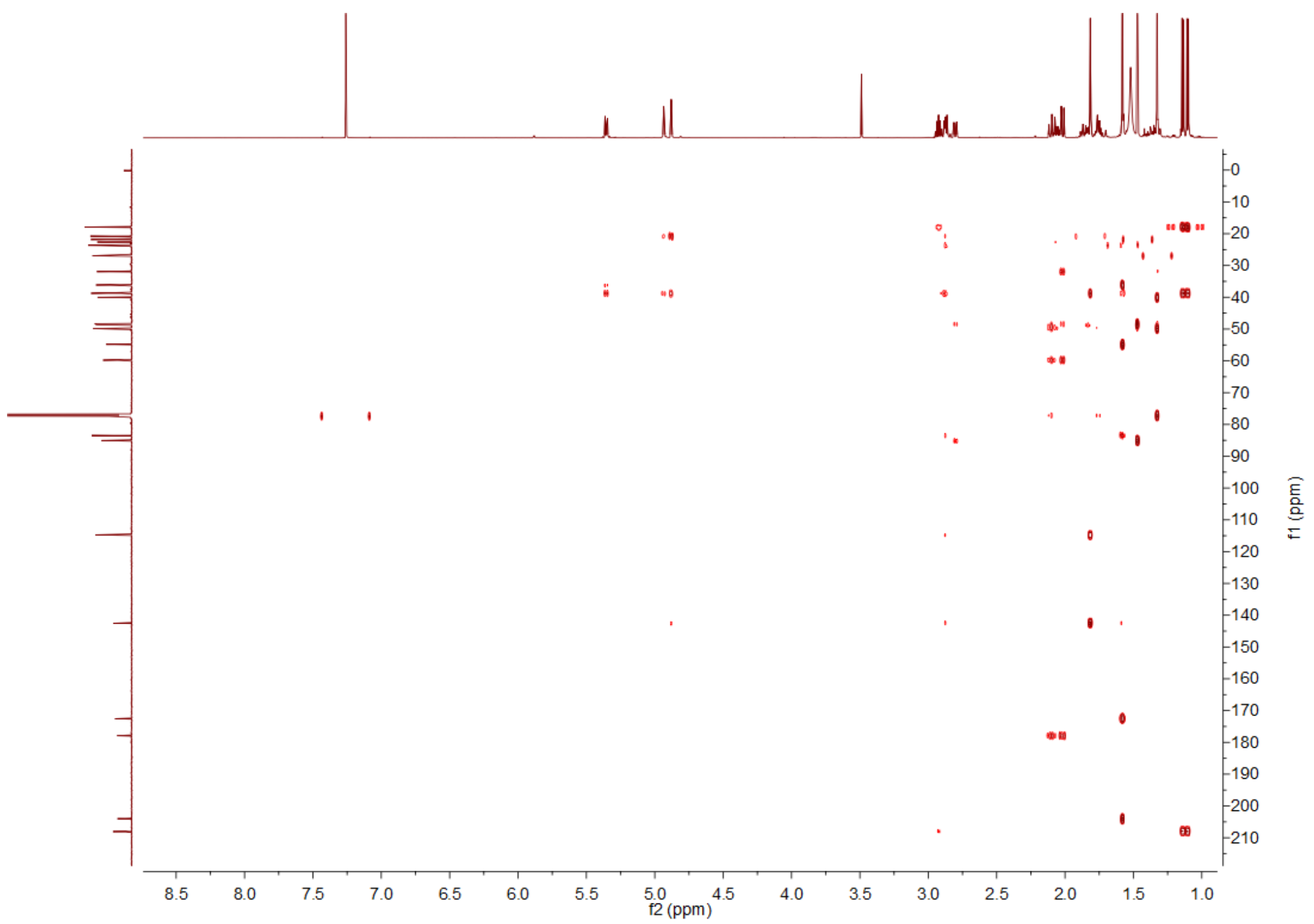

Figure S27. HMBC spectrum of compound **2** (recorded in $CDCl_3$).

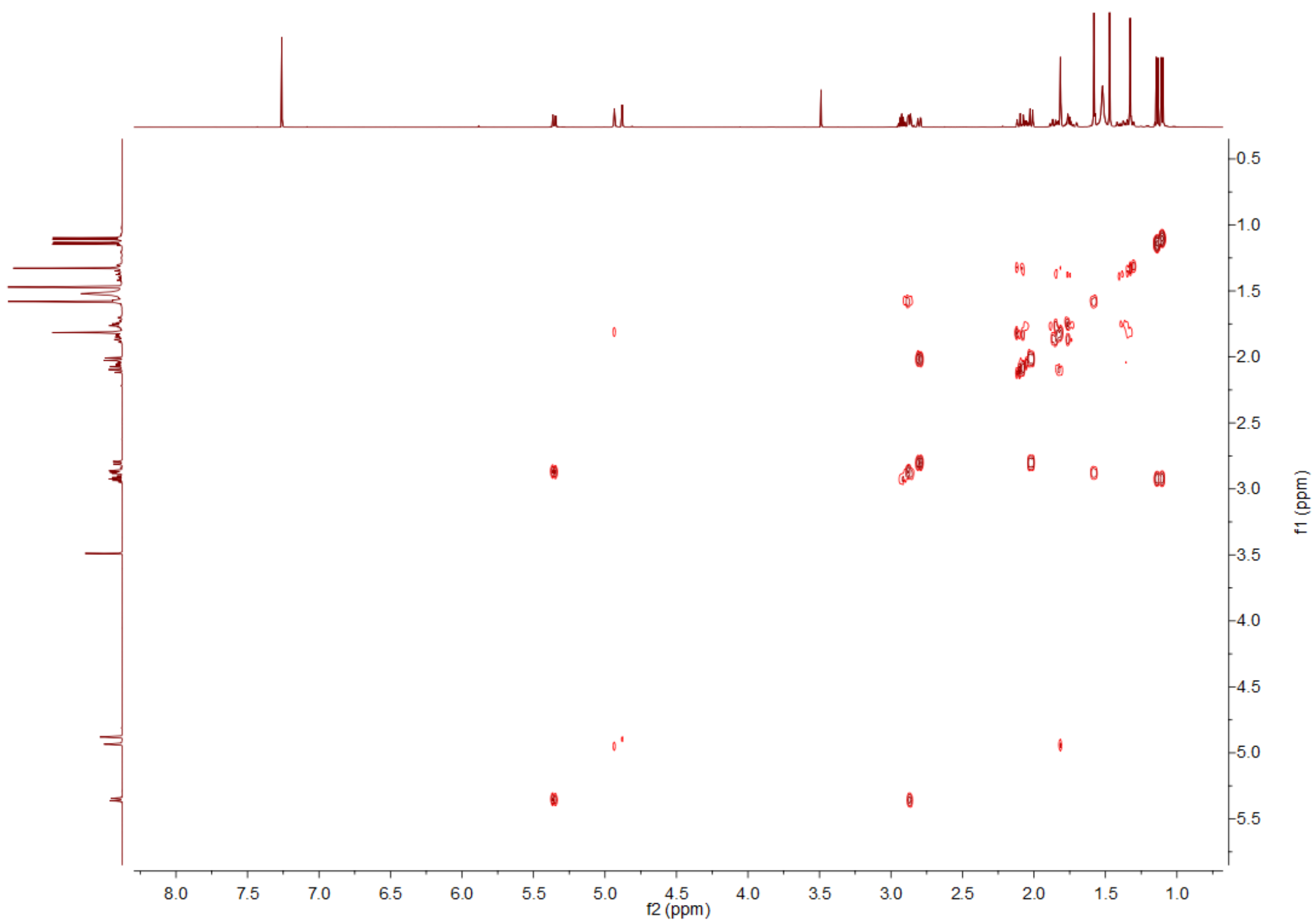

Figure S28. $^{1}H–^{1}H$ COSY spectrum of compound **2** (recorded in $CDCl_3$).

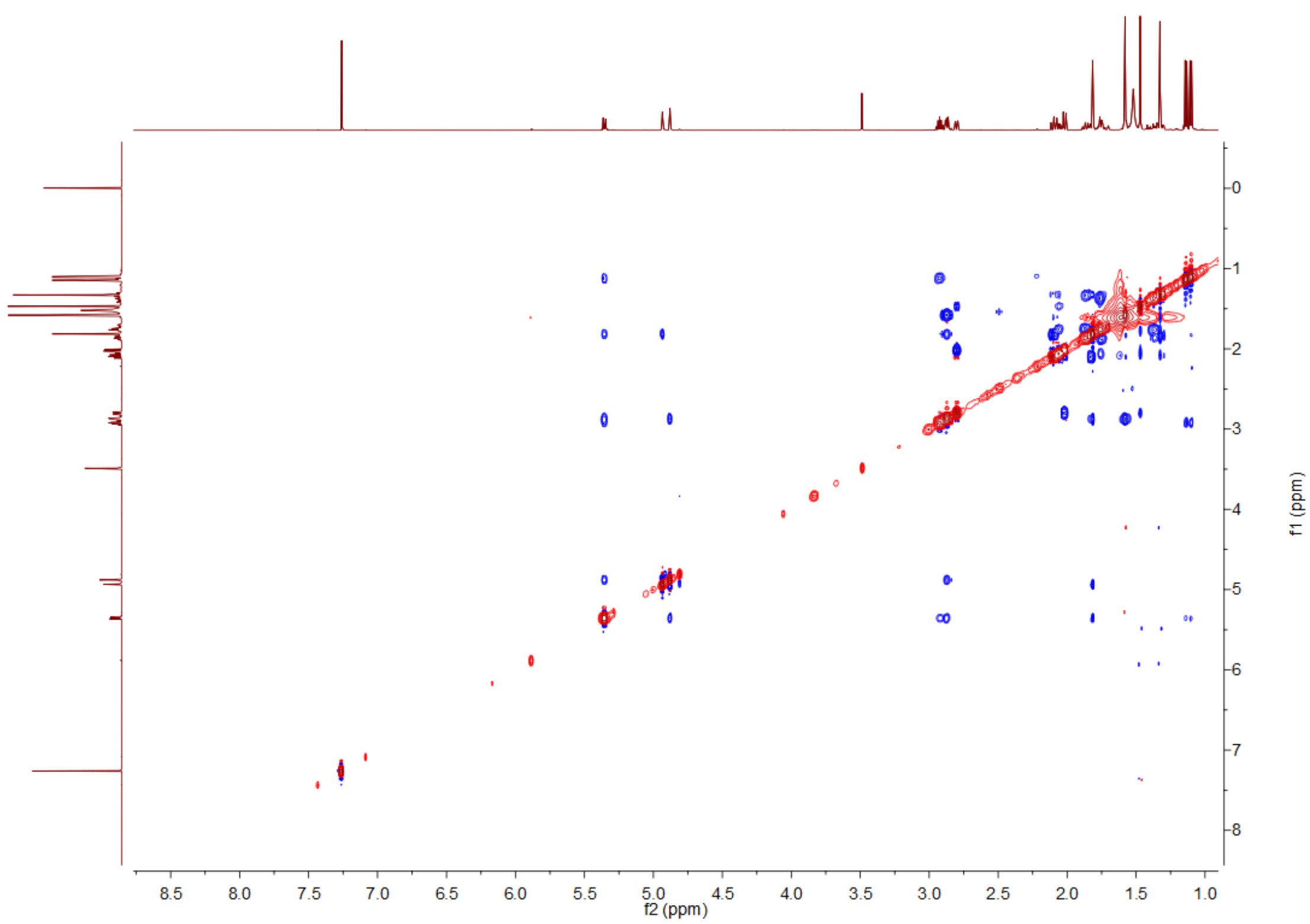

Figure S29. ROESY spectrum of compound **2** (recorded in $CDCl_3$).

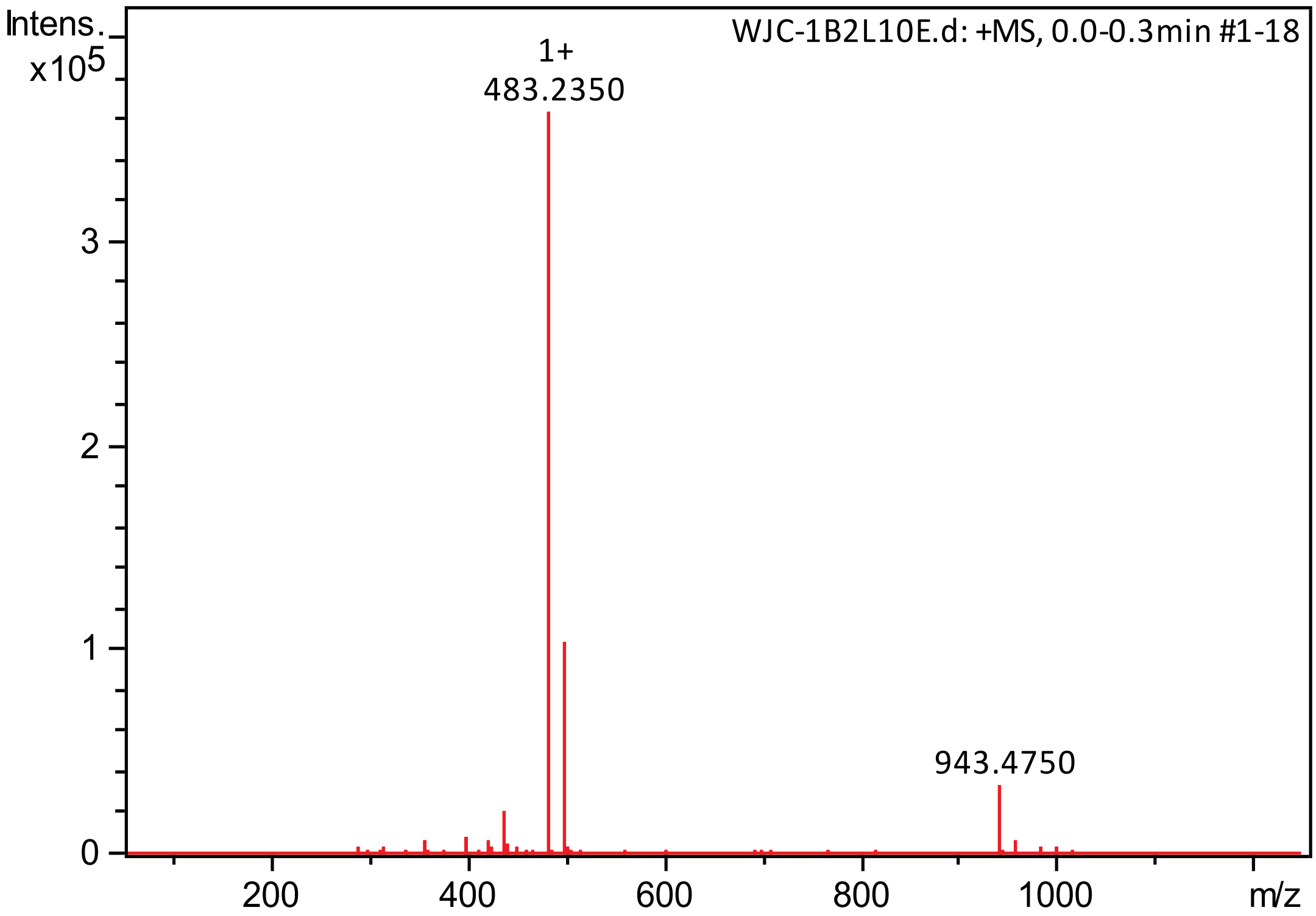

Figure S30. HRESIMS spectrum of compound **2**.

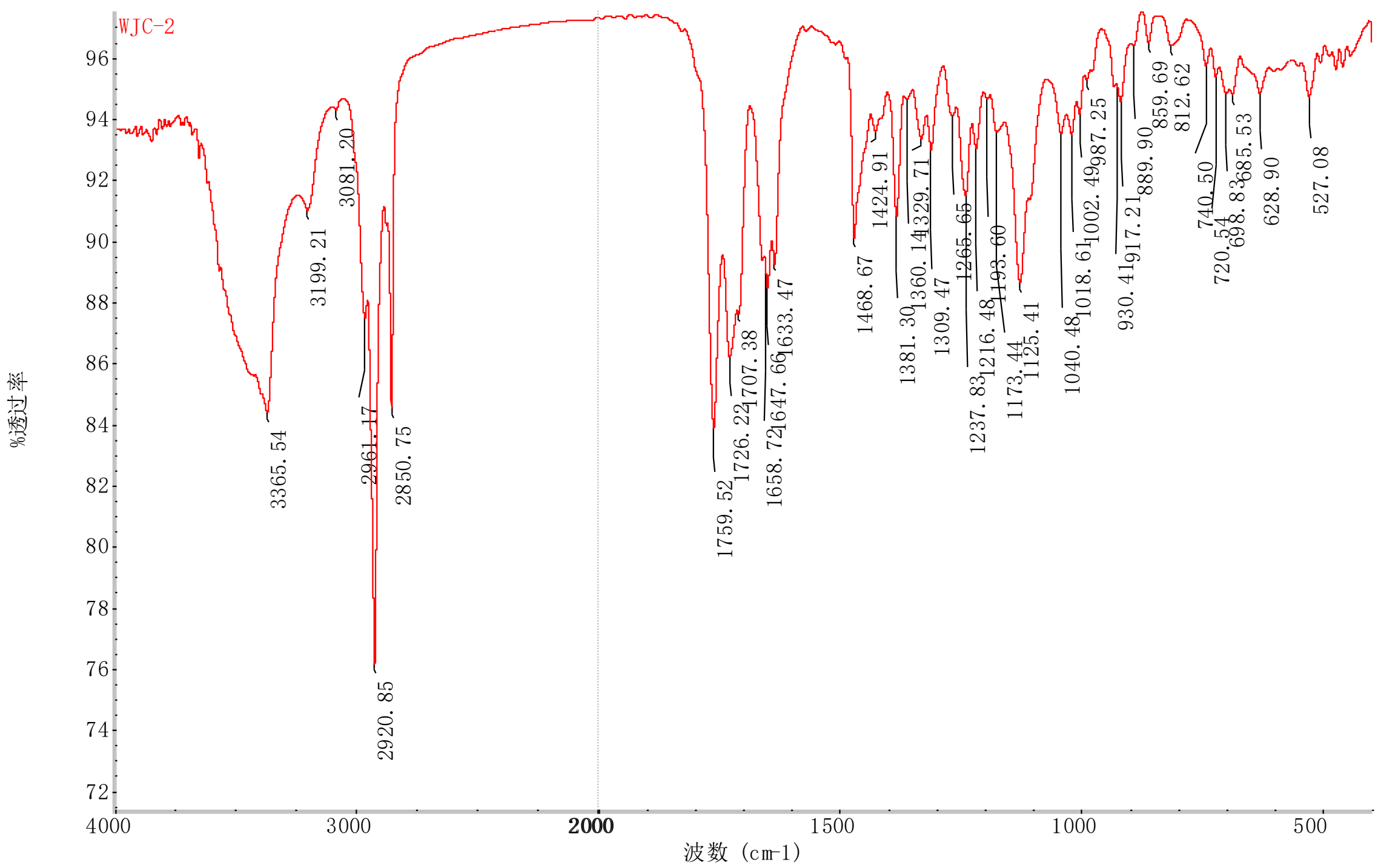

Figure S31. IR spectrum of compound **2**.

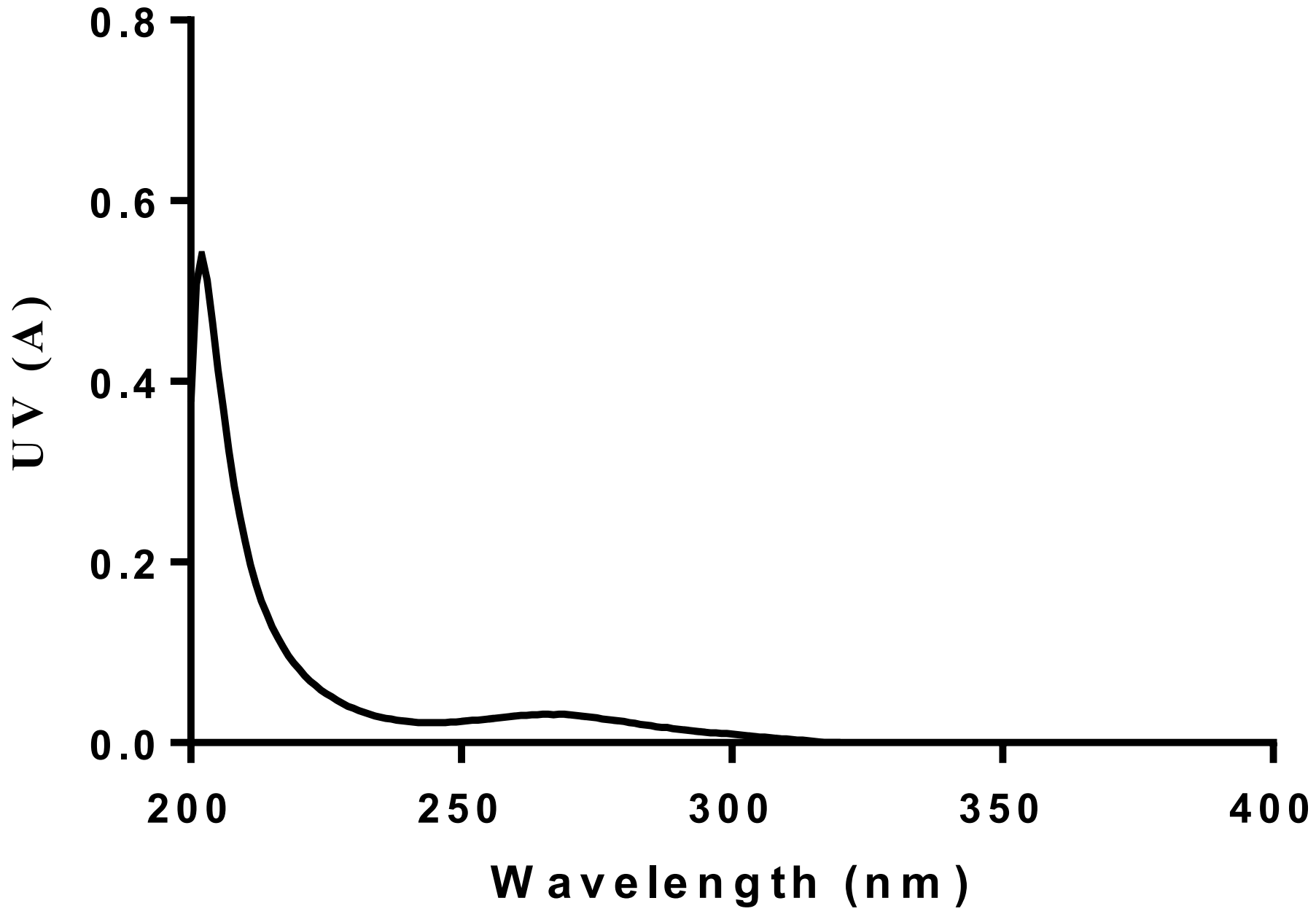

Figure S32. UV spectrum of compound **2**.

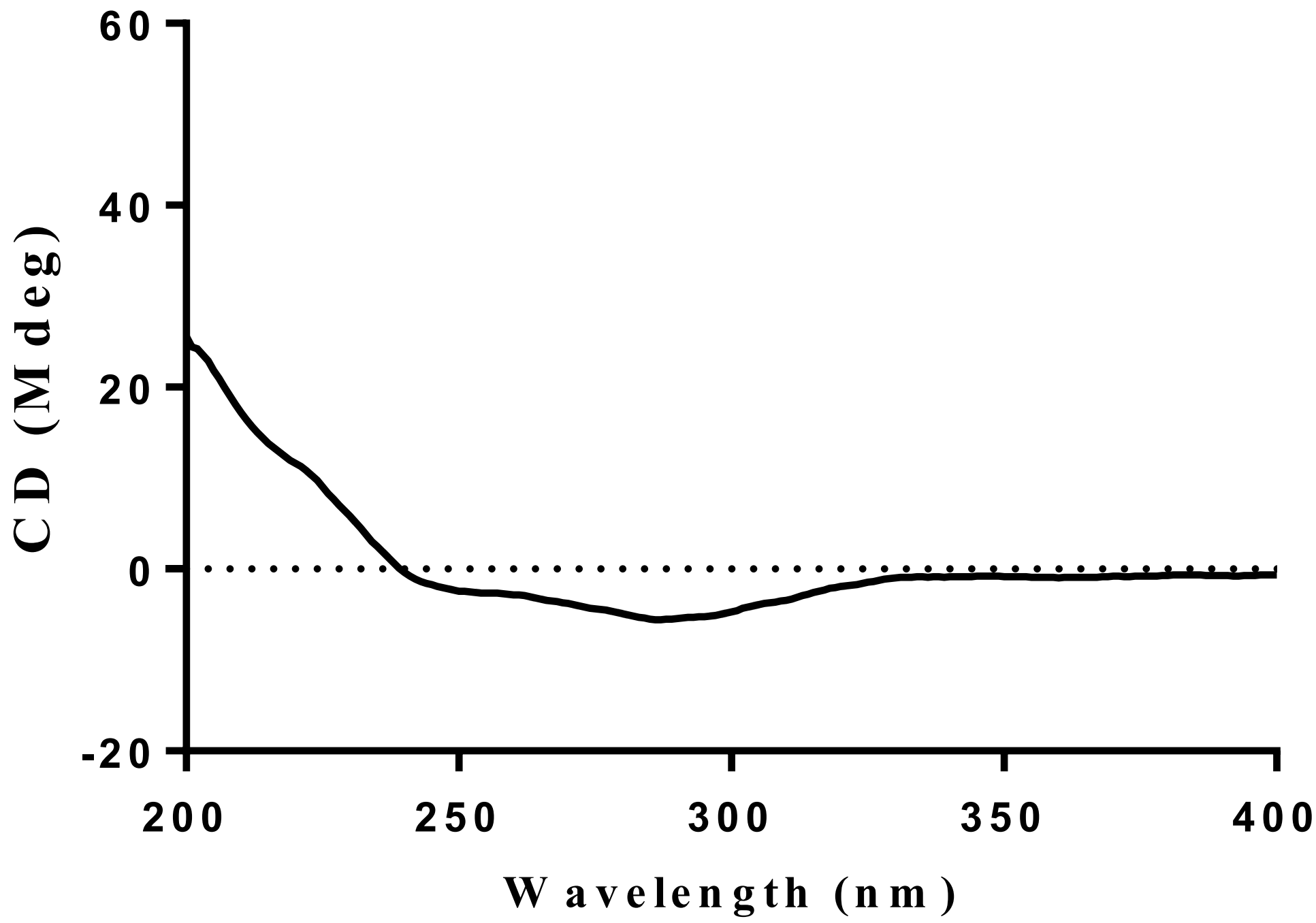

Figure S33. CD spectrum of **2** in MeOH.

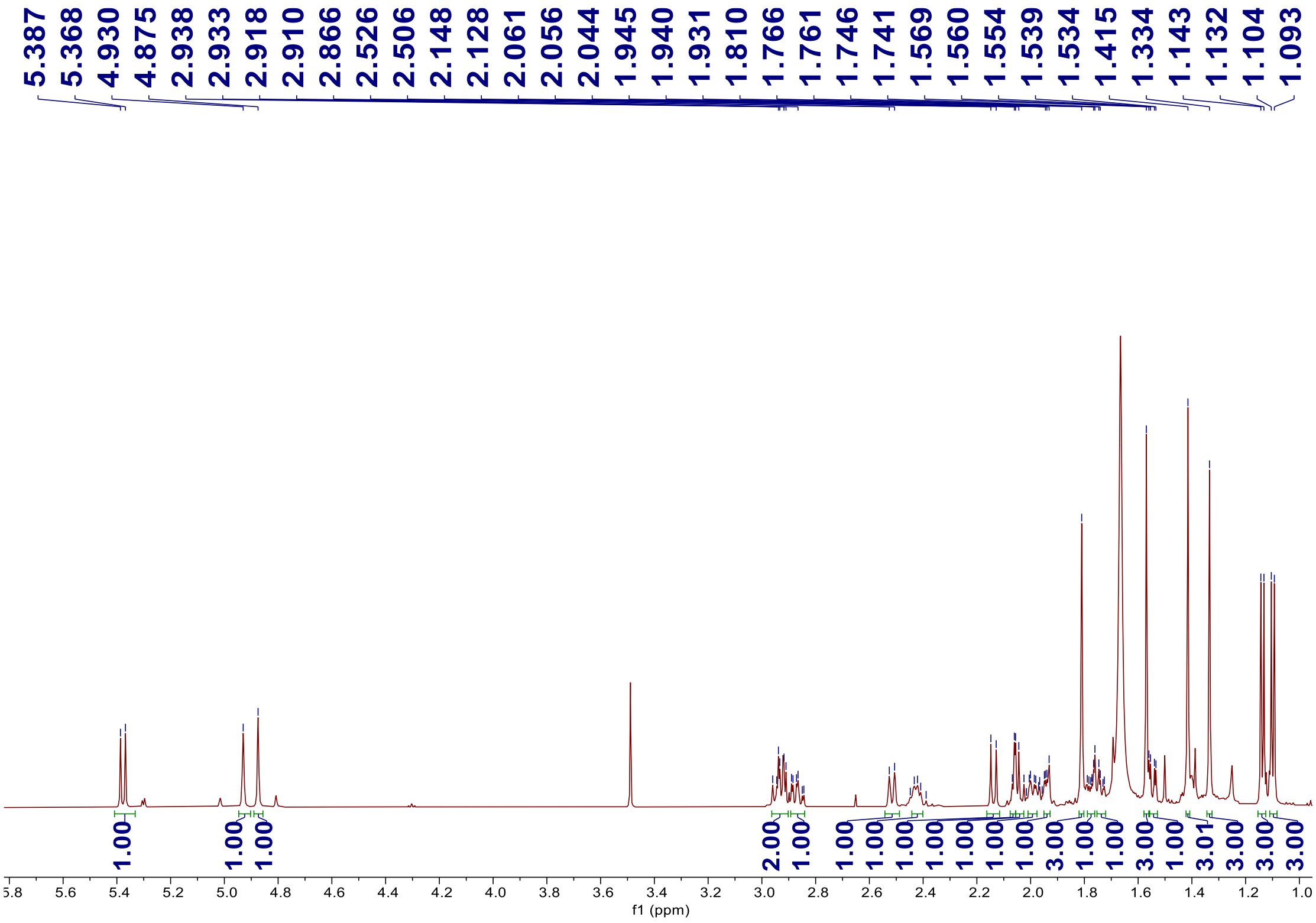

Figure S34. ^{1}H NMR spectrum of compound **3** (recorded in $CDCl_3$).

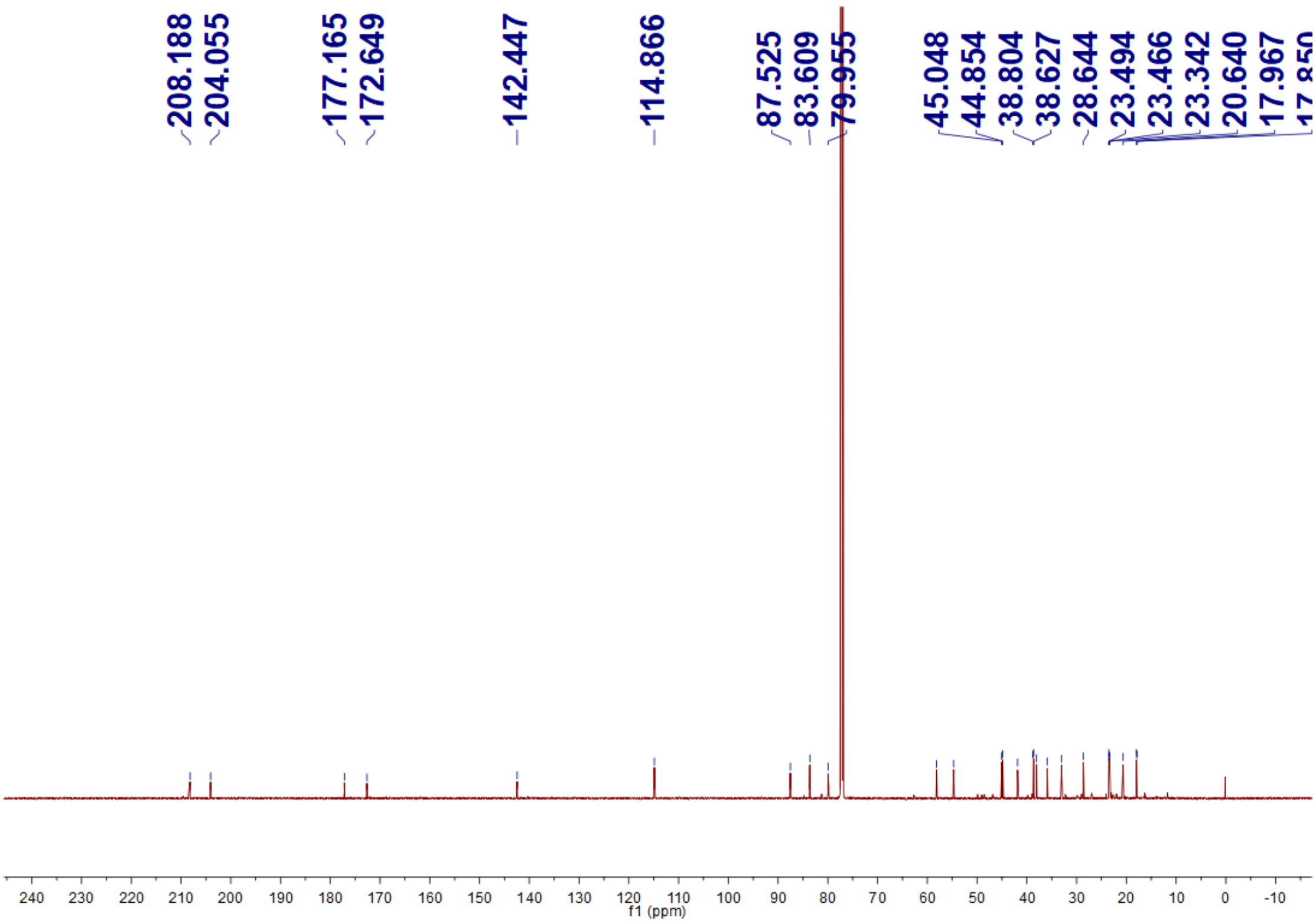

Figure S35. ^{13}C NMR spectrum of compound **3** (recorded in $CDCl_3$).

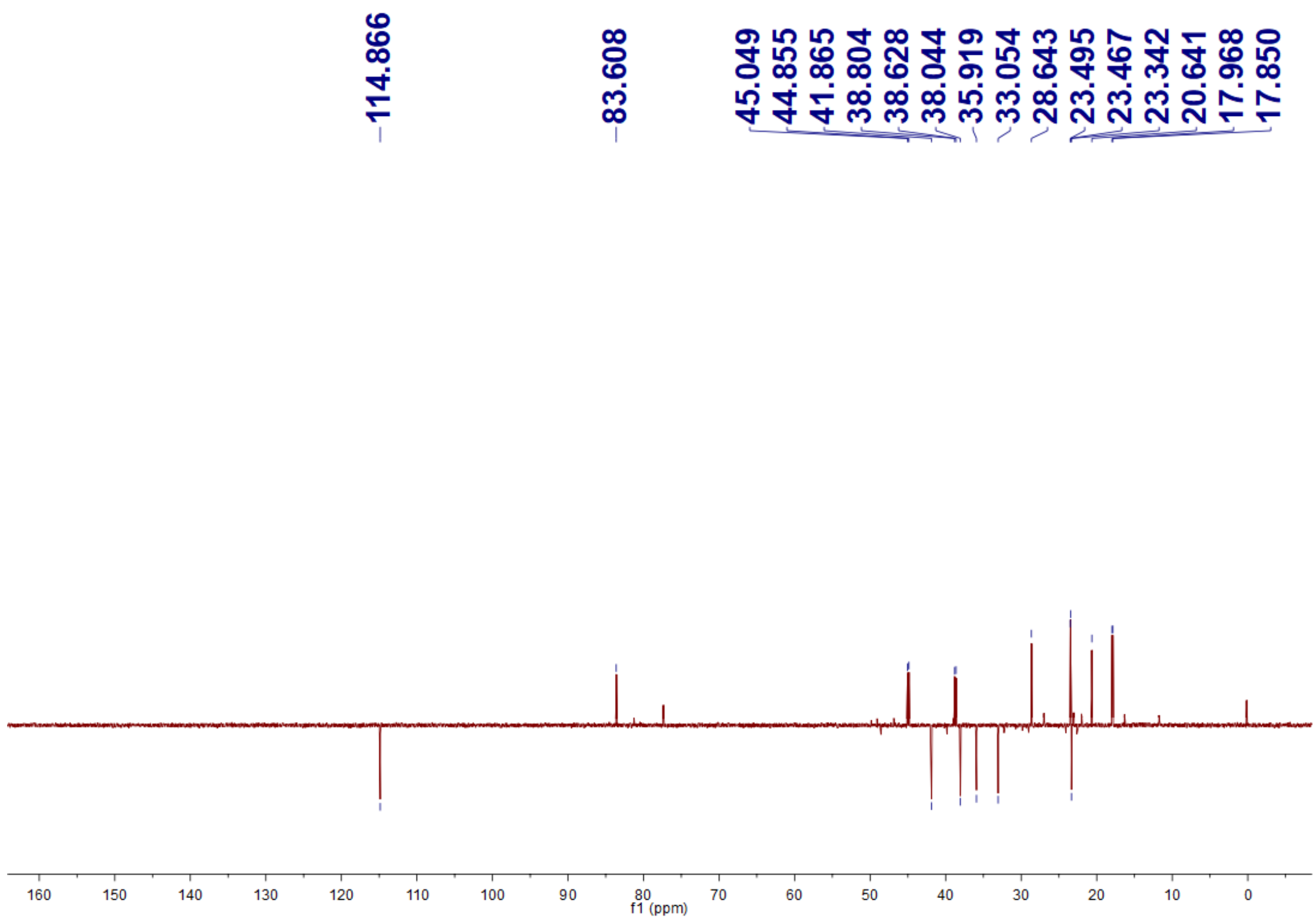

Figure S36. DEPT spectrum of compound **3** (recorded in $CDCl_3$).

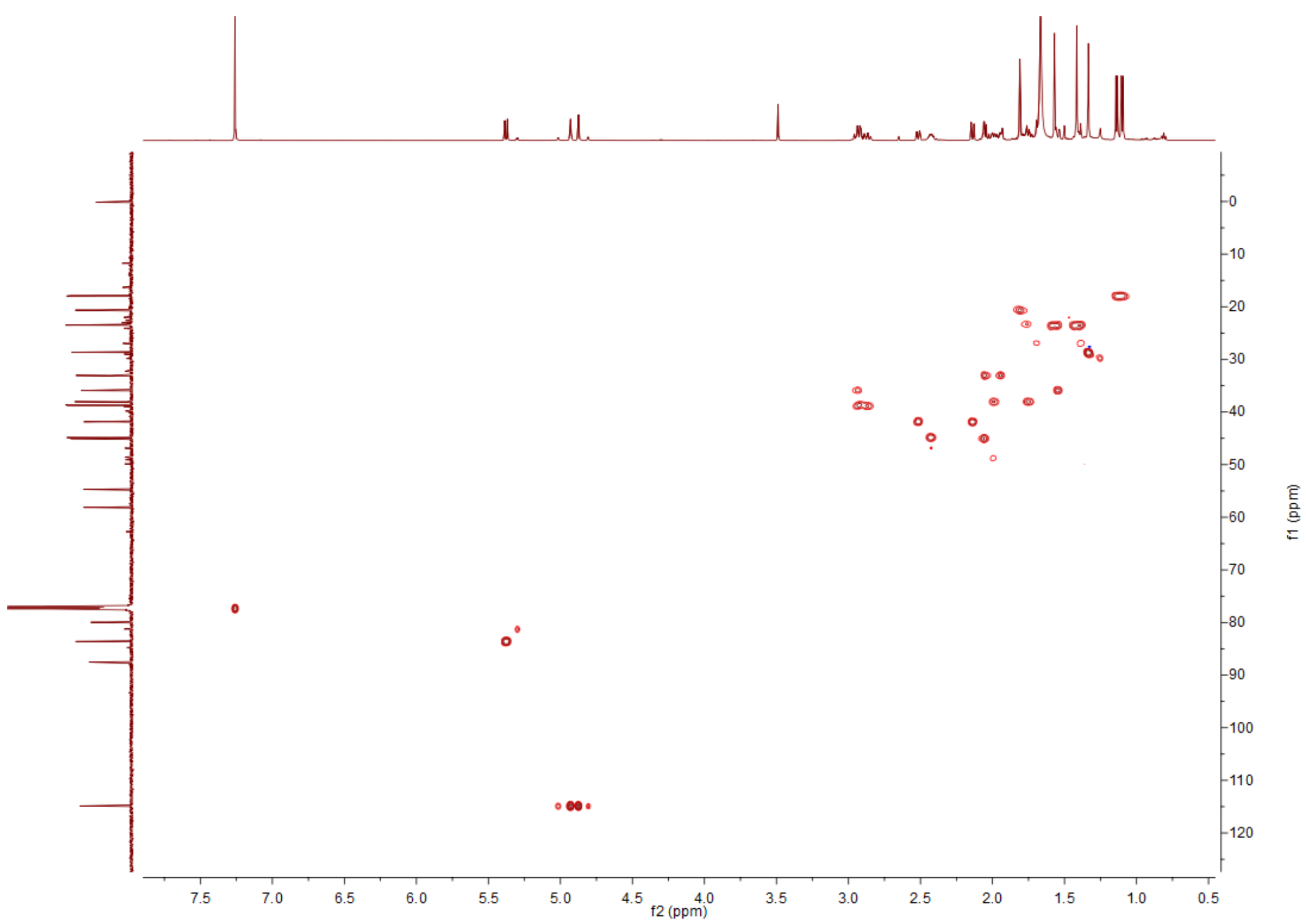

Figure S37. HSQC spectrum of compound **3** (recorded in $CDCl_3$).

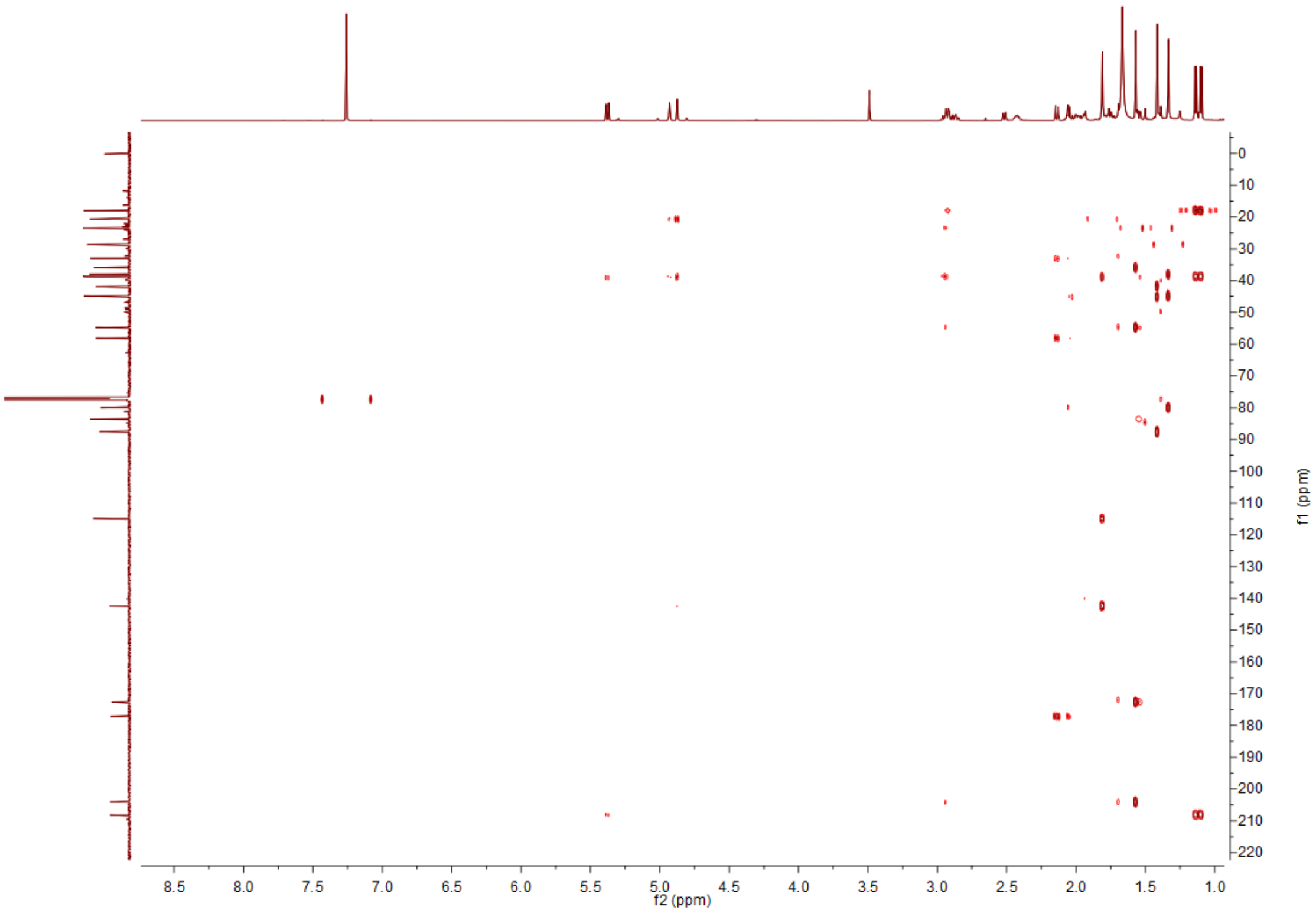

Figure S38. HMBC spectrum of compound **3** (recorded in $CDCl_3$).

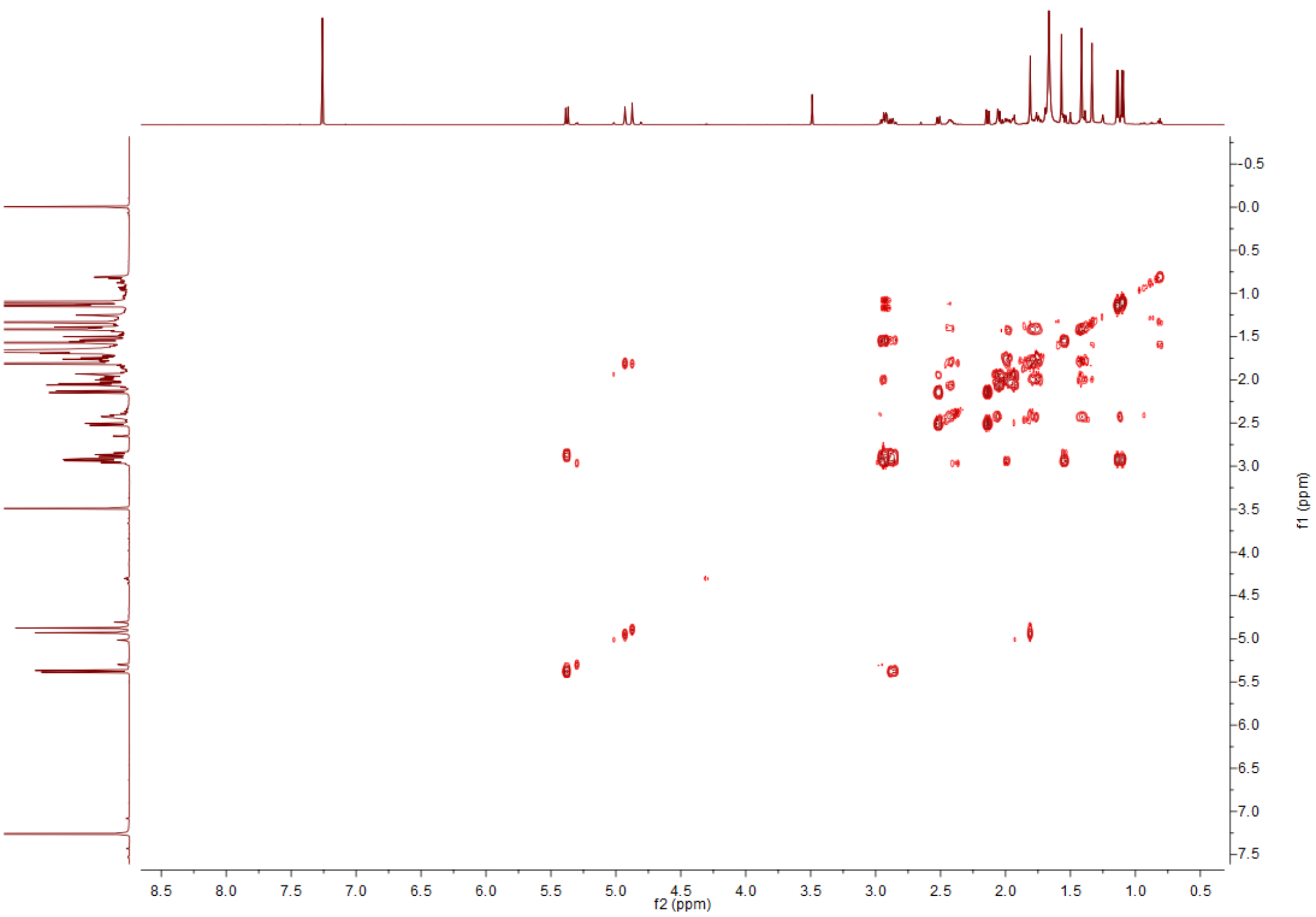

Figure S39. $^1H-^1H$ COSY spectrum of compound **3** (recorded in $CDCl_3$).

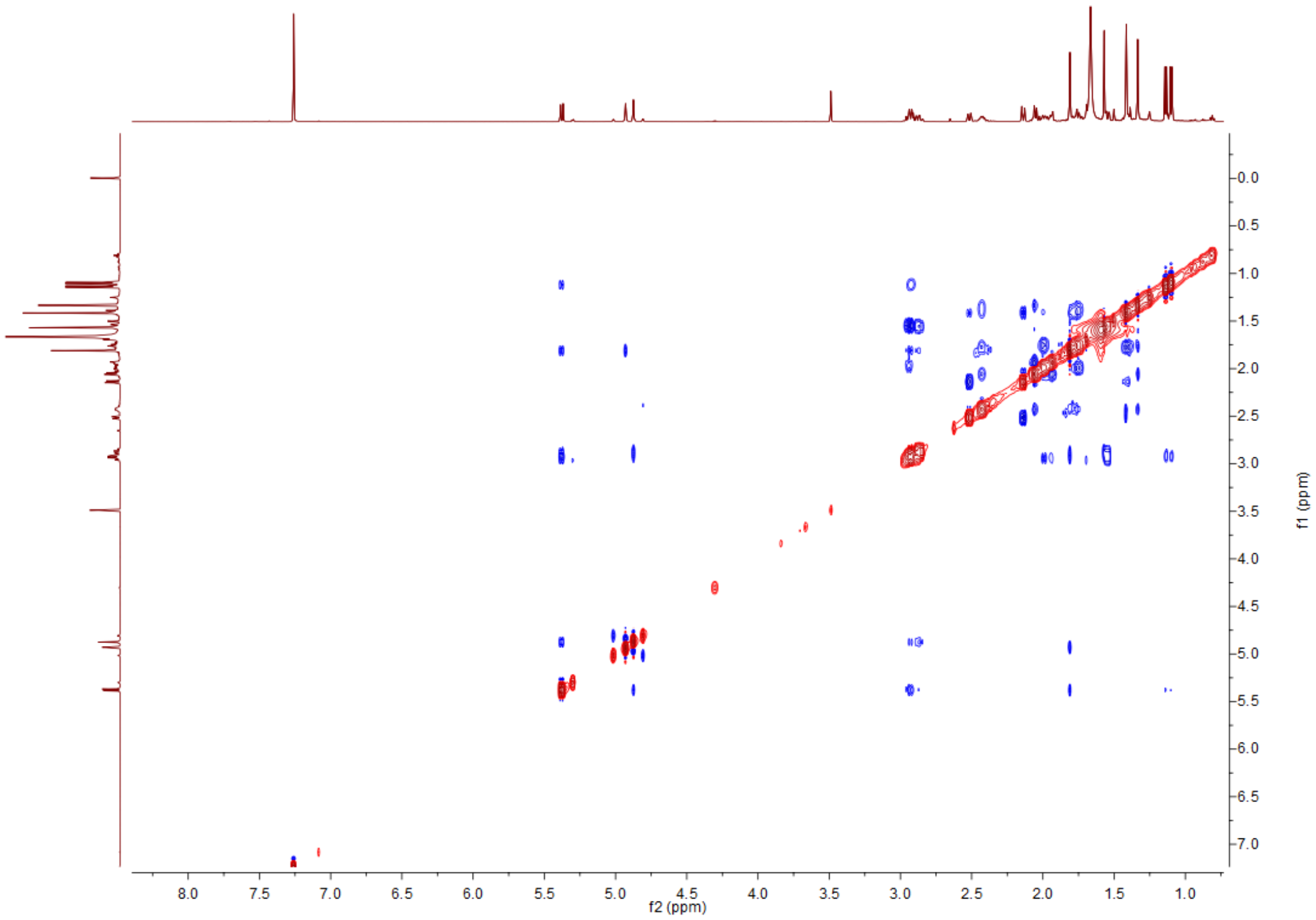

Figure S40. ROESY spectrum of compound **3** (recorded in $CDCl_3$).

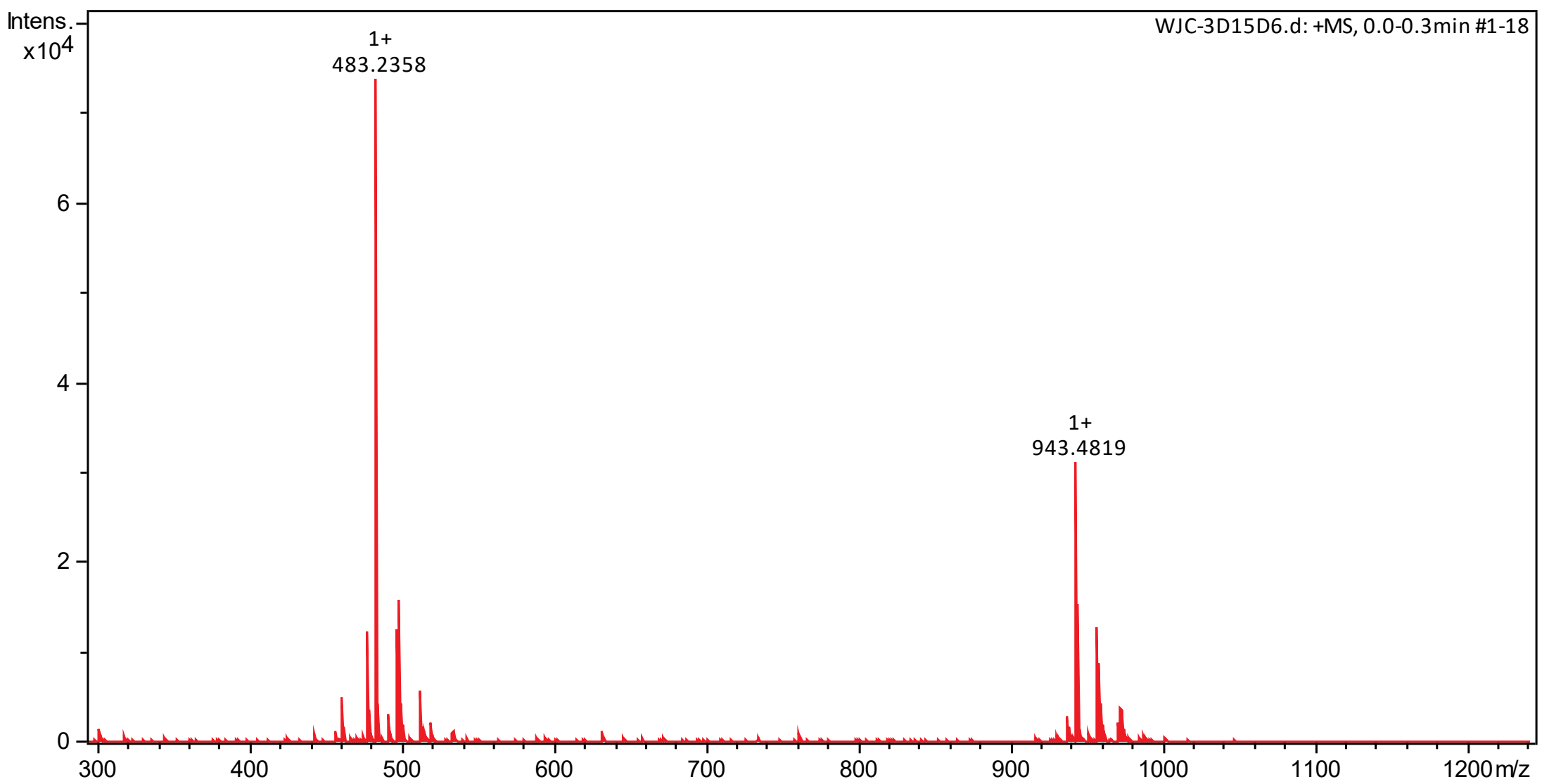

Figure S41. HRESIMS spectrum of compound **3**.

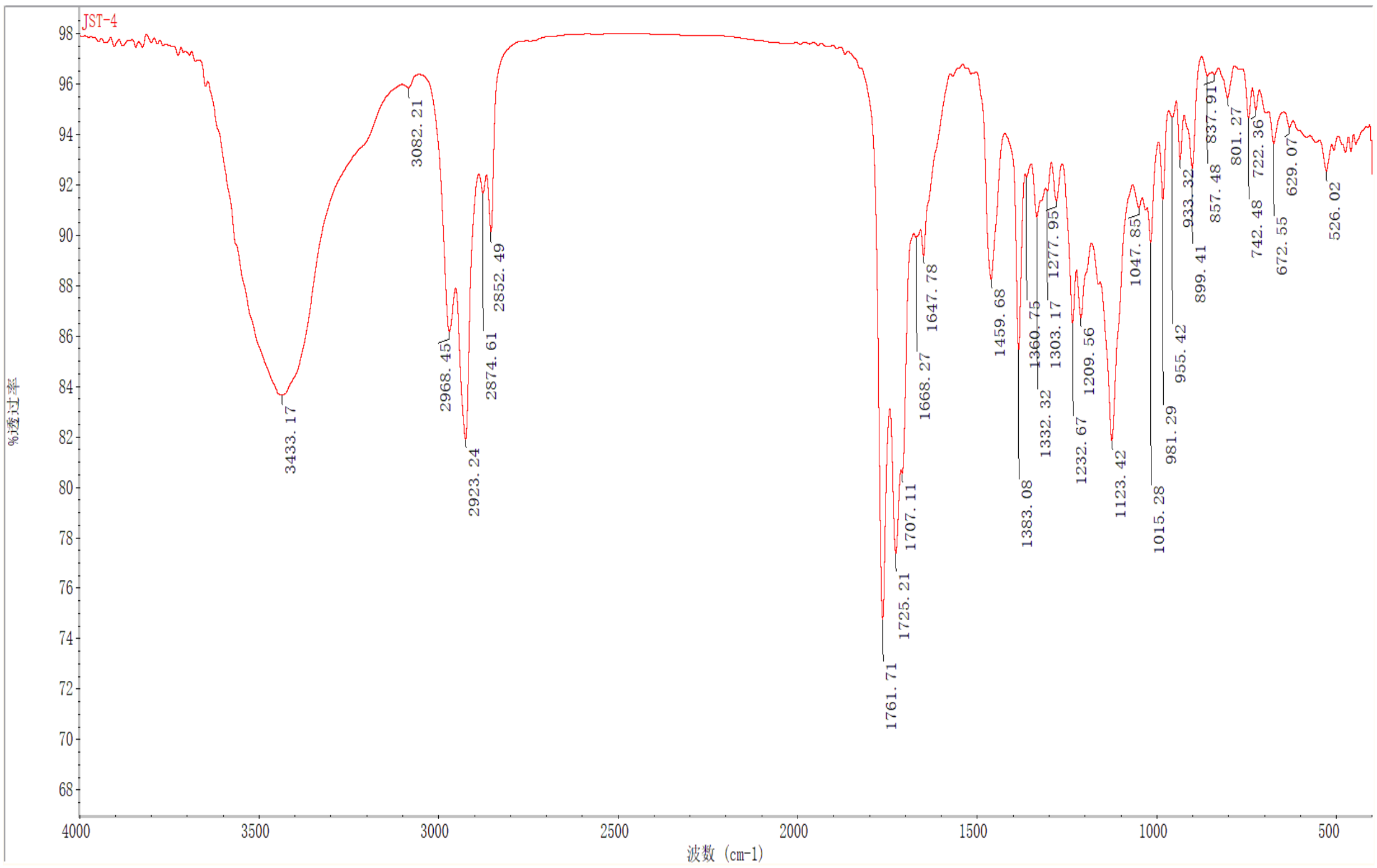

Figure S42. IR spectrum of compound **3**.

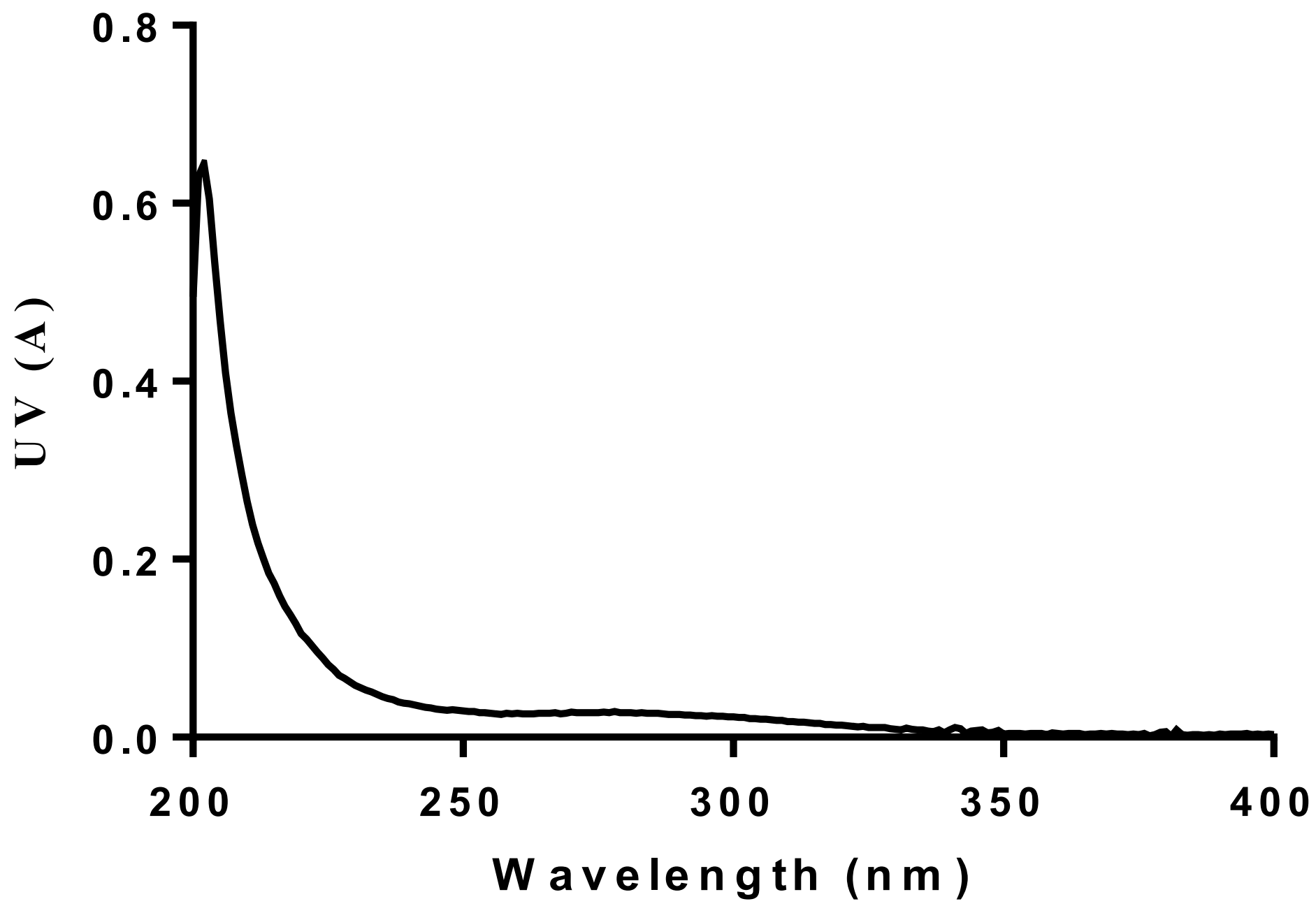

Figure S43. UV spectrum of compound **3**.

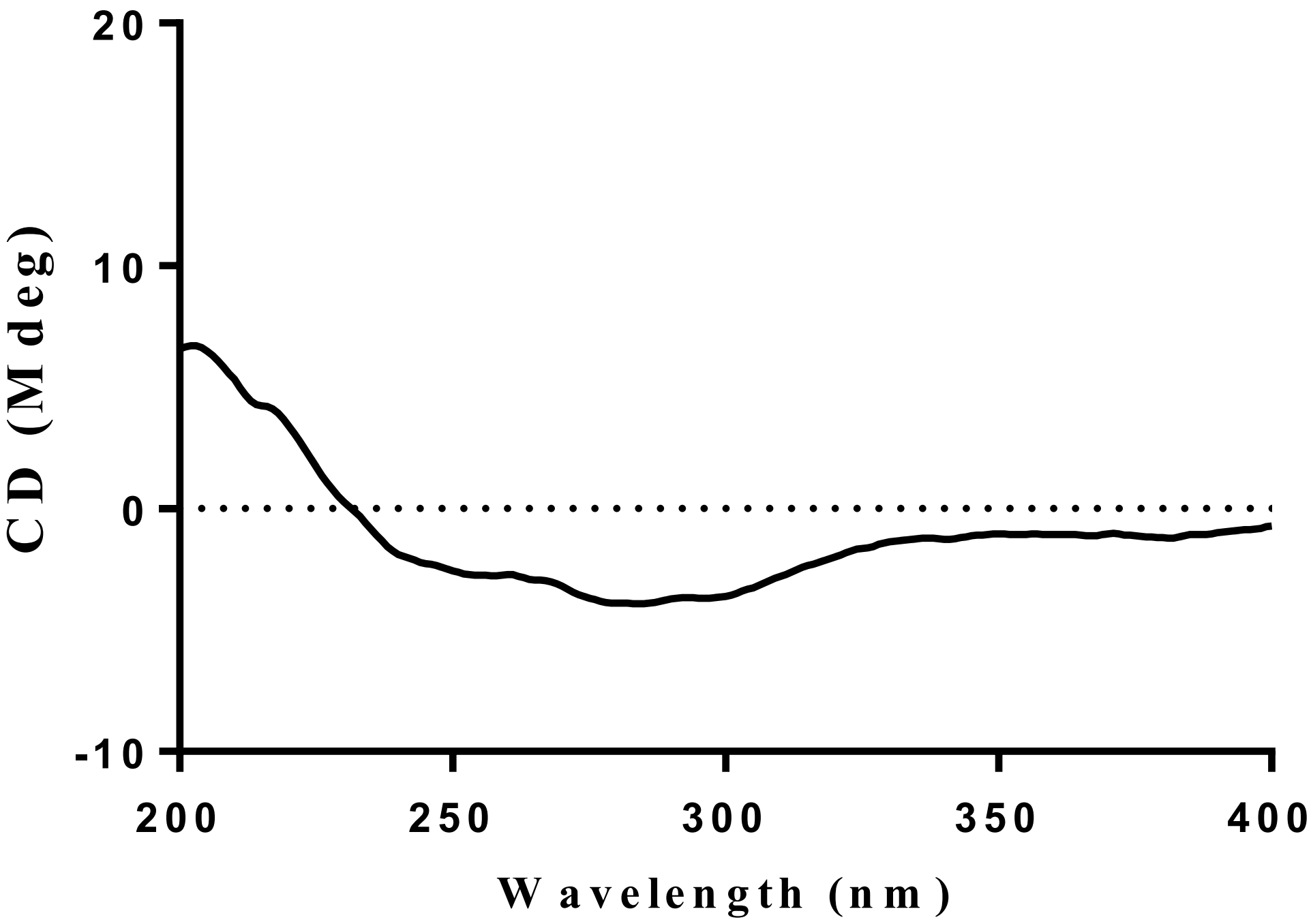

Figure S44. CD spectrum of **3** in MeOH.

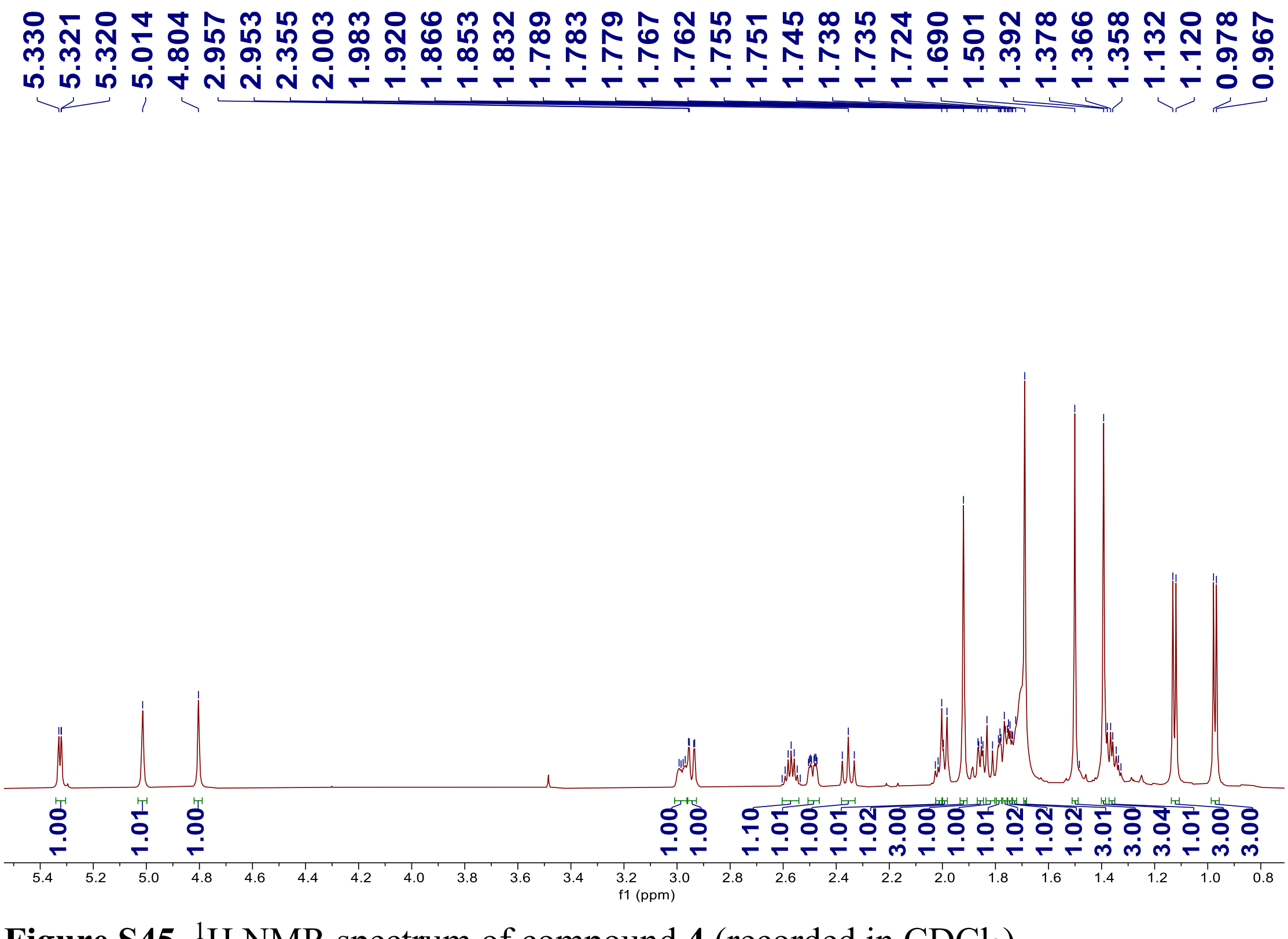

Figure S45. ^{1}H NMR spectrum of compound **4** (recorded in $CDCl_3$).

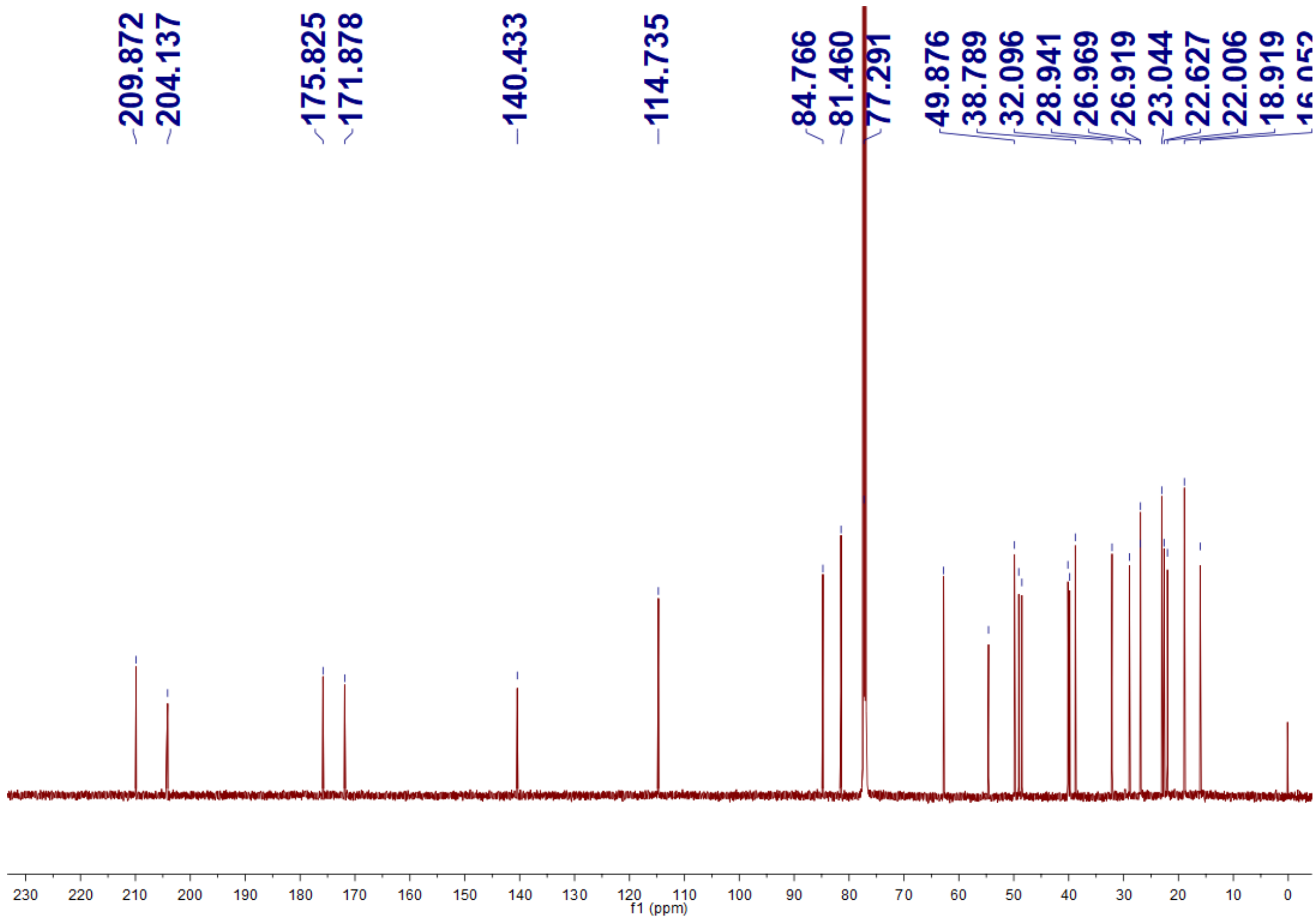

Figure S46. ^{13}C NMR spectrum of compound **4** (recorded in $CDCl_3$).

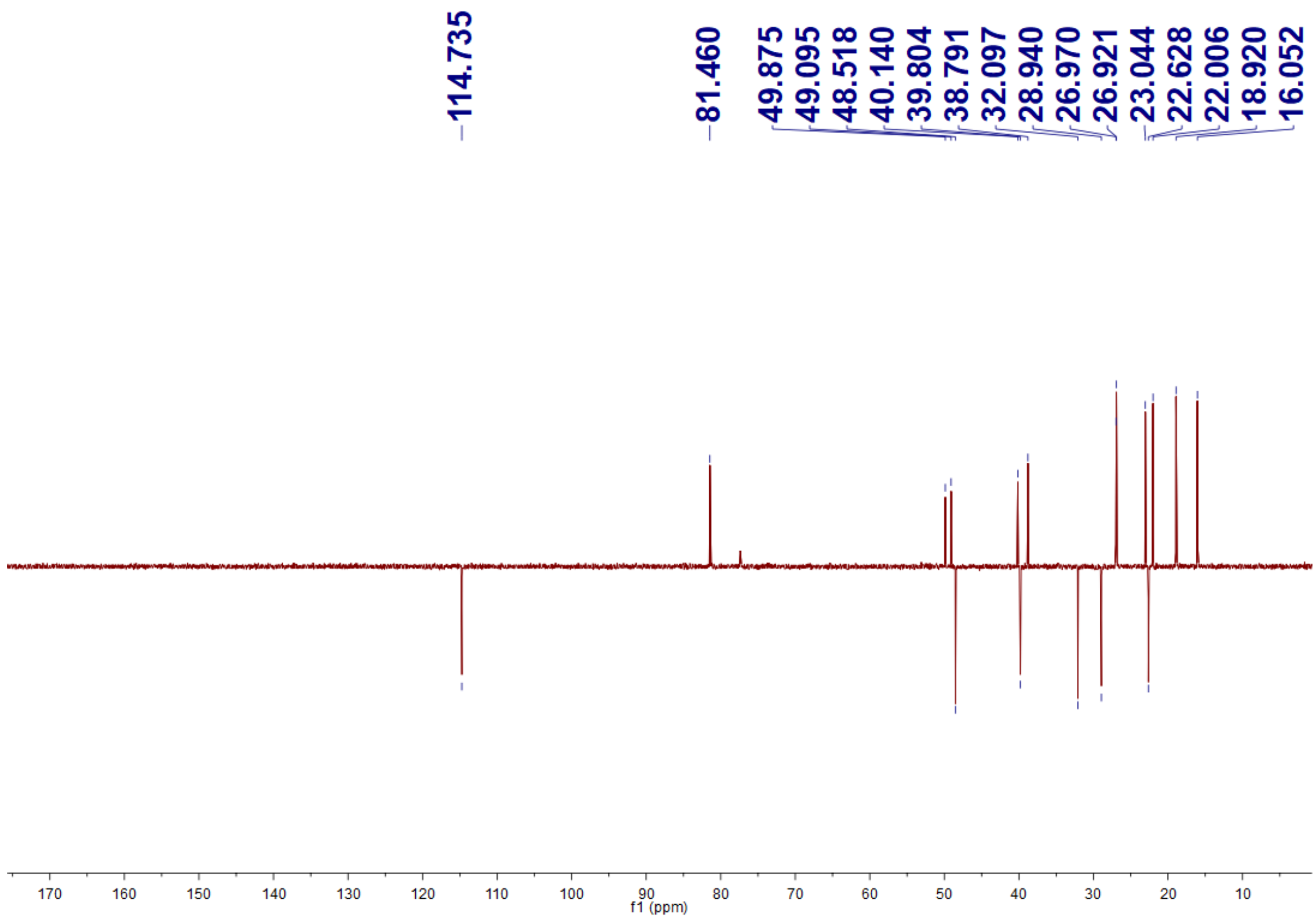

Figure S47. DEPT spectrum of compound **4** (recorded in $CDCl_3$).

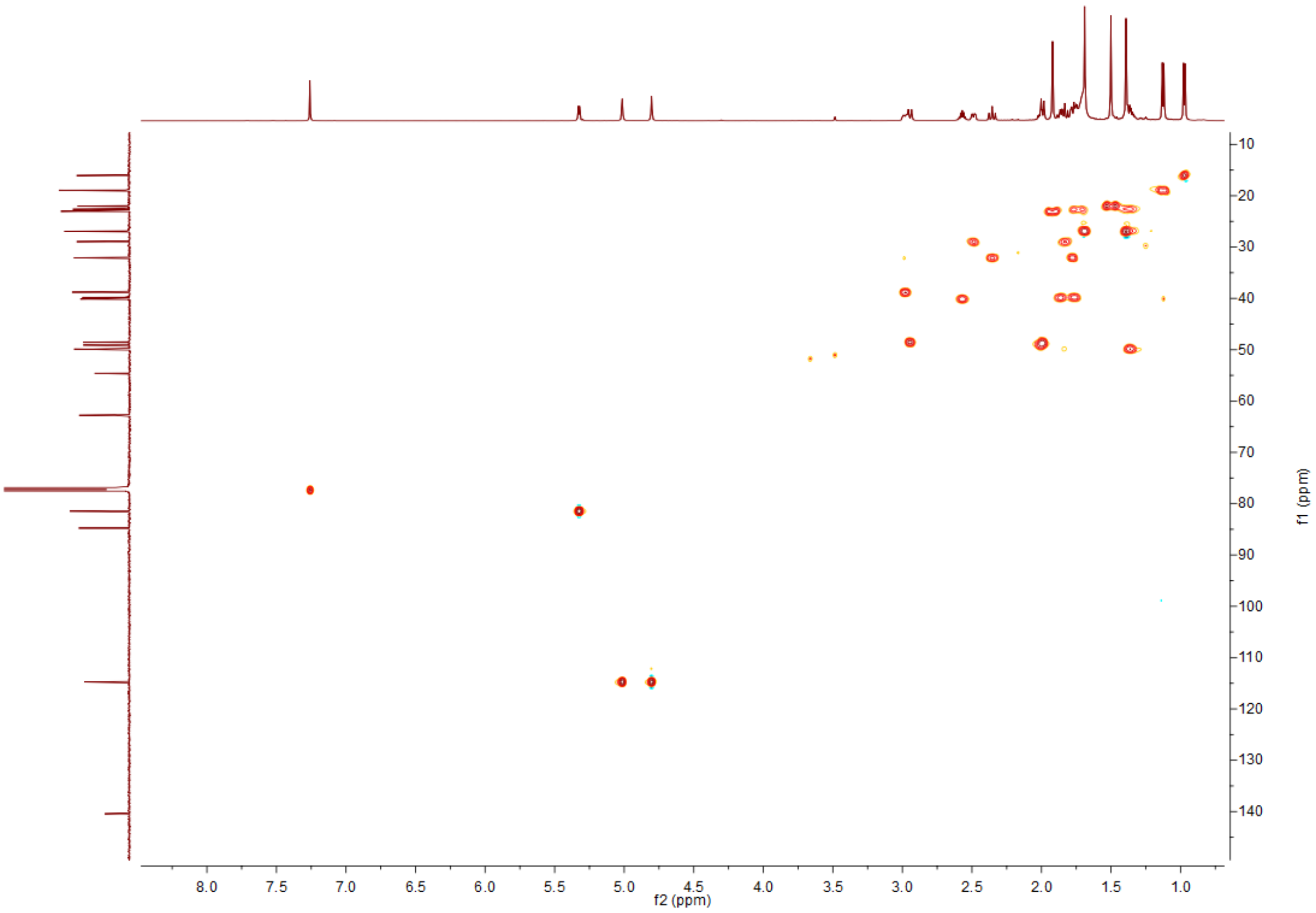

Figure S48. HSQC spectrum of compound **4** (recorded in $CDCl_3$).

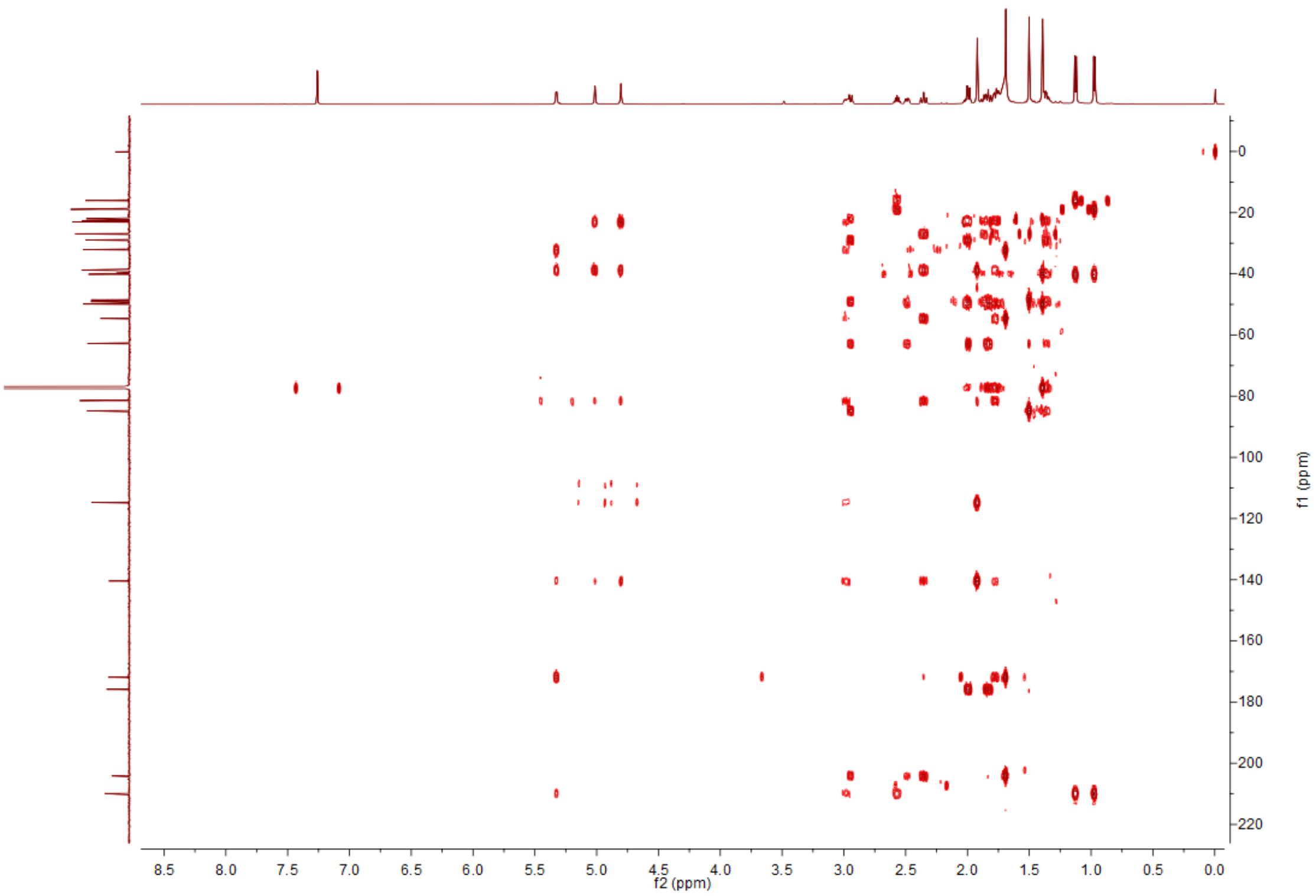

Figure S49. HMBC spectrum of compound **4** (recorded in $CDCl_3$).

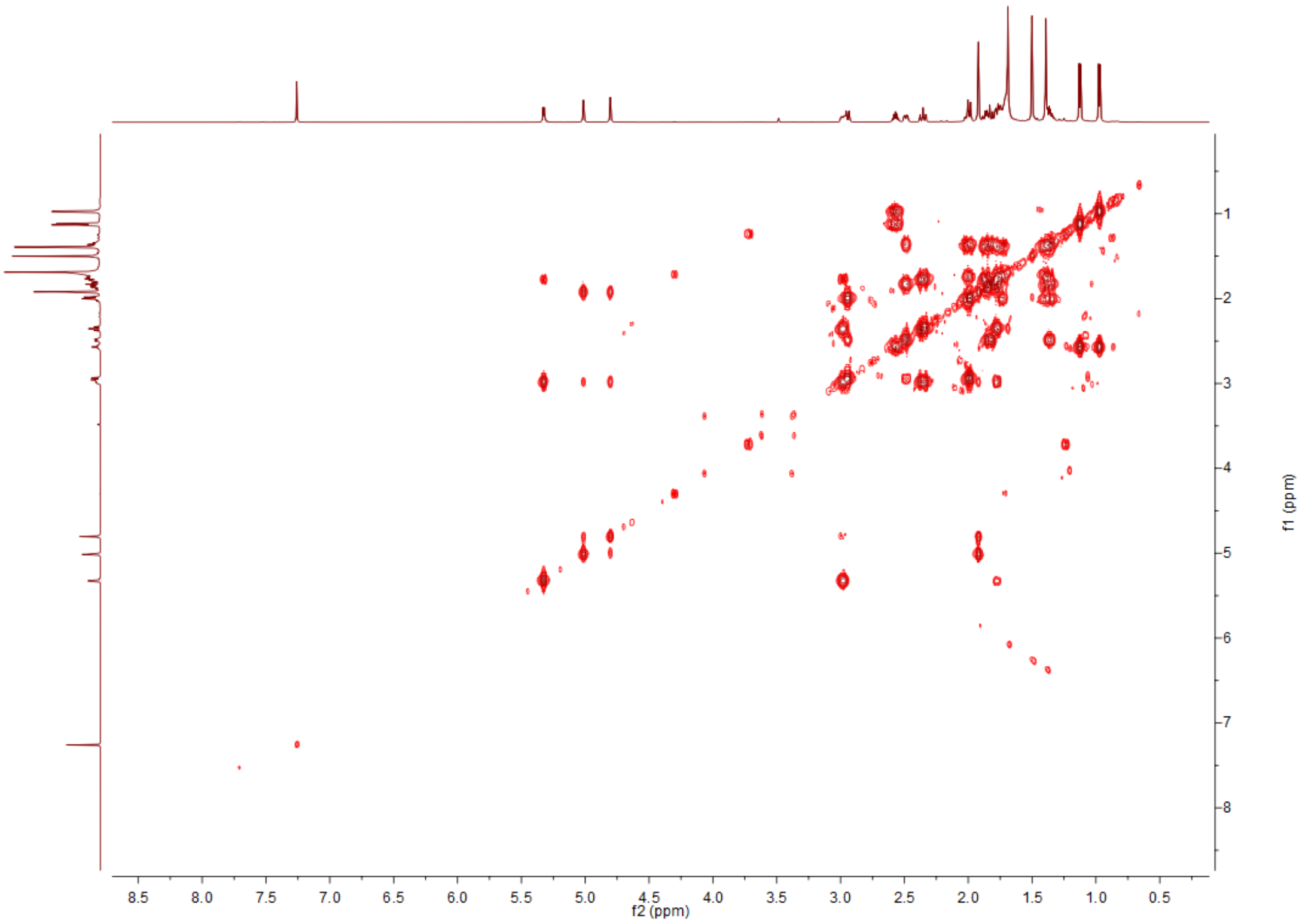

Figure S50. ^{1}H–^{1}H COSY spectrum of compound **4** (recorded in $CDCl_3$).

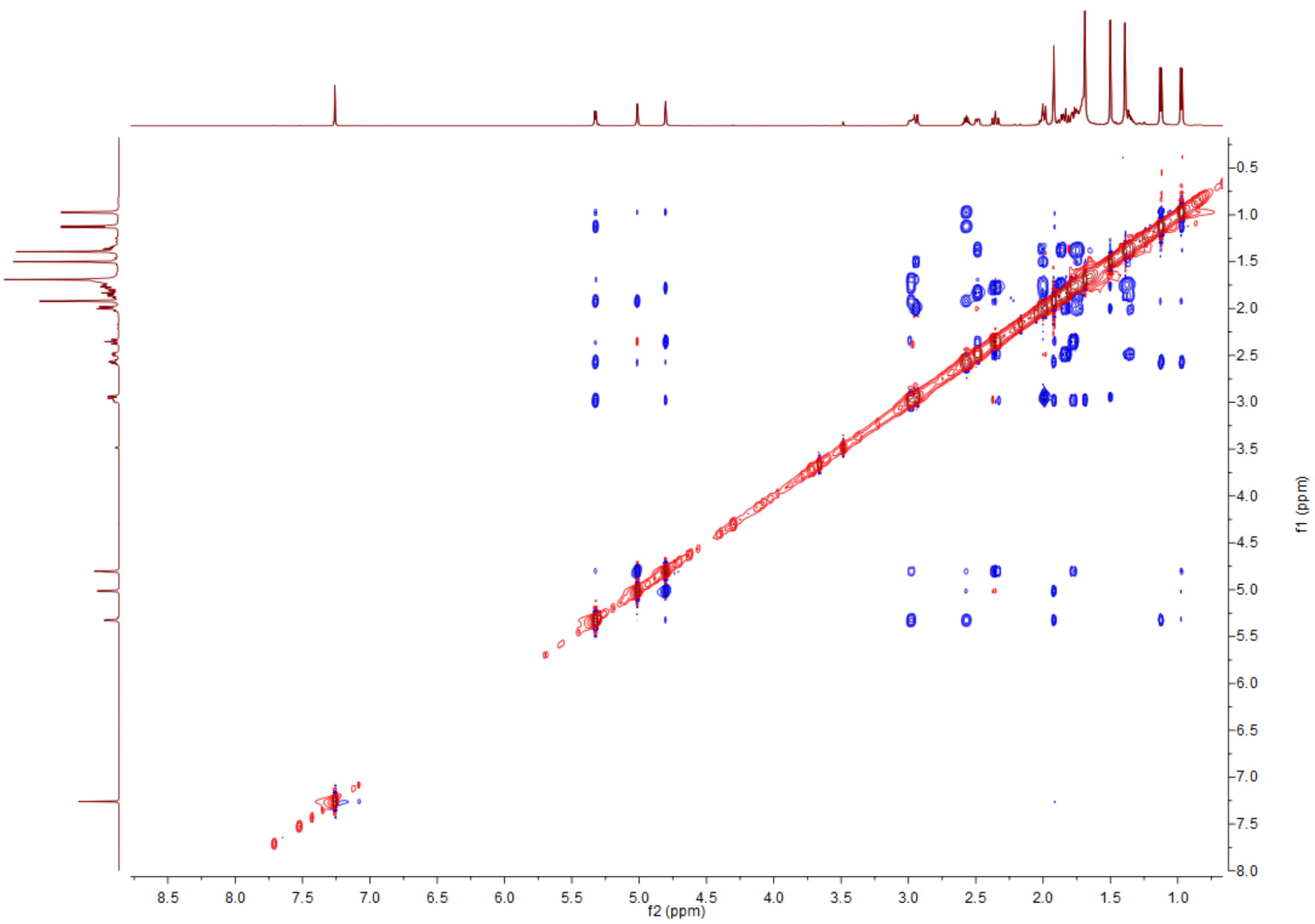

Figure S51. ROESY spectrum of compound **4** (recorded in $CDCl_3$).

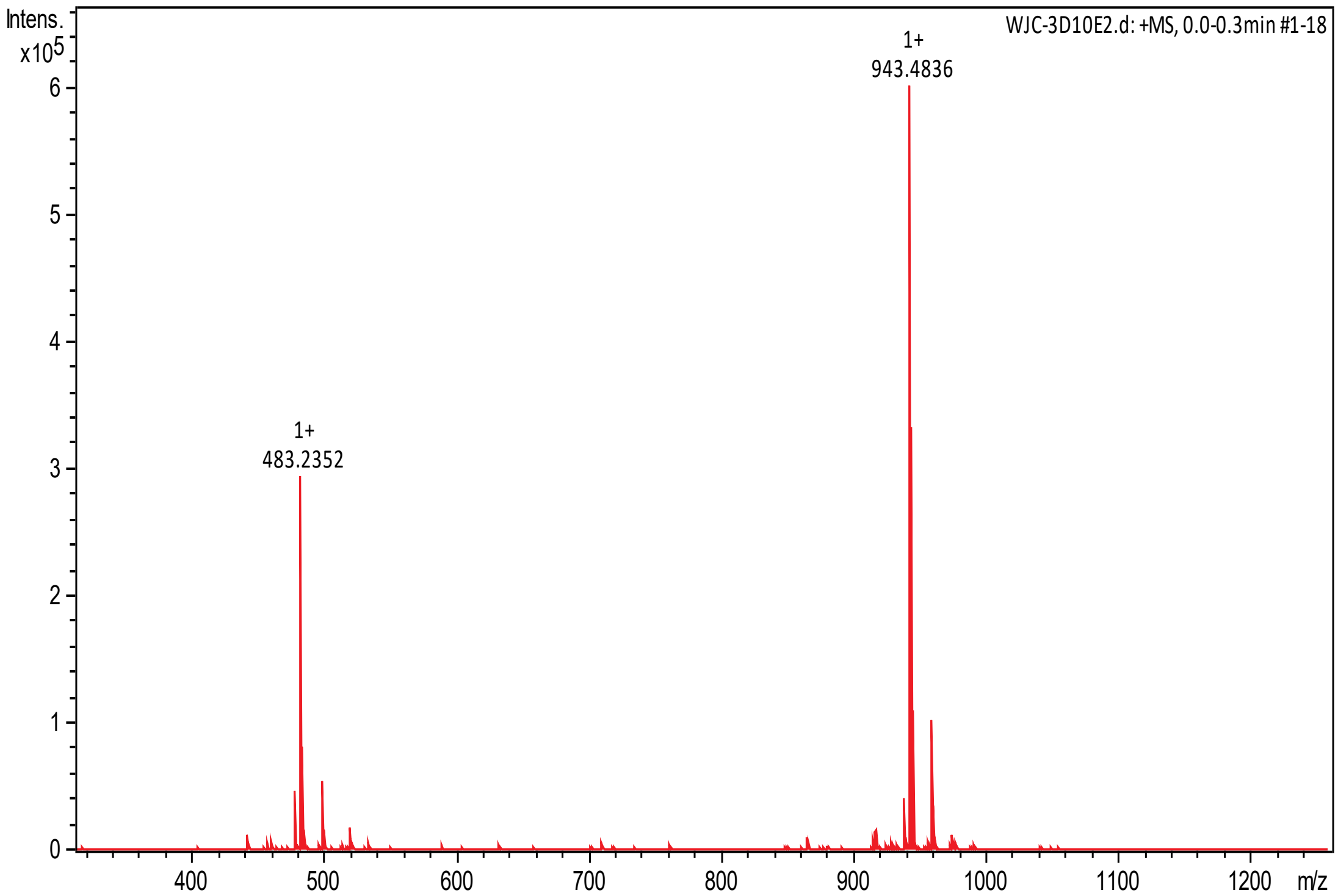

Figure S52. HRESIMS spectrum of compound **4**.

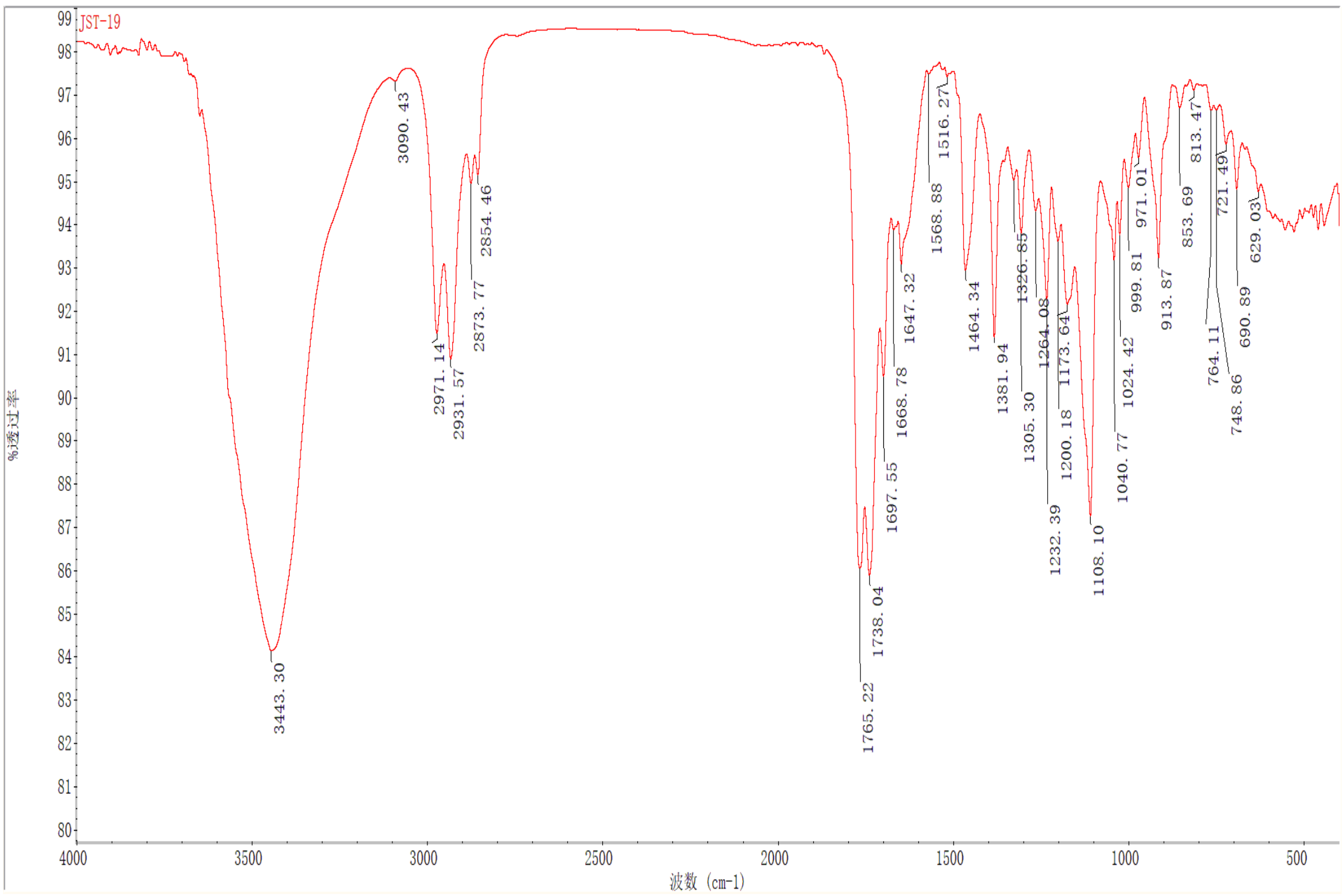

Figure S53. IR spectrum of compound **4**.

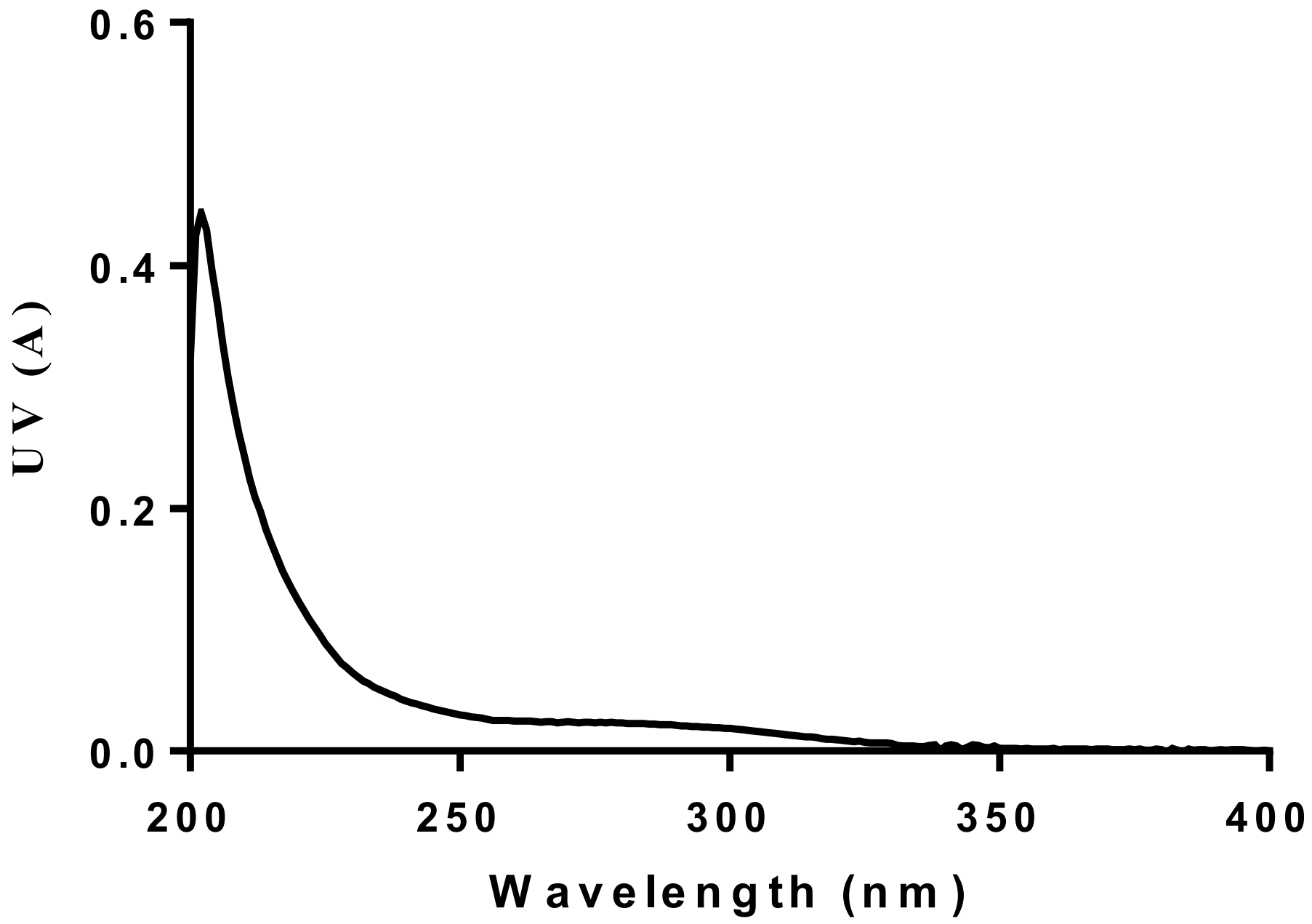

Figure S54. UV spectrum of compound **4**.

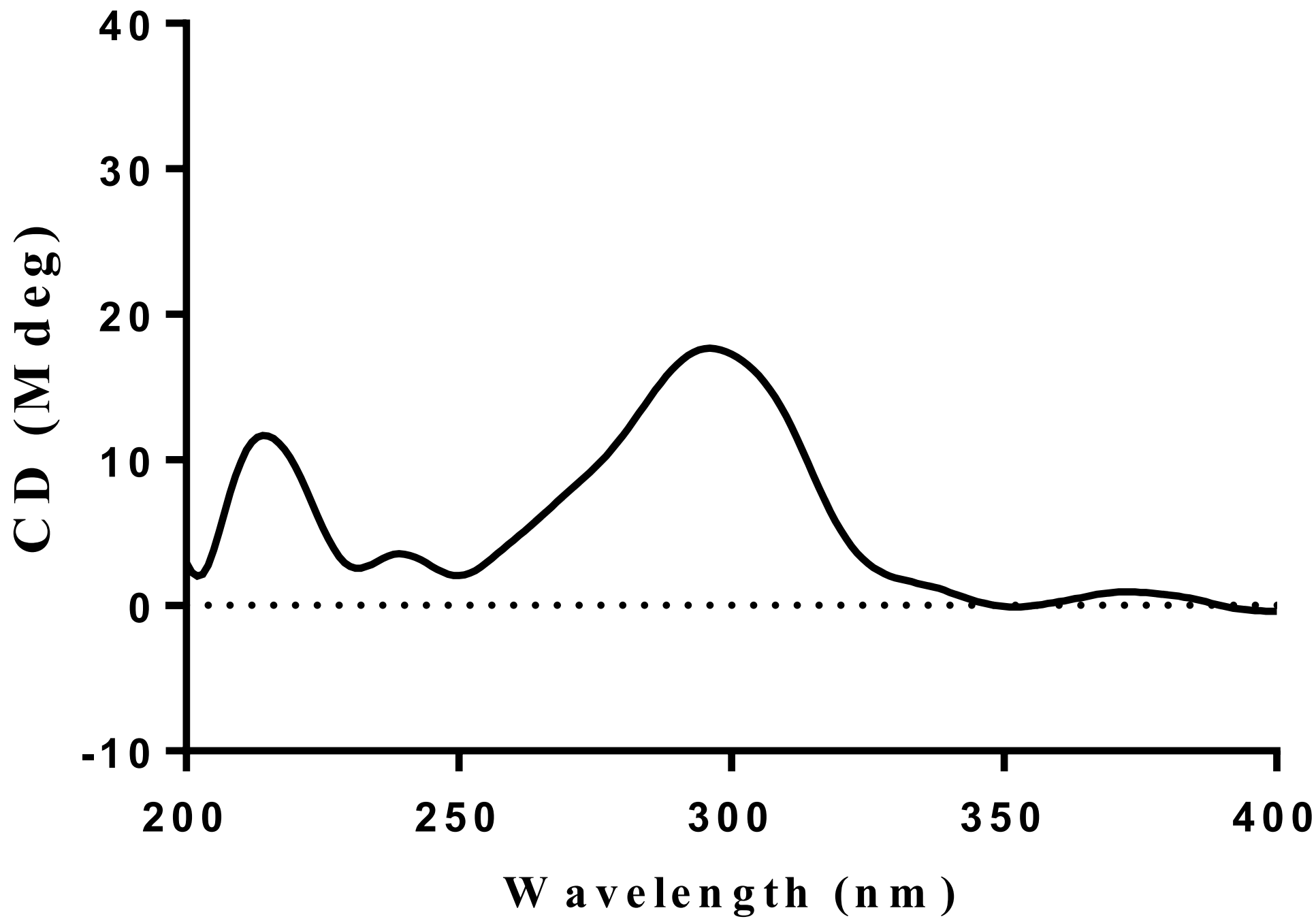

Figure S55. CD spectrum of **4** in MeOH.

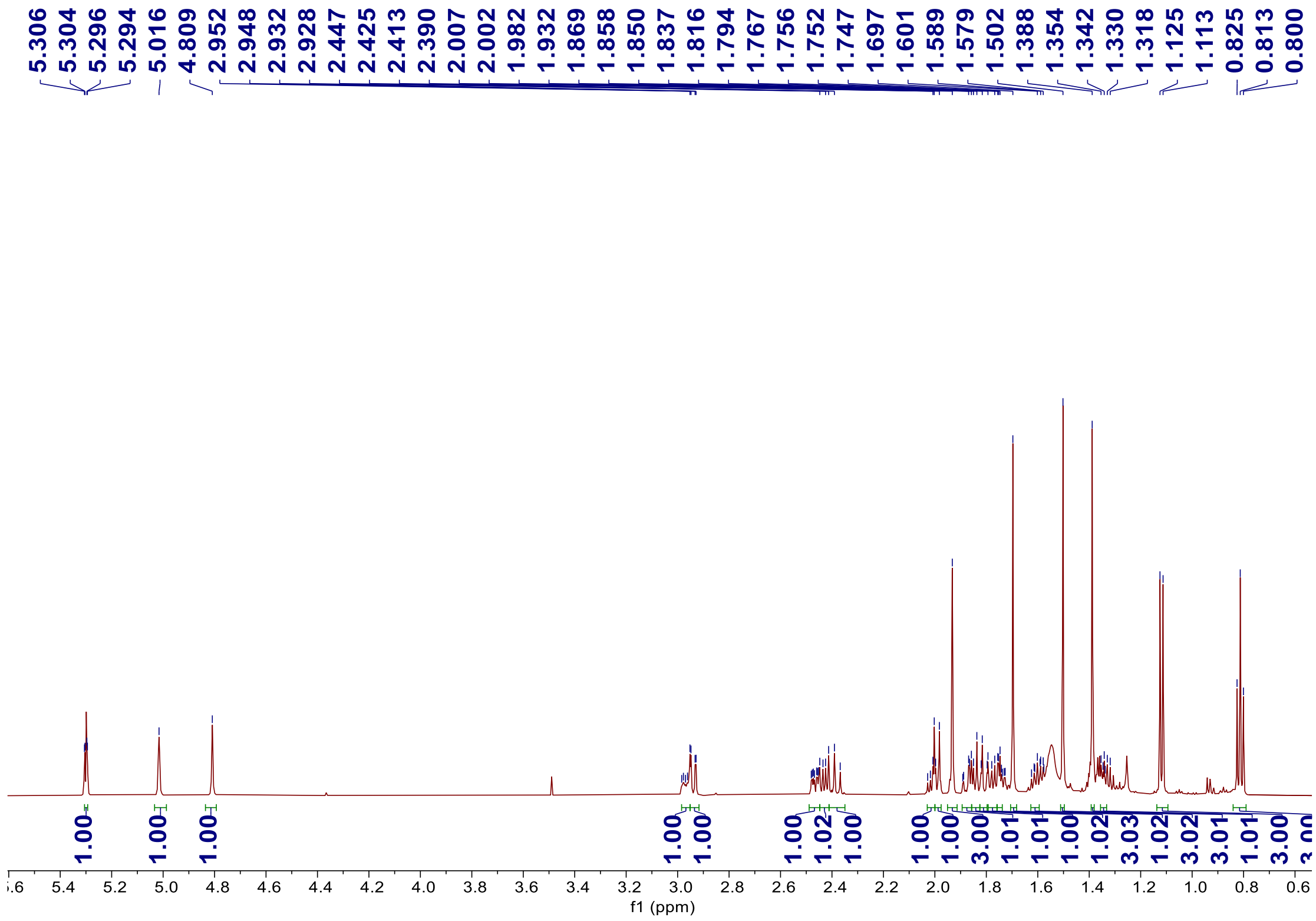

Figure S56. ^{1}H NMR spectrum of compound **5** (recorded in $CDCl_3$).

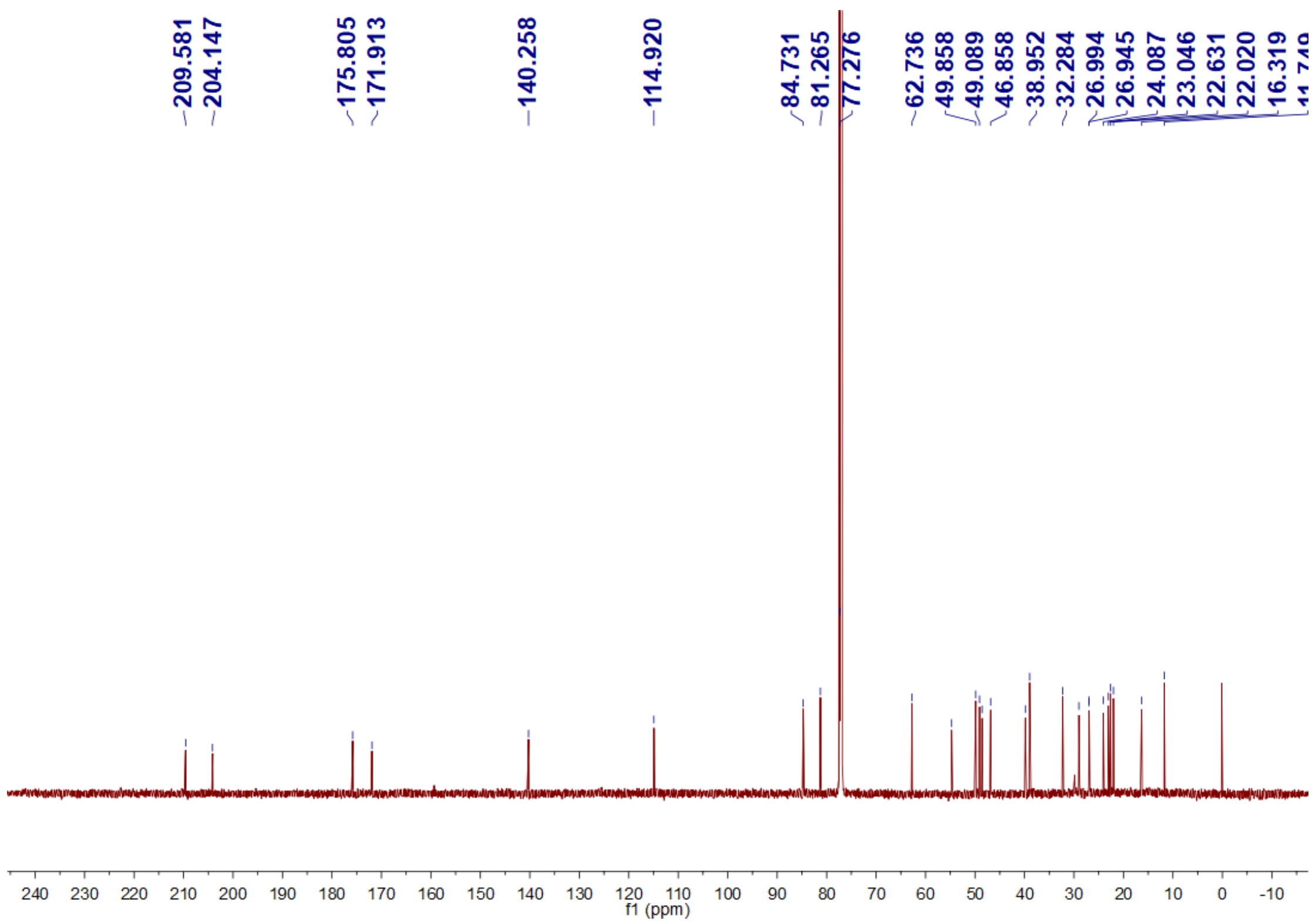

Figure S57. ^{13}C NMR spectrum of compound **5** (recorded in $CDCl_3$).

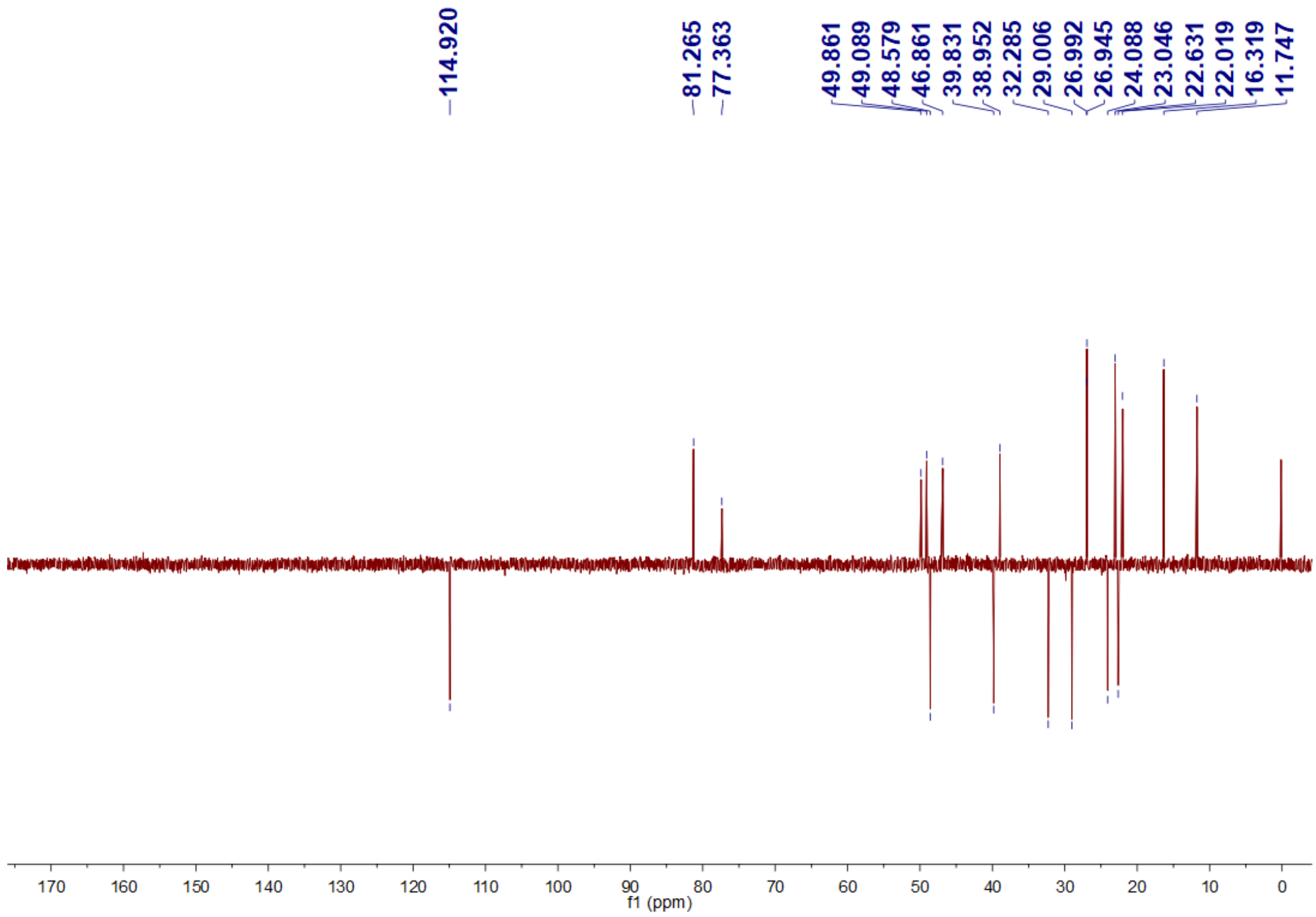

Figure S58. DEPT spectrum of compound **5** (recorded in $CDCl_3$).

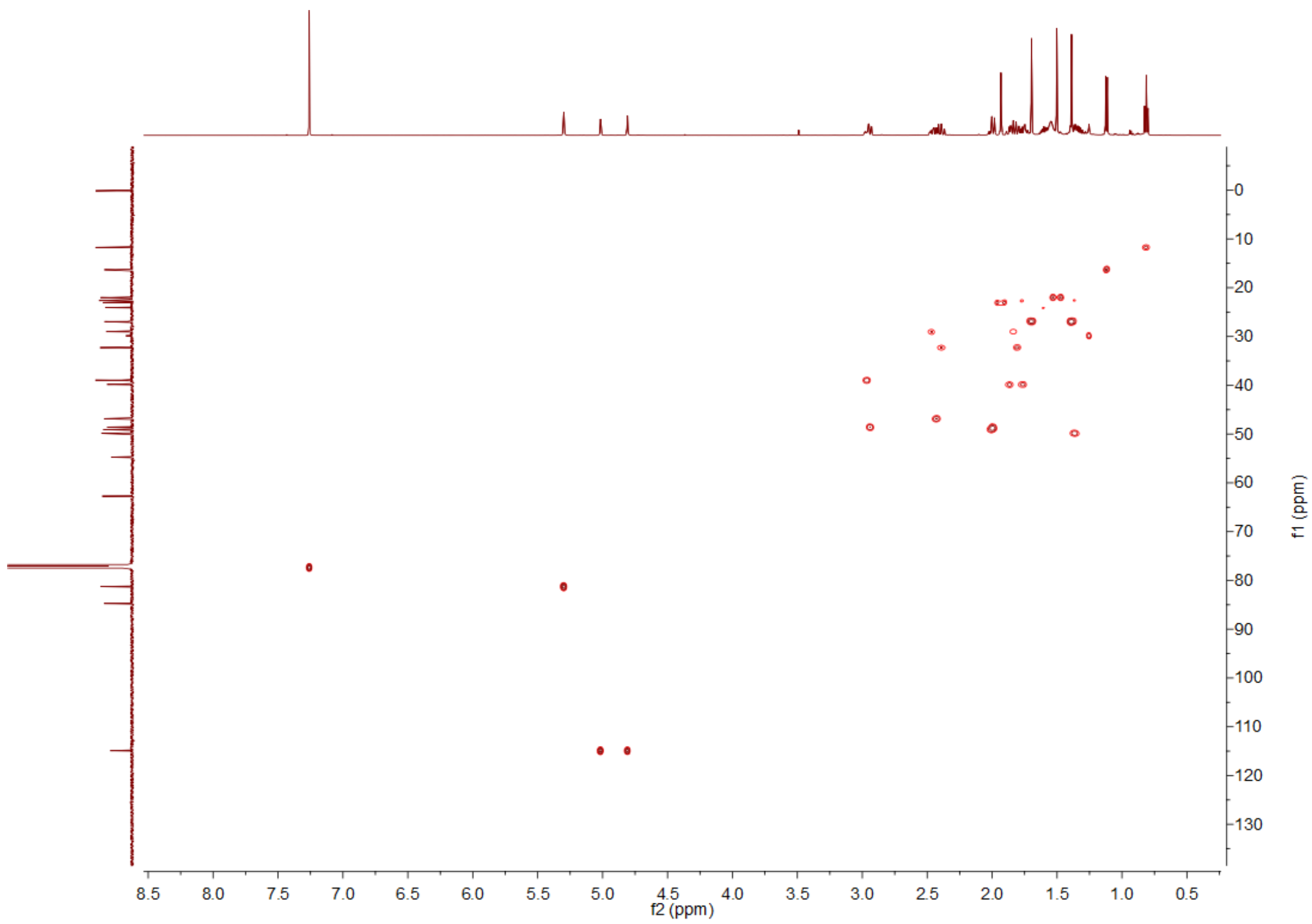

Figure S59. HSQC spectrum of compound **5** (recorded in $CDCl_3$).

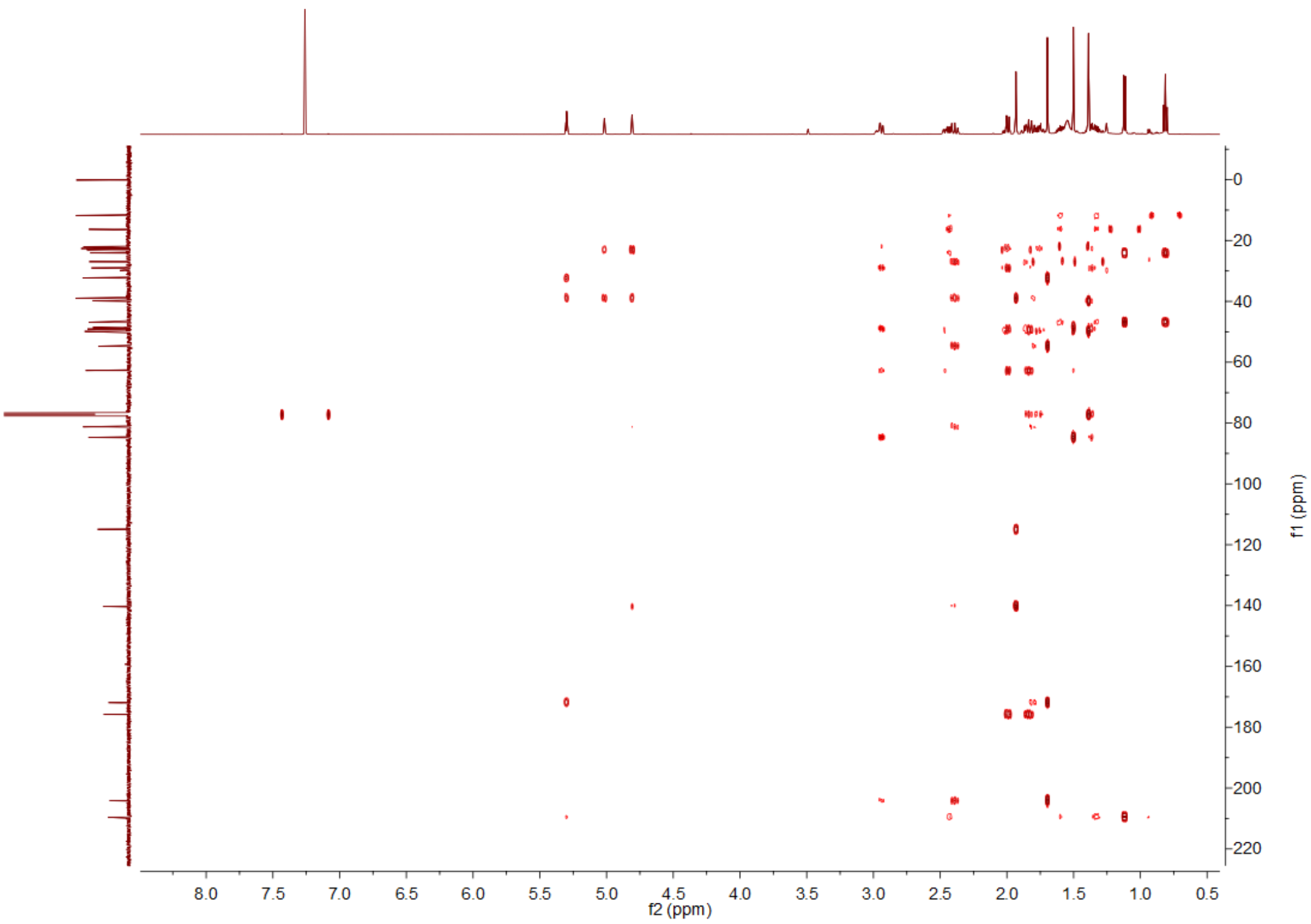

Figure S60. HMBC spectrum of compound **5** (recorded in $CDCl_3$).

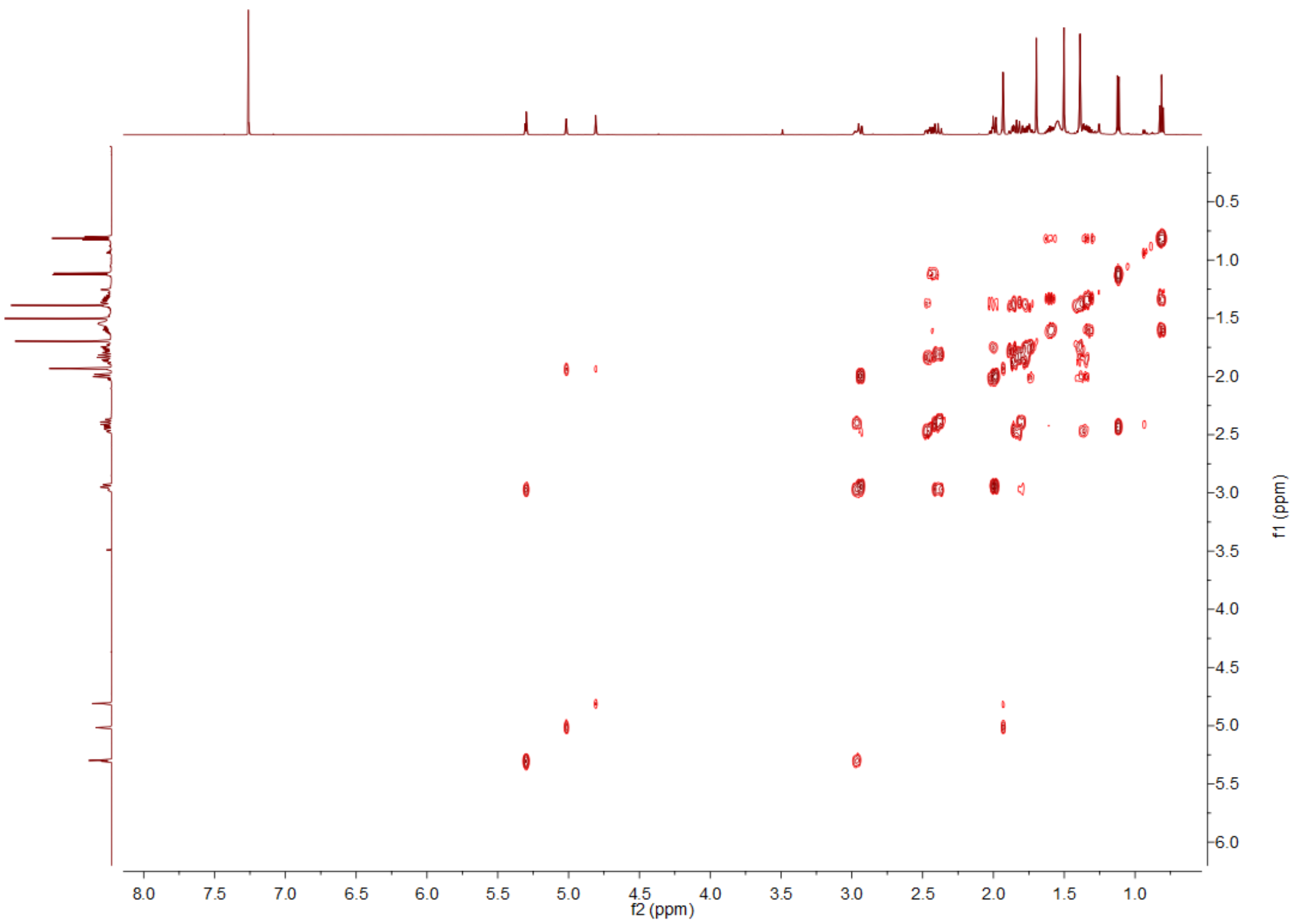

Figure S61. $^{1}H-^{1}H$ COSY spectrum of compound **5** (recorded in $CDCl_3$).

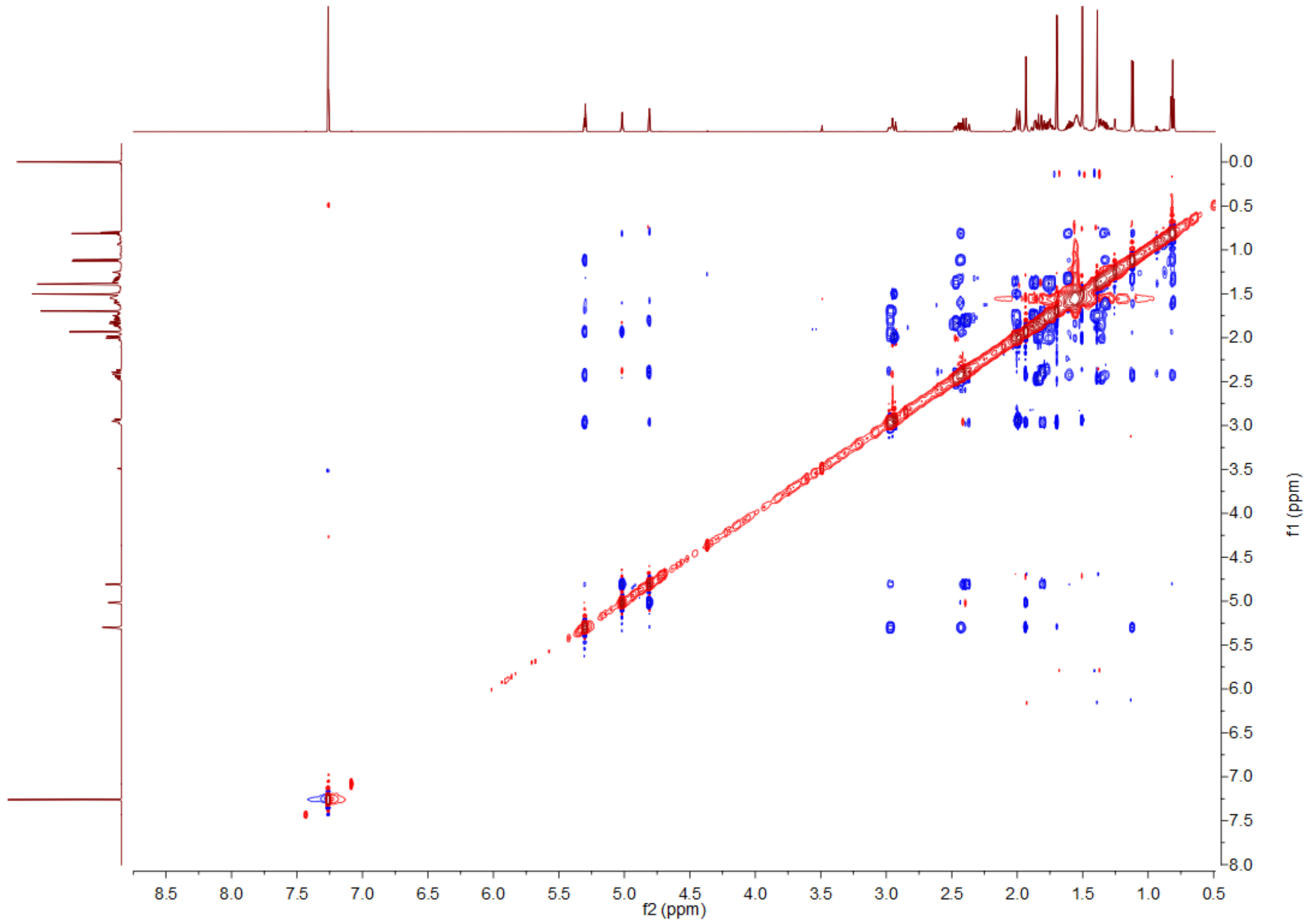

Figure S62. ROESY spectrum of compound **5** (recorded in $CDCl_3$).

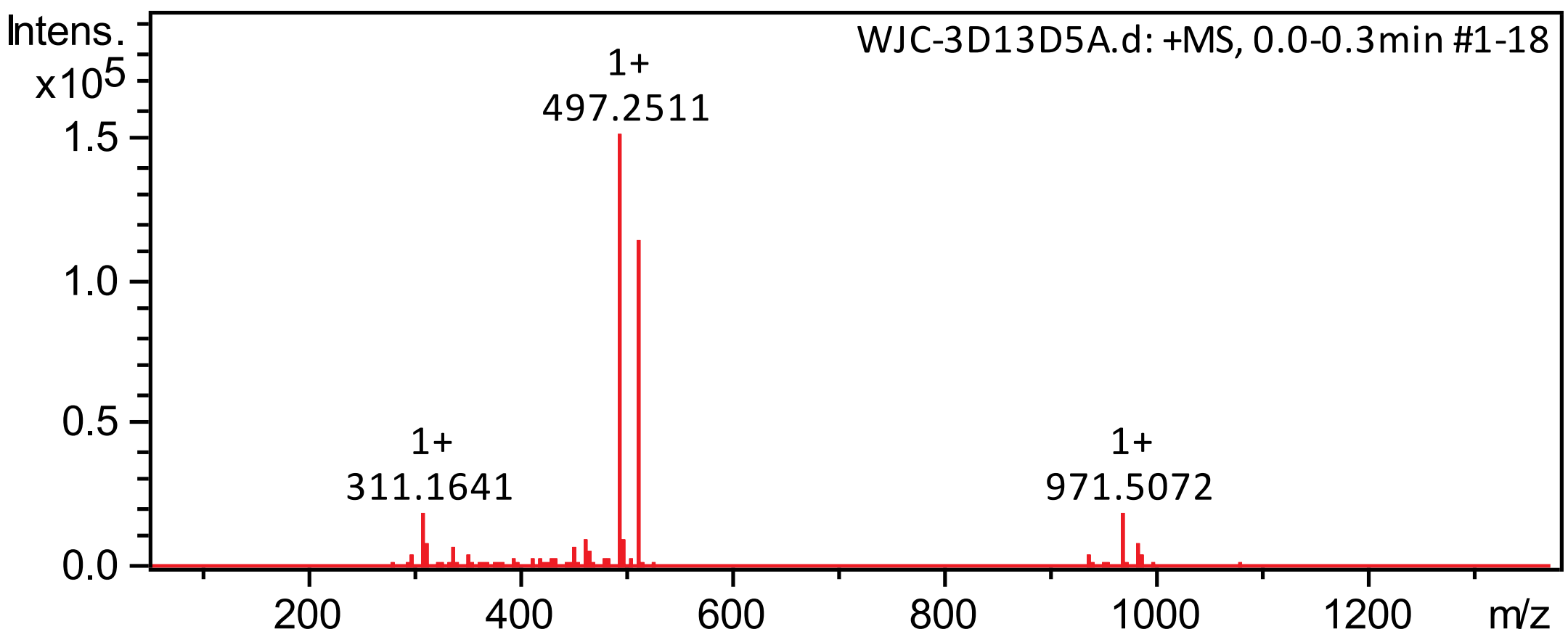

Figure S63. HRESIMS spectrum of compound **5**.

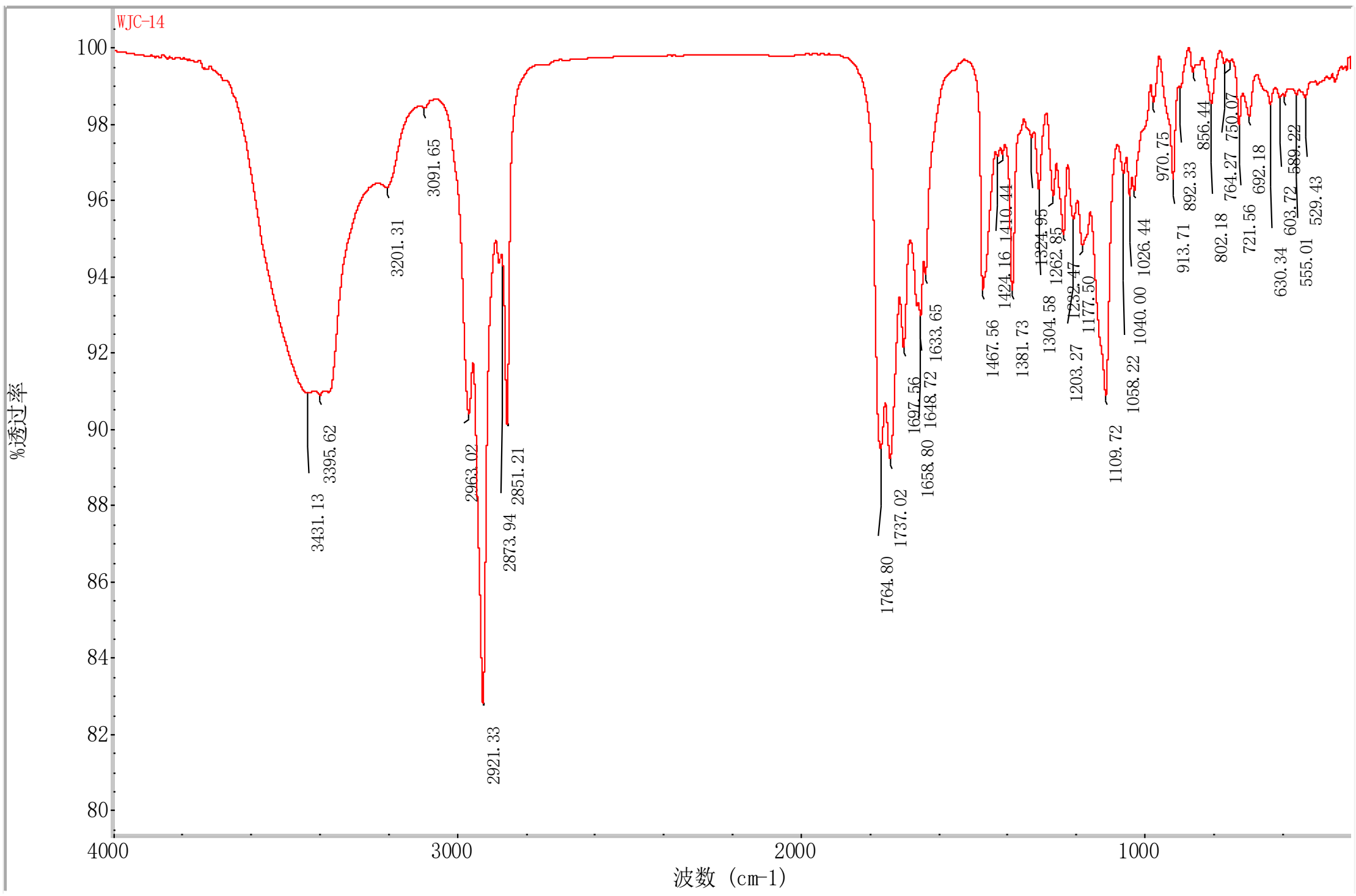

Figure S64. IR spectrum of compound **5**.

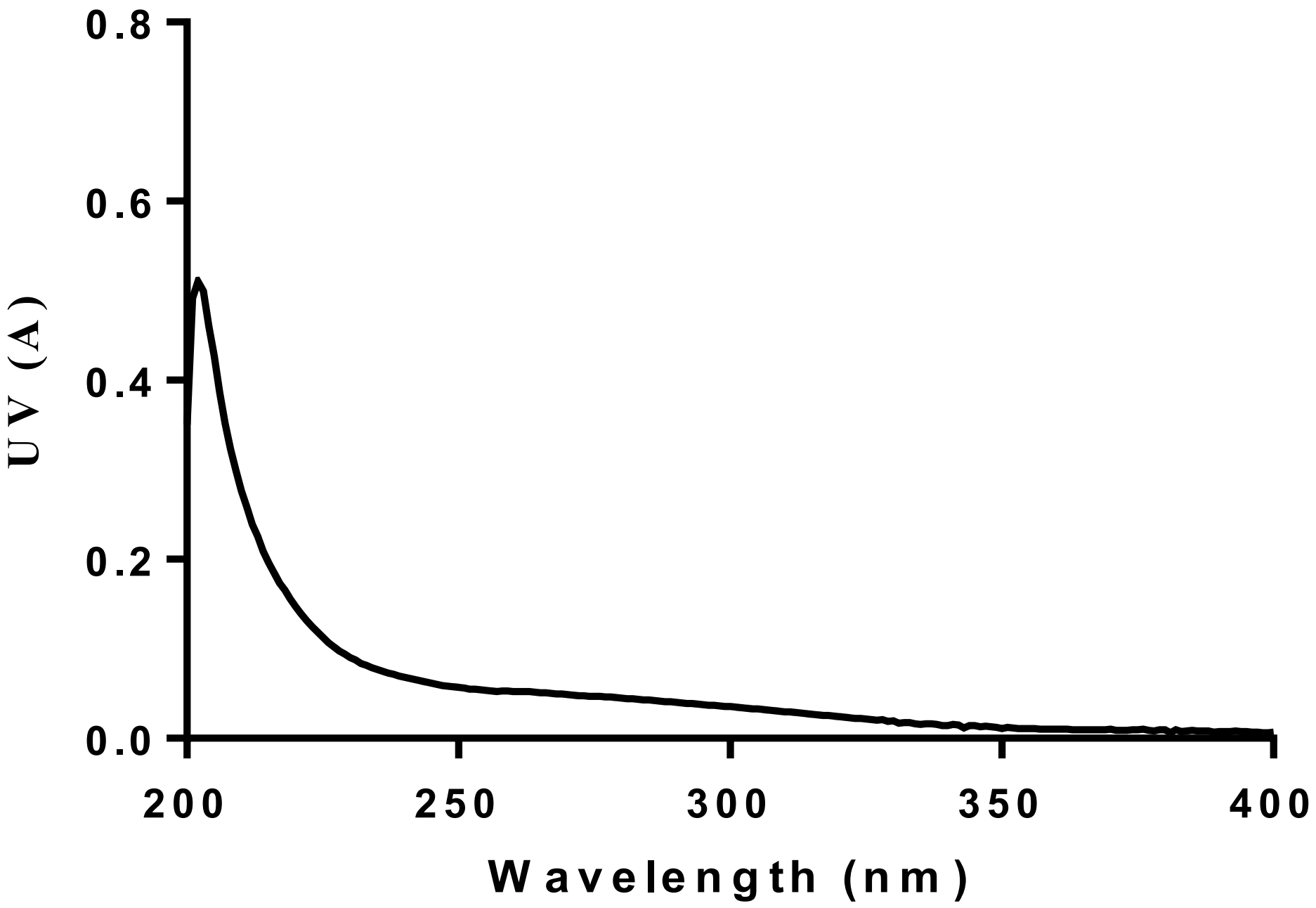

Figure S65. UV spectrum of **5** in MeOH.

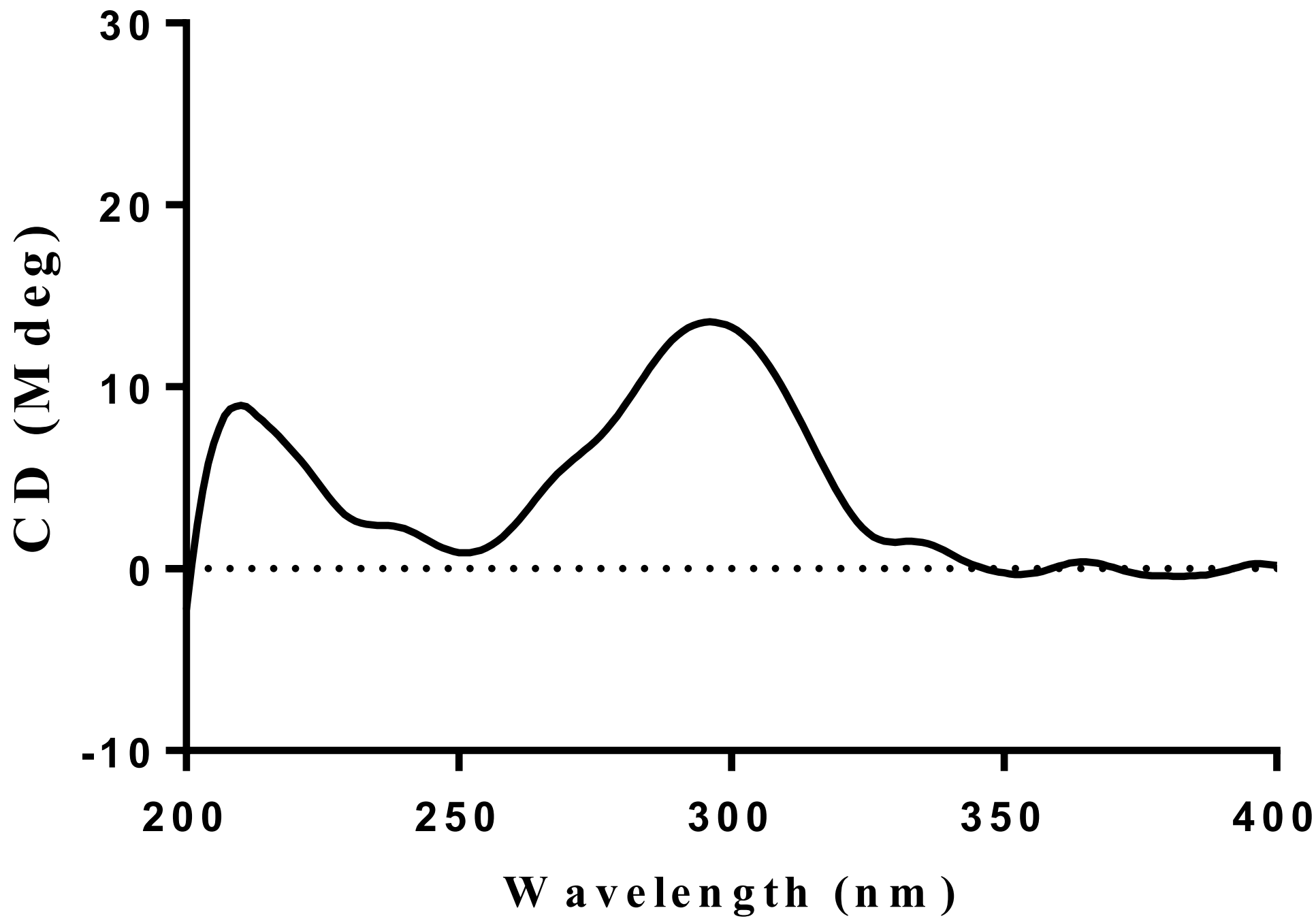

Figure S66. CD spectrum of **5** in MeOH.

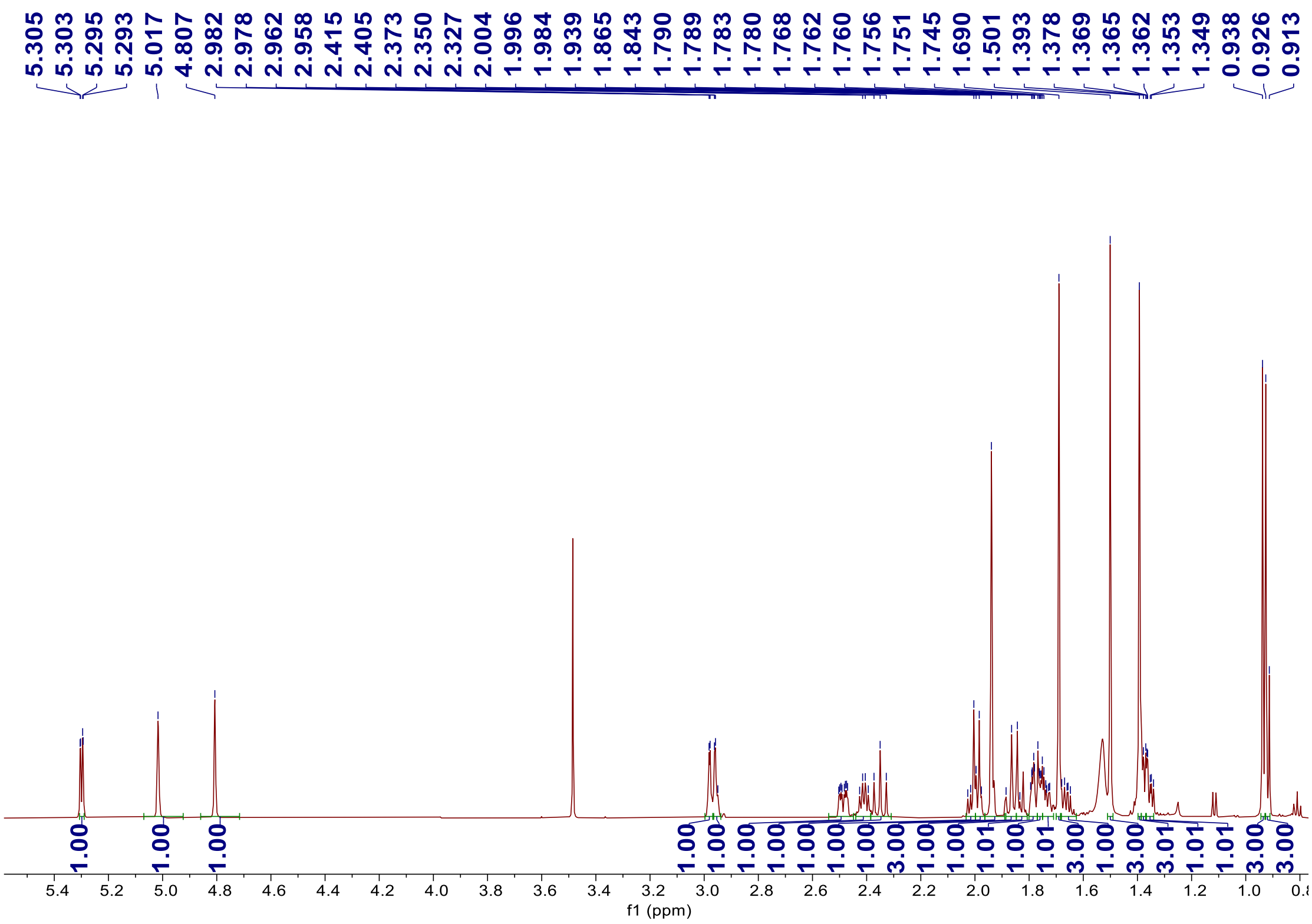

Figure S67. ^{1}H NMR spectrum of compound **6** (recorded in $CDCl_3$).

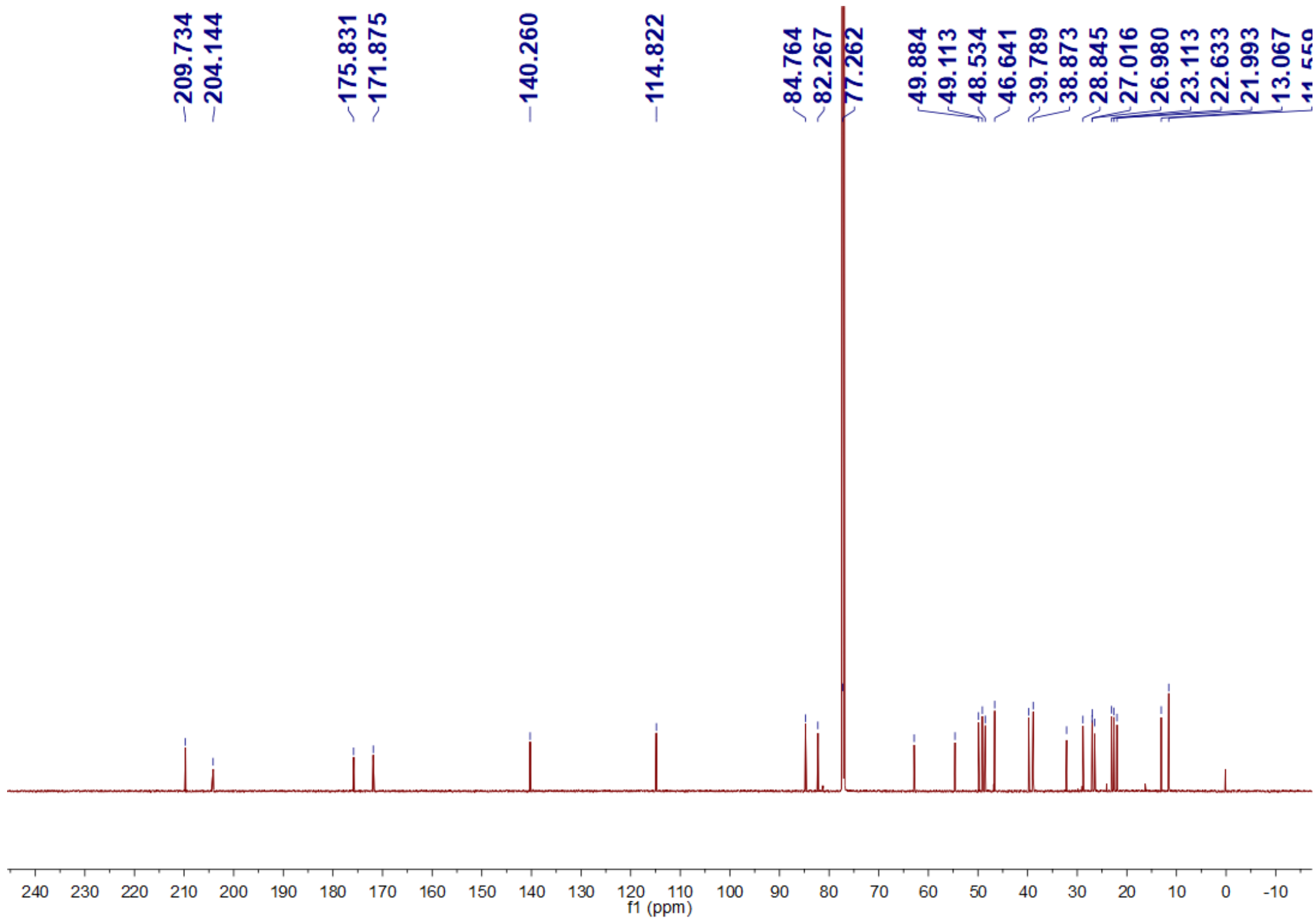

Figure S68. ^{13}C NMR spectrum of compound **6** (recorded in $CDCl_3$).

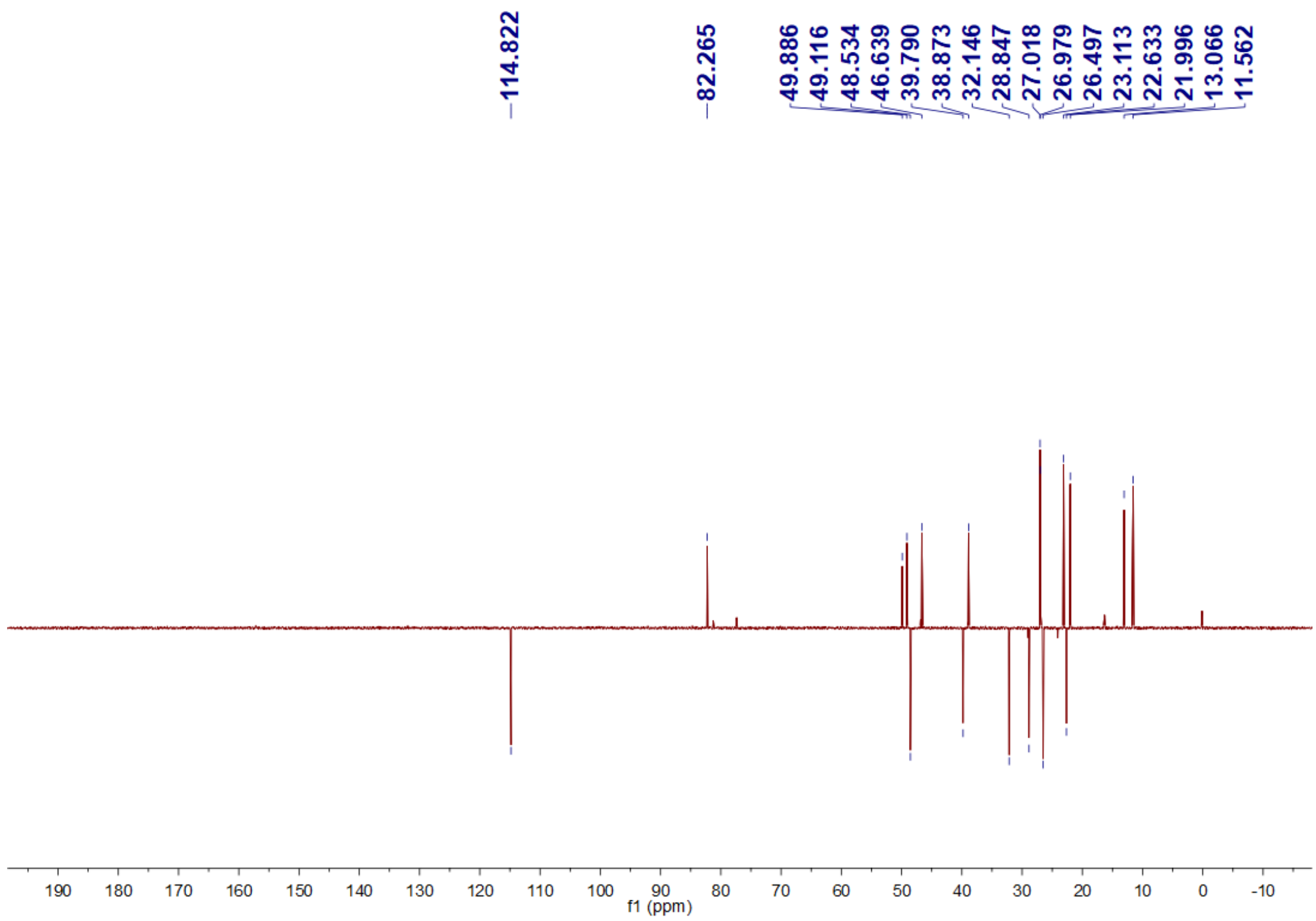

Figure S69. DEPT spectrum of compound **6** (recorded in $CDCl_3$).

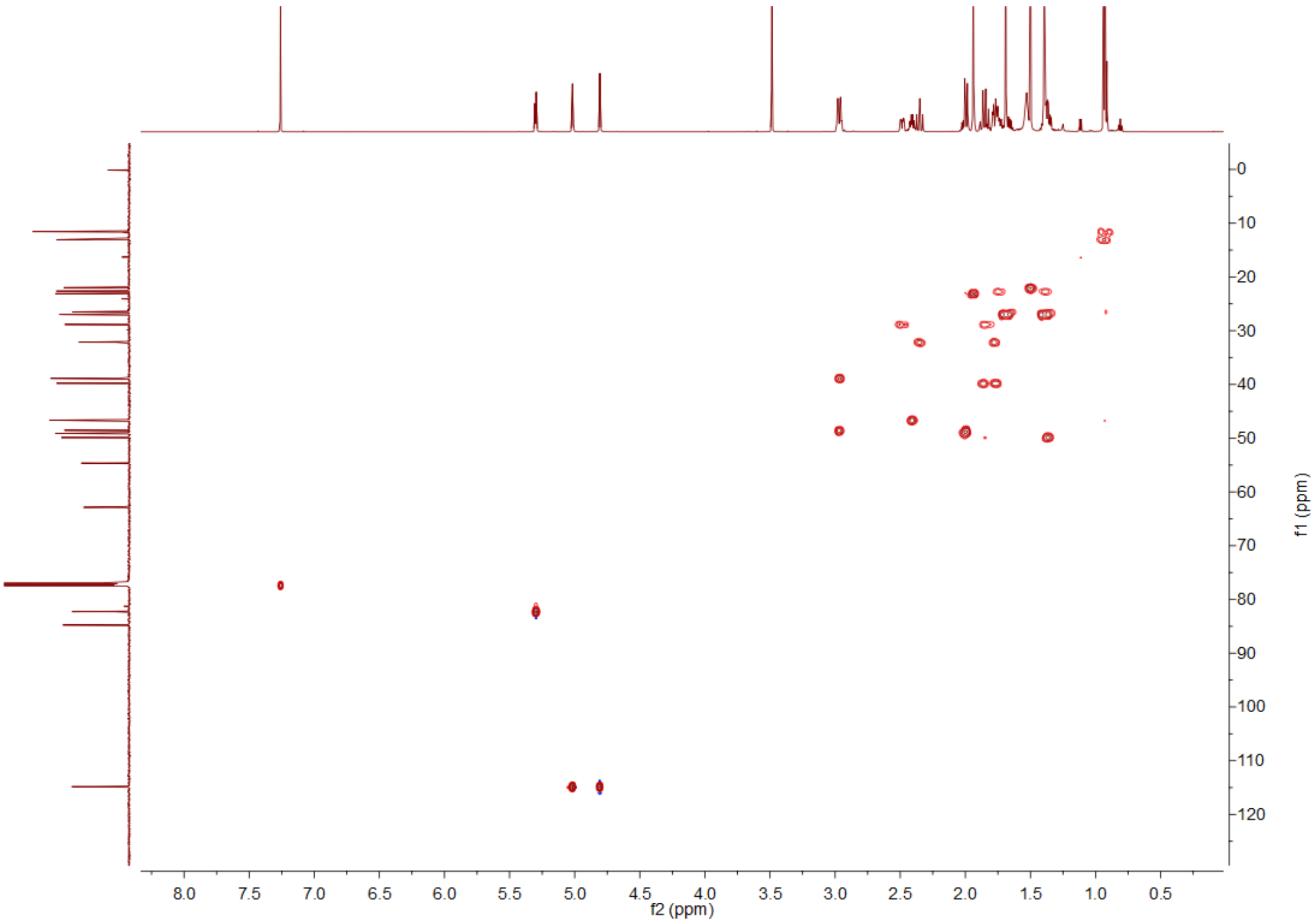

Figure S70. HSQC spectrum of compound **6** (recorded in $CDCl_3$).

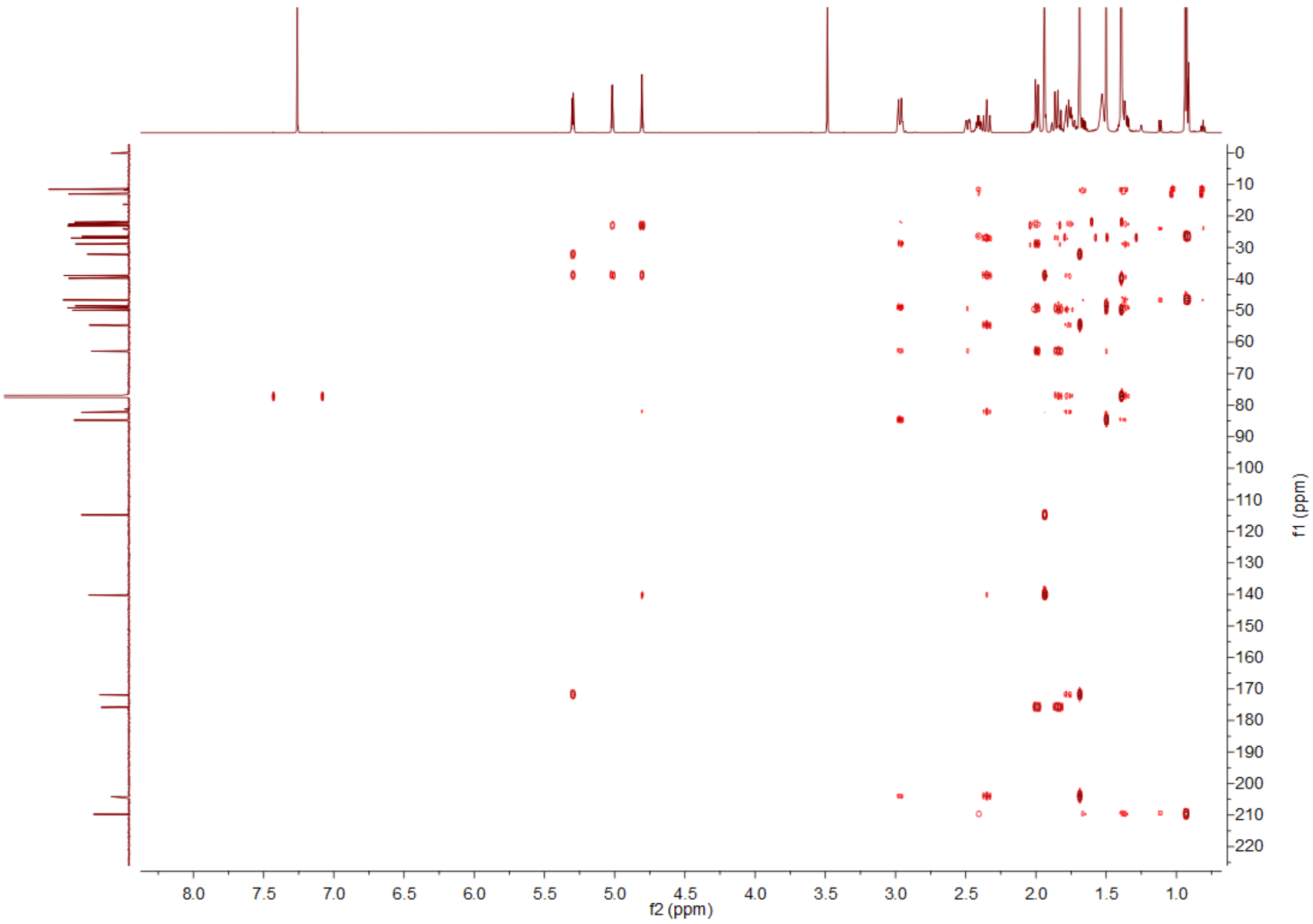

Figure S71. HMBC spectrum of compound **6** (recorded in $CDCl_3$).

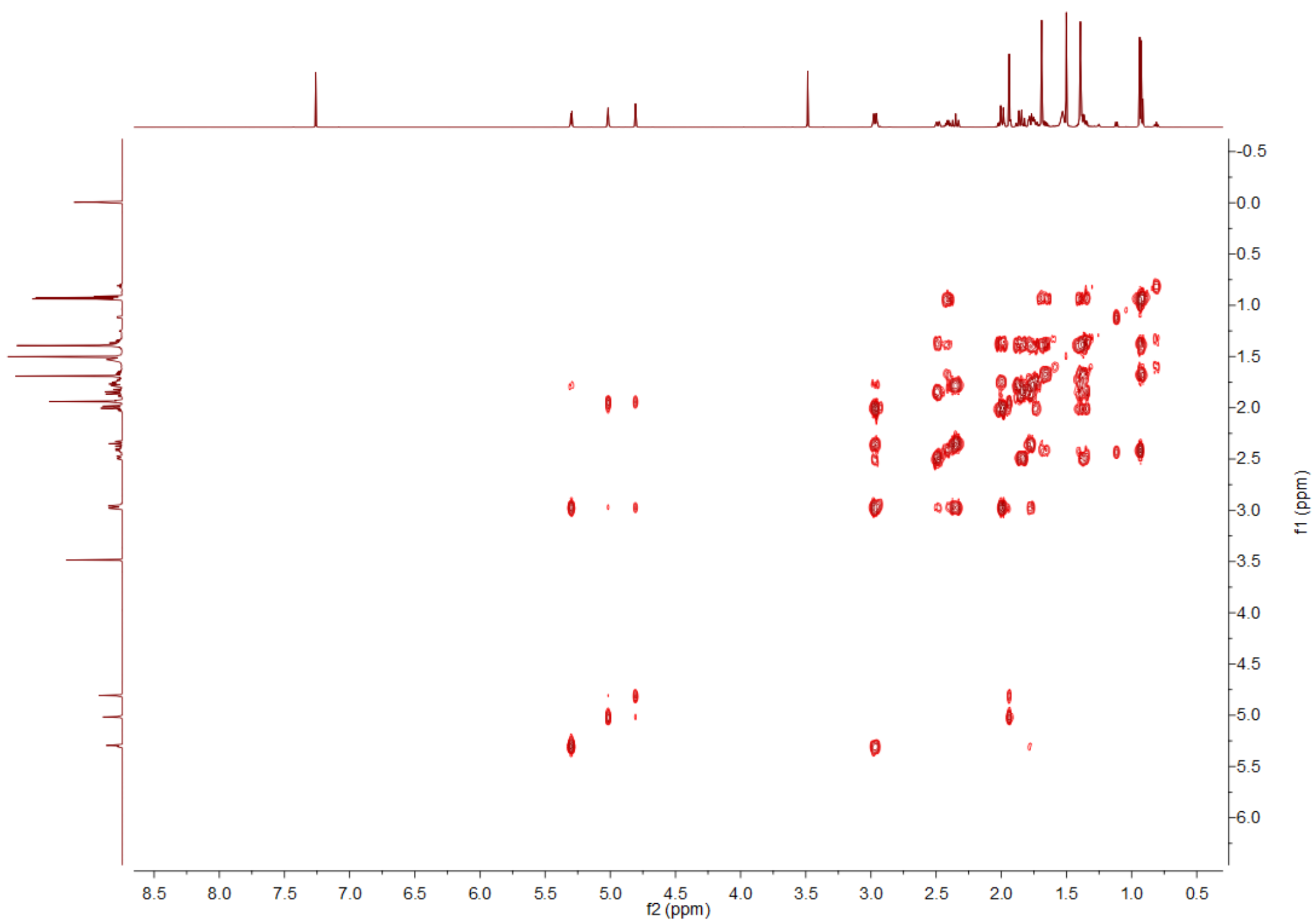

Figure S72. ^{1}H–^{1}H COSY spectrum of compound **6** (recorded in $CDCl_3$).

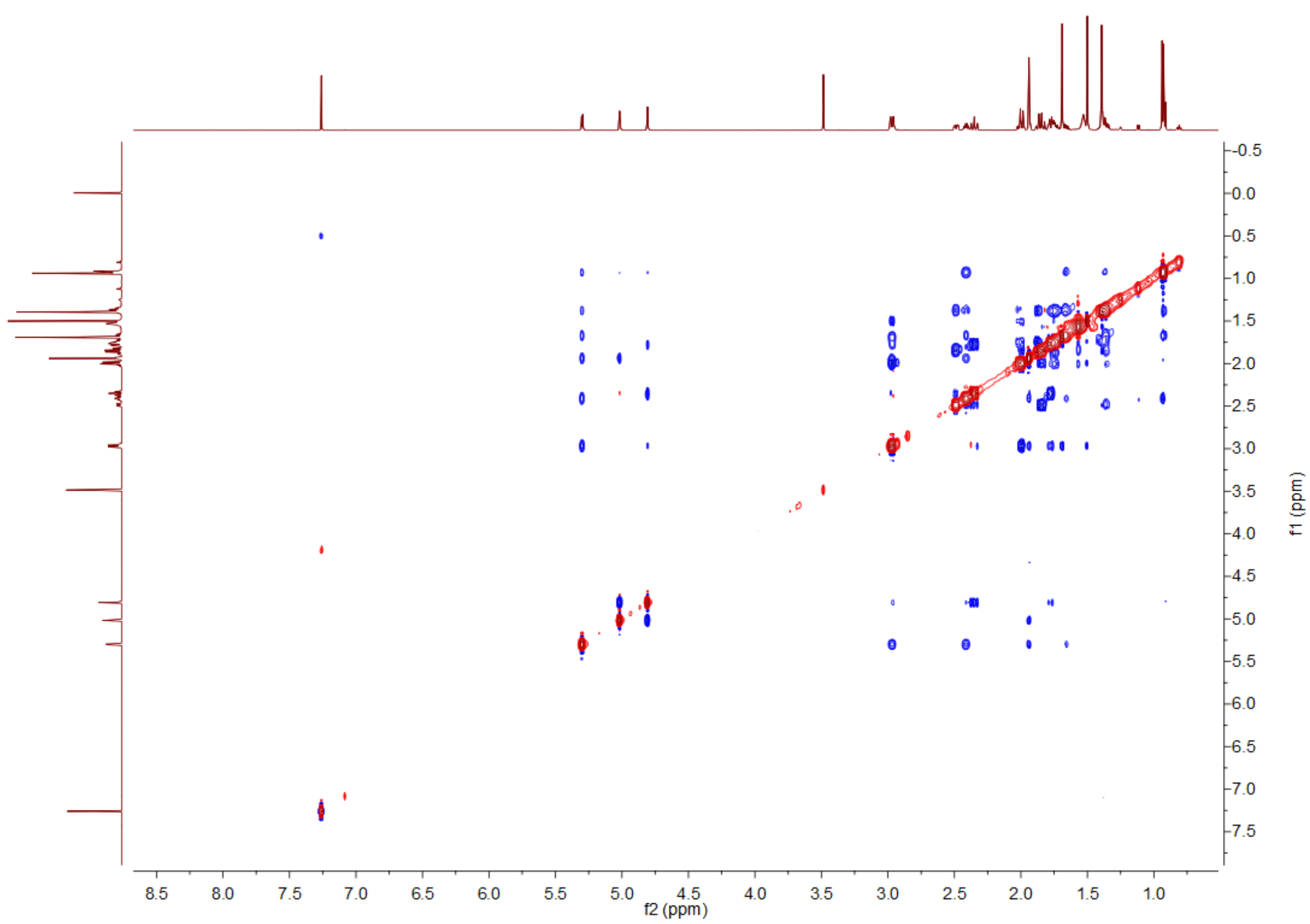

Figure S73. ROESY spectrum of compound **6** (recorded in $CDCl_3$).

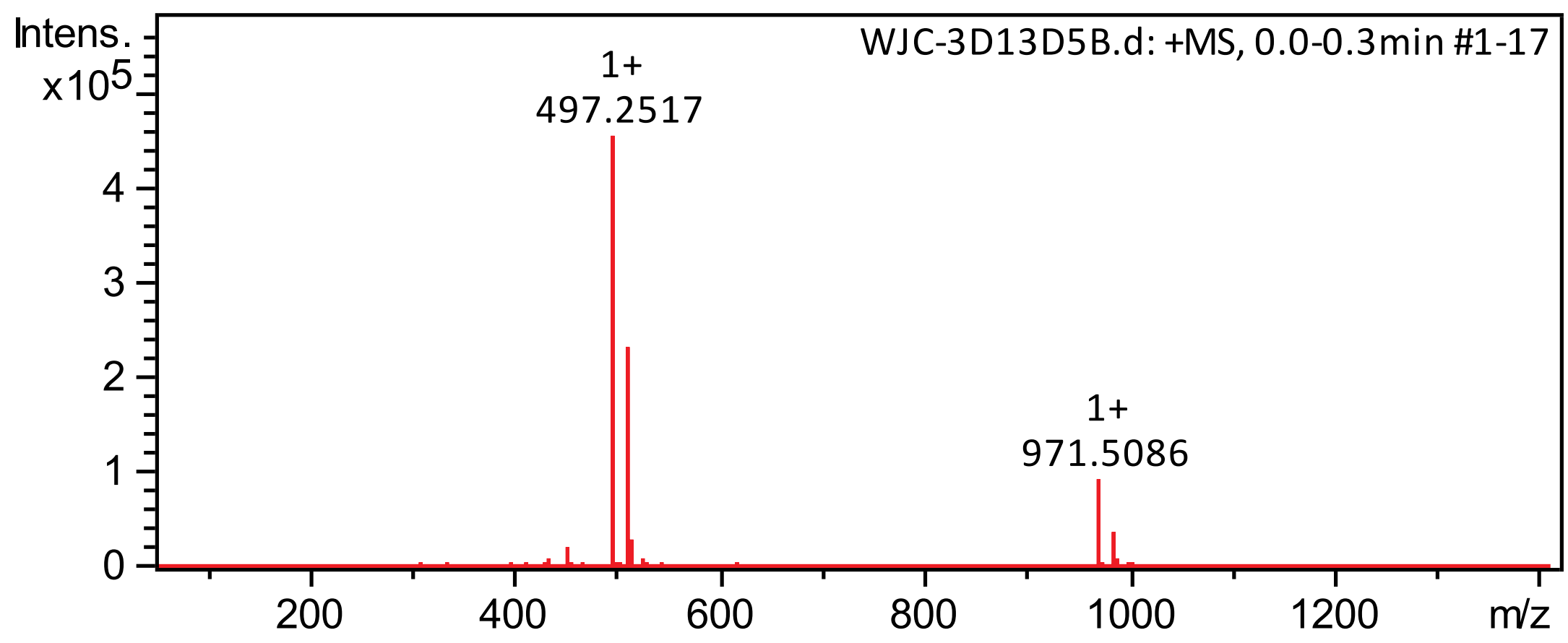

Figure S74. HRESIMS spectrum of compound **6**.

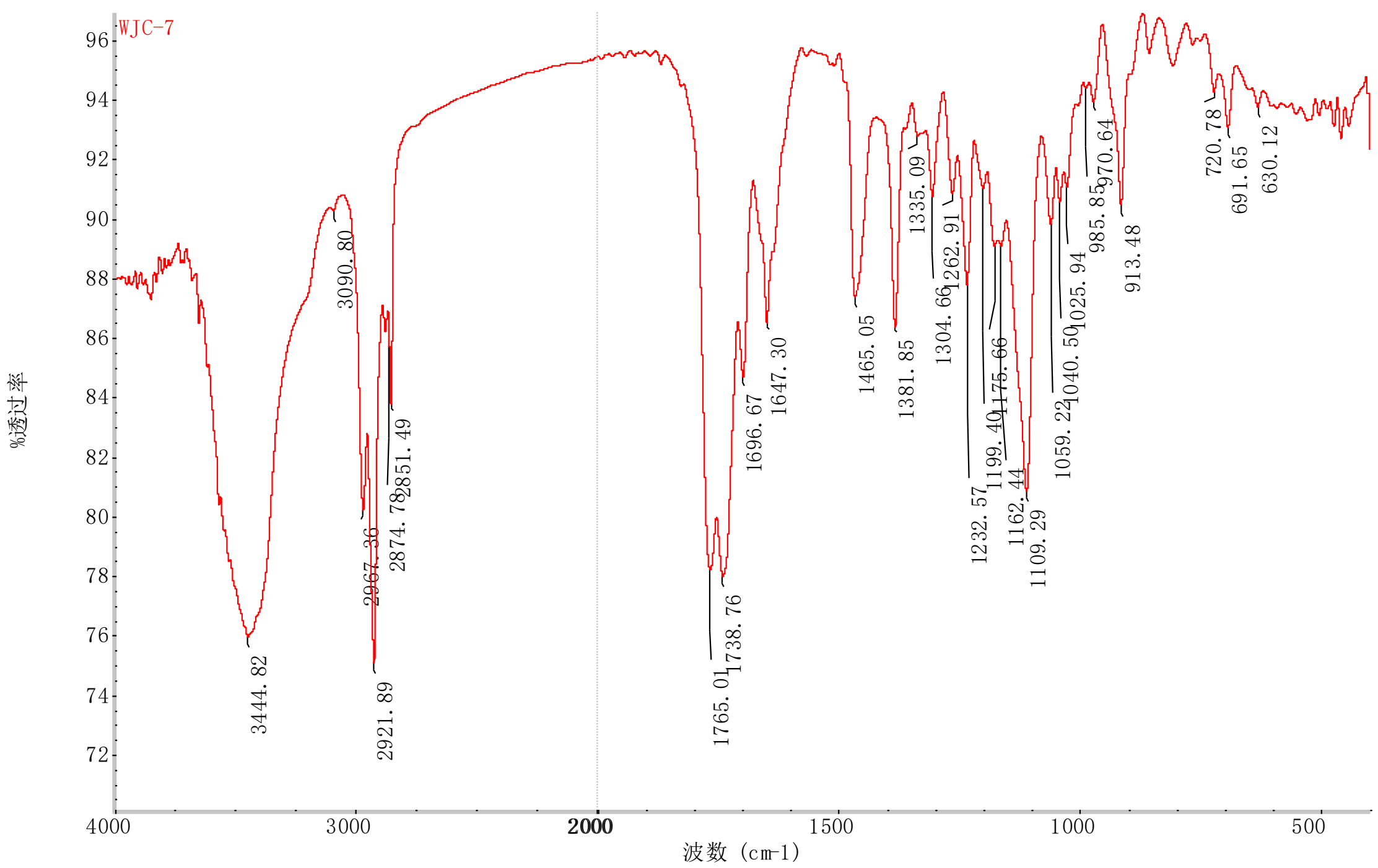

Figure S75. IR spectrum of compound **6**.

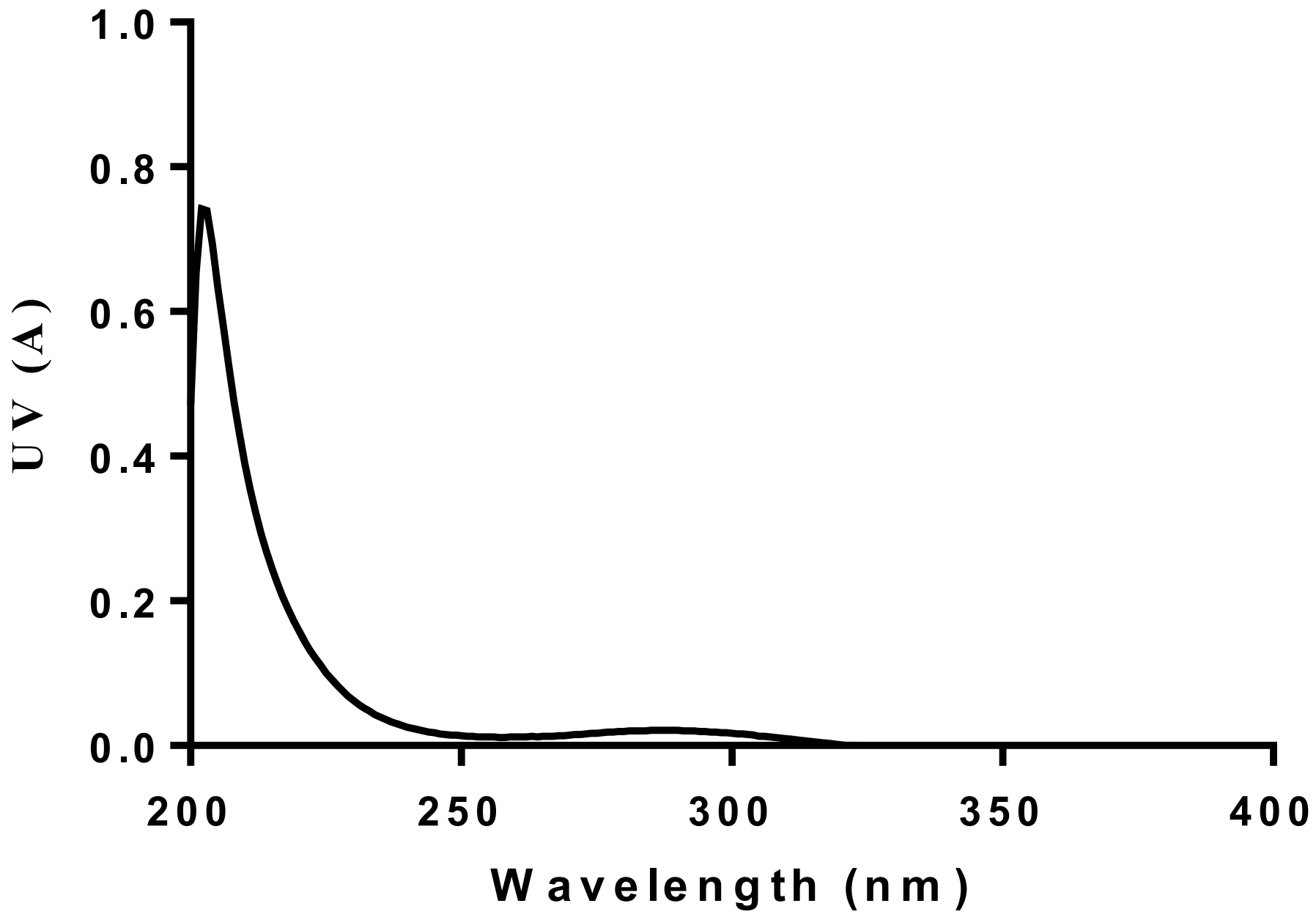

Figure S76. UV spectrum of compound **6**.

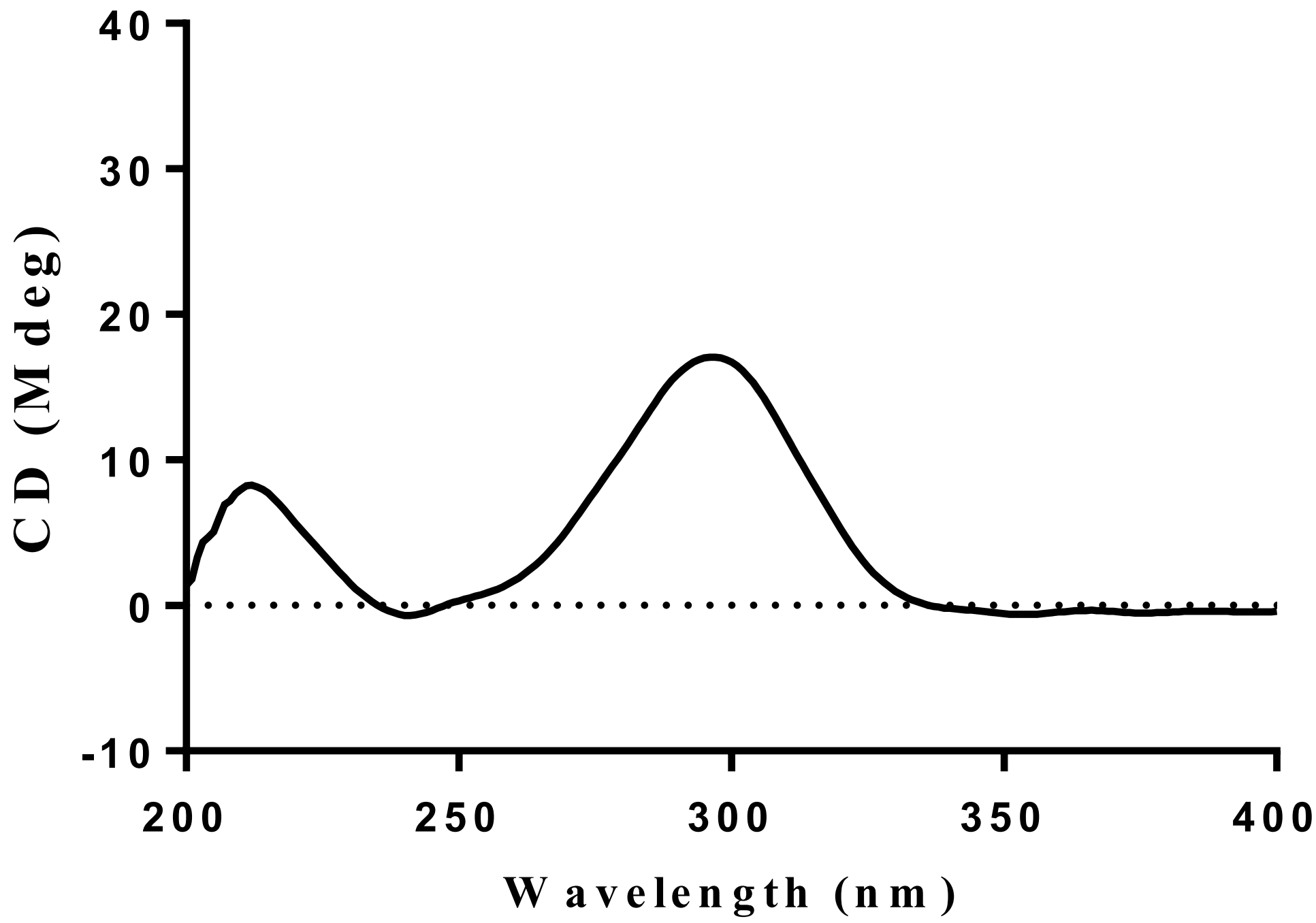

Figure S77. CD spectrum of **6** in MeOH.

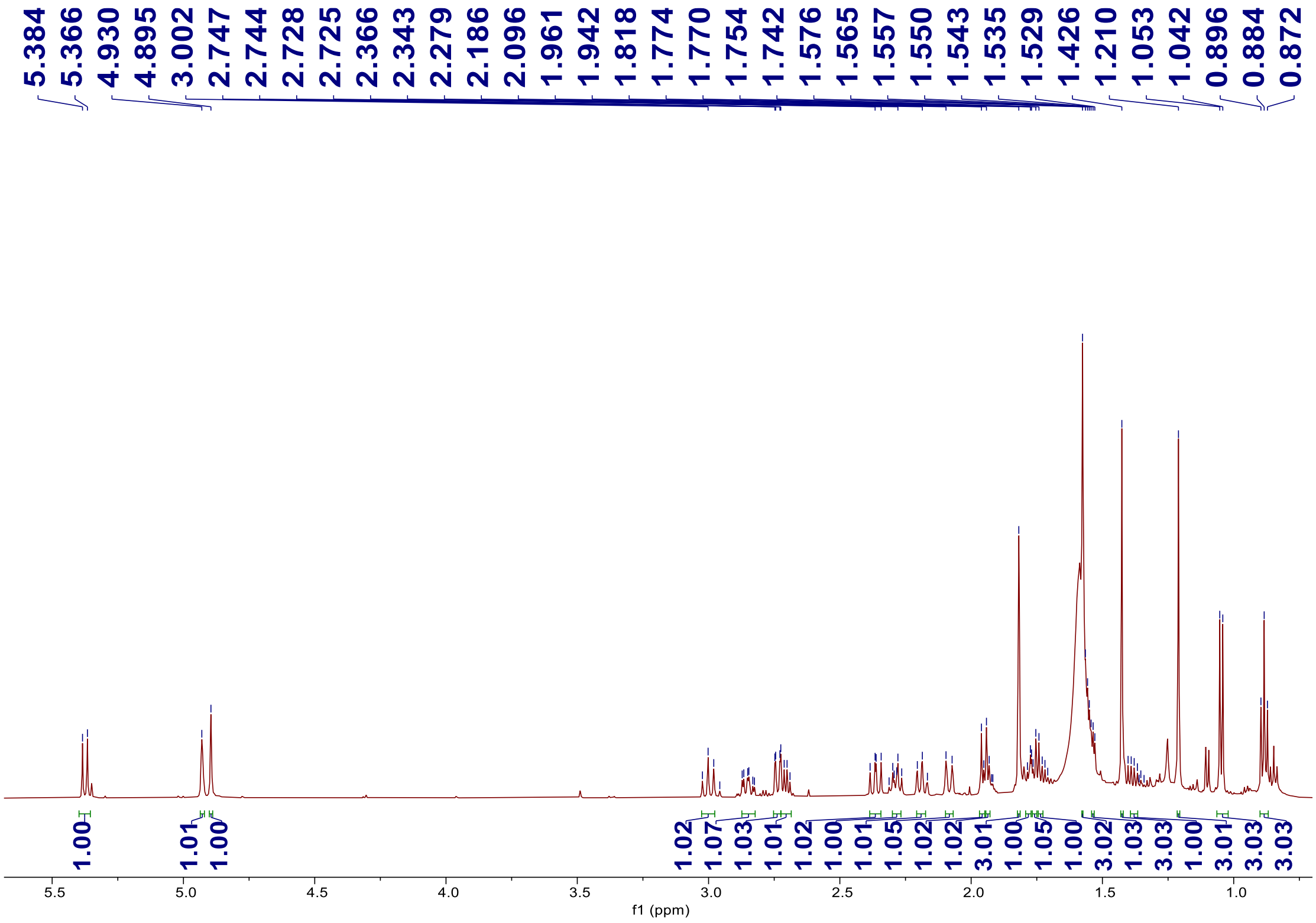

Figure S78. ^{1}H NMR spectrum of compound **7** (recorded in $CDCl_3$).

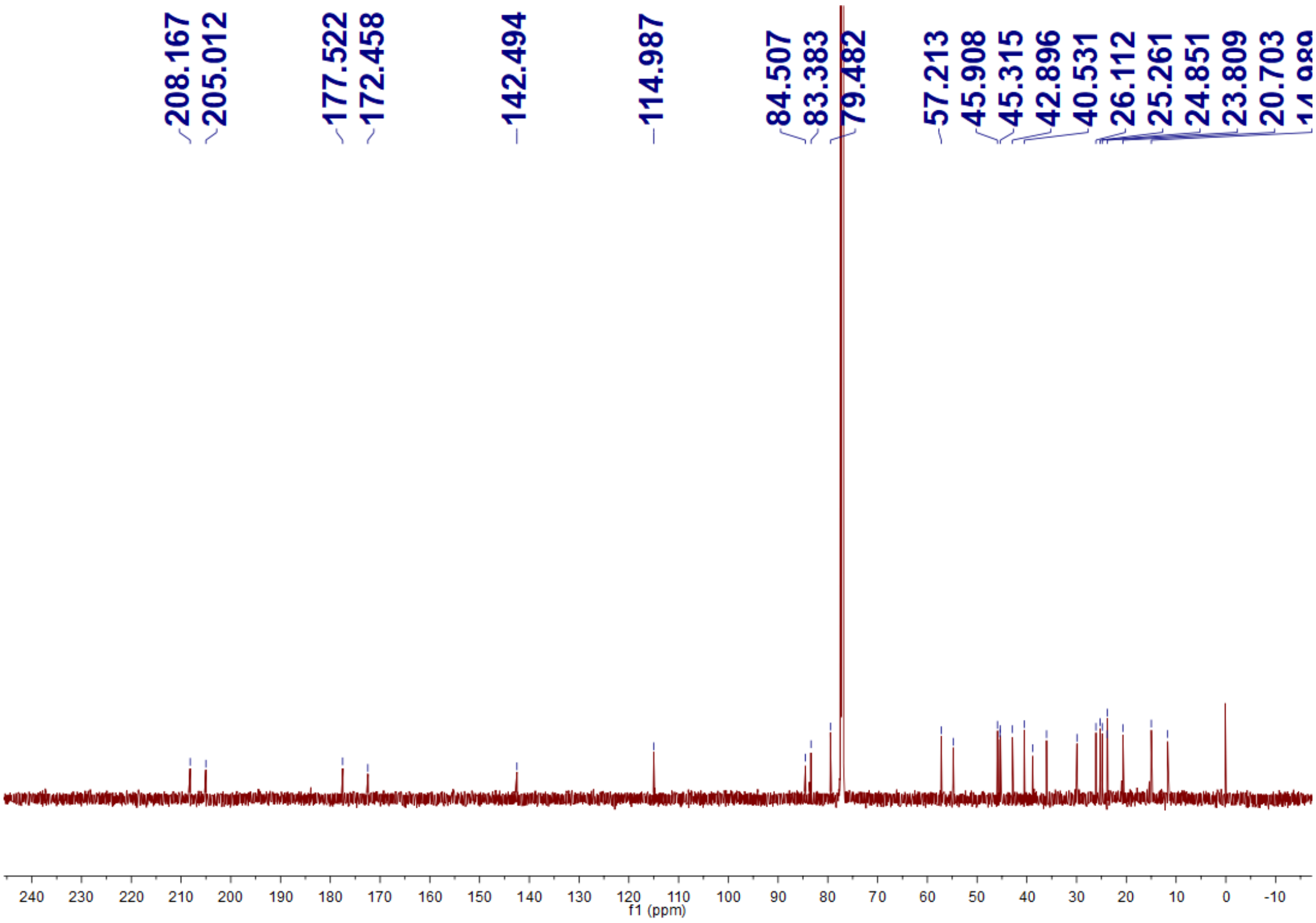

Figure S79. ^{13}C NMR spectrum of compound **7** (recorded in $CDCl_3$).

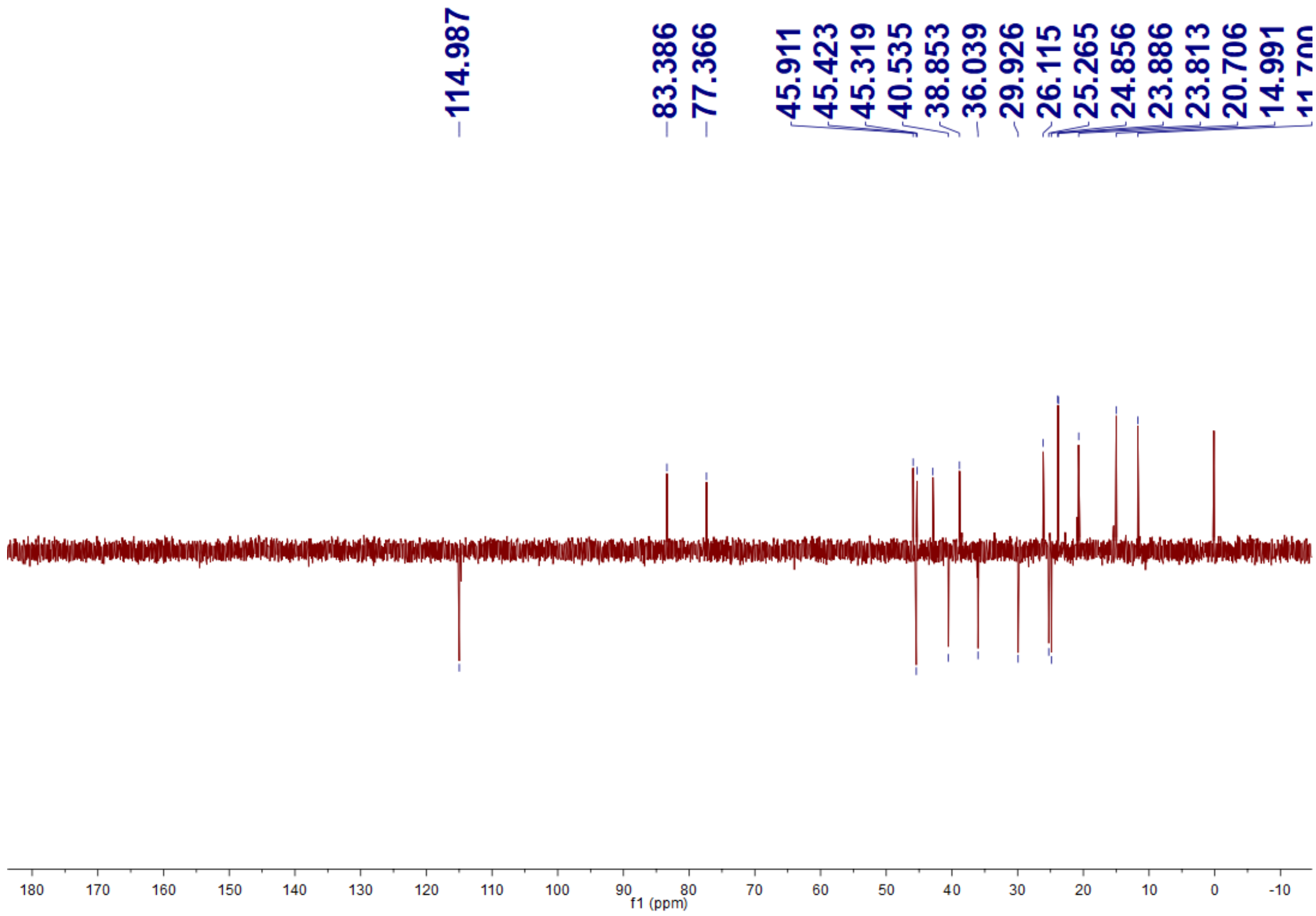

Figure S80. DEPT spectrum of compound **7** (recorded in $CDCl_3$).

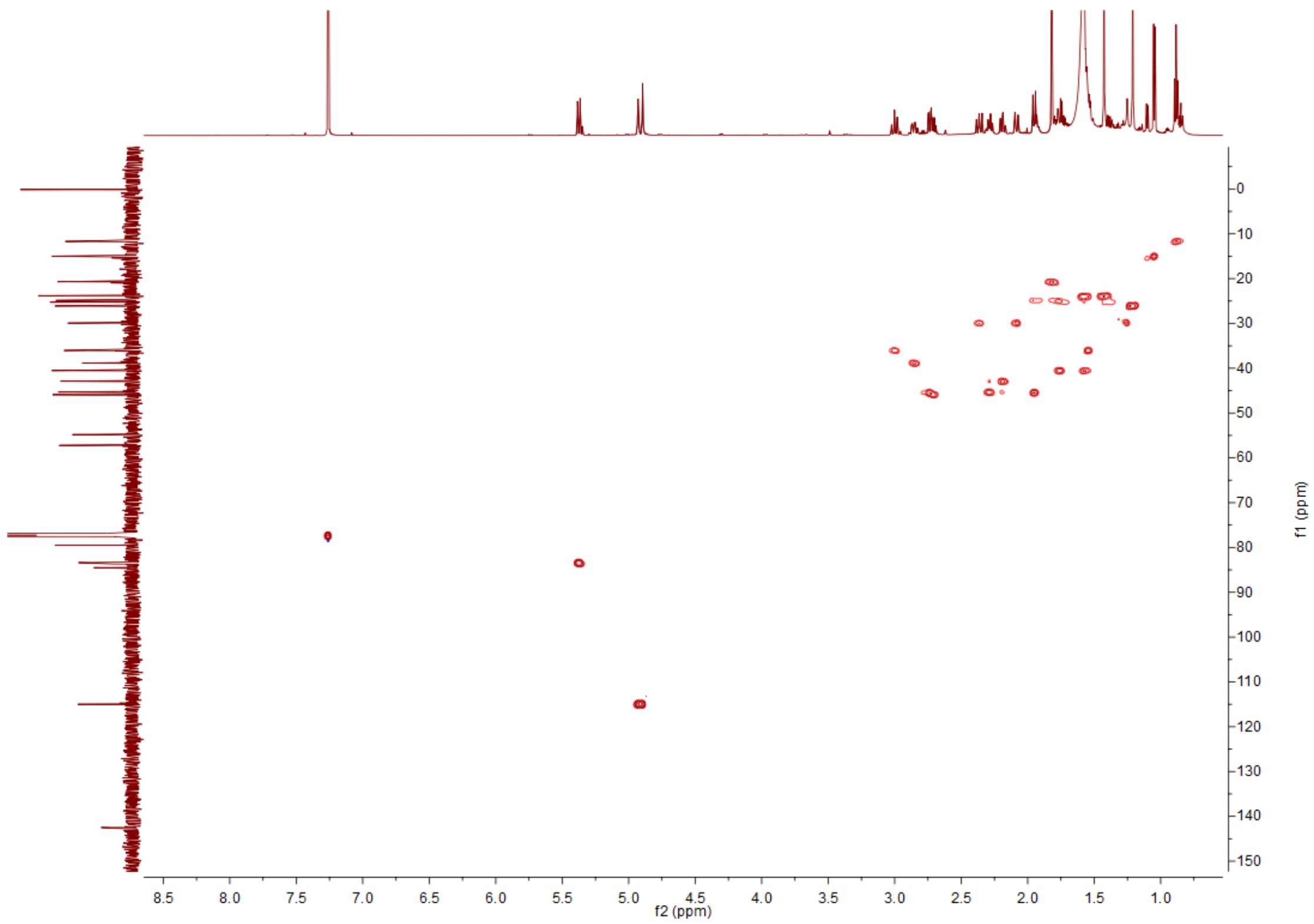

Figure S81. HSQC spectrum of compound **7** (recorded in $CDCl_3$).

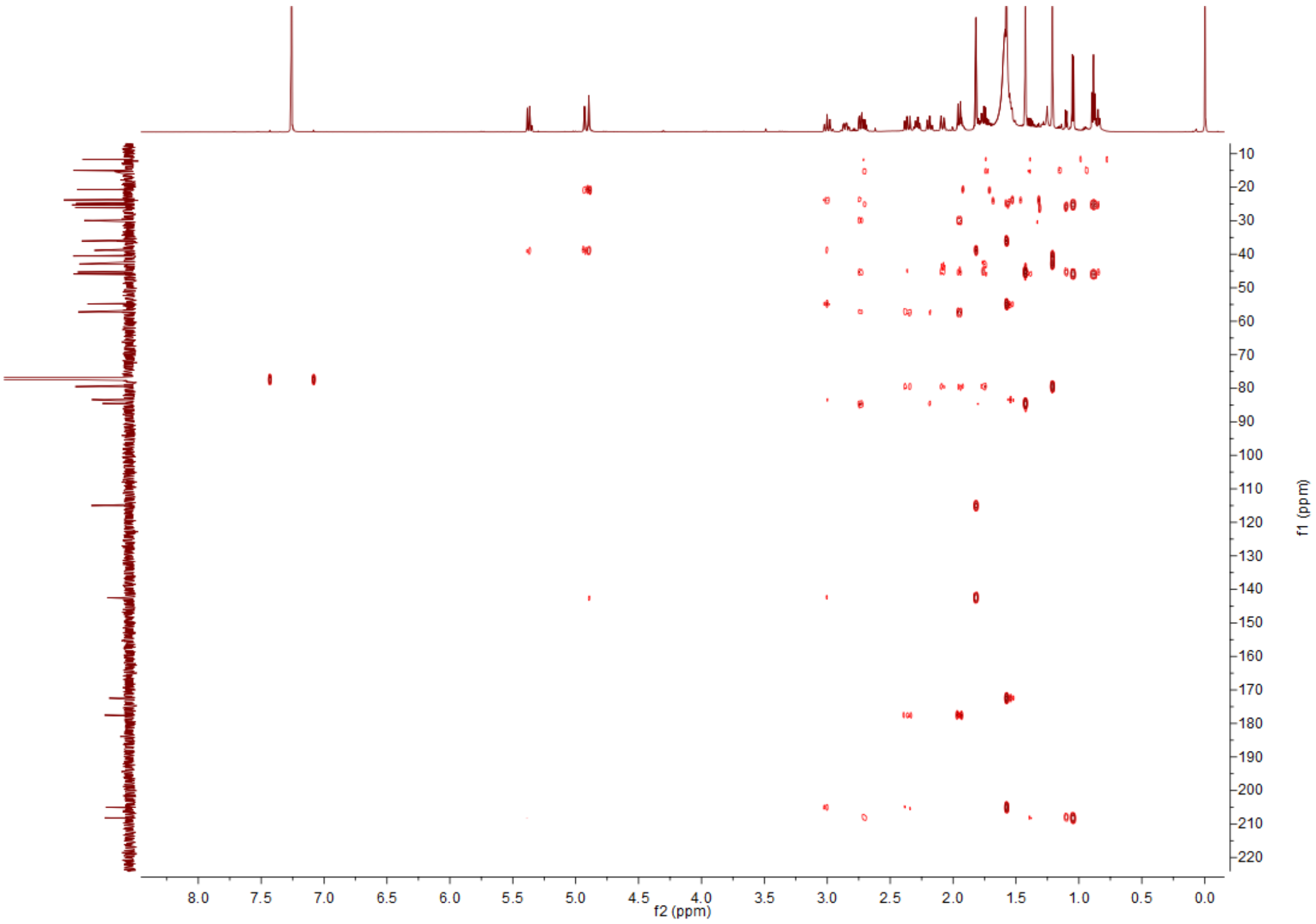

Figure S82. HMBC spectrum of compound **7** (recorded in $CDCl_3$).

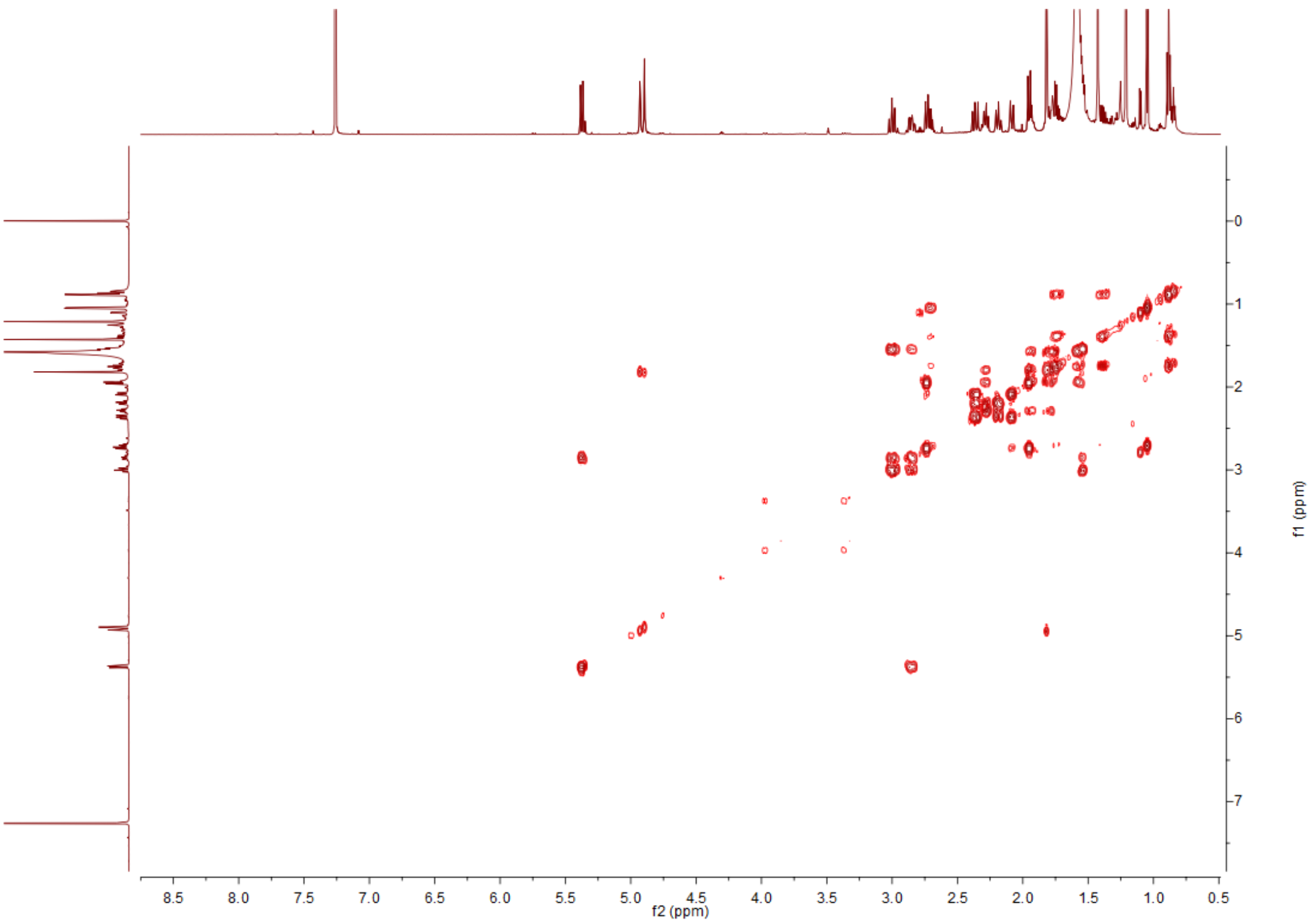

Figure S83. $^1H-^1H$ COSY spectrum of compound **7** (recorded in $CDCl_3$).

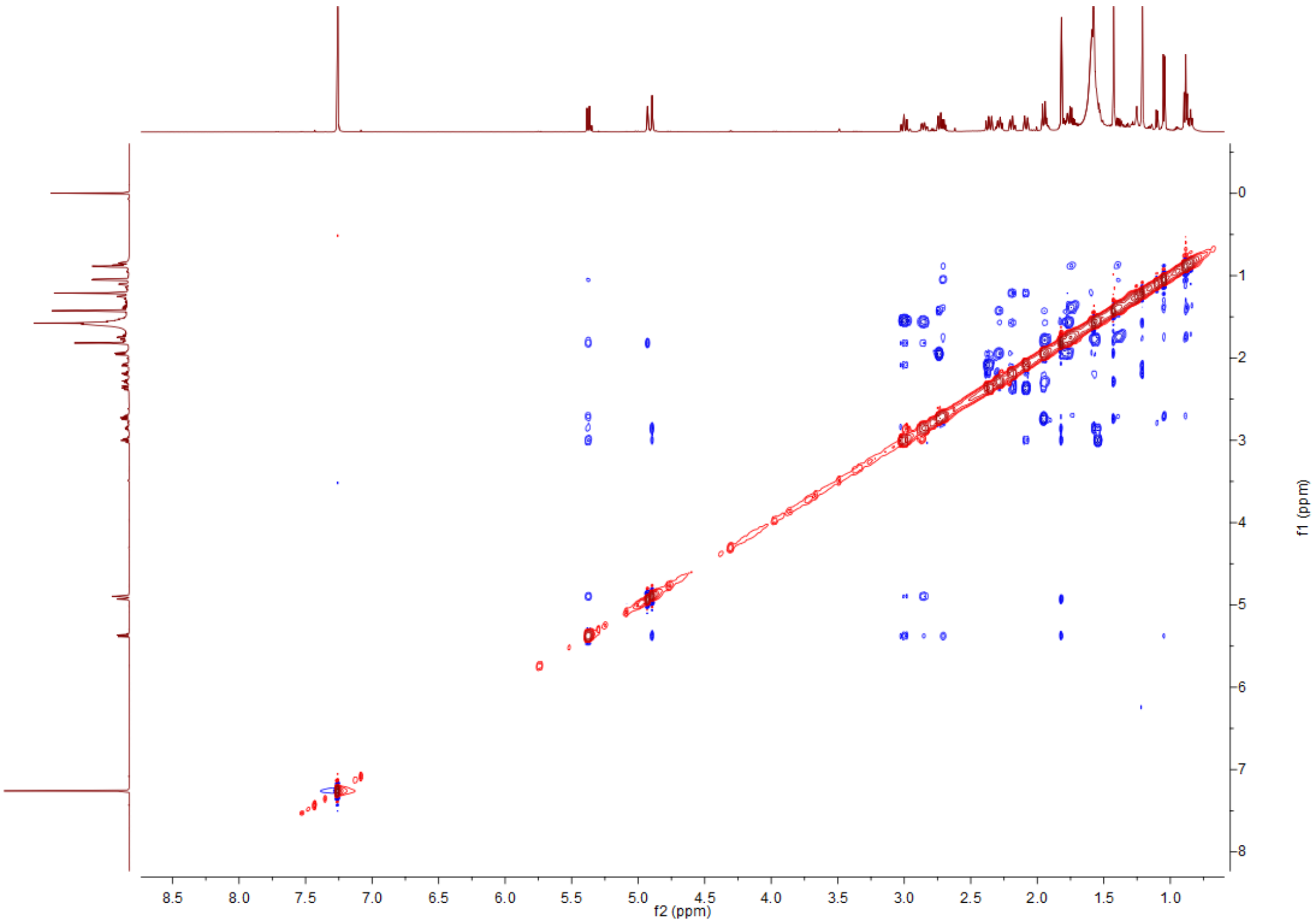

Figure S84. ROESY spectrum of compound **7** (recorded in $CDCl_3$).

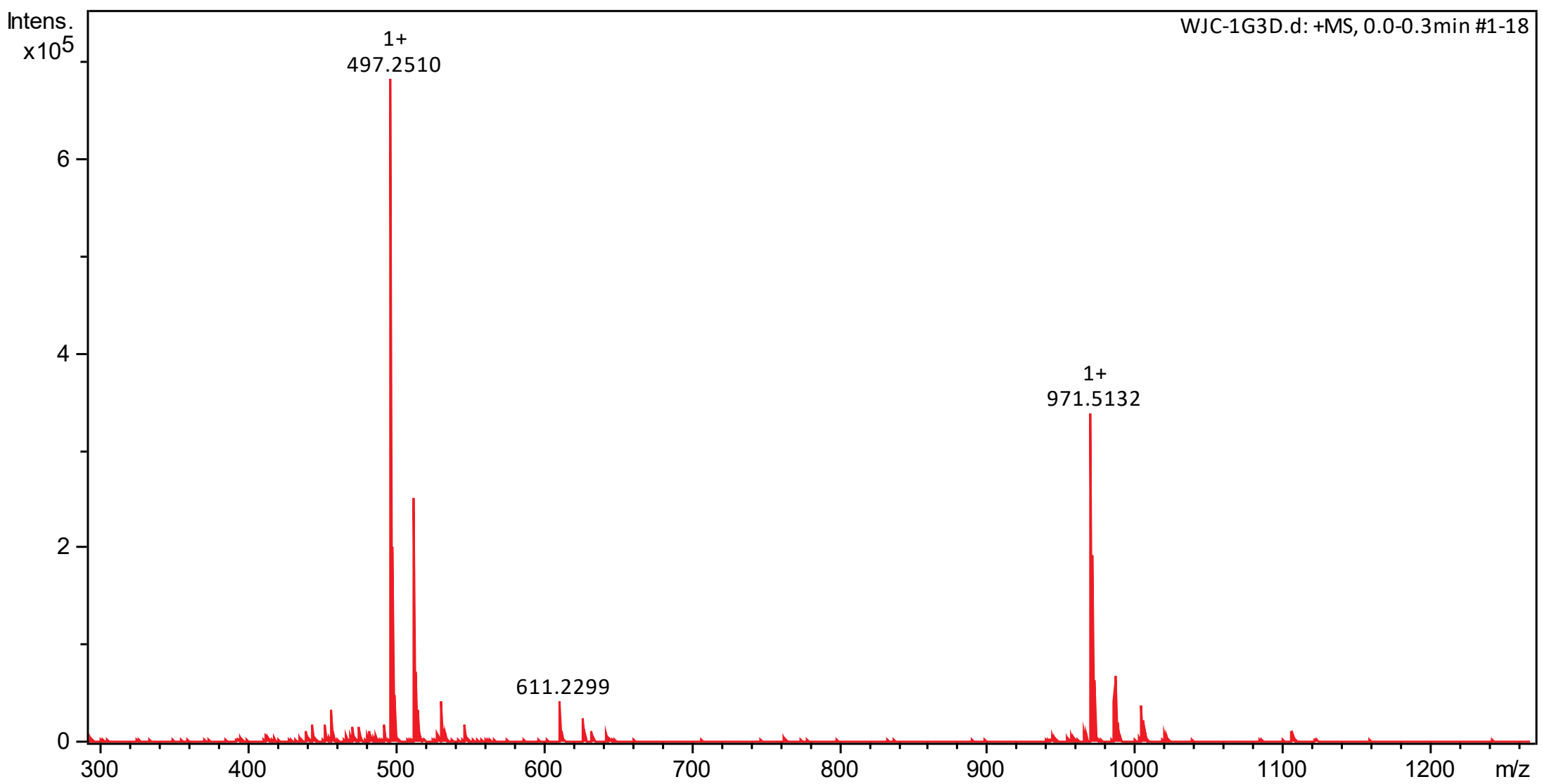

Figure S85. HRESIMS spectrum of compound **7**.

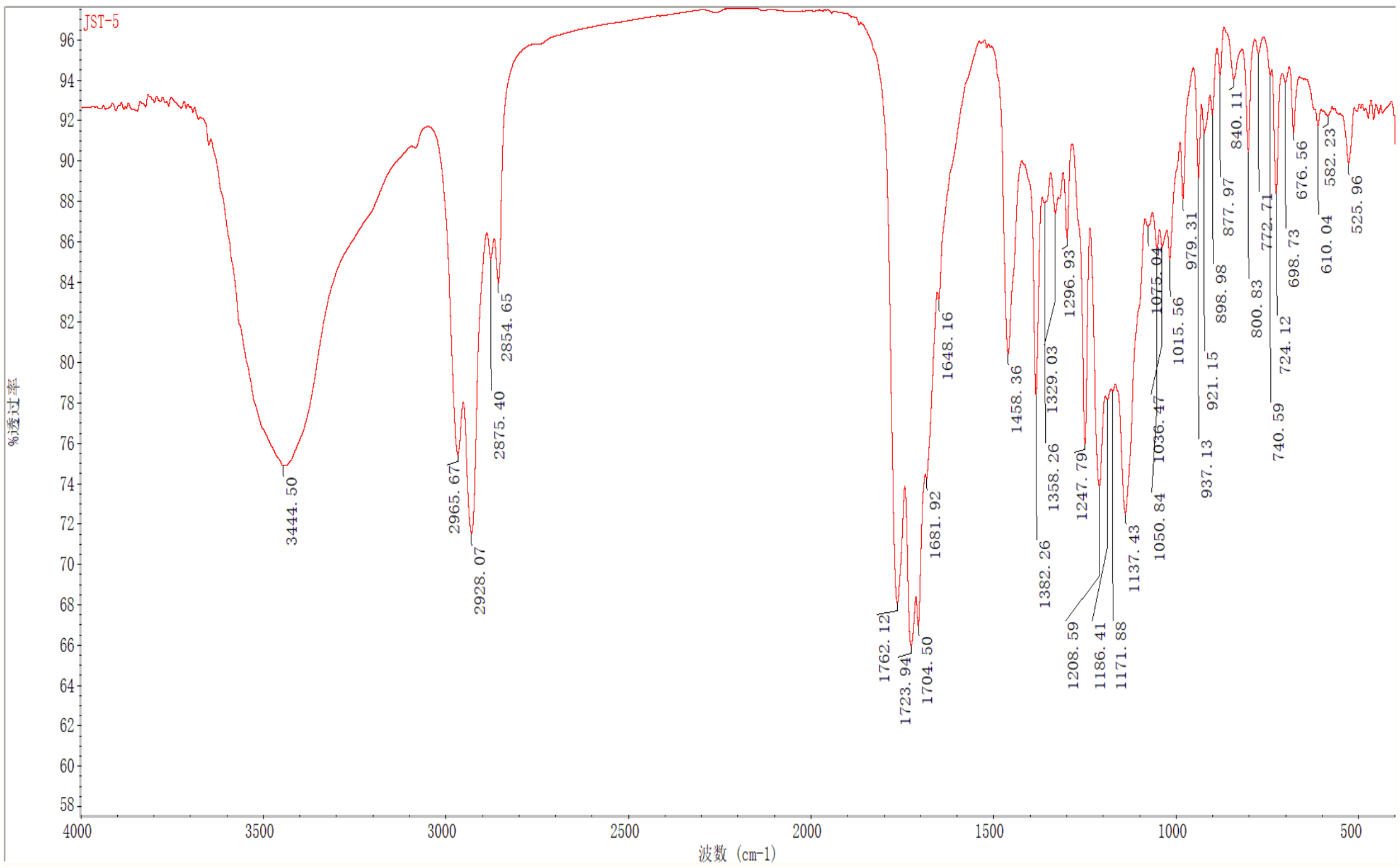

Figure S86. IR spectrum of compound **7**.

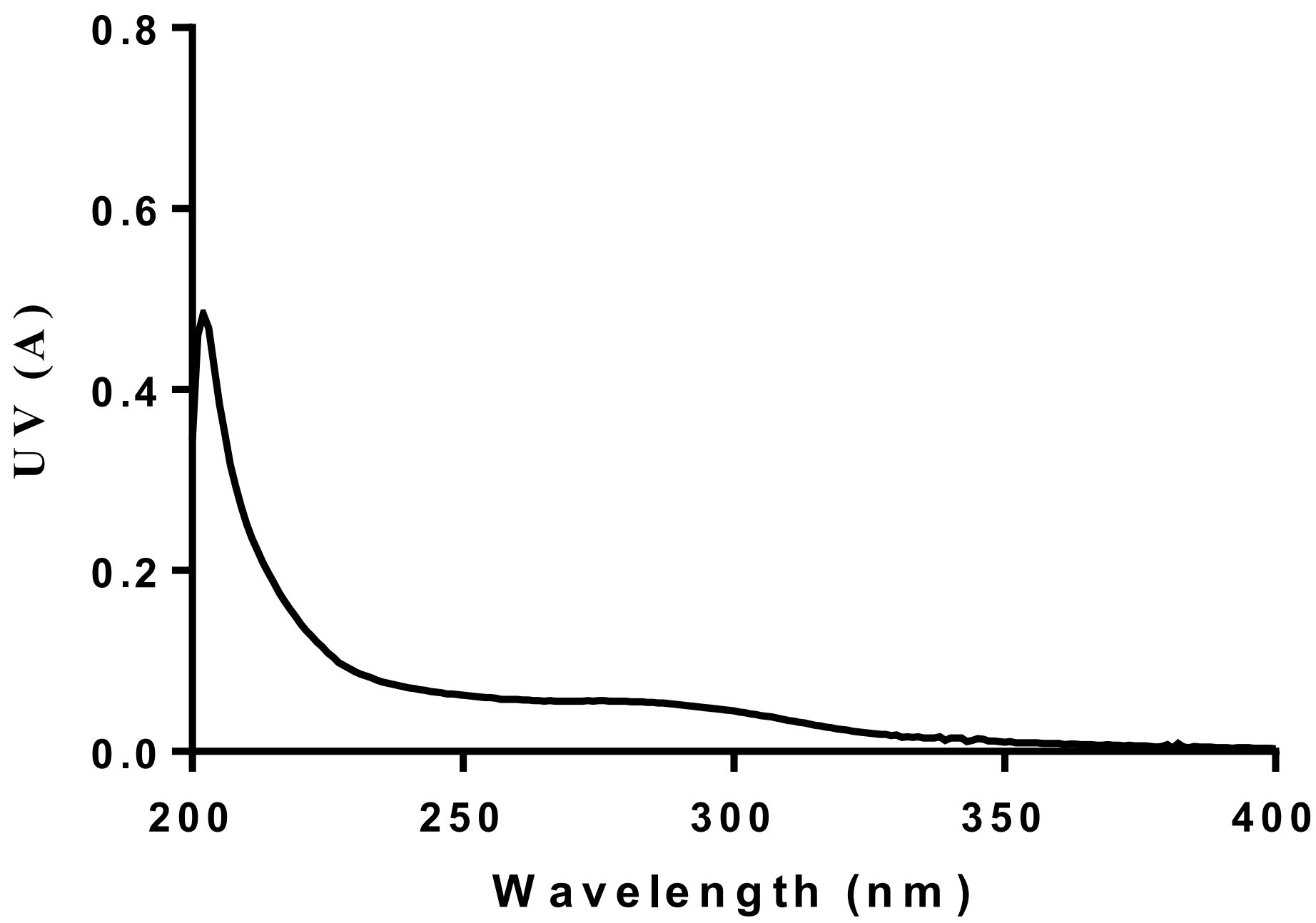

Figure S87. UV spectrum of compound **7**.

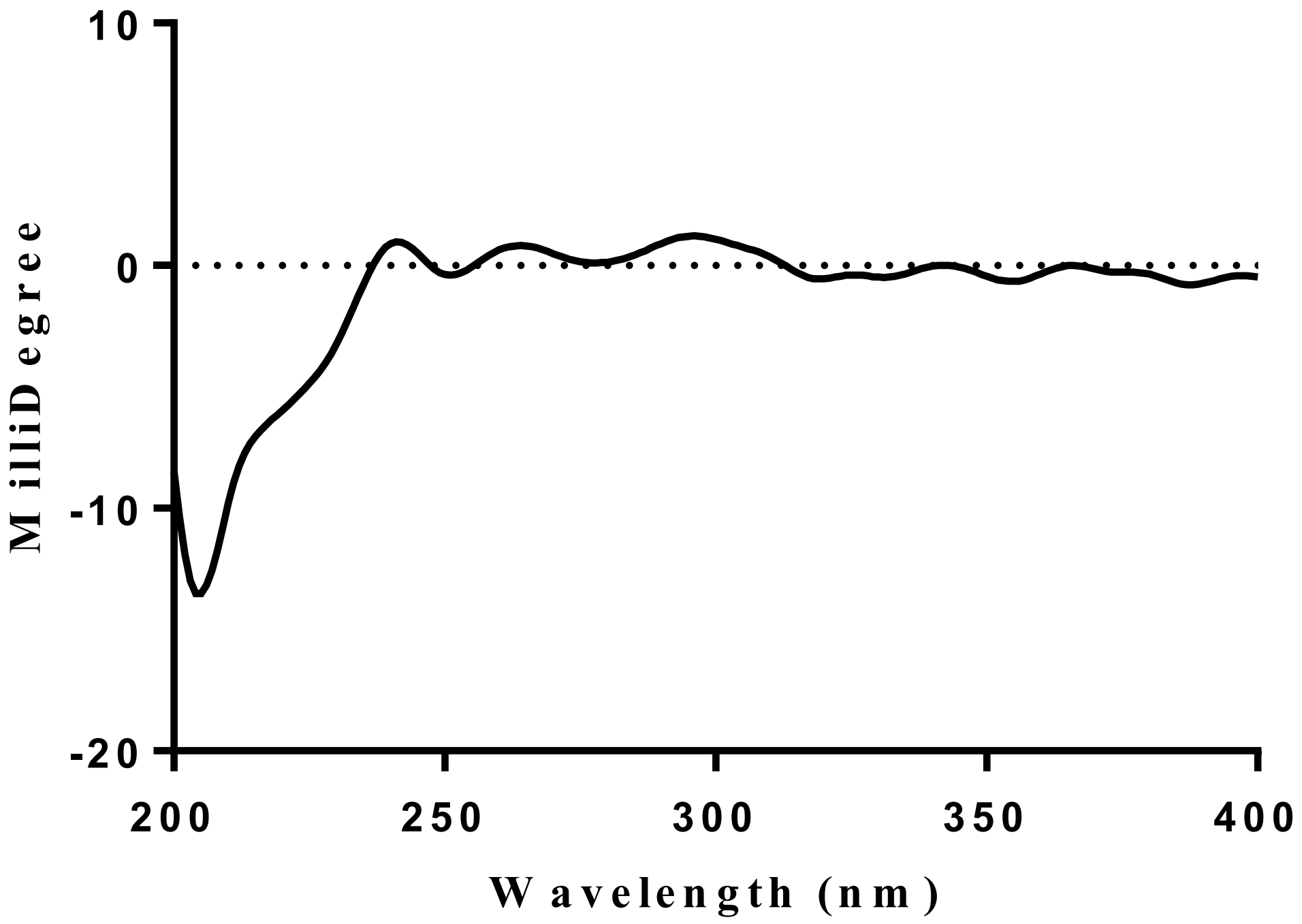

Figure S88. CD spectrum of **7** in MeOH.

Supplementary Tables

TABLE S1 ^{1}H and ^{13}C NMR data of **1** and **2** in $CDCl_3$ (δ in ppm and *J* in Hz).

No.	1		2	
	δ_H[a]	δ_C[b]	δ_H[a]	δ_C[b]
1		170.9 C		172.6 C
2		54.9 C		54.8 C
3	2.61, t (13.2); 1.63, ddd (13.2, 3.0, 1.2)	31.0 CH_2	2.88, m; 1.58, m	36.1 CH_2
4	3.09, ddd (12.0, 6.6, 3.0)	37.8 CH	2.87, m	38.8 CH
5	5.34, dd (6.6, 0.6)	81.4 CH	5.36, d (10.8)	83.5 CH
6		201.6 C		204.0 C
7	1.61, s	24.4 CH_3	1.58, s	23.7 CH_3
8		140.4 C		142.5 C
9	5.01, s; 4.79, s	114.9 CH_2	4.94, s; 4.88, s	114.7 CH_2
10	1.86, s	22.9 CH_3	1.82, s	20.8 CH_3
11		209.6 C		208.1 C
12	2.72, m	40.1 CH	2.92, m	38.7 CH
13	1.14, d (7.2)	18.2 CH_3	1.14, d (6.6)	17.9 CH_3
14	1.05, d (7.2)	16.8 CH_3	1.10, d (6.6)	17.9 CH_3
16		175.5 C		177.9 C
17		60.9 C		59.8 C
18	2.39, ddd (12.0, 4.8, 2.4); 1.75, m	30.1 CH_2	2.10, t (12.6); 1.83, m	31.9 CH_2
19	1.29, td (12.0, 4.8)	49.7 CH	1.31, m	49.8 CH
20		77.3 C		77.2 C
21	1.86, m; 1.76, m	39.8 CH_2	1.87, m; 1.76, m	40.0 CH_2
22	1.75, m; 1.36, m	22.6 CH_2	1.78, m; 1.36, m	22.6 CH_2
23	2.00, td (12.0, 6.0)	48.5 CH	2.06, td (12.0, 6.0)	48.3 CH
24		85.3 C		85.1 C
25	3.55, dd (12.0, 2.4); 1.90, d (12.0)	47.4 CH_2	2.80, dd (11.4, 1.8); 2.02, d (11.4)	48.7 CH_2
26	1.62, s	21.6 CH_3	1.47, s	21.8 CH_3
27	1.35, s	27.0 CH_3	1.33, s	26.9 CH_3

[a]Recorded at 600 MHz. [b]Recorded at 150 MHz. "m" means overlapped or multiplet with other signals.

TABLE S2 ^{1}H and ^{13}C NMR data of **3** and **4** in $CDCl_3$ (δ in ppm and *J* in Hz).

No.	3		4	
	δ_H[a]	δ_C[b]	δ_H[a]	δ_C[b]
1		172.7 C		171.9 C
2		54.7 C		54.6 C
3	2.94, t (13.2); 1.55, dd (12.0, 3.0)	35.9 CH_2	2.35, t (13.2); 1.78, m	32.1 CH_2
4	2.87, td (12.0, 3.6)	38.8 CH	2.98, m	38.8 CH
5	5.38, d (11.4)	83.6 CH	5.32, dd (6.0, 1.2)	81.5 CH
6		204.1 C		204.1 C
7	1.57, s	23.5 CH_3	1.69, s	26.9 CH_3
8		142.5 C		140.4 C
9	4.93, s; 4.87, s	114.9 CH_2	5.01, s; 4.80, s	114.7 CH_2
10	1.81, s	20.6 CH_3	1.92, s	23.0 CH_3
11		208.2 C		209.9 C
12	2.93, m	38.6 CH	2.57, m	40.1 CH
13	1.14, d (6.6)	18.0 CH_3	1.13, d (7.2)	18.9 CH_3
14	1.10, d (6.6)	17.9 CH_3	0.97, d (7.2)	16.1 CH_3
16		177.2 C		175.8 C
17		58.1 C		62.8 C
18	2.05, m; 1.94, m	33.1 CH_2	2.48, ddd (12.0, 4.8, 2.4); 1.83, m	28.9 CH_2
19	2.43, m	44.9 CH	1.36, m	49.9 CH
20		80.0 C		77.3 C
21	1.99, m; 1.75, m	38.0 CH_2	1.86, m; 1.76, m	39.8 CH_2
22	1.76, m; 1.41, m	23.3 CH_2	1.74, m; 1.37, m	22.6 CH_2
23	2.06, m	45.1 CH	2.00, m	49.1 CH
24		87.5 C		84.8 C
25	2.52, d (12.0); 2.14, d (12.0)	41.9 CH_2	2.94, dd (12.0, 1.8); 1.99, m	48.5 CH_2
26	1.42, s	23.5 CH_3	1.50, s	22.0 CH_3
27	1.33, s	28.6 CH_3	1.39, s	27.0 CH_3

[a]Recorded at 600 MHz. [b]Recorded at 150 MHz. "m" means overlapped or multiplet with other signals.

TABLE S3 ^{1}H and ^{13}C NMR data of **5–7** in $CDCl_3$ (δ in ppm and J in Hz).

No.	5		6		7	
	δ_H[a]	δ_C[b]	δ_H[a]	δ_C[b]	δ_H[a]	δ_C[b]
1		171.9 C		171.9 C		172.5 C
2		54.7 C		54.6 C		54.8 C
3	2.39, t (13.8); 1.81, m	32.3 CH_2	2.35, t (13.8); 1.78, m	32.2 CH_2	3.00, t (13.2); 1.55, m	36.0 CH_2
4	2.97, m	39.0 CH	2.96, m	38.9 CH	2.85, td (12.0, 3.6)	38.9 CH
5	5.30, dd (6.0, 1.2)	81.3 CH	5.30, dd (6.0, 1.2)	82.3 CH	5.38, d (12.0)	83.4 CH
6		204.2 C		204.1 C		205.0 C
7	1.70, s	27.0 CH_3	1.69, s	27.0 CH_3	1.58, s	23.9 CH_3
8		140.3 C		140.3 C		142.5 C
9	5.02, s; 4.81, s	114.9 CH_2	5.02, s; 4.81, s	114.8 CH_2	4.93, s; 4.90, s	115.0 CH_2
10	1.93, s	23.1 CH_3	1.94, s	23.1 CH_3	1.82, s	20.7 CH_3
11		209.6 C		209.7 C		208.2 C
12	2.43, m	46.9 CH	2.41, m	46.6 CH	2.71, m	45.9 CH
13	1.12, d (7.2)	16.3 CH_3	0.94, d (7.2)	13.1 CH_3	1.05, d (6.6)	15.0 CH_3
14	1.61, m; 1.33, m	24.1 CH_2	1.66. m; 1.36, m	26.5 CH_2	1.73, m; 1.38, m	25.3 CH_2
15	0.81, t (7.2)	11.8 CH_3	0.93, t (7.2)	11.6 CH_3	0.88, t (7.2)	11.7 CH_3
16		175.8 C		175.8 C		177.5 C
17		62.7 C		62.9 C		57.2 C
18	2.46, ddd (12.0, 4.8, 2.4); 1.84, m	29.0 CH_2	2.49, ddd (12.0, 4.8, 2.4); 1.83, m	28.8 CH_2	2.37, dd (13.8, 11.4); 2.08, dd (13.8, 1.2)	29.9 CH_2
19	1.36, m	49.9 CH	1.37, m	49.9 CH	2.19, m	42.9 CH
20		77.3 C		77.3 C		79.5 C
21	1.86, m; 1.36, m	39.8 CH_2	1.86, m; 1.77, m	39.8 CH_2	1.76, m; 1.58, m	40.5 CH_2
22	1.74, m; 1.39, m	22.6 CH_2	1.74, m; 1.39, m	22.6 CH_2	1.94, m; 1.77, m	24.9 CH_2
23	2.00, td (12.0, 6.0)	49.1 CH	2.00, td (12.0, 6.0)	49.1 CH	2.29, m	45.3 CH
24		84.7 C		84.8 C		84.5 C
25	2.94, dd (12.0, 2.4); 1.99, d (12.0)	48.6 CH_2	2.97, dd (12.0, 2.4); 1.99, d (12.0)	48.5 CH_2	2.73, m; 1.95, m	45.4 CH_2
26	1.50, s	22.0 CH_3	1.50, s	22.0 CH_3	1.43, s	23.8 CH_3
27	1.39, s	27.0 CH_3	1.39, s	27.0 CH_3	1.21, s	26.1 CH_3

[a]Recorded at 600 MHz. [b]Recorded at 150 MHz. "m" means overlapped or multiplet with other signals.

TABLE S4 *In vivo* pharmacokinetic properties of HPJ.

Parameter [a]	units	*i.v.*	*i.p.*	*i.g.*
dosage	mg/kg	5	10	20
C_{max}	μg/L	4662.933	5939.768	4471.89
T_{max}	h	0.25	1	2
$T_{1/2}$	h	1.145	1.44	2.341
$AUC_{(0-t)}$	μg/L*h	8808.908	14694.469	19872.528
$AUC_{(0-\infty)}$	μg/L*h	8896.302	15102.621	20008.038
$MRT_{(0-t)}$	h	1.611	2.273	4.602
$MRT_{(0-\infty)}$	h	1.824	2.496	4.869
$AUMC_{(0-t)}$		14273.279	33299.827	92580.152
$AUMC_{(0-\infty)}$		16357.822	37528.056	98542.644
CLz	L/h/kg	0.565	0.665	1.005
Vz or Vz/F	L/kg	0.921	1.392	3.36
F			84.9%	56.2%

[a] C_{max}, maximum plasma concentration; T_{max}, time to reach maximum concentration; $T_{1/2}$, half-life; AUC, area underthe curve; MRT, mean residence time; AUMC, area under the first moment curve; CLz, plasma clearance; Vz, volume of distribution; F, Absolute Bioavailability.

TABLE S5 PCR sequence.

	Forward Sequence	Reverse Sequence
TNF	CGTGGAACTGGCAGAAGAGG	GGAATGAGAAGAGGCTGAGACA
IL-6	GCCACTCACCTCTTCAGAACGA	GCCTCTTTGCTGCTTTCACACA
S100A9	CACCCAGACACCCTGAACCA	GGTTAGCCTCGCCATCAGCAT
IL-8	ATGGGTGAAGGCTACTGTTGG	CCGAATTGGAAAGGGAAATA
CCL20	GCGAATCAGAAGCAAGCAACTT	AGCATTGATGTCACAGCCTTCA
CD36	GTTGCCATAATTGAGTCCT	GAAAAGAATCTCAATGTCCGA
CD206	ATCCACGAGCAAATGTACCTCA	TAGCCAGTTCAGATACCGGAA
IL-23A	TGACTGAGCCCTTAGTGCCAA	CAGCACAAGTCCTAATGGGTT
MCP-1	CTACTCATTCACCAGCAAGAT	CCATTCCTTCTTGGGGTCA
CXCL1	ACCGAAGTCATAGCCACACTCA	CCTTCAGGAACAGCCACCAGT
β-actin	CATTGCTGACAGGATGCAGAAGG	TGCTGGAAGGTGGACAGTGAGG

TABLE S6 Gibbs free energies[a] and equilibrium populations[b] of low-energy conformers of 2*R*,4*S*,5*S*,17*S*,19*S*,20*R*,23*R*,24*S*,*M*-**4**.

Conformers	ΔG (a.u.)	*P*(%)/100	G (a.u.)
2*R*,4*S*,5*S*,17*S*,19*S*,20*R*,23*R*,24*S*,*M*-**4** (1)_tddft_	0.0004	26.69	-1577.837278
2*R*,4*S*,5*S*,17*S*,19*S*,20*R*,23*R*,24*S*,*M*-**4** (2)_tddft_	0.0013	10.27	-1577.836376
2*R*,4*S*,5*S*,17*S*,19*S*,20*R*,23*R*,24*S*,*M*-**4** (3)_tddft_	0.0	40.82	-1577.837679
2*R*,4*S*,5*S*,17*S*,19*S*,20*R*,23*R*,24*S*,*M*-**4** (4)_tddft_	0.00058	22.04	-1577.837097
2*R*,4*S*,5*S*,17*S*,19*S*,20*R*,23*R*,24*S*,*M*-**4** (5)_tddft_	0.00569	0.1	-1577.831986
2*R*,4*S*,5*S*,17*S*,19*S*,20*R*,23*R*,24*S*,*M*-**4** (6)_tddft_	0.0058	0.09	-1577.831875

[a]wB97M-V/def2-TZVP, in a.u. [b]From ΔG values at 298.15 K.

TABLE S7 Cartesian coordinates for the low-energy reoptimized random research conformers of 2*R*,4*S*,5*S*,17*S*,19*S*,20*R*,23*R*,24*S*,*M*-**4** at B3LYP-D3(BJ)/6-31G* level of theory in methanol.

2*R*,4*S*,5*S*,17*S*,19*S*,20*R*,23*R*,24*S*,*M*-**4** (1)			Standard Orientation (A.U.)		
Center number	Atomic number	Atomic Type	X	Y	Z
1	6	C	-0.468681	0.161306	4.537235
2	6	C	-1.431951	-0.354827	1.773379
3	6	C	0.869036	-1.439341	0.322549
4	8	O	0.817705	-3.971758	0.501213
5	6	C	-1.234238	-4.792883	2.164755
6	6	C	-0.1541	-4.653216	4.876873
7	6	C	-0.872521	-2.168835	6.178703
8	6	C	-3.197574	-2.684629	1.761608
9	6	C	-0.834527	-6.589751	6.918929
10	6	C	-0.033836	-5.245968	9.409823
11	6	C	0.45319	-2.402325	8.744102
12	6	C	-2.477142	2.097928	0.606888
13	6	C	-3.259126	2.352154	-2.243854
14	6	C	-3.854723	-0.200575	-3.447622
15	8	O	-2.58764	4.000295	1.869092
16	6	C	-2.055789	-7.389142	1.291597
17	8	O	2.556615	-0.285717	-0.65335
18	1	H	-2.906958	-2.288895	6.623118
19	6	C	-0.518516	-0.56664	10.742533
20	8	O	3.071166	-1.961648	8.281215
21	1	H	1.913762	-4.673549	4.661979
22	8	O	-6.310587	-0.903856	-3.716986
23	6	C	-8.395312	0.82769	-3.701731
24	6	C	-8.055821	3.036436	-1.894072
25	6	C	-5.474265	4.225724	-2.477841
26	8	O	-2.25027	-1.68445	-4.072904
27	6	C	-8.483481	2.430913	0.87729
28	6	C	-8.565028	4.690689	2.604625
29	6	C	-8.817056	0.078428	1.755775
30	6	C	-9.031697	1.692941	-6.411798
31	6	C	-8.1292	0.076963	-8.617186
32	8	O	-10.272275	3.592144	-6.716264
33	6	C	-10.171601	-0.081741	-10.669814
34	6	C	-5.665036	1.236949	-9.647294
35	1	H	-10.030568	-0.331735	-3.131027
36	6	C	-0.927681	3.50106	-3.598153
37	6	C	-12.56471	-1.449181	-9.811115
38	1	H	-1.499492	1.793854	5.290611
39	1	H	1.540869	0.683396	4.481365

40	1	H	-4.161367	-2.968357	-0.041321
41	1	H	-4.636475	-2.621651	3.245425
42	1	H	0.109865	-8.42101	6.657401
43	1	H	-2.887127	-6.948171	6.922847
44	1	H	-1.495966	-5.432803	10.876448
45	1	H	1.71671	-6.058789	10.183814
46	1	H	-2.580569	-7.322654	-0.718135
47	1	H	-0.516512	-8.765382	1.530167
48	1	H	-3.69268	-8.036036	2.397692
49	1	H	0.431545	-0.89364	12.569935
50	1	H	-2.564371	-0.791376	11.047623
51	1	H	-0.14443	1.39558	10.164834
52	1	H	3.971596	-2.142317	9.858477
53	1	H	-9.506014	4.423542	-2.451671
54	1	H	-5.08583	5.848894	-1.25075
55	1	H	-5.518983	4.948149	-4.428054
56	1	H	-9.88394	6.132274	1.878039
57	1	H	-9.162128	4.157054	4.521191
58	1	H	-6.687669	5.578011	2.742727
59	1	H	-9.185845	-0.257734	3.76059
60	1	H	-8.728481	-1.586784	0.544202
61	1	H	-7.696034	-1.8279	-7.901993
62	1	H	-10.641425	1.851846	-11.284018
63	1	H	-9.35196	-1.057583	-12.319032
64	1	H	-4.991939	0.134184	-11.276828
65	1	H	-4.138634	1.237971	-8.237977
66	1	H	-5.989575	3.195073	-10.272346
67	1	H	0.739697	2.293652	-3.405627
68	1	H	-1.360544	3.762504	-5.614604
69	1	H	-0.520084	5.357089	-2.760267
70	1	H	-13.953611	-1.579433	-11.354718
71	1	H	-12.142962	-3.389036	-9.17657
72	1	H	-13.486812	-0.447403	-8.2355

2*R*,4*S*,5*S*,17*S*,19*S*,20*R*,23*R*,24*S*,*M*-**4** (2)			Standard Orientation (A.U.)		
Center number	Atomic number	Atomic Type	X	Y	Z
1	6	C	-0.333788	-0.055636	4.20965
2	6	C	-1.485451	-0.542205	1.512452
3	6	C	0.60881	-1.925649	0.005205
4	8	O	0.296464	-4.427635	0.302046
5	6	C	-1.769548	-4.958434	2.067805
6	6	C	-0.577211	-4.856079	4.731608
7	6	C	-0.941672	-2.259053	5.958399
8	6	C	-3.4982	-2.655589	1.6581
9	6	C	-1.405094	-6.619825	6.871994
10	6	C	-0.329677	-5.302271	9.271793
11	6	C	0.45802	-2.559993	8.477091
12	6	C	-2.288749	1.975994	0.300524
13	6	C	-3.202171	2.225281	-2.510733
14	6	C	-4.152179	-0.275488	-3.587878
15	8	O	-2.08841	3.922197	1.482426
16	6	C	-2.907258	-7.471843	1.314733
17	8	O	2.355118	-0.999811	-1.101349
18	1	H	-2.9555	-2.117402	6.484005
19	6	C	-0.202221	-0.55455	10.439573
20	8	O	3.08692	-2.448475	7.895069
21	1	H	1.463759	-5.134975	4.448056
22	8	O	-6.681167	-0.691107	-3.709943
23	6	C	-8.546414	1.265252	-3.675789
24	6	C	-7.864574	3.459065	-1.931874
25	6	C	-5.20258	4.335589	-2.683994
26	8	O	-2.765227	-1.949875	-4.254797
27	6	C	-8.196994	2.945895	0.871958
28	6	C	-7.921715	5.226714	2.552304
29	6	C	-8.759286	0.663116	1.813896
30	6	C	-9.140382	2.24224	-6.360392
31	6	C	-7.757079	1.175399	-8.650489
32	8	O	-10.764594	3.842256	-6.563878
33	6	C	-9.335823	-0.987944	-9.829396
34	6	C	-7.281417	3.283667	-10.574347
35	1	H	-10.286701	0.338218	-3.020476
36	6	C	-0.844519	3.046999	-4.044235
37	6	C	-9.756975	-3.290204	-8.138291
38	1	H	-1.111664	1.720018	4.943242
39	1	H	1.720365	0.205135	4.047252
40	1	H	-4.558818	-2.892152	-0.096401
41	1	H	-4.863569	-2.378862	3.186609

42	1	H	-0.711171	-8.564688	6.648148
43	1	H	-3.484432	-6.711923	6.971985
44	1	H	-1.728183	-5.265877	10.810069
45	1	H	1.348892	-6.295787	9.992688
46	1	H	-3.495069	-7.409859	-0.67771
47	1	H	-1.522632	-9.004138	1.54982
48	1	H	-4.565265	-7.897924	2.494504
49	1	H	0.777585	-0.93451	12.240806
50	1	H	-2.245642	-0.517688	10.829823
51	1	H	0.381816	1.326903	9.773635
52	1	H	4.027448	-2.68637	9.440996
53	1	H	-9.19511	4.985515	-2.422413
54	1	H	-4.561441	5.932328	-1.529888
55	1	H	-5.266614	5.011135	-4.652578
56	1	H	-9.103731	6.799029	1.861967
57	1	H	-8.476019	4.795703	4.507129
58	1	H	-5.950914	5.895595	2.577195
59	1	H	-9.054966	0.401681	3.841871
60	1	H	-8.942133	-1.016106	0.632894
61	1	H	-5.943854	0.36047	-8.047165
62	1	H	-11.166401	-0.191248	-10.433824
63	1	H	-8.349101	-1.579505	-11.567254
64	1	H	-6.323657	2.52358	-12.256186
65	1	H	-6.083342	4.786014	-9.774359
66	1	H	-9.075792	4.152823	-11.158538
67	1	H	0.684549	1.663775	-3.884179
68	1	H	-1.365196	3.264904	-6.045826
69	1	H	-0.179759	4.877395	-3.321889
70	1	H	-10.741867	-4.792248	-9.188713
71	1	H	-7.956649	-4.054164	-7.433248
72	1	H	-10.928393	-2.836331	-6.477316

2*R*,4*S*,5*S*,17*S*,19*S*,20*R*,23*R*,24*S*,*M*-**4** (3)		Standard Orientation (A.U.)			
Center number	Atomic number	Atomic Type	X	Y	Z
1	6	C	-1.017255	-0.59433	4.53546
2	6	C	-1.97209	-1.044433	1.757258
3	6	C	0.236497	-2.387212	0.378657
4	8	O	-0.079235	-4.89314	0.593735
5	6	C	-2.252625	-5.472851	2.212554
6	6	C	-1.236466	-5.408925	4.949398
7	6	C	-1.695215	-2.8421	6.202427
8	6	C	-3.970687	-3.175746	1.731624
9	6	C	-2.180998	-7.219328	7.000153
10	6	C	-1.245256	-5.951386	9.481762
11	6	C	-0.444954	-3.198342	8.80892
12	6	C	-2.703072	1.492254	0.533056
13	6	C	-3.404831	1.785136	-2.332679
14	6	C	-4.249899	-0.708011	-3.511575
15	8	O	-2.59821	3.41663	1.762854
16	6	C	-3.315686	-7.980755	1.342987
17	8	O	2.056685	-1.421142	-0.564254
18	1	H	-3.739431	-2.718349	6.600655
19	6	C	-1.242377	-1.239713	10.767499
20	8	O	2.24337	-3.236209	8.582725
21	1	H	0.821902	-5.668782	4.797971
22	8	O	-6.760318	-1.146378	-3.824114
23	6	C	-8.650662	0.797668	-3.859319
24	6	C	-8.1031	2.980516	-2.073136
25	6	C	-5.402193	3.881194	-2.628469
26	8	O	-2.800349	-2.362692	-4.084791
27	6	C	-8.633467	2.459543	0.698203
28	6	C	-8.504222	4.73732	2.399447
29	6	C	-9.229353	0.166937	1.596081
30	6	C	-9.150844	1.680694	-6.592461
31	6	C	-8.515169	-0.11975	-8.747463
32	8	O	-10.087341	3.737371	-6.955563
33	6	C	-10.586702	-0.045527	-10.776627
34	6	C	-5.922202	0.635477	-9.834985
35	1	H	-10.408095	-0.174003	-3.300799
36	6	C	-0.943727	2.649957	-3.66818
37	6	C	-13.145961	-1.004013	-9.845744
38	1	H	-1.870349	1.156471	5.245536
39	1	H	1.040123	-0.282463	4.475474
40	1	H	-4.912965	-3.382536	-0.092946
41	1	H	-5.435052	-2.939569	3.173025

42	1	H	-1.465353	-9.156136	6.775892
43	1	H	-4.262566	-7.319816	6.973568
44	1	H	-2.718951	-5.95863	10.946651
45	1	H	0.418135	-6.917938	10.261825
46	1	H	-3.776354	-7.887262	-0.681401
47	1	H	-1.934324	-9.505131	1.639388
48	1	H	-5.040097	-8.440762	2.409115
49	1	H	-0.36992	-1.666398	12.606888
50	1	H	-3.309129	-1.201698	11.004987
51	1	H	-0.635589	0.670989	10.194488
52	1	H	2.809592	-1.548574	8.172818
53	1	H	-9.38664	4.508834	-2.669017
54	1	H	-4.860251	5.472244	-1.417047
55	1	H	-5.341463	4.574869	-4.588229
56	1	H	-9.1744	4.290965	4.314149
57	1	H	-6.549707	5.436891	2.550314
58	1	H	-9.663902	6.293965	1.639182
59	1	H	-9.663093	-0.104791	3.597736
60	1	H	-9.303195	-1.512548	0.403275
61	1	H	-8.353945	-2.042756	-7.970028
62	1	H	-10.770218	1.913748	-11.457835
63	1	H	-9.945455	-1.191428	-12.395039
64	1	H	-5.969131	2.59734	-10.527329
65	1	H	-5.434833	-0.607994	-11.429143
66	1	H	-4.396088	0.463223	-8.435297
67	1	H	-0.343473	4.463669	-2.853616
68	1	H	0.577372	1.268499	-3.435423
69	1	H	-1.320515	2.921647	-5.694809
70	1	H	-13.880668	0.179855	-8.298082
71	1	H	-14.562259	-0.973382	-11.369568
72	1	H	-13.013953	-2.963101	-9.146953

2*R*,4*S*,5*S*,17*S*,19*S*,20*R*,23*R*,24*S*,*M*-**4** (4)		Standard Orientation (A.U.)			
Center number	Atomic number	Atomic Type	X	Y	Z
1	6	C	-0.775163	-0.751319	4.167102
2	6	C	-1.889534	-1.199721	1.447978
3	6	C	0.220333	-2.572798	-0.047683
4	8	O	-0.106852	-5.077299	0.209426
5	6	C	-2.197427	-5.621987	1.940769
6	6	C	-1.039103	-5.558848	4.620832
7	6	C	-1.410511	-2.977052	5.877769
8	6	C	-3.911897	-3.30695	1.539468
9	6	C	-1.900326	-7.348084	6.726457
10	6	C	-0.849626	-6.067609	9.156831
11	6	C	-0.043516	-3.317226	8.409446
12	6	C	-2.65624	1.338873	0.2527
13	6	C	-3.532855	1.626943	-2.566631
14	6	C	-4.49378	-0.853102	-3.679482
15	8	O	-2.443828	3.270804	1.455436
16	6	C	-3.33489	-8.120778	1.1401
17	8	O	1.985803	-1.639924	-1.11712
18	1	H	-3.430308	-2.836063	6.380282
19	6	C	-0.722112	-1.336872	10.391052
20	8	O	2.59291	-3.206815	7.862139
21	1	H	1.004351	-5.841222	4.359553
22	8	O	-7.028077	-1.252535	-3.817275
23	6	C	-8.88916	0.716492	-3.723501
24	6	C	-8.182327	2.904105	-1.999021
25	6	C	-5.511329	3.753673	-2.747492
26	8	O	-3.115042	-2.531789	-4.34936
27	6	C	-8.53062	2.414062	0.806473
28	6	C	-8.248052	4.702604	2.474091
29	6	C	-9.102841	0.139273	1.763008
30	6	C	-9.586532	1.590942	-6.415364
31	6	C	-9.116086	-0.217667	-8.605643
32	8	O	-10.540617	3.649858	-6.715098
33	6	C	-11.314177	-0.119875	-10.49549
34	6	C	-6.58988	0.503371	-9.861286
35	1	H	-10.612403	-0.225784	-3.025847
36	6	C	-1.14699	2.444494	-4.058689
37	6	C	-13.817402	-1.054428	-9.402035
38	1	H	-1.561468	1.015423	4.913366
39	1	H	1.281191	-0.490339	4.036573
40	1	H	-4.951398	-3.517347	-0.230971
41	1	H	-5.294772	-3.047188	3.055209

42	1	H	-1.211537	-9.292613	6.484583
43	1	H	-3.981111	-7.432779	6.799214
44	1	H	-2.2661	-6.047849	10.678888
45	1	H	0.817098	-7.07677	9.883529
46	1	H	-3.894903	-8.030341	-0.859247
47	1	H	-1.959851	-9.661827	1.37407
48	1	H	-5.010534	-8.555026	2.29174
49	1	H	0.233954	-1.744609	12.198992
50	1	H	-2.770148	-1.299194	10.756254
51	1	H	-0.124488	0.551824	9.758569
52	1	H	3.512737	-3.469677	9.416435
53	1	H	-9.478935	4.449418	-2.517852
54	1	H	-4.860989	5.34533	-1.591462
55	1	H	-5.572861	4.427067	-4.714629
56	1	H	-8.815035	4.289145	4.429095
57	1	H	-6.271625	5.354594	2.50405
58	1	H	-9.412726	6.280072	1.766469
59	1	H	-9.404997	-0.109158	3.791682
60	1	H	-9.285917	-1.548001	0.593165
61	1	H	-8.927638	-2.141892	-7.837182
62	1	H	-11.521996	1.841767	-11.162731
63	1	H	-10.792418	-1.270402	-12.153254
64	1	H	-6.656716	2.464454	-10.554264
65	1	H	-6.223595	-0.749269	-11.480374
66	1	H	-4.9785	0.314204	-8.563399
67	1	H	-0.467965	4.25287	-3.29556
68	1	H	0.363056	1.039989	-3.907554
69	1	H	-1.642829	2.708836	-6.060517
70	1	H	-14.443271	0.137771	-7.813599
71	1	H	-15.327012	-1.01428	-10.833341
72	1	H	-13.658404	-3.013484	-8.708588

2*R*,4*S*,5*S*,17*S*,19*S*,20*R*,23*R*,24*S*,*M*-**4** (5)		Standard Orientation (A.U.)			
Center number	Atomic number	Atomic Type	X	Y	Z
1	6	C	-2.550867	-4.721957	0.30569
2	6	C	-1.789359	-1.858821	0.624286
3	6	C	-3.280862	-0.78884	2.89591
4	8	O	-1.858001	-1.014601	4.990326
5	6	C	0.456352	-2.444864	4.428957
6	6	C	-0.304071	-5.264273	4.568026
7	6	C	-0.856477	-6.367247	1.953684
8	6	C	0.916532	-1.643967	1.681138
9	6	C	1.446539	-7.289776	5.673655
10	6	C	0.258046	-9.798933	4.707754
11	6	C	-1.659703	-9.094723	2.589453
12	6	C	-2.06213	-0.388755	-1.867153
13	6	C	-4.553246	-0.201823	-3.452601
14	6	C	-6.997024	-0.541143	-1.929025
15	8	O	-0.159255	0.459505	-2.814637
16	6	C	2.446422	-1.628334	6.311734
17	8	O	-5.369937	0.085839	2.923146
18	1	H	0.977583	-6.545116	0.976069
19	6	C	-1.61793	-10.880598	0.327461
20	8	O	-4.095546	-9.120922	3.744825
21	1	H	-2.075273	-5.325602	5.657047
22	8	O	-8.356977	1.51408	-1.313454
23	6	C	-8.024623	4.010673	-2.315136
24	6	C	-5.393005	4.574362	-3.329661
25	6	C	-4.561936	2.307185	-4.931714
26	8	O	-7.798353	-2.593852	-1.347896
27	6	C	-3.455261	5.413262	-1.38437
28	6	C	-1.044276	6.466917	-2.473867
29	6	C	-3.834501	5.279906	1.115258
30	6	C	-10.129777	4.472133	-4.29067
31	6	C	-12.766256	3.417344	-3.778717
32	8	O	-9.694924	5.729964	-6.153829
33	6	C	-13.008297	0.57847	-4.431877
34	6	C	-13.673215	3.954931	-1.077221
35	1	H	-8.461709	5.281817	-0.725609
36	6	C	-4.423526	-2.388151	-5.400956
37	6	C	-11.91767	-0.152782	-7.001005
38	1	H	-2.321058	-5.278806	-1.678398
39	1	H	-4.56545	-4.928562	0.759196
40	1	H	1.556781	0.328276	1.598903
41	1	H	2.307193	-2.807317	0.68028

42	1	H	1.576444	-7.21536	7.744923
43	1	H	3.376578	-7.063723	4.92165
44	1	H	1.707258	-11.104126	3.991585
45	1	H	-0.789718	-10.784544	6.204407
46	1	H	2.723888	0.429546	6.224411
47	1	H	1.881524	-2.138036	8.245713
48	1	H	4.254534	-2.56224	5.890128
49	1	H	-3.008987	-10.287081	-1.107624
50	1	H	-2.103878	-12.811911	0.927032
51	1	H	0.254837	-10.914906	-0.578597
52	1	H	-5.338285	-8.645163	2.492201
53	1	H	-5.657533	6.159662	-4.654286
54	1	H	-2.680254	2.600242	-5.747857
55	1	H	-5.887015	2.122751	-6.522318
56	1	H	0.147019	7.306063	-0.993201
57	1	H	0.062753	4.972777	-3.410041
58	1	H	-1.447802	7.930388	-3.903269
59	1	H	-2.396437	5.956643	2.43655
60	1	H	-5.524651	4.45364	1.951081
61	1	H	-13.957056	4.470072	-5.125973
62	1	H	-12.127827	-0.563584	-2.935582
63	1	H	-15.044299	0.128863	-4.390644
64	1	H	-13.567679	5.982967	-0.617974
65	1	H	-12.554261	2.89616	0.316761
66	1	H	-15.659566	3.368995	-0.875177
67	1	H	-4.617233	-4.237414	-4.495608
68	1	H	-5.982693	-2.194727	-6.760848
69	1	H	-2.626072	-2.289673	-6.436499
70	1	H	-12.307946	-2.1503	-7.430744
71	1	H	-12.710567	1.009194	-8.536145
72	1	H	-9.851195	0.09639	-7.060889

2*R*,4*S*,5*S*,17*S*,19*S*,20*R*,23*R*,24*S*,*M*-**4** (6)		Standard Orientation (A.U.)			
Center number	Atomic number	Atomic Type	X	Y	Z
1	6	C	-0.995067	-1.478319	2.563695
2	6	C	-2.896376	-3.464415	1.472858
3	6	C	-1.366736	-5.940025	1.084133
4	8	O	-1.527755	-7.345762	3.20408
5	6	C	-2.947702	-5.992543	5.162235
6	6	C	-0.996642	-4.277196	6.511268
7	6	C	-1.016758	-1.589796	5.438876
8	6	C	-4.739608	-4.389362	3.54204
9	6	C	-1.085585	-3.745522	9.355627
10	6	C	0.699946	-1.431371	9.653248
11	6	C	1.073995	-0.250668	6.96008
12	6	C	-4.321974	-2.924444	-1.018306
13	6	C	-3.542314	-0.860934	-2.954855
14	6	C	-5.351049	-1.299132	-5.199569
15	8	O	-6.004845	-4.369315	-1.579927
16	6	C	-4.19742	-7.948167	6.832172
17	8	O	-0.15726	-6.553027	-0.726966
18	1	H	-2.799958	-0.727332	6.08646
19	6	C	0.866061	2.616294	6.921265
20	8	O	3.512683	-0.803334	5.960919
21	1	H	0.848546	-5.162748	6.097124
22	8	O	-7.672352	-0.20935	-5.041482
23	6	C	-8.09271	2.221962	-3.940884
24	6	C	-6.532981	2.744903	-1.566314
25	6	C	-3.799954	1.926803	-2.108592
26	8	O	-4.839716	-2.568784	-7.004585
27	6	C	-7.594325	1.693218	0.883364
28	6	C	-6.636194	2.979119	3.238432
29	6	C	-9.293975	-0.178151	0.978814
30	6	C	-7.692004	4.340356	-5.927116
31	6	C	-7.379018	3.585543	-8.676269
32	8	O	-7.67749	6.514105	-5.209191
33	6	C	-9.81782	2.308932	-9.691246
34	6	C	-6.557736	5.838547	-10.280582
35	1	H	-10.117978	2.26224	-3.468518
36	6	C	-0.816488	-1.395802	-3.820726
37	6	C	-9.275094	0.446219	-11.828514
38	1	H	-1.497315	0.42931	1.95802
39	1	H	0.922558	-1.842087	1.851288
40	1	H	-6.19589	-5.58204	2.673649
41	1	H	-5.669716	-2.863987	4.588022

42	1	H	-0.501788	-5.366203	10.515979
43	1	H	-3.026331	-3.245483	9.923632
44	1	H	-0.073484	-0.037573	10.986555
45	1	H	2.566787	-1.983382	10.383561
46	1	H	-5.393568	-9.208185	5.692446
47	1	H	-2.772128	-9.098843	7.814426
48	1	H	-5.383998	-7.015419	8.261137
49	1	H	2.351727	3.458353	8.107275
50	1	H	-0.987704	3.237523	7.628564
51	1	H	1.120622	3.331784	4.984728
52	1	H	3.77286	-2.6099	5.996147
53	1	H	-6.533674	4.818578	-1.383322
54	1	H	-2.566018	2.343136	-0.501722
55	1	H	-3.065545	3.09446	-3.672752
56	1	H	-4.557693	2.914974	3.366596
57	1	H	-7.156601	4.998507	3.235039
58	1	H	-7.409946	2.111445	4.960422
59	1	H	-10.035227	-0.848395	2.787018
60	1	H	-9.958276	-1.149834	-0.714229
61	1	H	-5.891786	2.124914	-8.719345
62	1	H	-10.780183	1.280154	-8.163783
63	1	H	-11.124115	3.810088	-10.316342
64	1	H	-6.354521	5.282435	-12.272943
65	1	H	-4.738021	6.60943	-9.634196
66	1	H	-7.957507	7.370724	-10.159618
67	1	H	-0.572717	-3.383464	-4.34729
68	1	H	-0.371289	-0.212241	-5.46982
69	1	H	0.53564	-0.949401	-2.312646
70	1	H	-11.036255	-0.367081	-12.581924
71	1	H	-8.275599	1.35617	-13.412569
72	1	H	-8.088448	-1.111493	-11.123204

TABLE S8 Gibbs free energies[a] and equilibrium populations[b] of low-energy conformers of 2*R*,4*S*,5*S*,17*S*,19*S*,20*R*,23*R*,24*S*,*P*-**4**.

Conformers	ΔG (a.u.)	*P*(%)/100	G (a.u.)
2*R*,4*S*,5*S*,17*S*,19*S*,20*R*,23*R*,24*S*,*P*-**4** (1)_tddft_	0.0013	13.5	-1577.840975
2*R*,4*S*,5*S*,17*S*,19*S*,20*R*,23*R*,24*S*,*P*-**4** (2)_tddft_	0.0	53.67	-1577.842278
2*R*,4*S*,5*S*,17*S*,19*S*,20*R*,23*R*,24*S*,*P*-**4** (3)_tddft_	0.01001	0.0	-1577.83227
2*R*,4*S*,5*S*,17*S*,19*S*,20*R*,23*R*,24*S*,*P*-**4** (4)_tddft_	0.0068	0.04	-1577.835476
2*R*,4*S*,5*S*,17*S*,19*S*,20*R*,23*R*,24*S*,*P*-**4** (5)_tddft_	0.00103	18.05	-1577.841249
2*R*,4*S*,5*S*,17*S*,19*S*,20*R*,23*R*,24*S*,*P*-**4** (6)_tddft_	0.00126	14.08	-1577.841015
2*R*,4*S*,5*S*,17*S*,19*S*,20*R*,23*R*,24*S*,*P*-**4** (7)_tddft_	0.01024	0.0	-1577.832035
2*R*,4*S*,5*S*,17*S*,19*S*,20*R*,23*R*,24*S*,*P*-**4** (8)_tddft_	0.00584	0.11	-1577.836434
2*R*,4*S*,5*S*,17*S*,19*S*,20*R*,23*R*,24*S*,*P*-**4** (9)_tddft_	0.00433	0.55	-1577.837949

[a]wB97M-V/def2-TZVP, in a.u.

[b]From ΔG values at 298.15 K.

TABLE S9 Cartesian coordinates for the low-energy reoptimized random research conformers of 2*R*,4*S*,5*S*,17*S*,19*S*,20*R*,23*R*,24*S*,*P*-**4** at B3LYP-D3(BJ)/6-31G* level of theory in methanol.

2*R*,4*S*,5*S*,17*S*,19*S*,20*R*,23*R*,24*S*,*P*-**4** (1)		Standard Orientation (A.U.)			
Center number	Atomic number	Atomic Type	X	Y	Z
1	6	C	-0.91435	0.984372	4.589557
2	6	C	-3.606105	0.103936	3.609122
3	6	C	-3.281213	-2.524259	2.444706
4	8	O	-3.635726	-4.273246	4.245714
5	6	C	-4.108324	-3.051886	6.709867
6	6	C	-1.477836	-2.521771	7.8606
7	6	C	-0.597171	0.187559	7.334785
8	6	C	-5.342849	-0.574849	5.855666
9	6	C	-0.977628	-2.74614	10.705579
10	6	C	1.53441	-1.261094	11.069788
11	6	C	2.030053	0.226946	8.561294
12	6	C	-4.777427	2.189076	1.973065
13	6	C	-4.622915	2.209802	-0.940446
14	6	C	-1.985151	1.46464	-1.863073
15	8	O	-5.937905	3.837701	3.054529
16	6	C	-5.764724	-4.82318	8.223304
17	8	O	-2.777157	-3.072594	0.29279
18	1	H	-1.764868	1.450413	8.513906
19	6	C	3.100241	2.869407	8.97532
20	8	O	3.639529	-1.180233	6.921059
21	1	H	-0.16223	-3.80729	6.891359
22	8	O	-1.788306	0.230376	-4.078898
23	6	C	-3.749934	-1.372086	-5.042106
24	6	C	-6.375281	-0.15716	-4.749287
25	6	C	-6.776441	0.508963	-1.95362
26	8	O	-0.109393	2.250591	-0.841534
27	6	C	-8.487015	-1.803801	-5.776927
28	6	C	-8.582501	-4.519631	-4.930129
29	6	C	-10.17724	-0.824283	-7.386661
30	6	C	-3.183589	-1.883973	-7.826057
31	6	C	-2.169935	0.287316	-9.417588
32	8	O	-3.614511	-3.953479	-8.705313
33	6	C	-3.542716	0.351836	-11.978315
34	6	C	0.712897	-0.027688	-9.646001
35	1	H	-3.659421	-3.166249	-4.00858
36	6	C	-5.003431	4.956761	-1.85484
37	6	C	-2.808059	2.584292	-13.652317
38	1	H	-0.762465	3.043949	4.392147
39	1	H	0.564115	0.161597	3.393734

40	1	H	-7.281809	-0.922992	5.188737
41	1	H	-5.42531	0.885157	7.317559
42	1	H	-0.847625	-4.713444	11.358414
43	1	H	-2.51101	-1.836429	11.783044
44	1	H	1.418566	0.030849	12.69464
45	1	H	3.135847	-2.540571	11.418304
46	1	H	-4.768007	-6.604198	8.616303
47	1	H	-6.278527	-3.946421	10.035907
48	1	H	-7.50704	-5.259976	7.177262
49	1	H	4.933109	2.775624	9.965764
50	1	H	1.819494	4.033742	10.129726
51	1	H	3.403483	3.821289	7.151412
52	1	H	5.320173	-1.19046	7.632532
53	1	H	-6.367064	1.613734	-5.842061
54	1	H	-6.899494	-1.246255	-0.855521
55	1	H	-8.583116	1.510765	-1.72209
56	1	H	-10.379829	-5.410426	-5.468782
57	1	H	-7.034094	-5.590574	-5.81966
58	1	H	-8.349273	-4.703587	-2.868789
59	1	H	-11.74424	-1.952496	-8.122043
60	1	H	-10.046651	1.136615	-8.028317
61	1	H	-2.532684	2.067461	-8.395638
62	1	H	-3.186612	-1.450602	-12.95813
63	1	H	-5.596887	0.3994	-11.619777
64	1	H	1.595836	-0.090049	-7.766266
65	1	H	1.165006	-1.794632	-10.648912
66	1	H	1.550766	1.558697	-10.695324
67	1	H	-3.483968	6.171613	-1.133018
68	1	H	-4.985984	5.051703	-3.929341
69	1	H	-6.813935	5.681459	-1.150159
70	1	H	-3.116544	4.40061	-12.676195
71	1	H	-0.807399	2.511833	-14.219041
72	1	H	-3.943475	2.609451	-15.396332

2*R*,4*S*,5*S*,17*S*,19*S*,20*R*,23*R*,24*S*,*P*-**4** (2)		Standard Orientation (A.U.)			
Center number	Atomic number	Atomic Type	X	Y	Z
1	6	C	-1.49577	1.18221	4.75512
2	6	C	-3.984775	0.026861	3.557288
3	6	C	-3.293075	-2.552057	2.434417
4	8	O	-3.637562	-4.338032	4.193967
5	6	C	-4.413479	-3.179071	6.6153
6	6	C	-1.940511	-2.400153	7.957785
7	6	C	-1.291968	0.384694	7.510582
8	6	C	-5.817597	-0.833025	5.658238
9	6	C	-1.617631	-2.603667	10.828142
10	6	C	0.754385	-0.948921	11.344559
11	6	C	1.242762	0.657853	8.926133
12	6	C	-5.21985	1.975398	1.801935
13	6	C	-4.743479	2.052831	-1.075396
14	6	C	-1.965123	1.589237	-1.713882
15	8	O	-6.694056	3.450028	2.742209
16	6	C	-5.997156	-5.111193	8.003377
17	8	O	-2.537227	-3.026597	0.337839
18	1	H	-2.6611	1.518064	8.601993
19	6	C	1.990237	3.38674	9.471206
20	8	O	3.216939	-0.595825	7.582043
21	1	H	-0.430746	-3.537301	7.088044
22	8	O	-1.416937	0.412638	-3.895837
23	6	C	-3.075754	-1.363834	-5.094099
24	6	C	-5.849209	-0.482029	-5.045327
25	6	C	-6.587876	0.149007	-2.309135
26	8	O	-0.287681	2.542577	-0.502147
27	6	C	-7.669159	-2.400468	-6.165075
28	6	C	-7.409639	-5.115073	-5.348353
29	6	C	-9.458436	-1.638861	-7.785205
30	6	C	-2.1746	-1.715164	-7.815778
31	6	C	-0.881173	0.49875	-9.127079
32	8	O	-2.546779	-3.714945	-8.866901
33	6	C	-1.61886	0.611732	-11.926667
34	6	C	1.992089	0.212565	-8.757245
35	1	H	-2.880852	-3.167099	-4.089825
36	6	C	-5.306261	4.753838	-2.030258
37	6	C	-4.410449	1.212037	-12.355408
38	1	H	-1.553491	3.248614	4.5771
39	1	H	0.130329	0.537893	3.640008
40	1	H	-7.65209	-1.367723	4.837829
41	1	H	-6.163684	0.60213	7.106922

42	1	H	-1.385027	-4.558128	11.490731
43	1	H	-3.282037	-1.810563	11.799105
44	1	H	0.48901	0.269048	13.006615
45	1	H	2.444091	-2.107282	11.679704
46	1	H	-7.599178	-5.718291	6.826285
47	1	H	-4.860314	-6.782826	8.48495
48	1	H	-6.738583	-4.294439	9.764706
49	1	H	0.503725	4.381058	10.534231
50	1	H	2.313111	4.436267	7.698852
51	1	H	3.749842	3.441977	10.578864
52	1	H	3.611528	0.382404	6.089933
53	1	H	-5.978758	1.259387	-6.178901
54	1	H	-6.652908	-1.613384	-1.218387
55	1	H	-8.500947	0.960098	-2.279068
56	1	H	-5.723766	-5.955901	-6.233386
57	1	H	-7.17439	-5.293347	-3.286526
58	1	H	-9.068142	-6.228878	-5.916433
59	1	H	-10.843856	-2.96258	-8.558174
60	1	H	-9.593751	0.329237	-8.400583
61	1	H	-1.47768	2.253758	-8.175079
62	1	H	-0.428913	2.048809	-12.856577
63	1	H	-1.148428	-1.219303	-12.799543
64	1	H	2.987621	1.807025	-9.64933
65	1	H	2.490458	0.175565	-6.741687
66	1	H	2.658798	-1.548644	-9.643303
67	1	H	-5.092732	4.862224	-4.093339
68	1	H	-7.242175	5.286103	-1.512006
69	1	H	-3.996697	6.110887	-1.164167
70	1	H	-4.859004	1.319387	-14.384533
71	1	H	-5.63362	-0.248777	-11.519743
72	1	H	-4.927408	3.042694	-11.500853

2*R*,4*S*,5*S*,17*S*,19*S*,20*R*,23*R*,24*S*,*P*-**4** (3)		Standard Orientation (A.U.)			
Center number	Atomic number	Atomic Type	X	Y	Z
1	6	C	-1.562378	1.348008	2.709519
2	6	C	-3.920916	-0.418869	2.709726
3	6	C	-3.075897	-3.08457	1.839991
4	8	O	-2.477676	-4.481802	3.860186
5	6	C	-2.596032	-2.926363	6.16459
6	6	C	-0.023318	-1.545516	6.302292
7	6	C	-0.192677	1.139094	5.237585
8	6	C	-4.717496	-1.07314	5.449038
9	6	C	1.358596	-1.07864	8.805687
10	6	C	3.305765	1.018487	8.118616
11	6	C	2.538246	2.072354	5.462667
12	6	C	-6.227873	0.300758	1.08733
13	6	C	-6.493348	2.83232	-0.371672
14	6	C	-4.078513	3.89787	-1.560269
15	8	O	-8.012713	-1.13015	1.14584
16	6	C	-3.171081	-4.67271	8.352281
17	8	O	-2.922092	-3.846061	-0.295698
18	1	H	-1.26757	2.265337	6.62658
19	6	C	2.827224	4.926748	5.202288
20	8	O	3.907165	0.817856	3.506958
21	1	H	1.265326	-2.626654	5.080579
22	8	O	-3.14395	2.572295	-3.510982
23	6	C	-4.564352	0.46242	-4.523681
24	6	C	-7.302145	1.277855	-4.974811
25	6	C	-8.425792	2.498501	-2.547494
26	8	O	-3.079792	5.84334	-0.926595
27	6	C	-8.929281	-0.852311	-6.005832
28	6	C	-9.022906	-3.295174	-4.54614
29	6	C	-10.228291	-0.520139	-8.151801
30	6	C	-3.154645	-0.142966	-7.016645
31	6	C	-0.953626	-1.982167	-6.914826
32	8	O	-3.822444	0.858605	-8.957729
33	6	C	-1.989454	-4.699431	-6.606992
34	6	C	0.994071	-1.280192	-4.880098
35	1	H	-4.402902	-1.131861	-3.202061
36	6	C	-7.499255	4.706208	1.619228
37	6	C	0.027704	-6.74075	-6.934246
38	1	H	-2.131568	3.313819	2.428856
39	1	H	-0.291896	0.853375	1.142115
40	1	H	-6.54646	-2.05243	5.428605
41	1	H	-4.841391	0.570196	6.706796

42	1	H	2.258579	-2.786372	9.571669
43	1	H	0.0285	-0.381121	10.249356
44	1	H	3.324699	2.529005	9.5468
45	1	H	5.236488	0.256318	8.003965
46	1	H	-4.923283	-5.731041	7.993891
47	1	H	-1.623405	-6.030233	8.636505
48	1	H	-3.402804	-3.571035	10.099232
49	1	H	2.234673	5.547251	3.30743
50	1	H	4.813902	5.496738	5.479873
51	1	H	1.68129	5.935081	6.616077
52	1	H	5.634554	1.405564	3.5448
53	1	H	-7.178246	2.73218	-6.450023
54	1	H	-10.000167	1.369947	-1.810811
55	1	H	-9.183927	4.373371	-3.021063
56	1	H	-7.17652	-4.259693	-4.580473
57	1	H	-9.47503	-2.985106	-2.54085
58	1	H	-10.425907	-4.594566	-5.357574
59	1	H	-11.446212	-2.007603	-8.91107
60	1	H	-10.094402	1.236102	-9.230438
61	1	H	-0.072058	-1.834276	-8.796595
62	1	H	-2.853234	-4.894876	-4.724802
63	1	H	-3.500417	-4.992169	-8.013626
64	1	H	1.565125	0.711674	-5.031486
65	1	H	0.241042	-1.602248	-2.971563
66	1	H	2.697608	-2.452442	-5.087861
67	1	H	-9.362153	4.079448	2.291722
68	1	H	-6.218767	4.859742	3.248756
69	1	H	-7.662867	6.598073	0.781662
70	1	H	1.467888	-6.643176	-5.436952
71	1	H	-0.825214	-8.636975	-6.849542
72	1	H	0.999814	-6.560921	-8.768634

2*R*,4*S*,5*S*,17*S*,19*S*,20*R*,23*R*,24*S*,*P*-**4** (4)_		Standard Orientation (A.U.)			
Center number	Atomic number	Atomic Type	X	Y	Z
1	6	C	-1.749596	1.102099	3.996403
2	6	C	-4.272472	-0.077446	2.913762
3	6	C	-3.740594	-2.852366	2.227476
4	8	O	-4.314639	-4.312117	4.223989
5	6	C	-5.018901	-2.730299	6.403899
6	6	C	-2.505307	-1.945422	7.672558
7	6	C	-1.639917	0.68958	6.843446
8	6	C	-6.211283	-0.463554	5.054141
9	6	C	-2.224704	-1.752789	10.548371
10	6	C	0.259763	-0.218265	10.872012
11	6	C	0.894235	0.974519	8.256677
12	6	C	-5.364937	1.584222	0.815753
13	6	C	-4.208318	1.955148	-1.872861
14	6	C	-1.807428	0.417735	-2.416794
15	8	O	-7.195866	2.862735	1.320897
16	6	C	-6.780028	-4.277213	8.039622
17	8	O	-2.911609	-3.693601	0.290175
18	1	H	-2.930048	2.064623	7.734327
19	6	C	1.849496	3.689296	8.411304
20	8	O	2.77893	-0.607932	7.153963
21	1	H	-1.076611	-3.303823	7.008475
22	8	O	-1.783992	-1.229964	-4.351075
23	6	C	-3.860127	-1.721269	-5.998552
24	6	C	-6.461477	-1.139053	-4.86164
25	6	C	-6.320889	1.566019	-3.863325
26	8	O	0.131742	0.796762	-1.284005
27	6	C	-7.507399	-3.122615	-3.051662
28	6	C	-6.760602	-5.822867	-3.53424
29	6	C	-9.1477	-2.534167	-1.211738
30	6	C	-3.533016	-0.304733	-8.53013
31	6	C	-1.127828	1.204109	-8.983017
32	8	O	-5.172519	-0.525815	-10.112813
33	6	C	-1.482843	3.160262	-11.086174
34	6	C	1.024227	-0.673712	-9.558518
35	1	H	-3.69849	-3.735802	-6.475196
36	6	C	-3.381066	4.769471	-1.941734
37	6	C	-3.336692	5.269885	-10.411249
38	1	H	-1.69396	3.132113	3.556145
39	1	H	-0.142538	0.241802	3.010646
40	1	H	-8.056159	-0.978954	4.254741
41	1	H	-6.476099	1.196683	6.260188

42	1	H	-2.148731	-3.603285	11.488298
43	1	H	-3.83436	-0.708864	11.361055
44	1	H	0.069638	1.246957	12.332753
45	1	H	1.853086	-1.435967	11.408763
46	1	H	-7.454517	-3.136701	9.64035
47	1	H	-8.42177	-4.909644	6.933032
48	1	H	-5.804686	-5.951296	8.791392
49	1	H	2.27697	4.432493	6.510877
50	1	H	3.595933	3.772391	9.537759
51	1	H	0.435432	4.943355	9.28125
52	1	H	3.196628	0.052355	5.50189
53	1	H	-7.727057	-1.157577	-6.520194
54	1	H	-8.117026	2.215569	-3.0594
55	1	H	-5.936296	2.826876	-5.473483
56	1	H	-7.063158	-6.345255	-5.531109
57	1	H	-4.74961	-6.111076	-3.084753
58	1	H	-7.864867	-7.123553	-2.351336
59	1	H	-9.967582	-4.020921	-0.034744
60	1	H	-9.760994	-0.607796	-0.82216
61	1	H	-0.631582	2.178803	-7.210098
62	1	H	0.383155	3.978092	-11.52658
63	1	H	-2.131811	2.180136	-12.804909
64	1	H	2.805815	0.360322	-9.845732
65	1	H	1.305997	-2.006439	-7.990859
66	1	H	0.612913	-1.747032	-11.294165
67	1	H	-1.921783	5.14953	-0.518179
68	1	H	-2.595395	5.233916	-3.809511
69	1	H	-5.035195	5.969215	-1.583248
70	1	H	-2.744743	6.277038	-8.684331
71	1	H	-3.465565	6.676381	-11.939044
72	1	H	-5.25016	4.518007	-10.095879

2*R*,4*S*,5*S*,17*S*,19*S*,20*R*,23*R*,24*S*,*P*-**4** (5)		Standard Orientation (A.U.)			
Center number	Atomic number	Atomic Type	X	Y	Z
1	6	C	-0.801847	1.219792	4.654154
2	6	C	-3.552279	0.493083	3.718657
3	6	C	-3.476896	-2.257447	2.816312
4	8	O	-4.018182	-3.780925	4.763568
5	6	C	-4.365458	-2.28661	7.098152
6	6	C	-1.693825	-1.909488	8.213933
7	6	C	-0.554049	0.640914	7.45999
8	6	C	-5.353423	0.204921	5.995955
9	6	C	-1.205441	-1.932012	11.067915
10	6	C	1.462309	-0.720317	11.296438
11	6	C	2.080896	0.526974	8.703174
12	6	C	-4.500448	2.49576	1.851065
13	6	C	-4.213943	2.246401	-1.043455
14	6	C	-1.656841	1.119469	-1.790651
15	8	O	-5.59325	4.307486	2.720573
16	6	C	-6.185573	-3.735461	8.758218
17	8	O	-3.011282	-3.050367	0.732093
18	1	H	-1.593545	2.114998	8.506797
19	6	C	3.382122	3.088836	8.919928
20	8	O	3.67114	-1.214939	7.395922
21	1	H	-0.502575	-3.393218	7.373163
22	8	O	-1.542184	-0.344488	-3.864688
23	6	C	-3.652167	-1.781338	-4.776982
24	6	C	-6.112142	-0.230777	-4.714949
25	6	C	-6.518563	0.720537	-2.003784
26	8	O	0.274156	1.783299	-0.780234
27	6	C	-8.382862	-1.685015	-5.693086
28	6	C	-8.86657	-4.275701	-4.617936
29	6	C	-9.87503	-0.650576	-7.456738
30	6	C	-3.049606	-2.619163	-7.47317
31	6	C	-1.627897	-0.785787	-9.171729
32	8	O	-3.766788	-4.667074	-8.202602
33	6	C	-2.829381	-0.757432	-11.817189
34	6	C	1.179278	-1.550329	-9.171178
35	1	H	-3.826184	-3.467629	-3.583506
36	6	C	-4.232048	4.928159	-2.200461
37	6	C	-1.666741	1.179047	-13.613057
38	1	H	-0.466958	3.231916	4.27342
39	1	H	0.579018	0.167571	3.51867
40	1	H	-7.314627	-0.017569	5.341502
41	1	H	-5.299959	1.797584	7.314022

42	1	H	-1.290383	-3.833921	11.89766
43	1	H	-2.621622	-0.76151	12.050431
44	1	H	1.531074	0.682581	12.827205
45	1	H	2.927523	-2.139825	11.680172
46	1	H	-5.363611	-5.556186	9.330826
47	1	H	-6.614769	-2.642058	10.472595
48	1	H	-7.961172	-4.10287	7.74208
49	1	H	2.200166	4.454523	9.952591
50	1	H	3.769682	3.885262	7.032516
51	1	H	5.196424	2.89075	9.917493
52	1	H	4.125246	-0.484654	5.78371
53	1	H	-5.838688	1.419737	-5.952629
54	1	H	-6.868132	-0.910711	-0.771267
55	1	H	-8.203615	1.934295	-1.922566
56	1	H	-7.436625	-5.613418	-5.325451
57	1	H	-8.749359	-4.293302	-2.538451
58	1	H	-10.740928	-4.977962	-5.172387
59	1	H	-11.550232	-1.634088	-8.1605
60	1	H	-9.469257	1.210221	-8.260812
61	1	H	-1.76506	1.116625	-8.331589
62	1	H	-2.699632	-2.674687	-12.619041
63	1	H	-4.868738	-0.373276	-11.606384
64	1	H	2.314468	-0.201929	-10.272312
65	1	H	1.930847	-1.586825	-7.234642
66	1	H	1.408413	-3.443139	-10.006158
67	1	H	-5.963465	5.919011	-1.635395
68	1	H	-2.59873	6.014359	-1.523006
69	1	H	-4.138895	4.833408	-4.272713
70	1	H	-1.749215	3.102128	-12.812045
71	1	H	0.328447	0.759559	-14.027843
72	1	H	-2.682553	1.211486	-15.429066

2*R*,4*S*,5*S*,17*S*,19*S*,20*R*,23*R*,24*S*,*P*-**4** (6)		Standard Orientation (A.U.)			
Center number	Atomic number	Atomic Type	X	Y	Z
1	6	C	-1.401537	1.748167	4.489583
2	6	C	-3.809271	0.266227	3.498755
3	6	C	-2.89945	-2.276745	2.452929
4	8	O	-2.934458	-3.993992	4.311629
5	6	C	-3.698267	-2.803155	6.721017
6	6	C	-1.259452	-1.686346	7.872113
7	6	C	-0.957643	1.124702	7.260599
8	6	C	-5.41139	-0.678313	5.746865
9	6	C	-0.757936	-1.70386	10.724106
10	6	C	1.428356	0.237138	11.024335
11	6	C	1.603603	1.751604	8.505603
12	6	C	-5.348015	1.975883	1.734752
13	6	C	-5.016947	1.986314	-1.165026
14	6	C	-2.242085	1.757145	-1.933924
15	8	O	-6.944867	3.305764	2.690827
16	6	C	-4.970986	-4.825056	8.290043
17	8	O	-2.221426	-2.777211	0.336165
18	1	H	-2.383596	2.156472	8.379281
19	6	C	2.067894	4.572693	8.86516
20	8	O	3.631492	0.649576	7.109384
21	1	H	0.320013	-2.691746	6.964549
22	8	O	-1.686843	0.518556	-4.077905
23	6	C	-3.238488	-1.438496	-5.132277
24	6	C	-6.07325	-0.785749	-4.984738
25	6	C	-6.721624	-0.136571	-2.229609
26	8	O	-0.608218	2.932455	-0.865146
27	6	C	-7.793025	-2.859237	-5.969851
28	6	C	-7.404814	-5.500364	-4.983333
29	6	C	-9.618379	-2.290916	-7.627356
30	6	C	-2.339128	-1.948652	-7.837358
31	6	C	-0.666431	-0.012953	-9.149528
32	8	O	-3.017421	-3.885396	-8.854786
33	6	C	-2.211261	2.419794	-9.654353
34	6	C	0.494915	-1.137238	-11.539201
35	1	H	-2.868842	-3.162032	-4.032528
36	6	C	-5.889824	4.581649	-2.178503
37	6	C	-0.648372	4.590293	-10.743656
38	1	H	-1.70003	3.783702	4.218637
39	1	H	0.22302	1.230968	3.308002
40	1	H	-7.21716	-1.454725	5.068738
41	1	H	-5.831758	0.782652	7.14897

42	1	H	-0.263185	-3.583202	11.456452
43	1	H	-2.448053	-1.057507	11.756953
44	1	H	1.101541	1.508917	12.634362
45	1	H	3.256296	-0.699586	11.325315
46	1	H	-3.62544	-6.333462	8.772742
47	1	H	-5.698199	-4.006055	10.056053
48	1	H	-6.558012	-5.665048	7.242951
49	1	H	2.17126	5.545464	7.024437
50	1	H	3.868948	4.883109	9.857802
51	1	H	0.540037	5.458731	9.964536
52	1	H	3.817666	1.568926	5.541259
53	1	H	-6.395139	0.904605	-6.150768
54	1	H	-6.531433	-1.853976	-1.083247
55	1	H	-8.703564	0.474919	-2.104652
56	1	H	-5.676158	-6.30716	-5.814098
57	1	H	-7.181721	-5.5442	-2.912902
58	1	H	-9.000659	-6.729285	-5.490684
59	1	H	-10.936729	-3.727243	-8.311198
60	1	H	-9.848051	-0.375448	-8.37015
61	1	H	0.829259	0.492495	-7.788476
62	1	H	-3.786746	1.955787	-10.942446
63	1	H	-3.066716	3.058376	-7.870146
64	1	H	1.506228	-2.902044	-11.113927
65	1	H	-0.979209	-1.593506	-12.933568
66	1	H	1.842369	0.18495	-12.405564
67	1	H	-5.805809	4.629513	-4.253228
68	1	H	-7.836492	4.944727	-1.564529
69	1	H	-4.67197	6.090508	-1.438511
70	1	H	-1.806648	6.308543	-10.938113
71	1	H	0.957231	5.046658	-9.497508
72	1	H	0.114701	4.138618	-12.6246

2*R*,4*S*,5*S*,17*S*,19*S*,20*R*,23*R*,24*S*,*P*-**4** (7)		Standard Orientation (A.U.)			
Center number	Atomic number	Atomic Type	X	Y	Z
1	6	C	-1.607357	0.731834	2.37286
2	6	C	-4.224124	-0.595146	2.610014
3	6	C	-3.886538	-3.418665	1.8852
4	8	O	-3.494937	-4.787929	3.979929
5	6	C	-3.172657	-3.100331	6.161583
6	6	C	-0.37689	-2.238186	6.04387
7	6	C	-0.090933	0.357041	4.797224
8	6	C	-4.948912	-0.920204	5.427042
9	6	C	1.27109	-1.908704	8.402989
10	6	C	3.568069	-0.372436	7.394282
11	6	C	2.794707	0.741577	4.759449
12	6	C	-6.480859	0.396414	1.053192
13	6	C	-6.481298	2.85838	-0.551849
14	6	C	-3.950687	3.713329	-1.636592
15	8	O	-8.451865	-0.745777	1.264765
16	6	C	-3.903824	-4.562316	8.505479
17	8	O	-3.891653	-4.310405	-0.202928
18	1	H	-0.805181	1.732795	6.193154
19	6	C	3.584169	3.47566	4.363462
20	8	O	3.933844	-0.632819	2.739329
21	1	H	0.549121	-3.673095	4.844336
22	8	O	-2.996842	2.207446	-3.440215
23	6	C	-4.436118	0.079867	-4.327589
24	6	C	-7.075603	1.038933	-5.112815
25	6	C	-8.270967	2.509893	-2.850582
26	8	O	-2.871465	5.638311	-1.074574
27	6	C	-8.842537	-0.990512	-6.099665
28	6	C	-9.238818	-3.305148	-4.496114
29	6	C	-10.045464	-0.634374	-8.297317
30	6	C	-2.959898	-1.257108	-6.428006
31	6	C	-0.359154	-0.333962	-7.276705
32	8	O	-3.931615	-3.094146	-7.386212
33	6	C	1.643734	-0.264259	-5.160443
34	6	C	-0.562627	2.208423	-8.669702
35	1	H	-4.580635	-1.341114	-2.811419
36	6	C	-7.49352	4.897743	1.262975
37	6	C	1.844981	-2.703607	-3.626192
38	1	H	-1.856907	2.762034	2.075601
39	1	H	-0.569853	-0.001573	0.730113
40	1	H	-6.924899	-1.533997	5.578888
41	1	H	-4.674053	0.788185	6.568908

42	1	H	1.826619	-3.709914	9.27548
43	1	H	0.241453	-0.814508	9.84499
44	1	H	4.112334	1.134505	8.717473
45	1	H	5.245716	-1.571133	7.129139
46	1	H	-5.850093	-5.271071	8.338896
47	1	H	-2.631688	-6.181495	8.784553
48	1	H	-3.779181	-3.336469	10.178973
49	1	H	5.655505	3.639188	4.438774
50	1	H	2.76162	4.70413	5.825125
51	1	H	2.953466	4.145566	2.498377
52	1	H	3.564784	-2.409516	2.936145
53	1	H	-6.742665	2.397596	-6.652474
54	1	H	-9.973128	1.542346	-2.170946
55	1	H	-8.854114	4.395107	-3.497848
56	1	H	-9.753682	-2.816366	-2.540372
57	1	H	-10.731039	-4.51137	-5.291322
58	1	H	-7.486167	-4.422061	-4.404263
59	1	H	-11.389529	-2.025564	-9.022983
60	1	H	-9.709218	1.04394	-9.456101
61	1	H	0.203988	-1.792564	-8.653577
62	1	H	3.477236	0.137917	-6.070145
63	1	H	1.262617	1.338199	-3.897354
64	1	H	-1.972446	2.122116	-10.1987
65	1	H	-1.080239	3.743261	-7.370279
66	1	H	1.272296	2.684758	-9.529512
67	1	H	-9.418289	4.404071	1.865131
68	1	H	-6.28771	5.075588	2.947108
69	1	H	-7.507251	6.74341	0.314192
70	1	H	2.42315	-4.312377	-4.816125
71	1	H	3.241053	-2.478314	-2.10079
72	1	H	0.034092	-3.226204	-2.742005

2*R*,4*S*,5*S*,17*S*,19*S*,20*R*,23*R*,24*S*,*P*-**4** (8)		Standard Orientation (A.U.)			
Center number	Atomic number	Atomic Type	X	Y	Z
1	6	C	-1.796791	1.063248	3.540716
2	6	C	-4.075069	-0.746039	3.000418
3	6	C	-2.932222	-3.321059	2.181868
4	8	O	-2.644117	-4.786187	4.225609
5	6	C	-3.325482	-3.38428	6.529169
6	6	C	-0.930206	-1.89116	7.281722
7	6	C	-0.99976	0.824682	6.29754
8	6	C	-5.358081	-1.589696	5.487673
9	6	C	-0.169728	-1.46842	10.043739
10	6	C	1.818474	0.688018	9.855823
11	6	C	1.575114	1.87152	7.169691
12	6	C	-6.043423	0.015502	0.991914
13	6	C	-5.815684	2.461257	-0.621476
14	6	C	-3.133539	2.991585	-1.562559
15	8	O	-7.908714	-1.291648	0.78799
16	6	C	-4.224761	-5.295525	8.455026
17	8	O	-2.326815	-3.951263	0.08801
18	1	H	-2.419942	1.845537	7.436099
19	6	C	1.734717	4.748281	7.129959
20	8	O	3.571773	0.814324	5.695549
21	1	H	0.660608	-2.854079	6.348114
22	8	O	-2.248208	1.334966	-3.271613
23	6	C	-3.913209	-0.497953	-4.406279
24	6	C	-6.328756	0.885191	-5.271013
25	6	C	-7.540795	2.264582	-2.979129
26	8	O	-1.857408	4.767476	-0.931596
27	6	C	-8.234364	-0.786772	-6.608826
28	6	C	-8.989187	-3.209202	-5.32245
29	6	C	-9.206785	-0.052963	-8.827802
30	6	C	-2.535577	-1.73132	-6.627448
31	6	C	-0.257537	-0.396976	-7.767708
32	8	O	-3.303694	-3.738265	-7.413284
33	6	C	-0.118863	-0.847262	-10.625053
34	6	C	2.106592	-1.371103	-6.374427
35	1	H	-4.302095	-2.006667	-3.038851
36	6	C	-6.751087	4.615895	1.100395
37	6	C	-2.295765	0.372418	-12.079409
38	1	H	-2.288808	3.027754	3.132588
39	1	H	-0.229895	0.576765	2.262059
40	1	H	-7.092768	-2.638984	5.048515
41	1	H	-5.820624	-0.033136	6.776609

42	1	H	0.58705	-3.173556	10.955882
43	1	H	-1.816001	-0.844993	11.159108
44	1	H	1.530909	2.123085	11.330415
45	1	H	3.755335	-0.032617	10.050992
46	1	H	-4.856031	-4.326699	10.181416
47	1	H	-5.807996	-6.404594	7.692366
48	1	H	-2.686934	-6.597962	8.962249
49	1	H	0.231533	5.60608	8.284566
50	1	H	1.53254	5.472262	5.185124
51	1	H	3.575693	5.377207	7.865342
52	1	H	3.56835	1.623406	4.057349
53	1	H	-5.684929	2.330283	-6.624557
54	1	H	-9.277478	1.297273	-2.39316
55	1	H	-8.071488	4.185731	-3.561348
56	1	H	-7.424824	-4.575721	-5.455364
57	1	H	-9.386721	-2.920239	-3.300835
58	1	H	-10.663173	-4.047194	-6.223108
59	1	H	-10.639656	-1.18194	-9.798138
60	1	H	-8.595583	1.690883	-9.7532
61	1	H	-0.433407	1.640962	-7.382081
62	1	H	1.703558	-0.106033	-11.31482
63	1	H	-0.104802	-2.901075	-10.96753
64	1	H	3.802838	-0.384155	-7.0652
65	1	H	1.958134	-1.061094	-4.325658
66	1	H	2.349558	-3.410263	-6.709953
67	1	H	-6.432023	6.443657	0.169758
68	1	H	-8.781756	4.374375	1.467847
69	1	H	-5.759103	4.662144	2.922577
70	1	H	-2.120236	0.039046	-14.126072
71	1	H	-4.131692	-0.399458	-11.475299
72	1	H	-2.340466	2.435489	-11.780656

2*R*,4*S*,5*S*,17*S*,19*S*,20*R*,23*R*,24*S*,*P*-**4** (9)		Standard Orientation (A.U.)			
Center number	Atomic number	Atomic Type	X	Y	Z
1	6	C	-0.807908	1.298598	4.16784
2	6	C	-3.468949	0.142391	3.416726
3	6	C	-2.981105	-2.425013	2.178074
4	8	O	-3.006856	-4.224878	3.967785
5	6	C	-3.358727	-3.082191	6.486932
6	6	C	-0.685695	-2.307789	7.383073
7	6	C	-0.125771	0.480327	6.837054
8	6	C	-4.904406	-0.729314	5.803578
9	6	C	0.141627	-2.522295	10.149955
10	6	C	2.578617	-0.883851	10.240267
11	6	C	2.61118	0.790963	7.793304
12	6	C	-5.006074	2.089297	1.91623
13	6	C	-5.016899	2.217735	-1.000206
14	6	C	-2.39043	1.752736	-2.108558
15	8	O	-6.329999	3.521094	3.111512
16	6	C	-4.673784	-5.020054	8.125187
17	8	O	-2.596589	-2.895566	-0.015906
18	1	H	-1.295242	1.578787	8.168136
19	6	C	3.358333	3.523988	8.276564
20	8	O	4.388134	-0.128846	5.994655
21	1	H	0.607487	-3.491598	6.248848
22	8	O	-2.217793	0.551787	-4.338319
23	6	C	-4.075205	-1.194148	-5.250539
24	6	C	-6.781197	-0.225733	-4.779068
25	6	C	-7.055012	0.344788	-1.94365
26	8	O	-0.534394	2.722685	-1.21376
27	6	C	-8.822713	-2.031745	-5.671716
28	6	C	-8.684962	-4.738949	-4.804011
29	6	C	-10.682799	-1.181886	-7.162286
30	6	C	-3.547606	-1.728555	-8.046123
31	6	C	-2.119926	0.157664	-9.695246
32	8	O	-4.336264	-3.694682	-8.919611
33	6	C	0.736911	0.259465	-9.092642
34	6	C	-3.325248	2.795236	-9.678507
35	1	H	-3.790483	-2.978486	-4.22917
36	6	C	-5.714338	4.940835	-1.791261
37	6	C	1.964863	-2.338216	-8.812897
38	1	H	-0.903188	3.366428	4.031161
39	1	H	0.628625	0.696231	2.800655
40	1	H	-6.855186	-1.26104	5.319746
41	1	H	-4.985695	0.696936	7.29838

42	1	H	0.46272	-4.480561	10.764417
43	1	H	-1.323532	-1.716216	11.391567
44	1	H	2.643436	0.28685	11.956
45	1	H	4.297911	-2.052601	10.245841
46	1	H	-6.468442	-5.605701	7.255967
47	1	H	-3.48088	-6.703343	8.376278
48	1	H	-5.073792	-4.214724	9.998585
49	1	H	5.292476	3.609115	9.035257
50	1	H	2.064562	4.419287	9.63573
51	1	H	3.319281	4.603506	6.500304
52	1	H	4.0101	-1.874323	5.619387
53	1	H	-7.007834	1.552795	-5.83037
54	1	H	-6.939781	-1.441162	-0.894976
55	1	H	-8.930913	1.157738	-1.570687
56	1	H	-8.328423	-4.890828	-2.757632
57	1	H	-10.4471	-5.751691	-5.232684
58	1	H	-7.123949	-5.70695	-5.781322
59	1	H	-12.214209	-2.420738	-7.78614
60	1	H	-10.73199	0.777276	-7.820548
61	1	H	-2.330116	-0.657173	-11.601011
62	1	H	1.644304	1.306955	-10.651967
63	1	H	1.043205	1.367502	-7.362895
64	1	H	-5.340762	2.721979	-10.193647
65	1	H	-3.145598	3.704833	-7.819422
66	1	H	-2.366157	4.012692	-11.067539
67	1	H	-7.527677	5.46694	-0.935033
68	1	H	-4.263265	6.272575	-1.137523
69	1	H	-5.861025	5.092615	-3.857221
70	1	H	1.574159	-3.553037	-10.458988
71	1	H	4.028548	-2.168379	-8.611246
72	1	H	1.258221	-3.330572	-7.124169

TABLE S6 Gibbs free energies[a] and equilibrium populations[b] of low-energy conformers of 2*R*,4*S*,5*S*,17*S*,19*S*,20*R*,23*R*,24*S*,*M*-**4**.

Conformers	ΔG (a.u.)	*P*(%)/100	G (a.u.)
2*R*,4*S*,5*S*,17*S*,19*S*,20*R*,23*R*,24*S*,*M*-**4** (1)_tddft_	0.0004	26.69	-1577.837278
2*R*,4*S*,5*S*,17*S*,19*S*,20*R*,23*R*,24*S*,*M*-**4** (2)_tddft_	0.0013	10.27	-1577.836376
2*R*,4*S*,5*S*,17*S*,19*S*,20*R*,23*R*,24*S*,*M*-**4** (3)_tddft_	0.0	40.82	-1577.837679
2*R*,4*S*,5*S*,17*S*,19*S*,20*R*,23*R*,24*S*,*M*-**4** (4)_tddft_	0.00058	22.04	-1577.837097
2*R*,4*S*,5*S*,17*S*,19*S*,20*R*,23*R*,24*S*,*M*-**4** (5)_tddft_	0.00569	0.1	-1577.831986
2*R*,4*S*,5*S*,17*S*,19*S*,20*R*,23*R*,24*S*,*M*-**4** (6)_tddft_	0.0058	0.09	-1577.831875

[a]wB97M-V/def2-TZVP, in a.u. [b]From ΔG values at 298.15 K.

TABLE S7 Cartesian coordinates for the low-energy reoptimized random research conformers of 2*R*,4*S*,5*S*,17*S*,19*S*,20*R*,23*R*,24*S*,*M*-**4** at B3LYP-D3(BJ)/6-31G* level of theory in methanol.

2*R*,4*S*,5*S*,17*S*,19*S*,20*R*,23*R*,24*S*,*M*-**4** (1)		Standard Orientation (A.U.)			
Center number	Atomic number	Atomic Type	X	Y	Z
1	6	C	-0.468681	0.161306	4.537235
2	6	C	-1.431951	-0.354827	1.773379
3	6	C	0.869036	-1.439341	0.322549
4	8	O	0.817705	-3.971758	0.501213
5	6	C	-1.234238	-4.792883	2.164755
6	6	C	-0.1541	-4.653216	4.876873
7	6	C	-0.872521	-2.168835	6.178703
8	6	C	-3.197574	-2.684629	1.761608
9	6	C	-0.834527	-6.589751	6.918929
10	6	C	-0.033836	-5.245968	9.409823
11	6	C	0.45319	-2.402325	8.744102
12	6	C	-2.477142	2.097928	0.606888
13	6	C	-3.259126	2.352154	-2.243854
14	6	C	-3.854723	-0.200575	-3.447622
15	8	O	-2.58764	4.000295	1.869092
16	6	C	-2.055789	-7.389142	1.291597
17	8	O	2.556615	-0.285717	-0.65335
18	1	H	-2.906958	-2.288895	6.623118
19	6	C	-0.518516	-0.56664	10.742533
20	8	O	3.071166	-1.961648	8.281215
21	1	H	1.913762	-4.673549	4.661979
22	8	O	-6.310587	-0.903856	-3.716986
23	6	C	-8.395312	0.82769	-3.701731
24	6	C	-8.055821	3.036436	-1.894072
25	6	C	-5.474265	4.225724	-2.477841
26	8	O	-2.25027	-1.68445	-4.072904
27	6	C	-8.483481	2.430913	0.87729
28	6	C	-8.565028	4.690689	2.604625
29	6	C	-8.817056	0.078428	1.755775
30	6	C	-9.031697	1.692941	-6.411798
31	6	C	-8.1292	0.076963	-8.617186
32	8	O	-10.272275	3.592144	-6.716264
33	6	C	-10.171601	-0.081741	-10.669814
34	6	C	-5.665036	1.236949	-9.647294
35	1	H	-10.030568	-0.331735	-3.131027
36	6	C	-0.927681	3.50106	-3.598153
37	6	C	-12.56471	-1.449181	-9.811115
38	1	H	-1.499492	1.793854	5.290611
39	1	H	1.540869	0.683396	4.481365

40	1	H	-4.161367	-2.968357	-0.041321
41	1	H	-4.636475	-2.621651	3.245425
42	1	H	0.109865	-8.42101	6.657401
43	1	H	-2.887127	-6.948171	6.922847
44	1	H	-1.495966	-5.432803	10.876448
45	1	H	1.71671	-6.058789	10.183814
46	1	H	-2.580569	-7.322654	-0.718135
47	1	H	-0.516512	-8.765382	1.530167
48	1	H	-3.69268	-8.036036	2.397692
49	1	H	0.431545	-0.89364	12.569935
50	1	H	-2.564371	-0.791376	11.047623
51	1	H	-0.14443	1.39558	10.164834
52	1	H	3.971596	-2.142317	9.858477
53	1	H	-9.506014	4.423542	-2.451671
54	1	H	-5.08583	5.848894	-1.25075
55	1	H	-5.518983	4.948149	-4.428054
56	1	H	-9.88394	6.132274	1.878039
57	1	H	-9.162128	4.157054	4.521191
58	1	H	-6.687669	5.578011	2.742727
59	1	H	-9.185845	-0.257734	3.76059
60	1	H	-8.728481	-1.586784	0.544202
61	1	H	-7.696034	-1.8279	-7.901993
62	1	H	-10.641425	1.851846	-11.284018
63	1	H	-9.35196	-1.057583	-12.319032
64	1	H	-4.991939	0.134184	-11.276828
65	1	H	-4.138634	1.237971	-8.237977
66	1	H	-5.989575	3.195073	-10.272346
67	1	H	0.739697	2.293652	-3.405627
68	1	H	-1.360544	3.762504	-5.614604
69	1	H	-0.520084	5.357089	-2.760267
70	1	H	-13.953611	-1.579433	-11.354718
71	1	H	-12.142962	-3.389036	-9.17657
72	1	H	-13.486812	-0.447403	-8.2355

2*R*,4*S*,5*S*,17*S*,19*S*,20*R*,23*R*,24*S*,*M*-**4** (2)		Standard Orientation (A.U.)			
Center number	Atomic number	Atomic Type	X	Y	Z
1	6	C	-0.333788	-0.055636	4.20965
2	6	C	-1.485451	-0.542205	1.512452
3	6	C	0.60881	-1.925649	0.005205
4	8	O	0.296464	-4.427635	0.302046
5	6	C	-1.769548	-4.958434	2.067805
6	6	C	-0.577211	-4.856079	4.731608
7	6	C	-0.941672	-2.259053	5.958399
8	6	C	-3.4982	-2.655589	1.6581
9	6	C	-1.405094	-6.619825	6.871994
10	6	C	-0.329677	-5.302271	9.271793
11	6	C	0.45802	-2.559993	8.477091
12	6	C	-2.288749	1.975994	0.300524
13	6	C	-3.202171	2.225281	-2.510733
14	6	C	-4.152179	-0.275488	-3.587878
15	8	O	-2.08841	3.922197	1.482426
16	6	C	-2.907258	-7.471843	1.314733
17	8	O	2.355118	-0.999811	-1.101349
18	1	H	-2.9555	-2.117402	6.484005
19	6	C	-0.202221	-0.55455	10.439573
20	8	O	3.08692	-2.448475	7.895069
21	1	H	1.463759	-5.134975	4.448056
22	8	O	-6.681167	-0.691107	-3.709943
23	6	C	-8.546414	1.265252	-3.675789
24	6	C	-7.864574	3.459065	-1.931874
25	6	C	-5.20258	4.335589	-2.683994
26	8	O	-2.765227	-1.949875	-4.254797
27	6	C	-8.196994	2.945895	0.871958
28	6	C	-7.921715	5.226714	2.552304
29	6	C	-8.759286	0.663116	1.813896
30	6	C	-9.140382	2.24224	-6.360392
31	6	C	-7.757079	1.175399	-8.650489
32	8	O	-10.764594	3.842256	-6.563878
33	6	C	-9.335823	-0.987944	-9.829396
34	6	C	-7.281417	3.283667	-10.574347
35	1	H	-10.286701	0.338218	-3.020476
36	6	C	-0.844519	3.046999	-4.044235
37	6	C	-9.756975	-3.290204	-8.138291
38	1	H	-1.111664	1.720018	4.943242
39	1	H	1.720365	0.205135	4.047252
40	1	H	-4.558818	-2.892152	-0.096401
41	1	H	-4.863569	-2.378862	3.186609

42	1	H	-0.711171	-8.564688	6.648148
43	1	H	-3.484432	-6.711923	6.971985
44	1	H	-1.728183	-5.265877	10.810069
45	1	H	1.348892	-6.295787	9.992688
46	1	H	-3.495069	-7.409859	-0.67771
47	1	H	-1.522632	-9.004138	1.54982
48	1	H	-4.565265	-7.897924	2.494504
49	1	H	0.777585	-0.93451	12.240806
50	1	H	-2.245642	-0.517688	10.829823
51	1	H	0.381816	1.326903	9.773635
52	1	H	4.027448	-2.68637	9.440996
53	1	H	-9.19511	4.985515	-2.422413
54	1	H	-4.561441	5.932328	-1.529888
55	1	H	-5.266614	5.011135	-4.652578
56	1	H	-9.103731	6.799029	1.861967
57	1	H	-8.476019	4.795703	4.507129
58	1	H	-5.950914	5.895595	2.577195
59	1	H	-9.054966	0.401681	3.841871
60	1	H	-8.942133	-1.016106	0.632894
61	1	H	-5.943854	0.36047	-8.047165
62	1	H	-11.166401	-0.191248	-10.433824
63	1	H	-8.349101	-1.579505	-11.567254
64	1	H	-6.323657	2.52358	-12.256186
65	1	H	-6.083342	4.786014	-9.774359
66	1	H	-9.075792	4.152823	-11.158538
67	1	H	0.684549	1.663775	-3.884179
68	1	H	-1.365196	3.264904	-6.045826
69	1	H	-0.179759	4.877395	-3.321889
70	1	H	-10.741867	-4.792248	-9.188713
71	1	H	-7.956649	-4.054164	-7.433248
72	1	H	-10.928393	-2.836331	-6.477316

2*R*,4*S*,5*S*,17*S*,19*S*,20*R*,23*R*,24*S*,*M*-**4** (3)		Standard Orientation (A.U.)			
Center number	Atomic number	Atomic Type	X	Y	Z
1	6	C	-1.017255	-0.59433	4.53546
2	6	C	-1.97209	-1.044433	1.757258
3	6	C	0.236497	-2.387212	0.378657
4	8	O	-0.079235	-4.89314	0.593735
5	6	C	-2.252625	-5.472851	2.212554
6	6	C	-1.236466	-5.408925	4.949398
7	6	C	-1.695215	-2.8421	6.202427
8	6	C	-3.970687	-3.175746	1.731624
9	6	C	-2.180998	-7.219328	7.000153
10	6	C	-1.245256	-5.951386	9.481762
11	6	C	-0.444954	-3.198342	8.80892
12	6	C	-2.703072	1.492254	0.533056
13	6	C	-3.404831	1.785136	-2.332679
14	6	C	-4.249899	-0.708011	-3.511575
15	8	O	-2.59821	3.41663	1.762854
16	6	C	-3.315686	-7.980755	1.342987
17	8	O	2.056685	-1.421142	-0.564254
18	1	H	-3.739431	-2.718349	6.600655
19	6	C	-1.242377	-1.239713	10.767499
20	8	O	2.24337	-3.236209	8.582725
21	1	H	0.821902	-5.668782	4.797971
22	8	O	-6.760318	-1.146378	-3.824114
23	6	C	-8.650662	0.797668	-3.859319
24	6	C	-8.1031	2.980516	-2.073136
25	6	C	-5.402193	3.881194	-2.628469
26	8	O	-2.800349	-2.362692	-4.084791
27	6	C	-8.633467	2.459543	0.698203
28	6	C	-8.504222	4.73732	2.399447
29	6	C	-9.229353	0.166937	1.596081
30	6	C	-9.150844	1.680694	-6.592461
31	6	C	-8.515169	-0.11975	-8.747463
32	8	O	-10.087341	3.737371	-6.955563
33	6	C	-10.586702	-0.045527	-10.776627
34	6	C	-5.922202	0.635477	-9.834985
35	1	H	-10.408095	-0.174003	-3.300799
36	6	C	-0.943727	2.649957	-3.66818
37	6	C	-13.145961	-1.004013	-9.845744
38	1	H	-1.870349	1.156471	5.245536
39	1	H	1.040123	-0.282463	4.475474
40	1	H	-4.912965	-3.382536	-0.092946
41	1	H	-5.435052	-2.939569	3.173025

42	1	H	-1.465353	-9.156136	6.775892
43	1	H	-4.262566	-7.319816	6.973568
44	1	H	-2.718951	-5.95863	10.946651
45	1	H	0.418135	-6.917938	10.261825
46	1	H	-3.776354	-7.887262	-0.681401
47	1	H	-1.934324	-9.505131	1.639388
48	1	H	-5.040097	-8.440762	2.409115
49	1	H	-0.36992	-1.666398	12.606888
50	1	H	-3.309129	-1.201698	11.004987
51	1	H	-0.635589	0.670989	10.194488
52	1	H	2.809592	-1.548574	8.172818
53	1	H	-9.38664	4.508834	-2.669017
54	1	H	-4.860251	5.472244	-1.417047
55	1	H	-5.341463	4.574869	-4.588229
56	1	H	-9.1744	4.290965	4.314149
57	1	H	-6.549707	5.436891	2.550314
58	1	H	-9.663902	6.293965	1.639182
59	1	H	-9.663093	-0.104791	3.597736
60	1	H	-9.303195	-1.512548	0.403275
61	1	H	-8.353945	-2.042756	-7.970028
62	1	H	-10.770218	1.913748	-11.457835
63	1	H	-9.945455	-1.191428	-12.395039
64	1	H	-5.969131	2.59734	-10.527329
65	1	H	-5.434833	-0.607994	-11.429143
66	1	H	-4.396088	0.463223	-8.435297
67	1	H	-0.343473	4.463669	-2.853616
68	1	H	0.577372	1.268499	-3.435423
69	1	H	-1.320515	2.921647	-5.694809
70	1	H	-13.880668	0.179855	-8.298082
71	1	H	-14.562259	-0.973382	-11.369568
72	1	H	-13.013953	-2.963101	-9.146953

2*R*,4*S*,5*S*,17*S*,19*S*,20*R*,23*R*,24*S*,*M*-**4** (4)		Standard Orientation (A.U.)			
Center number	Atomic number	Atomic Type	X	Y	Z
1	6	C	-0.775163	-0.751319	4.167102
2	6	C	-1.889534	-1.199721	1.447978
3	6	C	0.220333	-2.572798	-0.047683
4	8	O	-0.106852	-5.077299	0.209426
5	6	C	-2.197427	-5.621987	1.940769
6	6	C	-1.039103	-5.558848	4.620832
7	6	C	-1.410511	-2.977052	5.877769
8	6	C	-3.911897	-3.30695	1.539468
9	6	C	-1.900326	-7.348084	6.726457
10	6	C	-0.849626	-6.067609	9.156831
11	6	C	-0.043516	-3.317226	8.409446
12	6	C	-2.65624	1.338873	0.2527
13	6	C	-3.532855	1.626943	-2.566631
14	6	C	-4.49378	-0.853102	-3.679482
15	8	O	-2.443828	3.270804	1.455436
16	6	C	-3.33489	-8.120778	1.1401
17	8	O	1.985803	-1.639924	-1.11712
18	1	H	-3.430308	-2.836063	6.380282
19	6	C	-0.722112	-1.336872	10.391052
20	8	O	2.59291	-3.206815	7.862139
21	1	H	1.004351	-5.841222	4.359553
22	8	O	-7.028077	-1.252535	-3.817275
23	6	C	-8.88916	0.716492	-3.723501
24	6	C	-8.182327	2.904105	-1.999021
25	6	C	-5.511329	3.753673	-2.747492
26	8	O	-3.115042	-2.531789	-4.34936
27	6	C	-8.53062	2.414062	0.806473
28	6	C	-8.248052	4.702604	2.474091
29	6	C	-9.102841	0.139273	1.763008
30	6	C	-9.586532	1.590942	-6.415364
31	6	C	-9.116086	-0.217667	-8.605643
32	8	O	-10.540617	3.649858	-6.715098
33	6	C	-11.314177	-0.119875	-10.49549
34	6	C	-6.58988	0.503371	-9.861286
35	1	H	-10.612403	-0.225784	-3.025847
36	6	C	-1.14699	2.444494	-4.058689
37	6	C	-13.817402	-1.054428	-9.402035
38	1	H	-1.561468	1.015423	4.913366
39	1	H	1.281191	-0.490339	4.036573
40	1	H	-4.951398	-3.517347	-0.230971
41	1	H	-5.294772	-3.047188	3.055209

42	1	H	-1.211537	-9.292613	6.484583
43	1	H	-3.981111	-7.432779	6.799214
44	1	H	-2.2661	-6.047849	10.678888
45	1	H	0.817098	-7.07677	9.883529
46	1	H	-3.894903	-8.030341	-0.859247
47	1	H	-1.959851	-9.661827	1.37407
48	1	H	-5.010534	-8.555026	2.29174
49	1	H	0.233954	-1.744609	12.198992
50	1	H	-2.770148	-1.299194	10.756254
51	1	H	-0.124488	0.551824	9.758569
52	1	H	3.512737	-3.469677	9.416435
53	1	H	-9.478935	4.449418	-2.517852
54	1	H	-4.860989	5.34533	-1.591462
55	1	H	-5.572861	4.427067	-4.714629
56	1	H	-8.815035	4.289145	4.429095
57	1	H	-6.271625	5.354594	2.50405
58	1	H	-9.412726	6.280072	1.766469
59	1	H	-9.404997	-0.109158	3.791682
60	1	H	-9.285917	-1.548001	0.593165
61	1	H	-8.927638	-2.141892	-7.837182
62	1	H	-11.521996	1.841767	-11.162731
63	1	H	-10.792418	-1.270402	-12.153254
64	1	H	-6.656716	2.464454	-10.554264
65	1	H	-6.223595	-0.749269	-11.480374
66	1	H	-4.9785	0.314204	-8.563399
67	1	H	-0.467965	4.25287	-3.29556
68	1	H	0.363056	1.039989	-3.907554
69	1	H	-1.642829	2.708836	-6.060517
70	1	H	-14.443271	0.137771	-7.813599
71	1	H	-15.327012	-1.01428	-10.833341
72	1	H	-13.658404	-3.013484	-8.708588

2*R*,4*S*,5*S*,17*S*,19*S*,20*R*,23*R*,24*S*,*M*-**4** (5)		Standard Orientation (A.U.)			
Center number	Atomic number	Atomic Type	X	Y	Z
1	6	C	-2.550867	-4.721957	0.30569
2	6	C	-1.789359	-1.858821	0.624286
3	6	C	-3.280862	-0.78884	2.89591
4	8	O	-1.858001	-1.014601	4.990326
5	6	C	0.456352	-2.444864	4.428957
6	6	C	-0.304071	-5.264273	4.568026
7	6	C	-0.856477	-6.367247	1.953684
8	6	C	0.916532	-1.643967	1.681138
9	6	C	1.446539	-7.289776	5.673655
10	6	C	0.258046	-9.798933	4.707754
11	6	C	-1.659703	-9.094723	2.589453
12	6	C	-2.06213	-0.388755	-1.867153
13	6	C	-4.553246	-0.201823	-3.452601
14	6	C	-6.997024	-0.541143	-1.929025
15	8	O	-0.159255	0.459505	-2.814637
16	6	C	2.446422	-1.628334	6.311734
17	8	O	-5.369937	0.085839	2.923146
18	1	H	0.977583	-6.545116	0.976069
19	6	C	-1.61793	-10.880598	0.327461
20	8	O	-4.095546	-9.120922	3.744825
21	1	H	-2.075273	-5.325602	5.657047
22	8	O	-8.356977	1.51408	-1.313454
23	6	C	-8.024623	4.010673	-2.315136
24	6	C	-5.393005	4.574362	-3.329661
25	6	C	-4.561936	2.307185	-4.931714
26	8	O	-7.798353	-2.593852	-1.347896
27	6	C	-3.455261	5.413262	-1.38437
28	6	C	-1.044276	6.466917	-2.473867
29	6	C	-3.834501	5.279906	1.115258
30	6	C	-10.129777	4.472133	-4.29067
31	6	C	-12.766256	3.417344	-3.778717
32	8	O	-9.694924	5.729964	-6.153829
33	6	C	-13.008297	0.57847	-4.431877
34	6	C	-13.673215	3.954931	-1.077221
35	1	H	-8.461709	5.281817	-0.725609
36	6	C	-4.423526	-2.388151	-5.400956
37	6	C	-11.91767	-0.152782	-7.001005
38	1	H	-2.321058	-5.278806	-1.678398
39	1	H	-4.56545	-4.928562	0.759196
40	1	H	1.556781	0.328276	1.598903
41	1	H	2.307193	-2.807317	0.68028

42	1	H	1.576444	-7.21536	7.744923
43	1	H	3.376578	-7.063723	4.92165
44	1	H	1.707258	-11.104126	3.991585
45	1	H	-0.789718	-10.784544	6.204407
46	1	H	2.723888	0.429546	6.224411
47	1	H	1.881524	-2.138036	8.245713
48	1	H	4.254534	-2.56224	5.890128
49	1	H	-3.008987	-10.287081	-1.107624
50	1	H	-2.103878	-12.811911	0.927032
51	1	H	0.254837	-10.914906	-0.578597
52	1	H	-5.338285	-8.645163	2.492201
53	1	H	-5.657533	6.159662	-4.654286
54	1	H	-2.680254	2.600242	-5.747857
55	1	H	-5.887015	2.122751	-6.522318
56	1	H	0.147019	7.306063	-0.993201
57	1	H	0.062753	4.972777	-3.410041
58	1	H	-1.447802	7.930388	-3.903269
59	1	H	-2.396437	5.956643	2.43655
60	1	H	-5.524651	4.45364	1.951081
61	1	H	-13.957056	4.470072	-5.125973
62	1	H	-12.127827	-0.563584	-2.935582
63	1	H	-15.044299	0.128863	-4.390644
64	1	H	-13.567679	5.982967	-0.617974
65	1	H	-12.554261	2.89616	0.316761
66	1	H	-15.659566	3.368995	-0.875177
67	1	H	-4.617233	-4.237414	-4.495608
68	1	H	-5.982693	-2.194727	-6.760848
69	1	H	-2.626072	-2.289673	-6.436499
70	1	H	-12.307946	-2.1503	-7.430744
71	1	H	-12.710567	1.009194	-8.536145
72	1	H	-9.851195	0.09639	-7.060889

2*R*,4*S*,5*S*,17*S*,19*S*,20*R*,23*R*,24*S*,*M*-**4** (6)		Standard Orientation (A.U.)			
Center number	Atomic number	Atomic Type	X	Y	Z
1	6	C	-0.995067	-1.478319	2.563695
2	6	C	-2.896376	-3.464415	1.472858
3	6	C	-1.366736	-5.940025	1.084133
4	8	O	-1.527755	-7.345762	3.20408
5	6	C	-2.947702	-5.992543	5.162235
6	6	C	-0.996642	-4.277196	6.511268
7	6	C	-1.016758	-1.589796	5.438876
8	6	C	-4.739608	-4.389362	3.54204
9	6	C	-1.085585	-3.745522	9.355627
10	6	C	0.699946	-1.431371	9.653248
11	6	C	1.073995	-0.250668	6.96008
12	6	C	-4.321974	-2.924444	-1.018306
13	6	C	-3.542314	-0.860934	-2.954855
14	6	C	-5.351049	-1.299132	-5.199569
15	8	O	-6.004845	-4.369315	-1.579927
16	6	C	-4.19742	-7.948167	6.832172
17	8	O	-0.15726	-6.553027	-0.726966
18	1	H	-2.799958	-0.727332	6.08646
19	6	C	0.866061	2.616294	6.921265
20	8	O	3.512683	-0.803334	5.960919
21	1	H	0.848546	-5.162748	6.097124
22	8	O	-7.672352	-0.20935	-5.041482
23	6	C	-8.09271	2.221962	-3.940884
24	6	C	-6.532981	2.744903	-1.566314
25	6	C	-3.799954	1.926803	-2.108592
26	8	O	-4.839716	-2.568784	-7.004585
27	6	C	-7.594325	1.693218	0.883364
28	6	C	-6.636194	2.979119	3.238432
29	6	C	-9.293975	-0.178151	0.978814
30	6	C	-7.692004	4.340356	-5.927116
31	6	C	-7.379018	3.585543	-8.676269
32	8	O	-7.67749	6.514105	-5.209191
33	6	C	-9.81782	2.308932	-9.691246
34	6	C	-6.557736	5.838547	-10.280582
35	1	H	-10.117978	2.26224	-3.468518
36	6	C	-0.816488	-1.395802	-3.820726
37	6	C	-9.275094	0.446219	-11.828514
38	1	H	-1.497315	0.42931	1.95802
39	1	H	0.922558	-1.842087	1.851288
40	1	H	-6.19589	-5.58204	2.673649
41	1	H	-5.669716	-2.863987	4.588022

42	1	H	-0.501788	-5.366203	10.515979
43	1	H	-3.026331	-3.245483	9.923632
44	1	H	-0.073484	-0.037573	10.986555
45	1	H	2.566787	-1.983382	10.383561
46	1	H	-5.393568	-9.208185	5.692446
47	1	H	-2.772128	-9.098843	7.814426
48	1	H	-5.383998	-7.015419	8.261137
49	1	H	2.351727	3.458353	8.107275
50	1	H	-0.987704	3.237523	7.628564
51	1	H	1.120622	3.331784	4.984728
52	1	H	3.77286	-2.6099	5.996147
53	1	H	-6.533674	4.818578	-1.383322
54	1	H	-2.566018	2.343136	-0.501722
55	1	H	-3.065545	3.09446	-3.672752
56	1	H	-4.557693	2.914974	3.366596
57	1	H	-7.156601	4.998507	3.235039
58	1	H	-7.409946	2.111445	4.960422
59	1	H	-10.035227	-0.848395	2.787018
60	1	H	-9.958276	-1.149834	-0.714229
61	1	H	-5.891786	2.124914	-8.719345
62	1	H	-10.780183	1.280154	-8.163783
63	1	H	-11.124115	3.810088	-10.316342
64	1	H	-6.354521	5.282435	-12.272943
65	1	H	-4.738021	6.60943	-9.634196
66	1	H	-7.957507	7.370724	-10.159618
67	1	H	-0.572717	-3.383464	-4.34729
68	1	H	-0.371289	-0.212241	-5.46982
69	1	H	0.53564	-0.949401	-2.312646
70	1	H	-11.036255	-0.367081	-12.581924
71	1	H	-8.275599	1.35617	-13.412569
72	1	H	-8.088448	-1.111493	-11.123204

TABLE S8 Gibbs free energies[a] and equilibrium populations[b] of low-energy conformers of 2*R*,4*S*,5*S*,17*S*,19*S*,20*R*,23*R*,24*S*,*P*-**4**.

Conformers	ΔG (a.u.)	*P*(%)/100	G (a.u.)
2*R*,4*S*,5*S*,17*S*,19*S*,20*R*,23*R*,24*S*,*P*-**4** (1)_tddft_	0.0013	13.5	-1577.840975
2*R*,4*S*,5*S*,17*S*,19*S*,20*R*,23*R*,24*S*,*P*-**4** (2)_tddft_	0.0	53.67	-1577.842278
2*R*,4*S*,5*S*,17*S*,19*S*,20*R*,23*R*,24*S*,*P*-**4** (3)_tddft_	0.01001	0.0	-1577.83227
2*R*,4*S*,5*S*,17*S*,19*S*,20*R*,23*R*,24*S*,*P*-**4** (4)_tddft_	0.0068	0.04	-1577.835476
2*R*,4*S*,5*S*,17*S*,19*S*,20*R*,23*R*,24*S*,*P*-**4** (5)_tddft_	0.00103	18.05	-1577.841249
2*R*,4*S*,5*S*,17*S*,19*S*,20*R*,23*R*,24*S*,*P*-**4** (6)_tddft_	0.00126	14.08	-1577.841015
2*R*,4*S*,5*S*,17*S*,19*S*,20*R*,23*R*,24*S*,*P*-**4** (7)_tddft_	0.01024	0.0	-1577.832035
2*R*,4*S*,5*S*,17*S*,19*S*,20*R*,23*R*,24*S*,*P*-**4** (8)_tddft_	0.00584	0.11	-1577.836434
2*R*,4*S*,5*S*,17*S*,19*S*,20*R*,23*R*,24*S*,*P*-**4** (9)_tddft_	0.00433	0.55	-1577.837949

[a]wB97M-V/def2-TZVP, in a.u.

[b]From ΔG values at 298.15 K.

TABLE S9 Cartesian coordinates for the low-energy reoptimized random research conformers of 2*R*,4*S*,5*S*,17*S*,19*S*,20*R*,23*R*,24*S*,*P*-**4** at B3LYP-D3(BJ)/6-31G* level of theory in methanol.

2*R*,4*S*,5*S*,17*S*,19*S*,20*R*,23*R*,24*S*,*P*-**4** (1)		Standard Orientation (A.U.)			
Center number	Atomic number	Atomic Type	X	Y	Z
1	6	C	-0.91435	0.984372	4.589557
2	6	C	-3.606105	0.103936	3.609122
3	6	C	-3.281213	-2.524259	2.444706
4	8	O	-3.635726	-4.273246	4.245714
5	6	C	-4.108324	-3.051886	6.709867
6	6	C	-1.477836	-2.521771	7.8606
7	6	C	-0.597171	0.187559	7.334785
8	6	C	-5.342849	-0.574849	5.855666
9	6	C	-0.977628	-2.74614	10.705579
10	6	C	1.53441	-1.261094	11.069788
11	6	C	2.030053	0.226946	8.561294
12	6	C	-4.777427	2.189076	1.973065
13	6	C	-4.622915	2.209802	-0.940446
14	6	C	-1.985151	1.46464	-1.863073
15	8	O	-5.937905	3.837701	3.054529
16	6	C	-5.764724	-4.82318	8.223304
17	8	O	-2.777157	-3.072594	0.29279
18	1	H	-1.764868	1.450413	8.513906
19	6	C	3.100241	2.869407	8.97532
20	8	O	3.639529	-1.180233	6.921059
21	1	H	-0.16223	-3.80729	6.891359
22	8	O	-1.788306	0.230376	-4.078898
23	6	C	-3.749934	-1.372086	-5.042106
24	6	C	-6.375281	-0.15716	-4.749287
25	6	C	-6.776441	0.508963	-1.95362
26	8	O	-0.109393	2.250591	-0.841534
27	6	C	-8.487015	-1.803801	-5.776927
28	6	C	-8.582501	-4.519631	-4.930129
29	6	C	-10.17724	-0.824283	-7.386661
30	6	C	-3.183589	-1.883973	-7.826057
31	6	C	-2.169935	0.287316	-9.417588
32	8	O	-3.614511	-3.953479	-8.705313
33	6	C	-3.542716	0.351836	-11.978315
34	6	C	0.712897	-0.027688	-9.646001
35	1	H	-3.659421	-3.166249	-4.00858
36	6	C	-5.003431	4.956761	-1.85484
37	6	C	-2.808059	2.584292	-13.652317
38	1	H	-0.762465	3.043949	4.392147
39	1	H	0.564115	0.161597	3.393734

40	1	H	-7.281809	-0.922992	5.188737
41	1	H	-5.42531	0.885157	7.317559
42	1	H	-0.847625	-4.713444	11.358414
43	1	H	-2.51101	-1.836429	11.783044
44	1	H	1.418566	0.030849	12.69464
45	1	H	3.135847	-2.540571	11.418304
46	1	H	-4.768007	-6.604198	8.616303
47	1	H	-6.278527	-3.946421	10.035907
48	1	H	-7.50704	-5.259976	7.177262
49	1	H	4.933109	2.775624	9.965764
50	1	H	1.819494	4.033742	10.129726
51	1	H	3.403483	3.821289	7.151412
52	1	H	5.320173	-1.19046	7.632532
53	1	H	-6.367064	1.613734	-5.842061
54	1	H	-6.899494	-1.246255	-0.855521
55	1	H	-8.583116	1.510765	-1.72209
56	1	H	-10.379829	-5.410426	-5.468782
57	1	H	-7.034094	-5.590574	-5.81966
58	1	H	-8.349273	-4.703587	-2.868789
59	1	H	-11.74424	-1.952496	-8.122043
60	1	H	-10.046651	1.136615	-8.028317
61	1	H	-2.532684	2.067461	-8.395638
62	1	H	-3.186612	-1.450602	-12.95813
63	1	H	-5.596887	0.3994	-11.619777
64	1	H	1.595836	-0.090049	-7.766266
65	1	H	1.165006	-1.794632	-10.648912
66	1	H	1.550766	1.558697	-10.695324
67	1	H	-3.483968	6.171613	-1.133018
68	1	H	-4.985984	5.051703	-3.929341
69	1	H	-6.813935	5.681459	-1.150159
70	1	H	-3.116544	4.40061	-12.676195
71	1	H	-0.807399	2.511833	-14.219041
72	1	H	-3.943475	2.609451	-15.396332

2*R*,4*S*,5*S*,17*S*,19*S*,20*R*,23*R*,24*S*,*P*-**4** (2)		Standard Orientation (A.U.)			
Center number	Atomic number	Atomic Type	X	Y	Z
1	6	C	-1.49577	1.18221	4.75512
2	6	C	-3.984775	0.026861	3.557288
3	6	C	-3.293075	-2.552057	2.434417
4	8	O	-3.637562	-4.338032	4.193967
5	6	C	-4.413479	-3.179071	6.6153
6	6	C	-1.940511	-2.400153	7.957785
7	6	C	-1.291968	0.384694	7.510582
8	6	C	-5.817597	-0.833025	5.658238
9	6	C	-1.617631	-2.603667	10.828142
10	6	C	0.754385	-0.948921	11.344559
11	6	C	1.242762	0.657853	8.926133
12	6	C	-5.21985	1.975398	1.801935
13	6	C	-4.743479	2.052831	-1.075396
14	6	C	-1.965123	1.589237	-1.713882
15	8	O	-6.694056	3.450028	2.742209
16	6	C	-5.997156	-5.111193	8.003377
17	8	O	-2.537227	-3.026597	0.337839
18	1	H	-2.6611	1.518064	8.601993
19	6	C	1.990237	3.38674	9.471206
20	8	O	3.216939	-0.595825	7.582043
21	1	H	-0.430746	-3.537301	7.088044
22	8	O	-1.416937	0.412638	-3.895837
23	6	C	-3.075754	-1.363834	-5.094099
24	6	C	-5.849209	-0.482029	-5.045327
25	6	C	-6.587876	0.149007	-2.309135
26	8	O	-0.287681	2.542577	-0.502147
27	6	C	-7.669159	-2.400468	-6.165075
28	6	C	-7.409639	-5.115073	-5.348353
29	6	C	-9.458436	-1.638861	-7.785205
30	6	C	-2.1746	-1.715164	-7.815778
31	6	C	-0.881173	0.49875	-9.127079
32	8	O	-2.546779	-3.714945	-8.866901
33	6	C	-1.61886	0.611732	-11.926667
34	6	C	1.992089	0.212565	-8.757245
35	1	H	-2.880852	-3.167099	-4.089825
36	6	C	-5.306261	4.753838	-2.030258
37	6	C	-4.410449	1.212037	-12.355408
38	1	H	-1.553491	3.248614	4.5771
39	1	H	0.130329	0.537893	3.640008
40	1	H	-7.65209	-1.367723	4.837829
41	1	H	-6.163684	0.60213	7.106922

42	1	H	-1.385027	-4.558128	11.490731
43	1	H	-3.282037	-1.810563	11.799105
44	1	H	0.48901	0.269048	13.006615
45	1	H	2.444091	-2.107282	11.679704
46	1	H	-7.599178	-5.718291	6.826285
47	1	H	-4.860314	-6.782826	8.48495
48	1	H	-6.738583	-4.294439	9.764706
49	1	H	0.503725	4.381058	10.534231
50	1	H	2.313111	4.436267	7.698852
51	1	H	3.749842	3.441977	10.578864
52	1	H	3.611528	0.382404	6.089933
53	1	H	-5.978758	1.259387	-6.178901
54	1	H	-6.652908	-1.613384	-1.218387
55	1	H	-8.500947	0.960098	-2.279068
56	1	H	-5.723766	-5.955901	-6.233386
57	1	H	-7.17439	-5.293347	-3.286526
58	1	H	-9.068142	-6.228878	-5.916433
59	1	H	-10.843856	-2.96258	-8.558174
60	1	H	-9.593751	0.329237	-8.400583
61	1	H	-1.47768	2.253758	-8.175079
62	1	H	-0.428913	2.048809	-12.856577
63	1	H	-1.148428	-1.219303	-12.799543
64	1	H	2.987621	1.807025	-9.64933
65	1	H	2.490458	0.175565	-6.741687
66	1	H	2.658798	-1.548644	-9.643303
67	1	H	-5.092732	4.862224	-4.093339
68	1	H	-7.242175	5.286103	-1.512006
69	1	H	-3.996697	6.110887	-1.164167
70	1	H	-4.859004	1.319387	-14.384533
71	1	H	-5.63362	-0.248777	-11.519743
72	1	H	-4.927408	3.042694	-11.500853

2*R*,4*S*,5*S*,17*S*,19*S*,20*R*,23*R*,24*S*,*P*-**4** (3)		Standard Orientation (A.U.)			
Center number	Atomic number	Atomic Type	X	Y	Z
1	6	C	-1.562378	1.348008	2.709519
2	6	C	-3.920916	-0.418869	2.709726
3	6	C	-3.075897	-3.08457	1.839991
4	8	O	-2.477676	-4.481802	3.860186
5	6	C	-2.596032	-2.926363	6.16459
6	6	C	-0.023318	-1.545516	6.302292
7	6	C	-0.192677	1.139094	5.237585
8	6	C	-4.717496	-1.07314	5.449038
9	6	C	1.358596	-1.07864	8.805687
10	6	C	3.305765	1.018487	8.118616
11	6	C	2.538246	2.072354	5.462667
12	6	C	-6.227873	0.300758	1.08733
13	6	C	-6.493348	2.83232	-0.371672
14	6	C	-4.078513	3.89787	-1.560269
15	8	O	-8.012713	-1.13015	1.14584
16	6	C	-3.171081	-4.67271	8.352281
17	8	O	-2.922092	-3.846061	-0.295698
18	1	H	-1.26757	2.265337	6.62658
19	6	C	2.827224	4.926748	5.202288
20	8	O	3.907165	0.817856	3.506958
21	1	H	1.265326	-2.626654	5.080579
22	8	O	-3.14395	2.572295	-3.510982
23	6	C	-4.564352	0.46242	-4.523681
24	6	C	-7.302145	1.277855	-4.974811
25	6	C	-8.425792	2.498501	-2.547494
26	8	O	-3.079792	5.84334	-0.926595
27	6	C	-8.929281	-0.852311	-6.005832
28	6	C	-9.022906	-3.295174	-4.54614
29	6	C	-10.228291	-0.520139	-8.151801
30	6	C	-3.154645	-0.142966	-7.016645
31	6	C	-0.953626	-1.982167	-6.914826
32	8	O	-3.822444	0.858605	-8.957729
33	6	C	-1.989454	-4.699431	-6.606992
34	6	C	0.994071	-1.280192	-4.880098
35	1	H	-4.402902	-1.131861	-3.202061
36	6	C	-7.499255	4.706208	1.619228
37	6	C	0.027704	-6.74075	-6.934246
38	1	H	-2.131568	3.313819	2.428856
39	1	H	-0.291896	0.853375	1.142115
40	1	H	-6.54646	-2.05243	5.428605
41	1	H	-4.841391	0.570196	6.706796

42	1	H	2.258579	-2.786372	9.571669
43	1	H	0.0285	-0.381121	10.249356
44	1	H	3.324699	2.529005	9.5468
45	1	H	5.236488	0.256318	8.003965
46	1	H	-4.923283	-5.731041	7.993891
47	1	H	-1.623405	-6.030233	8.636505
48	1	H	-3.402804	-3.571035	10.099232
49	1	H	2.234673	5.547251	3.30743
50	1	H	4.813902	5.496738	5.479873
51	1	H	1.68129	5.935081	6.616077
52	1	H	5.634554	1.405564	3.5448
53	1	H	-7.178246	2.73218	-6.450023
54	1	H	-10.000167	1.369947	-1.810811
55	1	H	-9.183927	4.373371	-3.021063
56	1	H	-7.17652	-4.259693	-4.580473
57	1	H	-9.47503	-2.985106	-2.54085
58	1	H	-10.425907	-4.594566	-5.357574
59	1	H	-11.446212	-2.007603	-8.91107
60	1	H	-10.094402	1.236102	-9.230438
61	1	H	-0.072058	-1.834276	-8.796595
62	1	H	-2.853234	-4.894876	-4.724802
63	1	H	-3.500417	-4.992169	-8.013626
64	1	H	1.565125	0.711674	-5.031486
65	1	H	0.241042	-1.602248	-2.971563
66	1	H	2.697608	-2.452442	-5.087861
67	1	H	-9.362153	4.079448	2.291722
68	1	H	-6.218767	4.859742	3.248756
69	1	H	-7.662867	6.598073	0.781662
70	1	H	1.467888	-6.643176	-5.436952
71	1	H	-0.825214	-8.636975	-6.849542
72	1	H	0.999814	-6.560921	-8.768634

2*R*,4*S*,5*S*,17*S*,19*S*,20*R*,23*R*,24*S*,*P*-**4** (4)_		Standard Orientation (A.U.)			
Center number	Atomic number	Atomic Type	X	Y	Z
1	6	C	-1.749596	1.102099	3.996403
2	6	C	-4.272472	-0.077446	2.913762
3	6	C	-3.740594	-2.852366	2.227476
4	8	O	-4.314639	-4.312117	4.223989
5	6	C	-5.018901	-2.730299	6.403899
6	6	C	-2.505307	-1.945422	7.672558
7	6	C	-1.639917	0.68958	6.843446
8	6	C	-6.211283	-0.463554	5.054141
9	6	C	-2.224704	-1.752789	10.548371
10	6	C	0.259763	-0.218265	10.872012
11	6	C	0.894235	0.974519	8.256677
12	6	C	-5.364937	1.584222	0.815753
13	6	C	-4.208318	1.955148	-1.872861
14	6	C	-1.807428	0.417735	-2.416794
15	8	O	-7.195866	2.862735	1.320897
16	6	C	-6.780028	-4.277213	8.039622
17	8	O	-2.911609	-3.693601	0.290175
18	1	H	-2.930048	2.064623	7.734327
19	6	C	1.849496	3.689296	8.411304
20	8	O	2.77893	-0.607932	7.153963
21	1	H	-1.076611	-3.303823	7.008475
22	8	O	-1.783992	-1.229964	-4.351075
23	6	C	-3.860127	-1.721269	-5.998552
24	6	C	-6.461477	-1.139053	-4.86164
25	6	C	-6.320889	1.566019	-3.863325
26	8	O	0.131742	0.796762	-1.284005
27	6	C	-7.507399	-3.122615	-3.051662
28	6	C	-6.760602	-5.822867	-3.53424
29	6	C	-9.1477	-2.534167	-1.211738
30	6	C	-3.533016	-0.304733	-8.53013
31	6	C	-1.127828	1.204109	-8.983017
32	8	O	-5.172519	-0.525815	-10.112813
33	6	C	-1.482843	3.160262	-11.086174
34	6	C	1.024227	-0.673712	-9.558518
35	1	H	-3.69849	-3.735802	-6.475196
36	6	C	-3.381066	4.769471	-1.941734
37	6	C	-3.336692	5.269885	-10.411249
38	1	H	-1.69396	3.132113	3.556145
39	1	H	-0.142538	0.241802	3.010646
40	1	H	-8.056159	-0.978954	4.254741
41	1	H	-6.476099	1.196683	6.260188

42	1	H	-2.148731	-3.603285	11.488298
43	1	H	-3.83436	-0.708864	11.361055
44	1	H	0.069638	1.246957	12.332753
45	1	H	1.853086	-1.435967	11.408763
46	1	H	-7.454517	-3.136701	9.64035
47	1	H	-8.42177	-4.909644	6.933032
48	1	H	-5.804686	-5.951296	8.791392
49	1	H	2.27697	4.432493	6.510877
50	1	H	3.595933	3.772391	9.537759
51	1	H	0.435432	4.943355	9.28125
52	1	H	3.196628	0.052355	5.50189
53	1	H	-7.727057	-1.157577	-6.520194
54	1	H	-8.117026	2.215569	-3.0594
55	1	H	-5.936296	2.826876	-5.473483
56	1	H	-7.063158	-6.345255	-5.531109
57	1	H	-4.74961	-6.111076	-3.084753
58	1	H	-7.864867	-7.123553	-2.351336
59	1	H	-9.967582	-4.020921	-0.034744
60	1	H	-9.760994	-0.607796	-0.82216
61	1	H	-0.631582	2.178803	-7.210098
62	1	H	0.383155	3.978092	-11.52658
63	1	H	-2.131811	2.180136	-12.804909
64	1	H	2.805815	0.360322	-9.845732
65	1	H	1.305997	-2.006439	-7.990859
66	1	H	0.612913	-1.747032	-11.294165
67	1	H	-1.921783	5.14953	-0.518179
68	1	H	-2.595395	5.233916	-3.809511
69	1	H	-5.035195	5.969215	-1.583248
70	1	H	-2.744743	6.277038	-8.684331
71	1	H	-3.465565	6.676381	-11.939044
72	1	H	-5.25016	4.518007	-10.095879

2*R*,4*S*,5*S*,17*S*,19*S*,20*R*,23*R*,24*S*,*P*-**4** (5)		Standard Orientation (A.U.)			
Center number	Atomic number	Atomic Type	X	Y	Z
1	6	C	-0.801847	1.219792	4.654154
2	6	C	-3.552279	0.493083	3.718657
3	6	C	-3.476896	-2.257447	2.816312
4	8	O	-4.018182	-3.780925	4.763568
5	6	C	-4.365458	-2.28661	7.098152
6	6	C	-1.693825	-1.909488	8.213933
7	6	C	-0.554049	0.640914	7.45999
8	6	C	-5.353423	0.204921	5.995955
9	6	C	-1.205441	-1.932012	11.067915
10	6	C	1.462309	-0.720317	11.296438
11	6	C	2.080896	0.526974	8.703174
12	6	C	-4.500448	2.49576	1.851065
13	6	C	-4.213943	2.246401	-1.043455
14	6	C	-1.656841	1.119469	-1.790651
15	8	O	-5.59325	4.307486	2.720573
16	6	C	-6.185573	-3.735461	8.758218
17	8	O	-3.011282	-3.050367	0.732093
18	1	H	-1.593545	2.114998	8.506797
19	6	C	3.382122	3.088836	8.919928
20	8	O	3.67114	-1.214939	7.395922
21	1	H	-0.502575	-3.393218	7.373163
22	8	O	-1.542184	-0.344488	-3.864688
23	6	C	-3.652167	-1.781338	-4.776982
24	6	C	-6.112142	-0.230777	-4.714949
25	6	C	-6.518563	0.720537	-2.003784
26	8	O	0.274156	1.783299	-0.780234
27	6	C	-8.382862	-1.685015	-5.693086
28	6	C	-8.86657	-4.275701	-4.617936
29	6	C	-9.87503	-0.650576	-7.456738
30	6	C	-3.049606	-2.619163	-7.47317
31	6	C	-1.627897	-0.785787	-9.171729
32	8	O	-3.766788	-4.667074	-8.202602
33	6	C	-2.829381	-0.757432	-11.817189
34	6	C	1.179278	-1.550329	-9.171178
35	1	H	-3.826184	-3.467629	-3.583506
36	6	C	-4.232048	4.928159	-2.200461
37	6	C	-1.666741	1.179047	-13.613057
38	1	H	-0.466958	3.231916	4.27342
39	1	H	0.579018	0.167571	3.51867
40	1	H	-7.314627	-0.017569	5.341502
41	1	H	-5.299959	1.797584	7.314022

42	1	H	-1.290383	-3.833921	11.89766
43	1	H	-2.621622	-0.76151	12.050431
44	1	H	1.531074	0.682581	12.827205
45	1	H	2.927523	-2.139825	11.680172
46	1	H	-5.363611	-5.556186	9.330826
47	1	H	-6.614769	-2.642058	10.472595
48	1	H	-7.961172	-4.10287	7.74208
49	1	H	2.200166	4.454523	9.952591
50	1	H	3.769682	3.885262	7.032516
51	1	H	5.196424	2.89075	9.917493
52	1	H	4.125246	-0.484654	5.78371
53	1	H	-5.838688	1.419737	-5.952629
54	1	H	-6.868132	-0.910711	-0.771267
55	1	H	-8.203615	1.934295	-1.922566
56	1	H	-7.436625	-5.613418	-5.325451
57	1	H	-8.749359	-4.293302	-2.538451
58	1	H	-10.740928	-4.977962	-5.172387
59	1	H	-11.550232	-1.634088	-8.1605
60	1	H	-9.469257	1.210221	-8.260812
61	1	H	-1.76506	1.116625	-8.331589
62	1	H	-2.699632	-2.674687	-12.619041
63	1	H	-4.868738	-0.373276	-11.606384
64	1	H	2.314468	-0.201929	-10.272312
65	1	H	1.930847	-1.586825	-7.234642
66	1	H	1.408413	-3.443139	-10.006158
67	1	H	-5.963465	5.919011	-1.635395
68	1	H	-2.59873	6.014359	-1.523006
69	1	H	-4.138895	4.833408	-4.272713
70	1	H	-1.749215	3.102128	-12.812045
71	1	H	0.328447	0.759559	-14.027843
72	1	H	-2.682553	1.211486	-15.429066

2*R*,4*S*,5*S*,17*S*,19*S*,20*R*,23*R*,24*S*,*P*-**4** (6)		Standard Orientation (A.U.)			
Center number	Atomic number	Atomic Type	X	Y	Z
1	6	C	-1.401537	1.748167	4.489583
2	6	C	-3.809271	0.266227	3.498755
3	6	C	-2.89945	-2.276745	2.452929
4	8	O	-2.934458	-3.993992	4.311629
5	6	C	-3.698267	-2.803155	6.721017
6	6	C	-1.259452	-1.686346	7.872113
7	6	C	-0.957643	1.124702	7.260599
8	6	C	-5.41139	-0.678313	5.746865
9	6	C	-0.757936	-1.70386	10.724106
10	6	C	1.428356	0.237138	11.024335
11	6	C	1.603603	1.751604	8.505603
12	6	C	-5.348015	1.975883	1.734752
13	6	C	-5.016947	1.986314	-1.165026
14	6	C	-2.242085	1.757145	-1.933924
15	8	O	-6.944867	3.305764	2.690827
16	6	C	-4.970986	-4.825056	8.290043
17	8	O	-2.221426	-2.777211	0.336165
18	1	H	-2.383596	2.156472	8.379281
19	6	C	2.067894	4.572693	8.86516
20	8	O	3.631492	0.649576	7.109384
21	1	H	0.320013	-2.691746	6.964549
22	8	O	-1.686843	0.518556	-4.077905
23	6	C	-3.238488	-1.438496	-5.132277
24	6	C	-6.07325	-0.785749	-4.984738
25	6	C	-6.721624	-0.136571	-2.229609
26	8	O	-0.608218	2.932455	-0.865146
27	6	C	-7.793025	-2.859237	-5.969851
28	6	C	-7.404814	-5.500364	-4.983333
29	6	C	-9.618379	-2.290916	-7.627356
30	6	C	-2.339128	-1.948652	-7.837358
31	6	C	-0.666431	-0.012953	-9.149528
32	8	O	-3.017421	-3.885396	-8.854786
33	6	C	-2.211261	2.419794	-9.654353
34	6	C	0.494915	-1.137238	-11.539201
35	1	H	-2.868842	-3.162032	-4.032528
36	6	C	-5.889824	4.581649	-2.178503
37	6	C	-0.648372	4.590293	-10.743656
38	1	H	-1.70003	3.783702	4.218637
39	1	H	0.22302	1.230968	3.308002
40	1	H	-7.21716	-1.454725	5.068738
41	1	H	-5.831758	0.782652	7.14897

42	1	H	-0.263185	-3.583202	11.456452
43	1	H	-2.448053	-1.057507	11.756953
44	1	H	1.101541	1.508917	12.634362
45	1	H	3.256296	-0.699586	11.325315
46	1	H	-3.62544	-6.333462	8.772742
47	1	H	-5.698199	-4.006055	10.056053
48	1	H	-6.558012	-5.665048	7.242951
49	1	H	2.17126	5.545464	7.024437
50	1	H	3.868948	4.883109	9.857802
51	1	H	0.540037	5.458731	9.964536
52	1	H	3.817666	1.568926	5.541259
53	1	H	-6.395139	0.904605	-6.150768
54	1	H	-6.531433	-1.853976	-1.083247
55	1	H	-8.703564	0.474919	-2.104652
56	1	H	-5.676158	-6.30716	-5.814098
57	1	H	-7.181721	-5.5442	-2.912902
58	1	H	-9.000659	-6.729285	-5.490684
59	1	H	-10.936729	-3.727243	-8.311198
60	1	H	-9.848051	-0.375448	-8.37015
61	1	H	0.829259	0.492495	-7.788476
62	1	H	-3.786746	1.955787	-10.942446
63	1	H	-3.066716	3.058376	-7.870146
64	1	H	1.506228	-2.902044	-11.113927
65	1	H	-0.979209	-1.593506	-12.933568
66	1	H	1.842369	0.18495	-12.405564
67	1	H	-5.805809	4.629513	-4.253228
68	1	H	-7.836492	4.944727	-1.564529
69	1	H	-4.67197	6.090508	-1.438511
70	1	H	-1.806648	6.308543	-10.938113
71	1	H	0.957231	5.046658	-9.497508
72	1	H	0.114701	4.138618	-12.6246

2*R*,4*S*,5*S*,17*S*,19*S*,20*R*,23*R*,24*S*,*P*-**4** (7)		Standard Orientation (A.U.)			
Center number	Atomic number	Atomic Type	X	Y	Z
1	6	C	-1.607357	0.731834	2.37286
2	6	C	-4.224124	-0.595146	2.610014
3	6	C	-3.886538	-3.418665	1.8852
4	8	O	-3.494937	-4.787929	3.979929
5	6	C	-3.172657	-3.100331	6.161583
6	6	C	-0.37689	-2.238186	6.04387
7	6	C	-0.090933	0.357041	4.797224
8	6	C	-4.948912	-0.920204	5.427042
9	6	C	1.27109	-1.908704	8.402989
10	6	C	3.568069	-0.372436	7.394282
11	6	C	2.794707	0.741577	4.759449
12	6	C	-6.480859	0.396414	1.053192
13	6	C	-6.481298	2.85838	-0.551849
14	6	C	-3.950687	3.713329	-1.636592
15	8	O	-8.451865	-0.745777	1.264765
16	6	C	-3.903824	-4.562316	8.505479
17	8	O	-3.891653	-4.310405	-0.202928
18	1	H	-0.805181	1.732795	6.193154
19	6	C	3.584169	3.47566	4.363462
20	8	O	3.933844	-0.632819	2.739329
21	1	H	0.549121	-3.673095	4.844336
22	8	O	-2.996842	2.207446	-3.440215
23	6	C	-4.436118	0.079867	-4.327589
24	6	C	-7.075603	1.038933	-5.112815
25	6	C	-8.270967	2.509893	-2.850582
26	8	O	-2.871465	5.638311	-1.074574
27	6	C	-8.842537	-0.990512	-6.099665
28	6	C	-9.238818	-3.305148	-4.496114
29	6	C	-10.045464	-0.634374	-8.297317
30	6	C	-2.959898	-1.257108	-6.428006
31	6	C	-0.359154	-0.333962	-7.276705
32	8	O	-3.931615	-3.094146	-7.386212
33	6	C	1.643734	-0.264259	-5.160443
34	6	C	-0.562627	2.208423	-8.669702
35	1	H	-4.580635	-1.341114	-2.811419
36	6	C	-7.49352	4.897743	1.262975
37	6	C	1.844981	-2.703607	-3.626192
38	1	H	-1.856907	2.762034	2.075601
39	1	H	-0.569853	-0.001573	0.730113
40	1	H	-6.924899	-1.533997	5.578888
41	1	H	-4.674053	0.788185	6.568908

42	1	H	1.826619	-3.709914	9.27548
43	1	H	0.241453	-0.814508	9.84499
44	1	H	4.112334	1.134505	8.717473
45	1	H	5.245716	-1.571133	7.129139
46	1	H	-5.850093	-5.271071	8.338896
47	1	H	-2.631688	-6.181495	8.784553
48	1	H	-3.779181	-3.336469	10.178973
49	1	H	5.655505	3.639188	4.438774
50	1	H	2.76162	4.70413	5.825125
51	1	H	2.953466	4.145566	2.498377
52	1	H	3.564784	-2.409516	2.936145
53	1	H	-6.742665	2.397596	-6.652474
54	1	H	-9.973128	1.542346	-2.170946
55	1	H	-8.854114	4.395107	-3.497848
56	1	H	-9.753682	-2.816366	-2.540372
57	1	H	-10.731039	-4.51137	-5.291322
58	1	H	-7.486167	-4.422061	-4.404263
59	1	H	-11.389529	-2.025564	-9.022983
60	1	H	-9.709218	1.04394	-9.456101
61	1	H	0.203988	-1.792564	-8.653577
62	1	H	3.477236	0.137917	-6.070145
63	1	H	1.262617	1.338199	-3.897354
64	1	H	-1.972446	2.122116	-10.1987
65	1	H	-1.080239	3.743261	-7.370279
66	1	H	1.272296	2.684758	-9.529512
67	1	H	-9.418289	4.404071	1.865131
68	1	H	-6.28771	5.075588	2.947108
69	1	H	-7.507251	6.74341	0.314192
70	1	H	2.42315	-4.312377	-4.816125
71	1	H	3.241053	-2.478314	-2.10079
72	1	H	0.034092	-3.226204	-2.742005

2*R*,4*S*,5*S*,17*S*,19*S*,20*R*,23*R*,24*S*,*P*-**4** (8)		Standard Orientation (A.U.)			
Center number	Atomic number	Atomic Type	X	Y	Z
1	6	C	-1.796791	1.063248	3.540716
2	6	C	-4.075069	-0.746039	3.000418
3	6	C	-2.932222	-3.321059	2.181868
4	8	O	-2.644117	-4.786187	4.225609
5	6	C	-3.325482	-3.38428	6.529169
6	6	C	-0.930206	-1.89116	7.281722
7	6	C	-0.99976	0.824682	6.29754
8	6	C	-5.358081	-1.589696	5.487673
9	6	C	-0.169728	-1.46842	10.043739
10	6	C	1.818474	0.688018	9.855823
11	6	C	1.575114	1.87152	7.169691
12	6	C	-6.043423	0.015502	0.991914
13	6	C	-5.815684	2.461257	-0.621476
14	6	C	-3.133539	2.991585	-1.562559
15	8	O	-7.908714	-1.291648	0.78799
16	6	C	-4.224761	-5.295525	8.455026
17	8	O	-2.326815	-3.951263	0.08801
18	1	H	-2.419942	1.845537	7.436099
19	6	C	1.734717	4.748281	7.129959
20	8	O	3.571773	0.814324	5.695549
21	1	H	0.660608	-2.854079	6.348114
22	8	O	-2.248208	1.334966	-3.271613
23	6	C	-3.913209	-0.497953	-4.406279
24	6	C	-6.328756	0.885191	-5.271013
25	6	C	-7.540795	2.264582	-2.979129
26	8	O	-1.857408	4.767476	-0.931596
27	6	C	-8.234364	-0.786772	-6.608826
28	6	C	-8.989187	-3.209202	-5.32245
29	6	C	-9.206785	-0.052963	-8.827802
30	6	C	-2.535577	-1.73132	-6.627448
31	6	C	-0.257537	-0.396976	-7.767708
32	8	O	-3.303694	-3.738265	-7.413284
33	6	C	-0.118863	-0.847262	-10.625053
34	6	C	2.106592	-1.371103	-6.374427
35	1	H	-4.302095	-2.006667	-3.038851
36	6	C	-6.751087	4.615895	1.100395
37	6	C	-2.295765	0.372418	-12.079409
38	1	H	-2.288808	3.027754	3.132588
39	1	H	-0.229895	0.576765	2.262059
40	1	H	-7.092768	-2.638984	5.048515
41	1	H	-5.820624	-0.033136	6.776609

42	1	H	0.58705	-3.173556	10.955882
43	1	H	-1.816001	-0.844993	11.159108
44	1	H	1.530909	2.123085	11.330415
45	1	H	3.755335	-0.032617	10.050992
46	1	H	-4.856031	-4.326699	10.181416
47	1	H	-5.807996	-6.404594	7.692366
48	1	H	-2.686934	-6.597962	8.962249
49	1	H	0.231533	5.60608	8.284566
50	1	H	1.53254	5.472262	5.185124
51	1	H	3.575693	5.377207	7.865342
52	1	H	3.56835	1.623406	4.057349
53	1	H	-5.684929	2.330283	-6.624557
54	1	H	-9.277478	1.297273	-2.39316
55	1	H	-8.071488	4.185731	-3.561348
56	1	H	-7.424824	-4.575721	-5.455364
57	1	H	-9.386721	-2.920239	-3.300835
58	1	H	-10.663173	-4.047194	-6.223108
59	1	H	-10.639656	-1.18194	-9.798138
60	1	H	-8.595583	1.690883	-9.7532
61	1	H	-0.433407	1.640962	-7.382081
62	1	H	1.703558	-0.106033	-11.31482
63	1	H	-0.104802	-2.901075	-10.96753
64	1	H	3.802838	-0.384155	-7.0652
65	1	H	1.958134	-1.061094	-4.325658
66	1	H	2.349558	-3.410263	-6.709953
67	1	H	-6.432023	6.443657	0.169758
68	1	H	-8.781756	4.374375	1.467847
69	1	H	-5.759103	4.662144	2.922577
70	1	H	-2.120236	0.039046	-14.126072
71	1	H	-4.131692	-0.399458	-11.475299
72	1	H	-2.340466	2.435489	-11.780656

2*R*,4*S*,5*S*,17*S*,19*S*,20*R*,23*R*,24*S*,*P*-**4** (9)		Standard Orientation (A.U.)			
Center number	Atomic number	Atomic Type	X	Y	Z
1	6	C	-0.807908	1.298598	4.16784
2	6	C	-3.468949	0.142391	3.416726
3	6	C	-2.981105	-2.425013	2.178074
4	8	O	-3.006856	-4.224878	3.967785
5	6	C	-3.358727	-3.082191	6.486932
6	6	C	-0.685695	-2.307789	7.383073
7	6	C	-0.125771	0.480327	6.837054
8	6	C	-4.904406	-0.729314	5.803578
9	6	C	0.141627	-2.522295	10.149955
10	6	C	2.578617	-0.883851	10.240267
11	6	C	2.61118	0.790963	7.793304
12	6	C	-5.006074	2.089297	1.91623
13	6	C	-5.016899	2.217735	-1.000206
14	6	C	-2.39043	1.752736	-2.108558
15	8	O	-6.329999	3.521094	3.111512
16	6	C	-4.673784	-5.020054	8.125187
17	8	O	-2.596589	-2.895566	-0.015906
18	1	H	-1.295242	1.578787	8.168136
19	6	C	3.358333	3.523988	8.276564
20	8	O	4.388134	-0.128846	5.994655
21	1	H	0.607487	-3.491598	6.248848
22	8	O	-2.217793	0.551787	-4.338319
23	6	C	-4.075205	-1.194148	-5.250539
24	6	C	-6.781197	-0.225733	-4.779068
25	6	C	-7.055012	0.344788	-1.94365
26	8	O	-0.534394	2.722685	-1.21376
27	6	C	-8.822713	-2.031745	-5.671716
28	6	C	-8.684962	-4.738949	-4.804011
29	6	C	-10.682799	-1.181886	-7.162286
30	6	C	-3.547606	-1.728555	-8.046123
31	6	C	-2.119926	0.157664	-9.695246
32	8	O	-4.336264	-3.694682	-8.919611
33	6	C	0.736911	0.259465	-9.092642
34	6	C	-3.325248	2.795236	-9.678507
35	1	H	-3.790483	-2.978486	-4.22917
36	6	C	-5.714338	4.940835	-1.791261
37	6	C	1.964863	-2.338216	-8.812897
38	1	H	-0.903188	3.366428	4.031161
39	1	H	0.628625	0.696231	2.800655
40	1	H	-6.855186	-1.26104	5.319746
41	1	H	-4.985695	0.696936	7.29838

42	1	H	0.46272	-4.480561	10.764417
43	1	H	-1.323532	-1.716216	11.391567
44	1	H	2.643436	0.28685	11.956
45	1	H	4.297911	-2.052601	10.245841
46	1	H	-6.468442	-5.605701	7.255967
47	1	H	-3.48088	-6.703343	8.376278
48	1	H	-5.073792	-4.214724	9.998585
49	1	H	5.292476	3.609115	9.035257
50	1	H	2.064562	4.419287	9.63573
51	1	H	3.319281	4.603506	6.500304
52	1	H	4.0101	-1.874323	5.619387
53	1	H	-7.007834	1.552795	-5.83037
54	1	H	-6.939781	-1.441162	-0.894976
55	1	H	-8.930913	1.157738	-1.570687
56	1	H	-8.328423	-4.890828	-2.757632
57	1	H	-10.4471	-5.751691	-5.232684
58	1	H	-7.123949	-5.70695	-5.781322
59	1	H	-12.214209	-2.420738	-7.78614
60	1	H	-10.73199	0.777276	-7.820548
61	1	H	-2.330116	-0.657173	-11.601011
62	1	H	1.644304	1.306955	-10.651967
63	1	H	1.043205	1.367502	-7.362895
64	1	H	-5.340762	2.721979	-10.193647
65	1	H	-3.145598	3.704833	-7.819422
66	1	H	-2.366157	4.012692	-11.067539
67	1	H	-7.527677	5.46694	-0.935033
68	1	H	-4.263265	6.272575	-1.137523
69	1	H	-5.861025	5.092615	-3.857221
70	1	H	1.574159	-3.553037	-10.458988
71	1	H	4.028548	-2.168379	-8.611246
72	1	H	1.258221	-3.330572	-7.124169

TABLE S10 Gibbs free energies[a] and equilibrium populations[b] of low-energy conformers of 2*S*,4*R*,5*S*,12*R*,17*R*,19*S*,20*R*,23*S*,24*R*,*M*-**7**.

Conformers	ΔG (a.u.)	*P*(%)/100	G (a.u.)
2*S*,4*R*,5*S*,12*R*,17*R*,19*S*,20*R*,23*S*,24*R*,*M*-**7**(1)_tddft_	0.00393	1.52	-1577.85297
2*S*,4*R*,5*S*,12*R*,17*R*,19*S*,20*R*,23*S*,24*R*,*M*-**7**(2)_tddft_	0.0	97.55	-1577.856896
2*S*,4*R*,5*S*,12*R*,17*R*,19*S*,20*R*,23*S*,24*R*,*M*-**7**(3)_tddft_	0.00447	0.86	-1577.852426
2*S*,4*R*,5*S*,12*R*,17*R*,19*S*,20*R*,23*S*,24*R*,*M*-**7**(4)_tddft_	0.00685	0.07	-1577.85005

[a]wB97M-V/def2-TZVP, in a.u. [b]From ΔG values at 298.15 K.

TABLE S11 Cartesian coordinates for the low-energy reoptimized random research conformers of 2*S*,4*R*,5*S*,12*R*,17*R*,19*S*,20*R*,23*S*,24*R*,*M*-**7** at B3LYP-D3(BJ)/6-31G* level of theory in methanol.

2*S*,4*R*,5*S*,12*R*,17*R*,19*S*,20*R*,23*S*,24*R*,*M*-**7**(1)			Standard Orientation (A.U.)		
Center number	Atomic number	Atomic Type	X	Y	Z
1	6	C	0.843341	-3.375218	-4.924897
2	6	C	1.56686	-1.144778	-3.142667
3	6	C	3.548877	0.449471	-4.569621
4	8	O	5.874176	-0.432478	-3.998094
5	6	C	5.68775	-2.663385	-2.360497
6	6	C	5.298731	-5.005145	-4.021493
7	6	C	3.244655	-4.540898	-6.009804
8	6	C	3.189037	-2.169014	-0.95385
9	6	C	7.440109	-6.099231	-5.630041
10	6	C	6.025148	-7.759227	-7.60159
11	6	C	3.166531	-7.092364	-7.413783
12	6	C	8.016036	-2.742784	-0.703393
13	8	O	3.172298	2.136764	-6.036136
14	1	H	4.027242	-3.186879	-7.39129
15	6	C	1.826518	-6.985663	-9.961799
16	8	O	2.046417	-8.998371	-5.860493
17	1	H	4.610533	-6.515881	-2.76345
18	6	C	-0.788599	0.342436	-2.376442
19	6	C	-0.637747	2.735069	-0.680633
20	6	C	-1.127246	1.820265	2.061588
21	6	C	-3.735502	2.73485	2.86754
22	6	C	-4.04106	5.218632	1.395776
23	6	C	-2.935781	4.485239	-1.192752
24	8	O	-3.904704	3.02515	5.51737
25	6	C	-4.624156	0.964683	6.875561
26	6	C	-4.641041	1.502449	9.689989
27	8	O	-5.178131	-1.020806	5.911972
28	8	O	0.359289	0.731957	3.396561
29	6	C	-6.680301	6.328971	1.408884
30	6	C	-8.131484	6.440234	-0.662494
31	6	C	-7.572932	7.358848	3.910946
32	6	C	1.853592	4.227992	-0.720026
33	8	O	-2.851346	-0.435455	-2.993181
34	6	C	-7.134829	0.580182	10.835705
35	6	C	-2.343621	0.214983	10.940327
36	6	C	0.211446	1.192615	10.019565
37	1	H	-0.412204	-2.674163	-6.422546
38	1	H	-0.235007	-4.754031	-3.800523
39	1	H	3.436029	-0.781399	0.564418

40	1	H	2.392749	-3.887558	-0.107088
41	1	H	8.468967	-4.551861	-6.568768
42	1	H	8.81795	-7.188138	-4.520241
43	1	H	6.231339	-9.787427	-7.209178
44	1	H	6.729872	-7.418001	-9.525916
45	1	H	9.736389	-2.912997	-1.85585
46	1	H	8.155116	-1.01652	0.446242
47	1	H	7.915124	-4.383321	0.571313
48	1	H	-0.189089	-6.508222	-9.722066
49	1	H	2.671345	-5.54442	-11.201014
50	1	H	1.941908	-8.832246	-10.912133
51	1	H	0.249698	-8.679009	-5.780675
52	1	H	-5.123425	1.323749	2.204617
53	1	H	-2.774814	6.603518	2.318629
54	1	H	-2.393847	6.151404	-2.305384
55	1	H	-4.348121	3.416478	-2.26657
56	1	H	-4.46041	3.561536	9.934502
57	1	H	-10.022166	7.26943	-0.58066
58	1	H	-7.516444	5.724556	-2.495754
59	1	H	-7.672025	5.863043	5.353699
60	1	H	-6.236112	8.78154	4.645308
61	1	H	-9.445126	8.242991	3.743644
62	1	H	1.746043	5.780413	0.659475
63	1	H	3.490765	3.066573	-0.203484
64	1	H	2.179222	5.034145	-2.600058
65	1	H	-8.769998	1.555956	9.996104
66	1	H	-7.368645	-1.458276	10.501508
67	1	H	-7.16017	0.924186	12.88678
68	1	H	-2.516791	0.490628	12.999947
69	1	H	-2.48999	-1.838147	10.605145
70	1	H	1.769357	0.261646	11.038185
71	1	H	0.480428	0.843662	7.987998
72	1	H	0.387732	3.244876	10.336033

2*S*,4*R*,5*S*,12*R*,17*R*,19*S*,20*R*,23*S*,24*R*,*M*-**7**(2)			Standard Orientation (A.U.)		
Center number	Atomic number	Atomic Type	X	Y	Z
1	6	C	1.312248	-1.314555	-5.909345
2	6	C	0.591653	-0.091972	-3.329228
3	6	C	3.110707	0.590745	-2.011455
4	8	O	3.840349	-1.393554	-0.581982
5	6	C	2.09825	-3.520225	-0.891688
6	6	C	2.886694	-5.025981	-3.239457
7	6	C	3.395872	-3.25877	-5.48262
8	6	C	-0.399806	-2.174509	-1.544011
9	6	C	5.253894	-6.690101	-3.247272
10	6	C	5.851595	-7.018051	-6.109826
11	6	C	4.119417	-5.124587	-7.576503
12	6	C	2.056333	-5.02156	1.542452
13	8	O	4.380482	2.441225	-2.323679
14	1	H	5.155955	-2.22724	-5.049501
15	6	C	5.399247	-3.908609	-9.853216
16	8	O	1.8246	-6.324934	-8.342781
17	1	H	1.259723	-6.221726	-3.745448
18	6	C	-1.286713	2.065742	-3.716972
19	6	C	-1.883779	3.991869	-1.593023
20	6	C	-1.408572	3.016587	1.081429
21	6	C	-3.962271	2.577944	2.333028
22	6	C	-5.558011	4.717249	1.258925
23	6	C	-4.749807	4.650523	-1.564017
24	8	O	-3.739613	2.541435	4.990247
25	6	C	-2.806014	0.375449	5.992835
26	6	C	-2.057739	0.721435	8.726939
27	8	O	-2.573578	-1.54469	4.775737
28	8	O	0.598827	2.797476	2.133397
29	6	C	-8.38131	4.564371	1.656314
30	6	C	-9.495707	2.653512	2.884524
31	6	C	-9.865006	6.732361	0.559649
32	6	C	-0.228082	6.349671	-2.082128
33	8	O	-2.382942	2.293285	-5.70888
34	6	C	-2.194729	-1.77834	10.165806
35	6	C	0.590337	1.980477	8.825277
36	6	C	2.74062	0.432405	7.675566
37	1	H	1.925991	0.175684	-7.220445
38	1	H	-0.379452	-2.202607	-6.718888
39	1	H	-1.259918	-1.447955	0.194141
40	1	H	-1.760695	-3.430609	-2.478631
41	1	H	6.809982	-5.69855	-2.284467
42	1	H	4.972712	-8.506072	-2.278531

43	1	H	5.447366	-8.949723	-6.767057
44	1	H	7.857523	-6.644333	-6.509137
45	1	H	3.964691	-5.694831	2.014661
46	1	H	1.323781	-3.872657	3.108612
47	1	H	0.814794	-6.674899	1.312504
48	1	H	7.106002	-2.859865	-9.293426
49	1	H	5.971469	-5.358878	-11.238509
50	1	H	4.093479	-2.59547	-10.799055
51	1	H	2.229496	-7.545976	-9.637424
52	1	H	-4.702815	0.751459	1.647794
53	1	H	-4.875984	6.504623	2.103066
54	1	H	-5.121412	6.445362	-2.533391
55	1	H	-5.843599	3.189658	-2.557711
56	1	H	-3.403282	2.084114	9.54446
57	1	H	-11.547884	2.597162	3.116393
58	1	H	-8.429449	1.096472	3.717475
59	1	H	-9.681315	6.803032	-1.515809
60	1	H	-11.885087	6.590537	1.020752
61	1	H	-9.155089	8.556049	1.28242
62	1	H	-0.69152	7.83477	-0.700761
63	1	H	1.78422	5.881228	-1.926718
64	1	H	-0.623288	7.068581	-3.991203
65	1	H	-1.018352	-3.229491	9.25995
66	1	H	-1.537732	-1.520125	12.122682
67	1	H	-4.142506	-2.505575	10.226284
68	1	H	0.471592	3.817142	7.852985
69	1	H	0.995011	2.3908	10.829667
70	1	H	4.543782	1.44921	7.884285
71	1	H	2.957082	-1.416766	8.605127
72	1	H	2.448182	0.120251	5.643044

2*S*,4*R*,5*S*,12*R*,17*R*,19*S*,20*R*,23*S*,24*R*,*M*-**7**(3)			Standard Orientation (A.U.)		
Center number	Atomic number	Atomic Type	X	Y	Z
1	6	C	0.229711	-3.008825	-5.008638
2	6	C	1.661189	-1.097435	-3.287401
3	6	C	3.935397	-0.134647	-4.838661
4	8	O	5.93728	-1.651945	-4.381365
5	6	C	5.206198	-3.744851	-2.716653
6	6	C	4.063024	-5.86347	-4.330342
7	6	C	2.128685	-4.810676	-6.211358
8	6	C	3.038134	-2.559822	-1.179479
9	6	C	5.701293	-7.523963	-6.040665
10	6	C	3.753676	-8.700429	-7.902889
11	6	C	1.232044	-7.21996	-7.580197
12	6	C	7.498398	-4.509178	-1.188421
13	8	O	3.976394	1.587319	-6.311237
14	1	H	3.198516	-3.734174	-7.64332
15	6	C	-0.14948	-6.705926	-10.055519
16	8	O	-0.318811	-8.716149	-5.949265
17	1	H	3.033525	-7.115049	-3.021336
18	6	C	-0.122329	0.982978	-2.377678
19	6	C	0.816305	3.219622	-0.728118
20	6	C	0.215912	2.388058	2.02494
21	6	C	-2.244002	3.639016	2.794417
22	6	C	-2.13251	6.211715	1.478642
23	6	C	-0.940885	5.549654	-1.11707
24	8	O	-2.455929	3.79348	5.447349
25	6	C	-4.423488	2.615341	6.586223
26	6	C	-4.107784	2.653385	9.430536
27	8	O	-6.130119	1.632695	5.437778
28	8	O	1.519134	1.074143	3.347708
29	6	C	-4.624244	7.600086	1.317173
30	6	C	-4.762013	10.049234	1.942858
31	6	C	-6.889329	6.117968	0.432898
32	6	C	3.616861	3.936221	-0.934495
33	8	O	-2.366315	0.800645	-2.806649
34	6	C	-6.611607	2.080433	10.745335
35	6	C	-1.959797	0.795574	10.134953
36	6	C	-1.131116	0.967721	12.893637
37	1	H	-0.850348	-1.961533	-6.440333
38	1	H	-1.136056	-4.029215	-3.81716
39	1	H	3.762374	-1.311148	0.306776
40	1	H	1.8272	-3.981449	-0.275488
41	1	H	7.07225	-6.33461	-7.060424

42	1	H	6.772064	-8.965728	-4.996187
43	1	H	3.375622	-10.696709	-7.476315
44	1	H	4.421612	-8.595797	-9.867509
45	1	H	9.031985	-5.154374	-2.432832
46	1	H	8.191406	-2.910939	-0.054356
47	1	H	6.992939	-6.066211	0.094228
48	1	H	1.017386	-5.57229	-11.351434
49	1	H	-0.640569	-8.496408	-10.993201
50	1	H	-1.914765	-5.65102	-9.710223
51	1	H	-1.94289	-7.893451	-5.801427
52	1	H	-3.797248	2.486701	2.014586
53	1	H	-0.788886	7.380688	2.560016
54	1	H	0.122318	7.165041	-1.868079
55	1	H	-2.431248	5.080229	-2.475778
56	1	H	-3.45636	4.573533	9.921308
57	1	H	-6.532264	11.104699	1.792824
58	1	H	-3.105863	11.080697	2.625651
59	1	H	-7.457229	4.711963	1.86285
60	1	H	-8.509956	7.366349	0.074642
61	1	H	-6.483768	5.053026	-1.311331
62	1	H	4.044192	5.420109	0.458248
63	1	H	4.884389	2.345722	-0.535296
64	1	H	4.041826	4.660151	-2.828406
65	1	H	-7.27358	0.173458	10.245869
66	1	H	-6.410006	2.193846	12.809592
67	1	H	-8.088447	3.424333	10.167491
68	1	H	-2.59997	-1.14272	9.699855
69	1	H	-0.336707	1.167109	8.88738
70	1	H	-2.663607	0.462896	14.207275
71	1	H	0.458065	-0.322663	13.266894
72	1	H	-0.501952	2.896867	13.367742

2*S*,4*R*,5*S*,12*R*,17*R*,19*S*,20*R*,23*S*,24*R*,*M*-**7**(4)			Standard Orientation (A.U.)		
Center number	Atomic number	Atomic Type	X	Y	Z
1	6	C	0.786651	-3.15161	-4.657464
2	6	C	1.372414	-0.503197	-3.513025
3	6	C	2.786401	1.001788	-5.57718
4	8	O	5.291296	0.587349	-5.312314
5	6	C	5.767602	-1.22093	-3.260717
6	6	C	5.540028	-3.909619	-4.312278
7	6	C	3.152506	-4.190798	-5.928061
8	6	C	3.480404	-0.761839	-1.523174
9	6	C	7.562328	-5.042745	-6.043178
10	6	C	6.162714	-7.273919	-7.352102
11	6	C	3.299507	-6.980562	-6.750464
12	6	C	8.307177	-0.598618	-2.103924
13	8	O	1.887136	2.264023	-7.229878
14	1	H	3.46684	-3.088796	-7.671518
15	6	C	1.568166	-7.642887	-8.955676
16	8	O	2.796724	-8.612672	-4.656228
17	1	H	5.323083	-5.170136	-2.668788
18	6	C	-1.065496	0.776392	-2.637209
19	6	C	-1.003033	3.353645	-1.226184
20	6	C	-0.598394	2.68878	1.59143
21	6	C	-3.177799	2.049733	2.676548
22	6	C	-4.882618	4.075062	1.493582
23	6	C	-3.715325	4.504187	-1.177493
24	8	O	-3.17818	2.113358	5.342472
25	6	C	-2.144943	0.074703	6.520652
26	6	C	-1.858933	0.543264	9.328024
27	8	O	-1.545334	-1.816853	5.404726
28	8	O	1.339934	2.852626	2.768997
29	6	C	-7.670607	3.40516	1.625301
30	6	C	-8.78827	1.924843	-0.095735
31	6	C	-9.034433	4.400012	3.914838
32	6	C	0.996763	5.223892	-2.158146
33	8	O	-3.064576	-0.299795	-2.929349
34	6	C	0.038394	2.700527	9.756335
35	6	C	-4.448616	1.026591	10.572066
36	6	C	-4.348301	1.137153	13.455125
37	1	H	-0.791549	-2.980741	-5.994653
38	1	H	0.157667	-4.381841	-3.100542
39	1	H	3.747052	0.971387	-0.422245
40	1	H	3.142256	-2.317292	-0.19222
41	1	H	8.157818	-3.622907	-7.444158

42	1	H	9.258376	-5.663236	-5.016398
43	1	H	6.781526	-9.119641	-6.630537
44	1	H	6.481095	-7.265733	-9.405265
45	1	H	8.320173	1.342148	-1.359579
46	1	H	8.703422	-1.912209	-0.540928
47	1	H	9.8229	-0.779692	-3.513404
48	1	H	-0.433587	-7.404523	-8.422857
49	1	H	1.93153	-6.41816	-10.597189
50	1	H	1.851186	-9.623236	-9.525167
51	1	H	1.007882	-8.536637	-4.294973
52	1	H	-3.717016	0.158826	1.99407
53	1	H	-4.57074	5.797548	2.624265
54	1	H	-3.61308	6.537731	-1.577302
55	1	H	-4.878964	3.631781	-2.645753
56	1	H	-1.069453	-1.228342	10.083151
57	1	H	-10.780209	1.406545	0.087539
58	1	H	-7.752848	1.142484	-1.700835
59	1	H	-11.023771	3.802292	3.956647
60	1	H	-8.104369	3.731814	5.657469
61	1	H	-8.970474	6.484829	3.974285
62	1	H	0.630122	5.764027	-4.125978
63	1	H	0.946987	6.92549	-0.965693
64	1	H	2.92084	4.462245	-2.043526
65	1	H	-0.749957	4.50529	9.089362
66	1	H	0.506303	2.886038	11.772474
67	1	H	1.802029	2.357776	8.710763
68	1	H	-5.233534	2.802666	9.815231
69	1	H	-5.759494	-0.486768	9.988266
70	1	H	-3.185445	2.723839	14.132121
71	1	H	-6.253876	1.380466	14.25484
72	1	H	-3.548679	-0.614593	14.251034

TABLE S12 Gibbs free energies[a] and equilibrium populations[b] of low-energy conformers of 2*S*,4*R*,5*S*,12*R*,17*R*,19*S*,20*R*,23*S*,24*R*,*P*-**7**.

Conformers	ΔG (a.u.)	*P*(%)/100	G (a.u.)
2*S*,4*R*,5*S*,12*R*,17*R*,19*S*,20*R*,23*S*,24*R*,*P*-**7**(1)_tddft_	0.0	29.55	-1577.855384
2*S*,4*R*,5*S*,12*R*,17*R*,19*S*,20*R*,23*S*,24*R*,*P*-**7**(2)_tddft_	0.00004	12.96	-1577.855348
2*S*,4*R*,5*S*,12*R*,17*R*,19*S*,20*R*,23*S*,24*R*,*P*-**7**(3)_tddft_	0.00209	8.21	-1577.853296
2*S*,4*R*,5*S*,12*R*,17*R*,19*S*,20*R*,23*S*,24*R*,*P*-**7**(4)_tddft_	0.00213	6.97	-1577.853258
2*S*,4*R*,5*S*,12*R*,17*R*,19*S*,20*R*,23*S*,24*R*,*P*-**7**(5)_tddft_	0.00004	12.95	-1577.855348
2*S*,4*R*,5*S*,12*R*,17*R*,19*S*,20*R*,23*S*,24*R*,*P*-**7**(6)_tddft_	0.00186	9.35	-1577.853526
2*S*,4*R*,5*S*,12*R*,17*R*,19*S*,20*R*,23*S*,24*R*,*P*-**7**(7)_tddft_	0.00103	10.95	-1577.854355
2*S*,4*R*,5*S*,12*R*,17*R*,19*S*,20*R*,23*S*,24*R*,*P*-**7**(8)_tddft_	0.00214	6.95	-1577.853241
2*S*,4*R*,5*S*,12*R*,17*R*,19*S*,20*R*,23*S*,24*R*,*P*-**7**(9)_tddft_	0.00343	2.09	-1577.851952

[a]wB97M-V/def2-TZVP, in a.u. [b]From ΔG values at 298.15 K.

TABLE S13 Cartesian coordinates for the low-energy reoptimized random research conformers of 2*S*,4*R*,5*S*,12*R*,17*R*,19*S*,20*R*,23*S*,24*R*,*P*-**7** at B3LYP-D3(BJ)/6-31G* level of theory in methanol.

2*S*,4*R*,5*S*,12*R*,17*R*,19*S*,20*R*,23*S*,24*R*,*P*-**7**(1)			Standard Orientation (A.U.)		
Center number	Atomic number	Atomic Type	X	Y	Z
1	6	C	1.323463	0.21302	-0.49957
2	6	C	1.616043	-1.27299	-0.11568
3	6	C	1.923415	-1.32475	1.403241
4	8	O	3.242722	-1.11784	1.603289
5	6	C	3.924072	-0.88452	0.304613
6	6	C	3.815078	0.607055	-0.04775
7	6	C	2.378328	1.119807	0.151927
8	6	C	3.00947	-1.66409	-0.65658
9	6	C	4.636779	1.677138	0.683406
10	6	C	3.899733	2.997328	0.308727
11	6	C	2.494417	2.609855	-0.25364
12	6	C	0.566264	-2.37066	-0.42326
13	8	O	-0.93283	-2.10417	-0.74379
14	1	H	-1.09187	-1.09349	-1.87256
15	6	C	0.93324	-3.52682	-0.31945
16	8	O	5.339015	-1.41701	0.428713
17	1	H	1.147822	-1.50039	2.316555
18	6	C	2.188348	1.134153	1.235315
19	6	C	1.365269	3.494854	0.274479
20	6	C	2.587968	2.74326	-1.68717
21	6	C	4.048211	0.681641	-1.11845
22	6	C	-2.18938	-0.30437	-1.9053
23	6	C	-3.11944	-0.1046	-0.81089
24	8	O	-3.11262	-1.25654	0.229283
25	6	C	-1.6663	-1.65881	0.548043
26	6	C	-0.32971	-1.05376	-2.81816
27	8	O	-3.97304	-0.92599	1.448135
28	8	O	-5.45696	-1.15147	1.271372
29	6	C	-3.45942	-0.50174	2.608824
30	6	C	-2.80513	1.265384	-0.17317
31	6	C	-3.96749	2.220388	0.04328
32	6	C	-1.65302	1.558955	0.096251
33	8	O	-3.69331	3.206282	1.181716
34	6	C	-4.23859	2.950864	-1.29522
35	6	C	-4.09441	-0.05762	-1.30058
36	6	C	-1.52915	-3.42628	-1.30186
37	1	H	0.320199	0.516738	-0.19231
38	1	H	1.376292	0.289514	-1.58964
39	1	H	3.167715	-2.74003	-0.5505

40	1	H	3.154775	-1.38159	-1.70229
41	1	H	4.599407	1.507572	1.766478
42	1	H	5.690151	1.690932	0.387724
43	1	H	4.43623	3.549351	-0.46874
44	1	H	3.804279	3.661856	1.172777
45	1	H	5.866262	-1.28333	-0.52186
46	1	H	5.893681	-0.88172	1.205431
47	1	H	5.331048	-2.48375	0.67384
48	1	H	1.53523	4.53638	-0.02035
49	1	H	0.391667	3.177858	-0.11747
50	1	H	1.312122	3.451411	1.368095
51	1	H	1.702333	2.582589	-2.05285
52	1	H	-3.59933	-2.10328	-0.26733
53	1	H	-1.12965	-0.83254	1.019274
54	1	H	-1.66377	-2.48743	1.262613
55	1	H	-6.01612	-0.84595	2.160219
56	1	H	-5.86043	-0.60098	0.411442
57	1	H	-5.66437	-2.21343	1.082806
58	1	H	-4.1037	-0.28751	3.457569
59	1	H	-2.39483	-0.3615	2.767616
60	1	H	-4.8563	1.622411	0.281127
61	1	H	-2.80722	3.811746	0.967957
62	1	H	-4.54808	3.878262	1.309742
63	1	H	-3.52564	2.679294	2.12676
64	1	H	-5.10959	3.604214	-1.18294
65	1	H	-4.44397	2.254724	-2.11564
66	1	H	-3.37853	3.568124	-1.57656
67	1	H	-2.5547	-3.28443	-1.65074
68	1	H	-0.93153	-3.7825	-2.14361
69	1	H	-1.52404	-4.19269	-0.52392
70	1	H	-1.11076	0.229403	-0.12153
71	1	H	-1.63973	-1.2431	-0.2227
72	1	H	-2.05856	-1.69769	1.197162

2*S*,4*R*,5*S*,12*R*,17*R*,19*S*,20*R*,23*S*,24*R*,*P*-**7**(2)			Standard Orientation (A.U.)		
Center number	Atomic number	Atomic Type	X	Y	Z
1	6	C	-2.24841	-0.51344	-0.89554
2	6	C	-1.22889	-0.88539	0.271752
3	6	C	-0.62651	0.416276	0.797618
4	8	O	-1.38737	0.896425	1.792524
5	6	C	-2.57929	0.012713	1.994046
6	6	C	-3.64483	0.523337	0.991361
7	6	C	-3.64511	-0.29707	-0.30916
8	6	C	-2.0015	-1.33577	1.537502
9	6	C	-5.14333	0.538599	1.336995
10	6	C	-5.82001	0.71929	-0.05522
11	6	C	-4.74775	0.397146	-1.14455
12	6	C	-0.25294	-1.96676	-0.21846
13	8	O	1.152892	-1.69777	-0.82535
14	1	H	1.207959	-0.46075	-1.71836
15	6	C	-0.60611	-3.12443	-0.07883
16	8	O	-2.95131	0.114195	3.460348
17	1	H	0.374738	0.992845	0.419476
18	6	C	-4.0559	-1.28626	-0.06385
19	6	C	-5.28259	-0.44583	-2.30189
20	6	C	-4.29151	1.671656	-1.6405
21	6	C	-3.36119	1.552974	0.741847
22	6	C	2.360991	0.247341	-1.79446
23	6	C	3.389575	0.234406	-0.76955
24	8	O	3.533101	-1.15664	-0.11696
25	6	C	2.162424	-1.65014	0.355767
26	6	C	0.326037	-0.19391	-2.50975
27	8	O	4.659939	-1.26533	0.905855
28	8	O	6.007694	-0.74815	0.456778
29	6	C	4.51565	-1.8709	2.089139
30	6	C	3.193927	1.454335	0.163526
31	6	C	2.727652	2.762086	-0.47332
32	6	C	3.518433	1.37828	1.334727
33	8	O	2.370351	3.796332	0.59677
34	6	C	3.814436	3.289681	-1.43924
35	6	C	4.303093	0.448075	-1.33551
36	6	C	1.480926	-2.88313	-1.77671
37	1	H	-1.87839	0.374588	-1.41405
38	1	H	-2.25944	-1.32717	-1.62629
39	1	H	-1.30676	-1.72533	2.288712
40	1	H	-2.75739	-2.09755	1.349591
41	1	H	-5.44475	-0.41553	1.78608
42	1	H	-5.41555	1.332376	2.038651

43	1	H	-6.69209	0.067183	-0.16189
44	1	H	-6.16055	1.74778	-0.20696
45	1	H	-2.10299	-0.16682	4.092033
46	1	H	-3.25794	1.133341	3.716172
47	1	H	-3.78498	-0.55993	3.679921
48	1	H	-5.65965	-1.4112	-1.9472
49	1	H	-6.10001	0.079985	-2.80723
50	1	H	-4.49751	-0.64414	-3.04314
51	1	H	-3.6921	1.500683	-2.3856
52	1	H	3.85266	-1.8023	-0.9482
53	1	H	2.240146	-2.66033	0.767371
54	1	H	1.798165	-0.99651	1.153313
55	1	H	6.791353	-1.03049	1.165742
56	1	H	6.282335	-1.14357	-0.53039
57	1	H	6.012364	0.346197	0.37722
58	1	H	5.36492	-1.99189	2.756892
59	1	H	3.56686	-2.25451	2.449436
60	1	H	1.83806	2.533833	-1.06755
61	1	H	3.239344	4.0457	1.214652
62	1	H	2.011638	4.716037	0.122
63	1	H	1.58245	3.41615	1.25332
64	1	H	4.000981	2.598541	-2.26734
65	1	H	3.487526	4.242065	-1.86978
66	1	H	4.760568	3.465277	-0.91371
67	1	H	0.72921	-2.95118	-2.56715
68	1	H	2.458207	-2.75183	-2.24791
69	1	H	1.481263	-3.81801	-1.2137
70	1	H	-3.35466	-1.40199	1.401415
71	1	H	-3.8922	-0.65681	0.225735
72	1	H	-3.5123	0.82998	0.476355

2*S*,4*R*,5*S*,12*R*,17*R*,19*S*,20*R*,23*S*,24*R*,*P*-**7**(3)			Standard Orientation (A.U.)		
Center number	Atomic number	Atomic Type	X	Y	Z
1	6	C	-2.25342	-0.5104	-0.90085
2	6	C	-1.23337	-0.88248	0.265687
3	6	C	-0.63406	0.417551	0.794606
4	8	O	-1.38894	0.893307	1.791863
5	6	C	-2.58127	0.009601	1.99235
6	6	C	-3.64683	0.525844	0.992235
7	6	C	-3.64811	-0.29424	-0.30983
8	6	C	-2.00454	-1.33735	1.530702
9	6	C	-5.14552	0.542519	1.339401
10	6	C	-5.82991	0.699798	-0.05344
11	6	C	-4.74333	0.395308	-1.14315
12	6	C	-0.25537	-1.96094	-0.22629
13	8	O	1.152581	-1.69425	-0.83053
14	1	H	1.213474	-0.45685	-1.72337
15	6	C	-0.60829	-3.11654	-0.08965
16	8	O	-2.95146	0.106111	3.459417
17	1	H	0.348426	0.989123	0.422765
18	6	C	-4.06279	-1.2813	-0.06392
19	6	C	-5.27063	-0.43768	-2.31159
20	6	C	-4.16417	1.616582	-1.6396
21	6	C	-3.36024	1.554963	0.746763
22	6	C	2.369234	0.248931	-1.79527
23	6	C	3.394838	0.232954	-0.76765
24	8	O	3.532707	-1.15924	-0.11618
25	6	C	2.159745	-1.65013	0.353004
26	6	C	0.334377	-0.18835	-2.51982
27	8	O	4.657053	-1.27182	0.908925
28	8	O	6.006577	-0.75564	0.464052
29	6	C	4.509291	-1.87959	2.090692
30	6	C	3.200824	1.45295	0.165911
31	6	C	2.743213	2.763431	-0.4719
32	6	C	3.520447	1.374651	1.33874
33	8	O	2.384445	3.797953	0.597417
34	6	C	3.837363	3.287887	-1.43123
35	6	C	4.310555	0.444614	-1.33086
36	6	C	1.479511	-2.87998	-1.78168
37	1	H	-1.89562	0.376618	-1.42578
38	1	H	-2.26867	-1.32761	-1.62778
39	1	H	-1.3098	-1.72988	2.280268
40	1	H	-2.76069	-2.09842	1.340909
41	1	H	-5.44433	-0.40569	1.802334

42	1	H	-5.41674	1.344737	2.031766
43	1	H	-6.68577	0.024302	-0.15165
44	1	H	-6.20414	1.718306	-0.20152
45	1	H	-2.10235	-0.17682	4.089179
46	1	H	-3.25806	1.1243	3.719096
47	1	H	-3.78467	-0.56897	3.677737
48	1	H	-5.6643	-1.4004	-1.96716
49	1	H	-6.0842	0.091071	-2.82443
50	1	H	-4.47674	-0.62649	-3.04235
51	1	H	-4.86305	2.097479	-2.1123
52	1	H	3.852253	-1.80483	-0.94753
53	1	H	2.234336	-2.661	0.763627
54	1	H	1.795304	-0.99671	1.150546
55	1	H	6.013008	0.338879	0.387059
56	1	H	6.788237	-1.04077	1.174119
57	1	H	6.282862	-1.14913	-0.52342
58	1	H	5.356904	-2.0032	2.760088
59	1	H	3.559128	-2.26248	2.448126
60	1	H	1.85603	2.539445	-1.07108
61	1	H	1.591832	3.419987	1.249477
62	1	H	3.251259	4.043409	1.219954
63	1	H	2.031618	4.719536	0.12181
64	1	H	4.781433	3.458812	-0.90042
65	1	H	4.025516	2.59719	-2.25936
66	1	H	3.516764	4.242259	-1.86217
67	1	H	0.729522	-2.9454	-2.57399
68	1	H	2.458322	-2.7512	-2.25045
69	1	H	1.475807	-3.81523	-1.21927
70	1	H	-2.25554	1.223421	-0.31019
71	1	H	-3.03401	-1.27271	-0.89724
72	1	H	-4.44887	2.002143	0.147953

2*S*,4*R*,5*S*,12*R*,17*R*,19*S*,20*R*,23*S*,24*R*,*P*-**7**(4)		Standard Orientation (A.U.)			
Center number	Atomic number	Atomic Type	X	Y	Z
1	6	C	1.152938	0.193989	0.164736
2	6	C	1.630287	-1.29847	0.121643
3	6	C	1.956891	-1.6531	-1.34978
4	8	O	3.250223	-1.38268	-1.60242
5	6	C	3.877192	-0.75445	-0.40315
6	6	C	3.542591	0.759796	-0.51284
7	6	C	2.343837	1.128891	0.369986
8	6	C	3.056275	-1.42979	0.713245
9	6	C	4.538702	1.868304	-0.14145
10	6	C	3.62777	3.129335	-0.12067
11	6	C	2.15962	2.647529	0.130229
12	6	C	0.659108	-2.38206	0.652729
13	8	O	-0.87677	-2.14724	0.823056
14	1	H	-1.09725	-1.12323	1.937105
15	6	C	1.117571	-3.48441	0.882257
16	8	O	5.351502	-1.10643	-0.43823
17	1	H	1.207664	-2.10148	-2.18971
18	6	C	2.674023	1.035022	1.413492
19	6	C	1.477779	3.371337	1.293518
20	6	C	1.425308	2.892592	-1.08553
21	6	C	3.259775	0.929195	-1.56
22	6	C	-2.17033	-0.29902	1.896978
23	6	C	-3.02659	-0.08634	0.733213
24	8	O	-2.98717	-1.25818	-0.26938
25	6	C	-1.54363	-1.72632	-0.51244
26	6	C	-0.39037	-1.08778	2.925325
27	8	O	-3.78674	-0.95207	-1.53723
28	8	O	-5.23543	-1.37844	-1.50031
29	6	C	-3.25049	-0.3745	-2.61823
30	6	C	-2.61515	1.313477	0.194826
31	6	C	-3.53338	2.47666	0.536159
32	6	C	-1.56899	1.454668	-0.41639
33	8	O	-2.85389	3.827942	0.302928
34	6	C	-4.85947	2.366139	-0.25276
35	6	C	-4.02563	-0.01293	1.167068
36	6	C	-1.49049	-3.46855	1.358006
37	1	H	0.636155	0.44494	-0.76317
38	1	H	0.439269	0.342094	0.973808
39	1	H	3.330132	-2.48411	0.796244
40	1	H	3.176441	-0.96167	1.691705
41	1	H	4.967845	1.687048	0.851994

42	1	H	5.36842	1.967926	-0.84769
43	1	H	3.94854	3.851261	0.636522
44	1	H	3.650718	3.639349	-1.08879
45	1	H	5.48756	-2.19212	-0.46061
46	1	H	5.836459	-0.6726	-1.31842
47	1	H	5.847761	-0.71323	0.454557
48	1	H	2.041494	3.245562	2.224418
49	1	H	1.401857	4.442809	1.078224
50	1	H	0.464093	2.984729	1.459033
51	1	H	0.494049	2.663237	-0.91413
52	1	H	-3.51858	-2.07405	0.233113
53	1	H	-1.54599	-2.58349	-1.19191
54	1	H	-0.97103	-0.93816	-0.99813
55	1	H	-5.31464	-2.46963	-1.40163
56	1	H	-5.76491	-0.95183	-0.63811
57	1	H	-5.76658	-1.07693	-2.4077
58	1	H	-3.85129	-0.18334	-3.50385
59	1	H	-2.20938	-0.0725	-2.66762
60	1	H	-3.77618	2.367629	1.604224
61	1	H	-3.52632	4.639263	0.599729
62	1	H	-1.93096	3.917952	0.884221
63	1	H	-2.60108	3.961929	-0.7539
64	1	H	-4.67758	2.43778	-1.33004
65	1	H	-5.3897	1.429067	-0.05724
66	1	H	-5.52064	3.187698	0.039987
67	1	H	-2.5538	-3.35009	1.579508
68	1	H	-1.37435	-4.25669	0.609834
69	1	H	-0.98281	-3.78164	2.27174
70	1	H	-3.4958	3.225123	0.276939
71	1	H	-2.03272	2.709088	0.066748
72	1	H	-0.67679	-2.31907	-0.78453

2*S*,4*R*,5*S*,12*R*,17*R*,19*S*,20*R*,23*S*,24*R*,*P*-**7**(5)			Standard Orientation (A.U.)		
Center number	Atomic number	Atomic Type	X	Y	Z
1	6	C	-1.1378	0.204974	-0.19682
2	6	C	-1.65527	-1.27219	-0.10048
3	6	C	-1.96036	-1.57242	1.38684
4	8	O	-3.23575	-1.24205	1.659473
5	6	C	-3.87199	-0.63961	0.45127
6	6	C	-3.49161	0.867784	0.494072
7	6	C	-2.3034	1.163186	-0.42901
8	6	C	-3.09771	-1.38185	-0.65604
9	6	C	-4.46179	1.991372	0.100546
10	6	C	-3.51281	3.22078	0.021273
11	6	C	-2.07062	2.687031	-0.27368
12	6	C	-0.7218	-2.38951	-0.62846
13	8	O	0.819749	-2.19776	-0.8127
14	1	H	1.064576	-1.19851	-1.94348
15	6	C	-1.21187	-3.48015	-0.84726
16	8	O	-5.35478	-0.94532	0.533146
17	1	H	-1.21065	-2.02534	2.22423
18	6	C	-2.6568	1.025675	-1.45976
19	6	C	-1.43015	3.332808	-1.50524
20	6	C	-1.26821	2.973124	0.888212
21	6	C	-3.18017	1.068907	1.527765
22	6	C	2.148011	-0.39198	-1.90777
23	6	C	3.019027	-0.18111	-0.76728
24	8	O	2.957308	-1.33994	0.266771
25	6	C	1.500316	-1.76488	0.508692
26	6	C	0.363298	-1.16894	-2.93593
27	8	O	3.726147	-1.03171	1.551879
28	8	O	2.997104	-0.37466	2.701305
29	6	C	5.016202	-1.37598	1.645909
30	6	C	2.713819	1.227656	-0.2074
31	6	C	3.907113	2.115347	0.109103
32	6	C	1.559029	1.602537	-0.08312
33	8	O	4.445716	2.69412	-1.22423
34	6	C	3.551625	3.234436	1.092195
35	6	C	4.023029	-0.16706	-1.19687
36	6	C	1.399243	-3.54342	-1.32178
37	1	H	-0.61512	0.477736	0.723126
38	1	H	-0.42002	0.308927	-1.00678
39	1	H	-3.40465	-2.42972	-0.69221
40	1	H	-3.2264	-0.94908	-1.64945
41	1	H	-4.91342	1.788796	-0.87885

42	1	H	-5.2756	2.141616	0.816353
43	1	H	-3.83891	3.941708	-0.73469
44	1	H	-3.48324	3.745057	0.981738
45	1	H	-5.52242	-2.02485	0.598171
46	1	H	-5.80512	-0.46568	1.407941
47	1	H	-5.86069	-0.5691	-0.36156
48	1	H	-2.04387	3.177167	-2.39936
49	1	H	-1.31795	4.411278	-1.3481
50	1	H	-0.43585	2.911592	-1.69897
51	1	H	-0.3516	2.722656	0.672023
52	1	H	3.482214	-2.16923	-0.21598
53	1	H	1.472262	-2.60466	1.209602
54	1	H	0.946846	-0.95096	0.978557
55	1	H	3.690449	-0.1322	3.51136
56	1	H	2.485146	0.548214	2.40496
57	1	H	2.222825	-1.03777	3.107572
58	1	H	5.595959	-1.16756	2.541442
59	1	H	5.53664	-1.88128	0.835619
60	1	H	4.690565	1.475044	0.535366
61	1	H	5.336518	3.296664	-1.02097
62	1	H	4.724982	1.913586	-1.93926
63	1	H	3.695139	3.338414	-1.69478
64	1	H	4.434328	3.85139	1.288464
65	1	H	2.766692	3.877773	0.682573
66	1	H	3.197277	2.835627	2.048184
67	1	H	0.882315	-3.86111	-2.22864
68	1	H	2.46481	-3.45464	-1.54687
69	1	H	1.265652	-4.31437	-0.55863
70	1	H	0.869394	-2.11325	-0.87023
71	1	H	1.151003	-1.01991	-1.90127
72	1	H	-1.1528	-3.38901	-1.11011

2*S*,4*R*,5*S*,12*R*,17*R*,19*S*,20*R*,23*S*,24*R*,*P*-**7**(6)			Standard Orientation (A.U.)		
Center number	Atomic number	Atomic Type	X	Y	Z
1	6	C	-2.24835	-0.5135	-0.89554
2	6	C	-1.22892	-0.88546	0.271808
3	6	C	-0.62658	0.416165	0.797827
4	8	O	-1.38747	0.896223	1.792738
5	6	C	-2.57944	0.01254	1.994065
6	6	C	-3.64486	0.523283	0.9913
7	6	C	-3.64508	-0.29706	-0.30926
8	6	C	-2.00163	-1.33592	1.537481
9	6	C	-5.14339	0.538674	1.336855
10	6	C	-5.82009	0.718961	-0.05543
11	6	C	-4.74764	0.397245	-1.14466
12	6	C	-0.25289	-1.9668	-0.21834
13	8	O	1.152938	-1.6978	-0.82534
14	1	H	1.207958	-0.46079	-1.71836
15	6	C	-0.60592	-3.12448	-0.07853
16	8	O	-2.95159	0.113909	3.460343
17	1	H	0.374723	0.992729	0.419809
18	6	C	-4.05596	-1.28624	-0.06402
19	6	C	-5.28216	-0.44546	-2.30234
20	6	C	-4.29147	1.671943	-1.64019
21	6	C	-3.36111	1.552899	0.741859
22	6	C	2.360854	0.247475	-1.7944
23	6	C	3.389557	0.234494	-0.76961
24	8	O	3.533175	-1.15658	-0.11709
25	6	C	2.16255	-1.65015	0.355724
26	6	C	0.326008	-0.19405	-2.50977
27	8	O	4.660119	-1.26521	0.905603
28	8	O	6.007752	-0.74781	0.456431
29	6	C	4.515992	-1.87077	2.088911
30	6	C	3.194035	1.454375	0.163589
31	6	C	2.72738	2.762081	-0.47307
32	6	C	3.518893	1.378293	1.334678
33	8	O	2.370328	3.796218	0.597212
34	6	C	3.813734	3.289844	-1.43936
35	6	C	4.303008	0.448219	-1.33566
36	6	C	1.480867	-2.88319	-1.77668
37	1	H	-1.87823	0.374471	-1.41407
38	1	H	-2.25937	-1.32727	-1.62625
39	1	H	-1.30693	-1.72549	2.288725
40	1	H	-2.7575	-2.0977	1.349489
41	1	H	-5.44488	-0.41528	1.786246

42	1	H	-5.41555	1.332692	2.038258
43	1	H	-6.69175	0.066299	-0.16208
44	1	H	-6.16126	1.74722	-0.20729
45	1	H	-2.10334	-0.16719	4.092079
46	1	H	-3.25821	1.133042	3.716228
47	1	H	-3.78531	-0.56021	3.679779
48	1	H	-5.65908	-1.41104	-1.94802
49	1	H	-6.09961	0.080336	-2.80764
50	1	H	-4.49693	-0.64338	-3.04353
51	1	H	-3.69186	1.501258	-2.3852
52	1	H	3.852697	-1.80218	-0.94839
53	1	H	2.24035	-2.66034	0.76732
54	1	H	1.7983	-0.99654	1.153291
55	1	H	6.282226	-1.14282	-0.53094
56	1	H	6.012307	0.346578	0.377279
57	1	H	6.791572	-1.03034	1.165138
58	1	H	5.365328	-1.99171	2.756593
59	1	H	3.567262	-2.25445	2.449297
60	1	H	1.837583	2.53371	-1.06695
61	1	H	3.23947	4.045531	1.214906
62	1	H	2.01148	4.715975	0.122643
63	1	H	1.582596	3.415933	1.25391
64	1	H	4.00009	2.598745	-2.26754
65	1	H	3.486532	4.242184	-1.86979
66	1	H	4.760028	3.46557	-0.91417
67	1	H	0.729087	-2.95127	-2.56706
68	1	H	2.458111	-2.75195	-2.24796
69	1	H	1.481215	-3.81804	-1.21362
70	1	H	-5.37739	-0.95327	0.154612
71	1	H	-1.37053	-2.23462	2.0376
72	1	H	-2.531	1.228133	-1.37357

2*S*,4*R*,5*S*,12*R*,17*R*,19*S*,20*R*,23*S*,24*R*,*P*-**7**(7)			Standard Orientation (A.U.)		
Center number	Atomic number	Atomic Type	X	Y	Z
1	6	C	-1.1094	0.20243	-0.18526
2	6	C	-1.62359	-1.27972	-0.16677
3	6	C	-1.96149	-1.65572	1.297844
4	8	O	-3.24854	-1.36092	1.553602
5	6	C	-3.85759	-0.6926	0.367241
6	6	C	-3.48549	0.810038	0.509103
7	6	C	-2.27944	1.16918	-0.36812
8	6	C	-3.05159	-1.36458	-0.76173
9	6	C	-4.45523	1.949673	0.163713
10	6	C	-3.51487	3.188265	0.174713
11	6	C	-2.06017	2.678944	-0.09969
12	6	C	-0.67828	-2.37611	-0.72005
13	8	O	0.865881	-2.17942	-0.85667
14	1	H	1.129248	-1.1401	-1.94724
15	6	C	-1.16469	-3.4556	-0.99497
16	8	O	-5.34015	-1.00893	0.393116
17	1	H	-1.22616	-2.1403	2.130021
18	6	C	-2.6134	1.103418	-1.41248
19	6	C	-1.37679	3.409929	-1.25753
20	6	C	-1.30962	2.885085	1.113069
21	6	C	-3.19758	0.95006	1.559213
22	6	C	2.212887	-0.33349	-1.86728
23	6	C	3.048163	-0.15914	-0.6792
24	8	O	2.962775	-1.34003	0.307942
25	6	C	1.511646	-1.80387	0.50123
26	6	C	0.445101	-1.07516	-2.94977
27	8	O	3.716292	-1.0453	1.609682
28	8	O	2.955874	-0.54058	2.813376
29	6	C	5.036461	-1.26426	1.6593
30	6	C	2.665146	1.256923	-0.16717
31	6	C	3.557571	2.401236	-0.61962
32	6	C	1.667615	1.422377	0.517094
33	8	O	2.861706	3.758782	-0.49881
34	6	C	4.881866	2.372394	0.182237
35	6	C	4.060605	-0.11626	-1.0844
36	6	C	1.460175	-3.50378	-1.40415
37	1	H	-0.581	0.425801	0.743544
38	1	H	-0.39885	0.346329	-0.9986
39	1	H	-3.35076	-2.41	-0.86643
40	1	H	-3.15931	-0.87435	-1.73076
41	1	H	-4.88702	1.802429	-0.83425

42	1	H	-5.28331	2.051647	0.871525
43	1	H	-3.82245	3.941098	-0.55742
44	1	H	-3.51981	3.667827	1.158524
45	1	H	-5.50256	-2.09122	0.39252
46	1	H	-5.81585	-0.58199	1.281668
47	1	H	-5.82524	-0.5852	-0.49178
48	1	H	-1.95888	3.320261	-2.18125
49	1	H	-1.26793	4.473809	-1.02009
50	1	H	-0.37735	2.99977	-1.44967
51	1	H	-0.38418	2.641042	0.930635
52	1	H	3.511998	-2.14913	-0.18404
53	1	H	1.48564	-2.68392	1.150842
54	1	H	0.93719	-1.02663	1.00243
55	1	H	2.213799	-1.27655	3.148291
56	1	H	3.638534	-0.35025	3.646616
57	1	H	2.406632	0.381344	2.596933
58	1	H	5.613249	-1.0573	2.557143
59	1	H	5.587218	-1.66205	0.80969
60	1	H	3.804239	2.204951	-1.6732
61	1	H	3.52943	4.552333	-0.84942
62	1	H	1.945923	3.794237	-1.0971
63	1	H	2.59452	3.970275	0.541471
64	1	H	5.397827	1.410225	0.110346
65	1	H	5.552602	3.146193	-0.20394
66	1	H	4.693797	2.579038	1.241434
67	1	H	2.533128	-3.40739	-1.58728
68	1	H	1.299428	-4.30553	-0.679
69	1	H	0.974554	-3.78214	-2.34087
70	1	H	-1.26078	3.501043	-0.99115
71	1	H	-1.36233	2.823583	1.337291
72	1	H	-3.28012	0.903883	1.546844

2*S*,4*R*,5*S*,12*R*,17*R*,19*S*,20*R*,23*S*,24*R*,*P*-**7**(8)			Standard Orientation (A.U.)		
Center number	Atomic number	Atomic Type	X	Y	Z
1	6	C	-2.25165	-0.51175	-0.90263
2	6	C	-1.22864	-0.88831	0.259745
3	6	C	-0.62404	0.410625	0.788533
4	8	O	-1.37791	0.885147	1.791748
5	6	C	-2.57059	0.002903	1.993238
6	6	C	-3.63812	0.523116	0.997329
7	6	C	-3.64421	-0.29378	-0.30698
8	6	C	-1.99744	-1.34415	1.526397
9	6	C	-5.13584	0.540928	1.348273
10	6	C	-5.823	0.70558	-0.0422
11	6	C	-4.7404	0.399995	-1.13568
12	6	C	-0.25542	-1.96835	-0.23791
13	8	O	1.154645	-1.69846	-0.83464
14	1	H	1.213101	-0.46071	-1.7265
15	6	C	-0.61462	-3.12593	-0.11298
16	8	O	-2.93651	0.096596	3.461608
17	1	H	0.374138	0.990548	0.407094
18	6	C	-4.05999	-1.28068	-0.06224
19	6	C	-5.27359	-0.42979	-2.30373
20	6	C	-4.15954	1.620501	-1.63207
21	6	C	-3.35086	1.552534	0.753851
22	6	C	2.366604	0.247733	-1.79677
23	6	C	3.389397	0.235642	-0.76629
24	8	O	3.530086	-1.15513	-0.11211
25	6	C	2.157121	-1.65023	0.352389
26	6	C	0.334931	-0.19404	-2.52192
27	8	O	4.650814	-1.26266	0.917495
28	8	O	6.000868	-0.74458	0.476452
29	6	C	4.49987	-1.86801	2.100086
30	6	C	3.189521	1.456423	0.164805
31	6	C	2.723878	2.763047	-0.47482
32	6	C	3.51174	1.382425	1.336804
33	8	O	2.358571	3.796746	0.593125
34	6	C	3.815508	3.292576	-1.43423
35	6	C	4.306091	0.448994	-1.32732
36	6	C	1.490267	-2.88256	-1.78469
37	1	H	-1.89327	0.375462	-1.42708
38	1	H	-2.27127	-1.32769	-1.63088
39	1	H	-1.30134	-1.73972	2.273171
40	1	H	-2.75573	-2.10321	1.336771
41	1	H	-5.43515	-0.40887	1.807668

42	1	H	-5.40414	1.340539	2.044796
43	1	H	-6.68233	0.034502	-0.1405
44	1	H	-6.19269	1.726386	-0.18593
45	1	H	-2.08613	-0.18935	4.088301
46	1	H	-3.24049	1.114756	3.724504
47	1	H	-3.77038	-0.57743	3.680798
48	1	H	-5.66884	-1.39194	-1.95949
49	1	H	-6.0873	0.10194	-2.81329
50	1	H	-4.48248	-0.61972	-3.03721
51	1	H	-4.85892	2.104621	-2.1007
52	1	H	3.855319	-1.8007	-0.94119
53	1	H	2.233897	-2.66054	0.763969
54	1	H	1.787698	-0.9975	1.148228
55	1	H	6.281625	-1.13966	-0.50913
56	1	H	6.005137	0.34977	0.397142
57	1	H	6.780516	-1.02649	1.190005
58	1	H	5.345197	-1.98817	2.772987
59	1	H	3.549216	-2.2522	2.454815
60	1	H	1.83827	2.533115	-1.07421
61	1	H	2.00071	4.715703	0.116248
62	1	H	1.567731	3.415007	1.24523
63	1	H	3.223599	4.047867	1.215828
64	1	H	4.757958	3.470394	-0.90286
65	1	H	4.008707	2.60145	-2.26083
66	1	H	3.489253	4.244071	-1.86725
67	1	H	0.742828	-2.952	-2.57902
68	1	H	2.469686	-2.74875	-2.25073
69	1	H	1.48989	-3.81789	-1.22238
70	1	H	2.214127	-0.19713	-1.74669
71	1	H	3.01601	-0.07275	-0.53038
72	1	H	2.924099	-1.30564	0.39206

2*S*,4*R*,5*S*,12*R*,17*R*,19*S*,20*R*,23*S*,24*R*,*P*-**7**(9)			Standard Orientation (A.U.)		
Center number	Atomic number	Atomic Type	X	Y	Z
1	6	C	1.175506	0.231569	0.107331
2	6	C	1.658458	-1.25677	0.19124
3	6	C	2.046577	-1.70918	-1.23717
4	8	O	3.34813	-1.44741	-1.45487
5	6	C	3.922966	-0.73506	-0.27622
6	6	C	3.588162	0.766466	-0.50247
7	6	C	2.351205	1.18665	0.301136
8	6	C	3.058881	-1.33807	0.849205
9	6	C	4.563203	1.904492	-0.16577
10	6	C	3.646772	3.158012	-0.26717
11	6	C	2.170645	2.683617	-0.05078
12	6	C	0.668587	-2.30631	0.754372
13	8	O	-0.87054	-2.05866	0.866173
14	1	H	-1.11728	-0.96422	1.90465
15	6	C	1.116456	-3.39427	1.060617
16	8	O	5.398739	-1.07931	-0.22733
17	1	H	1.333048	-2.21697	-2.07439
18	6	C	2.635644	1.16689	1.362031
19	6	C	1.430949	3.481387	1.025666
20	6	C	1.491779	2.837931	-1.31295
21	6	C	3.34925	0.862621	-1.56971
22	6	C	-2.18596	-0.14286	1.780883
23	6	C	-3.0128	-0.00592	0.586379
24	8	O	-2.94662	-1.23852	-0.34273
25	6	C	-1.49743	-1.72205	-0.51187
26	6	C	-0.43712	-0.86464	2.907069
27	8	O	-3.70446	-1.00709	-1.65137
28	8	O	-5.16477	-1.39277	-1.62552
29	6	C	-3.12414	-0.52317	-2.75556
30	6	C	-2.59228	1.349369	-0.04739
31	6	C	-3.51854	2.545647	0.108945
32	6	C	-1.50408	1.453073	-0.59029
33	8	O	-4.90954	2.314982	-0.51054
34	6	C	-3.61972	2.945228	1.597199
35	6	C	-4.02257	0.087625	0.988443
36	6	C	-1.50422	-3.34146	1.467044
37	1	H	0.70426	0.413265	-0.86006
38	1	H	0.421763	0.432726	0.866456
39	1	H	3.33293	-2.38247	1.015525
40	1	H	3.136371	-0.80333	1.79736
41	1	H	4.951656	1.79345	0.854397

42	1	H	5.421102	1.962406	-0.8424
43	1	H	3.93046	3.929061	0.455636
44	1	H	3.71103	3.605331	-1.26399
45	1	H	5.539616	-2.16302	-0.16861
46	1	H	5.917267	-0.70448	-1.11547
47	1	H	5.857187	-0.62178	0.654964
48	1	H	1.945183	3.417475	1.990924
49	1	H	1.369046	4.53634	0.736793
50	1	H	0.40853	3.106371	1.161918
51	1	H	0.553928	2.61851	-1.16583
52	1	H	-3.49663	-2.02164	0.190933
53	1	H	-1.48228	-2.62011	-1.13617
54	1	H	-0.90794	-0.96652	-1.02838
55	1	H	-5.27707	-2.46899	-1.43678
56	1	H	-5.70875	-0.87868	-0.8224
57	1	H	-5.65857	-1.15744	-2.57272
58	1	H	-3.6954	-0.38329	-3.66979
59	1	H	-2.0743	-0.2516	-2.79749
60	1	H	-3.02262	3.356804	-0.43576
61	1	H	-5.4803	3.248418	-0.46491
62	1	H	-4.83889	2.009255	-1.55897
63	1	H	-5.483	1.55589	0.033109
64	1	H	-2.63345	3.153795	2.024499
65	1	H	-4.22869	3.851047	1.685967
66	1	H	-4.09591	2.163306	2.199013
67	1	H	-2.57287	-3.20648	1.650211
68	1	H	-1.37022	-4.17648	0.774813
69	1	H	-1.0237	-3.59637	2.413031
70	1	H	1.477049	-1.80436	0.52231
71	1	H	0.497832	-0.91844	-2.92119
72	1	H	3.64071	-1.05969	1.725275

Supplementary Source Data

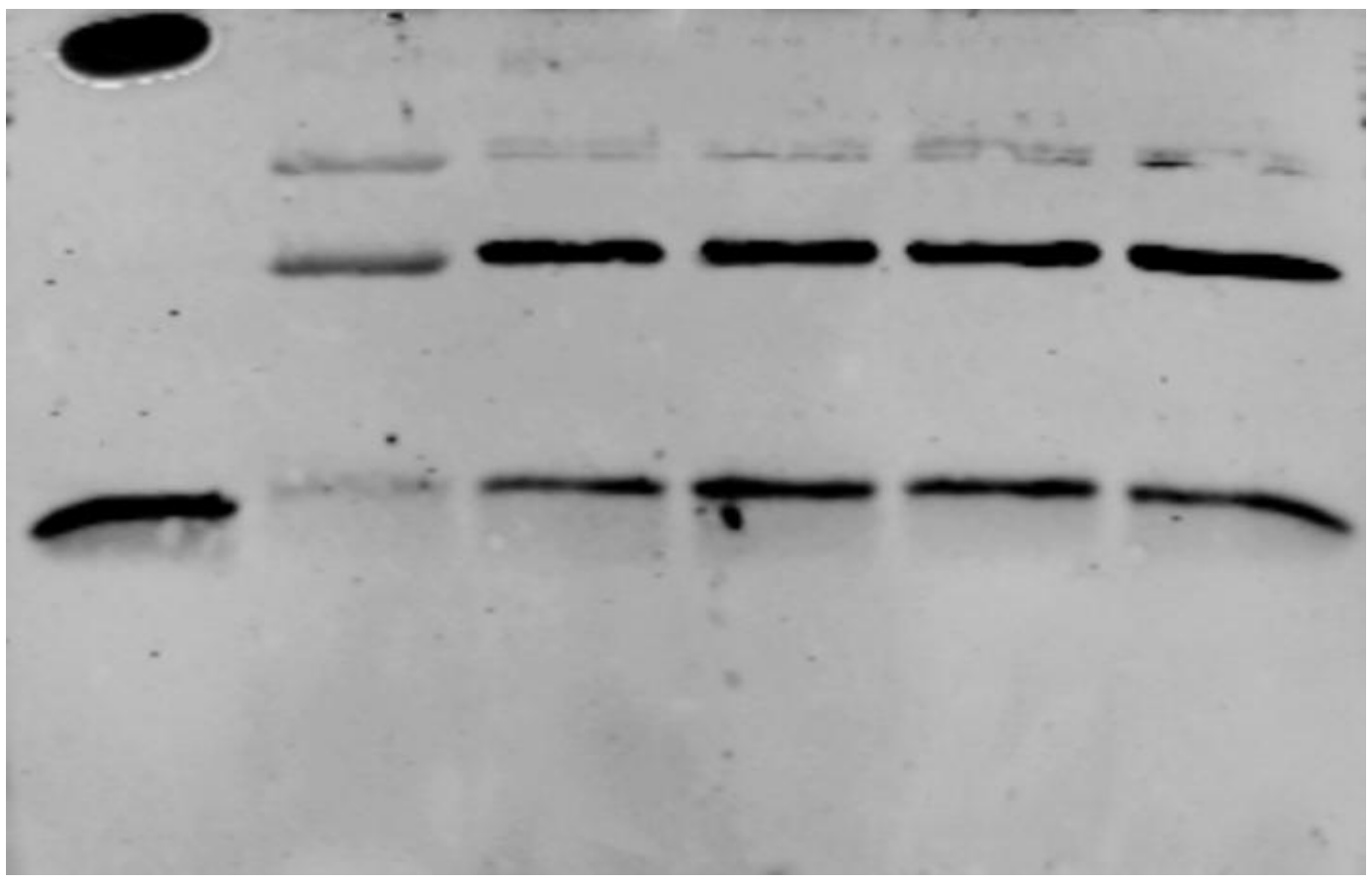

Source Data S1. Original uncropped western blot image for the TNF cross-linking assay.

Full membrane image from the BS3-mediated TNF cross-linking assay used to assess HPJ-associated changes in TNF monomer, dimer, and trimer species. TNF was detected with an anti-TNF antibody. The corresponding cropped result is shown in Figure 4F.

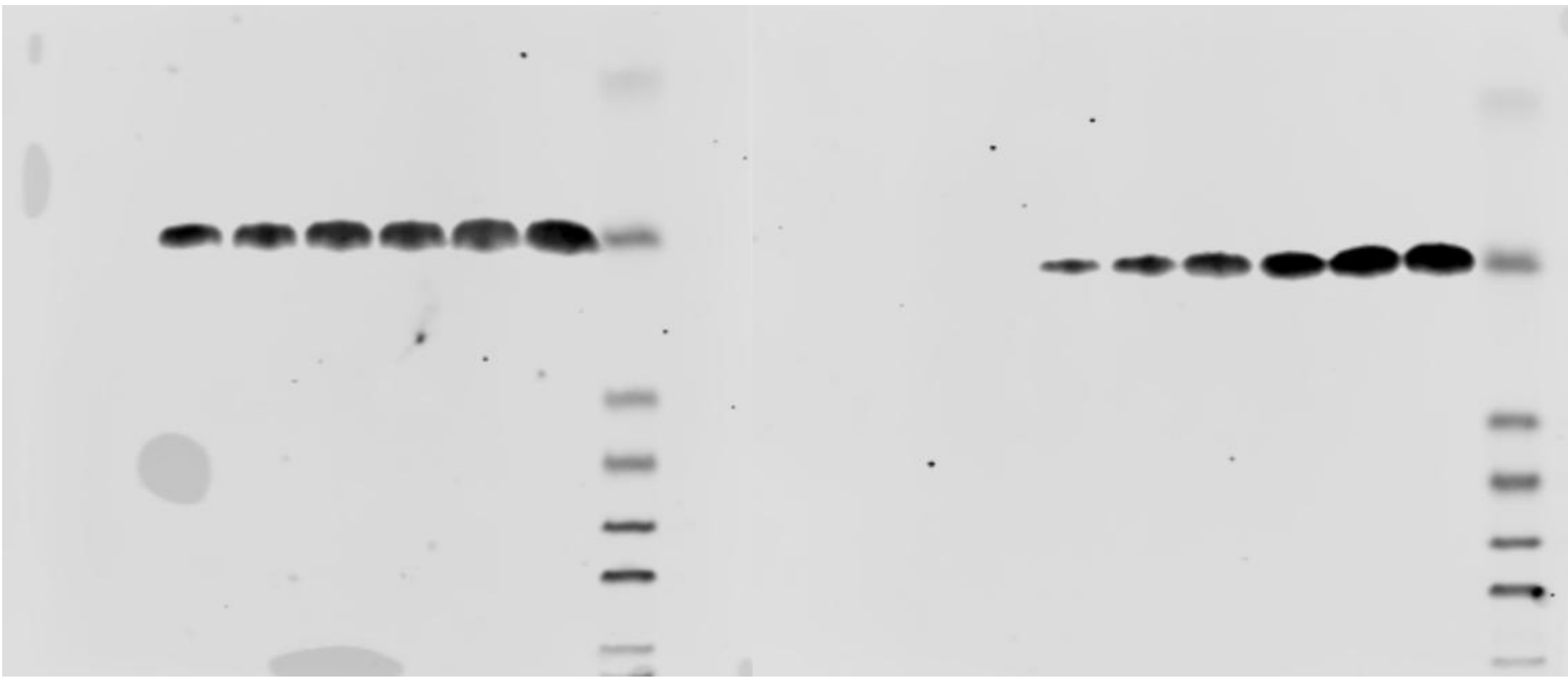

Source Data S2. Original uncropped western blot image for the CETSA assay.

Full membrane image from the cellular thermal shift assay (CETSA) evaluating HPJ-induced thermal stabilization of TNF after heat treatment. TNF was detected with an anti-TNF antibody. The corresponding cropped result is shown in Figure 4E.

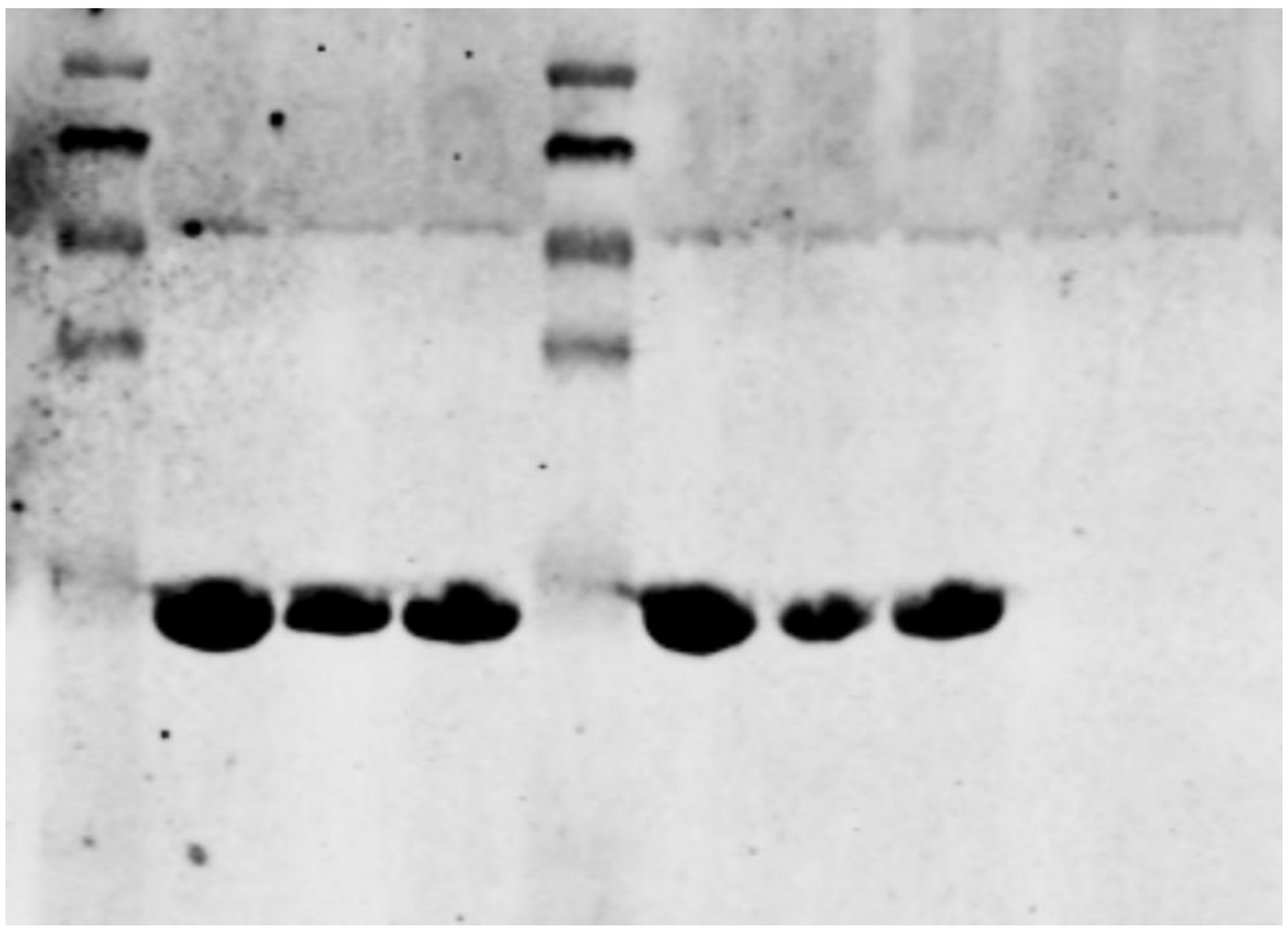

Source Data S3. Original uncropped western blot image for the DARTS assay.

Full membrane image from the drug affinity responsive target stability (DARTS) assay evaluating HPJ-associated protection of TNF against protease digestion. TNF was detected with an anti-TNF antibody. The corresponding cropped result is shown in Figure 4D.

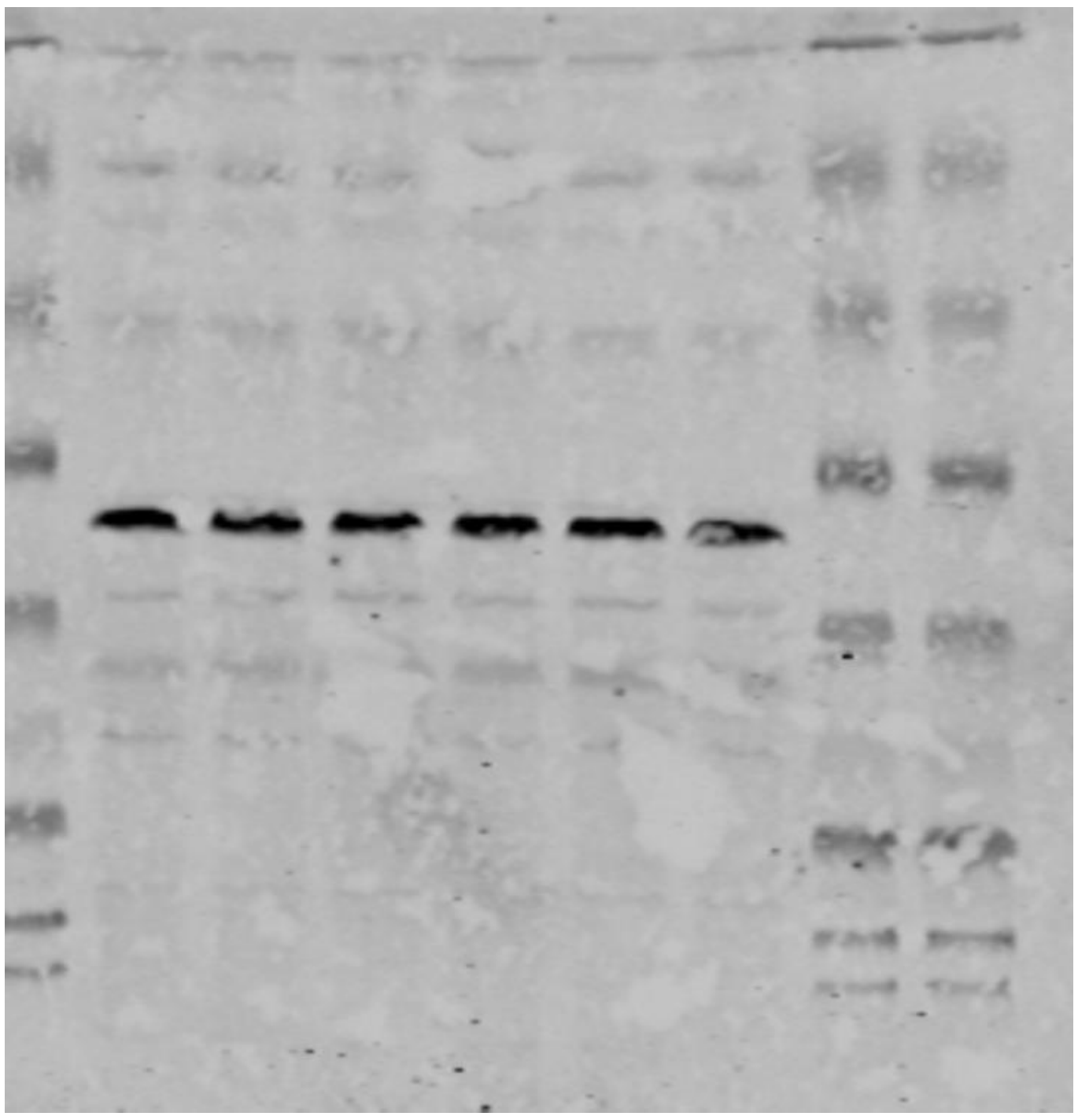

Source Data S4. Original uncropped western blot image for p65.

Full membrane image used to detect total p65 in TNF-stimulated cells treated with HPJ or vehicle control. The blot supports evaluation of NF-κB pathway modulation and corresponds to the related western blot panels in the main or supplementary figures.

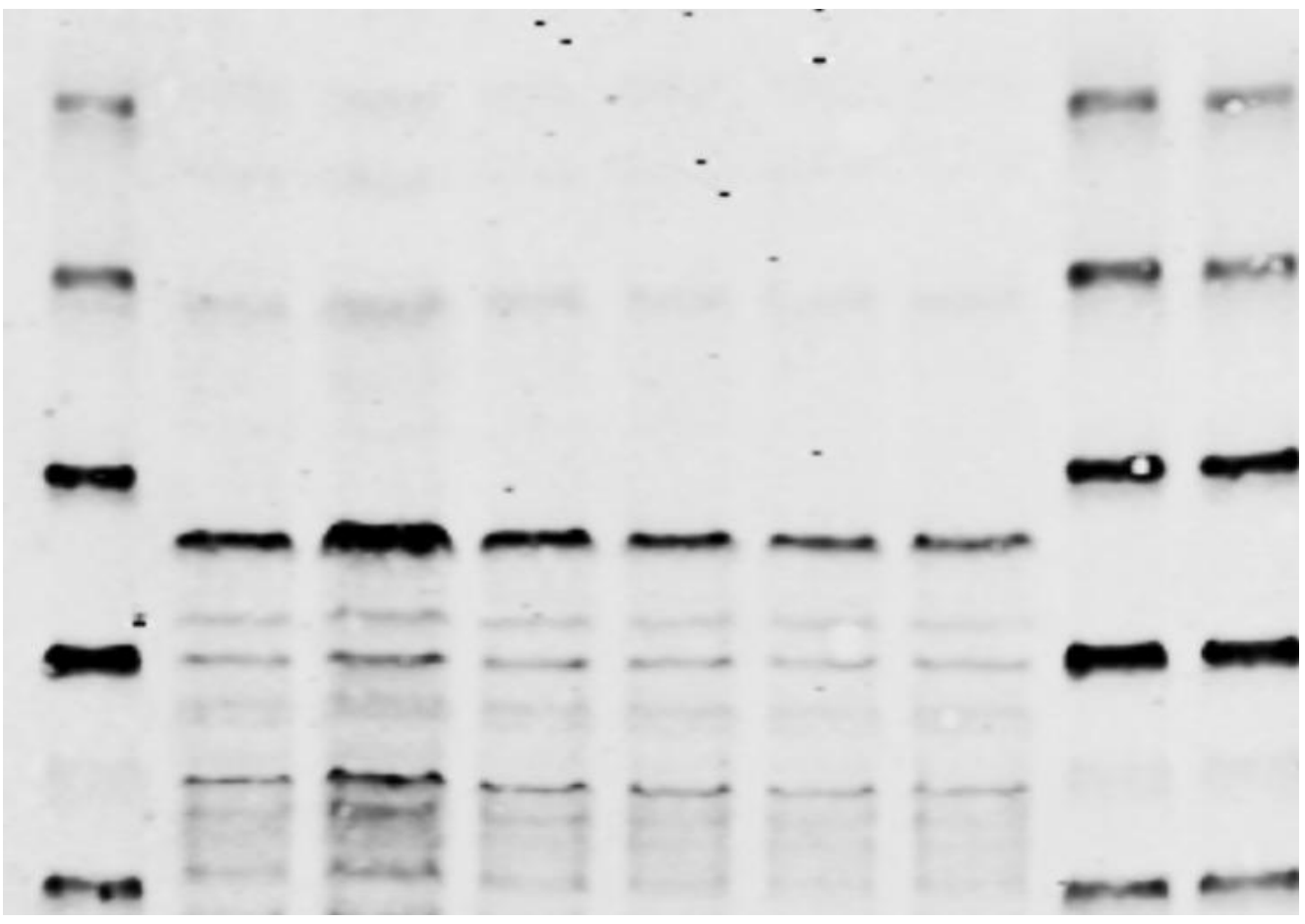

Source Data S5. Original uncropped western blot image for phosphorylated p65 (p-p65).

Full membrane image used to detect phosphorylated p65 (p-p65) in TNF-stimulated cells treated with HPJ or vehicle control. The blot supports evaluation of NF-κB activation and corresponds to the related western blot panels in the main or supplementary figures.

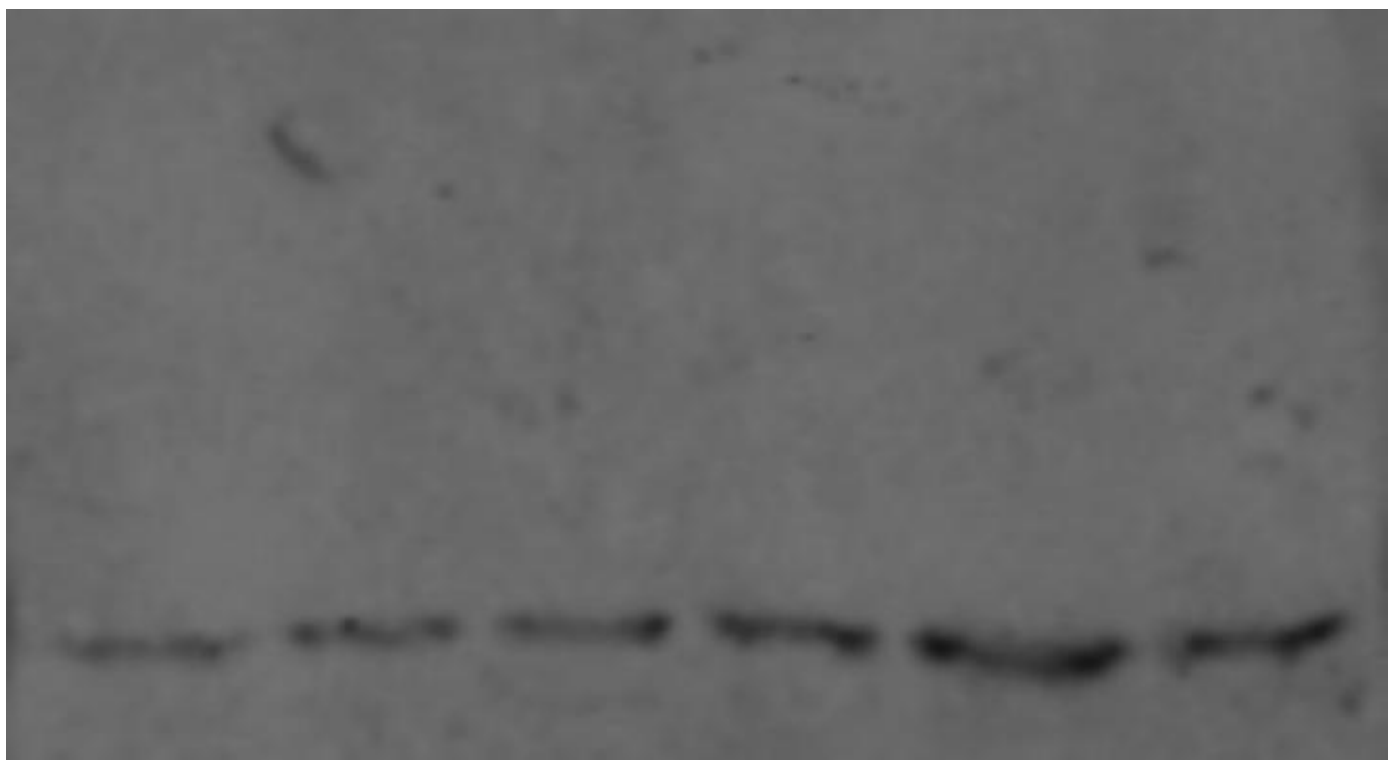

Source Data S6. Original uncropped western blot image for TNF.

Full membrane image used to detect TNF in the corresponding western blot analysis. The blot supports assessment of TNF-related signaling after HPJ treatment and corresponds to the related western blot panels in the main or supplementary figures.

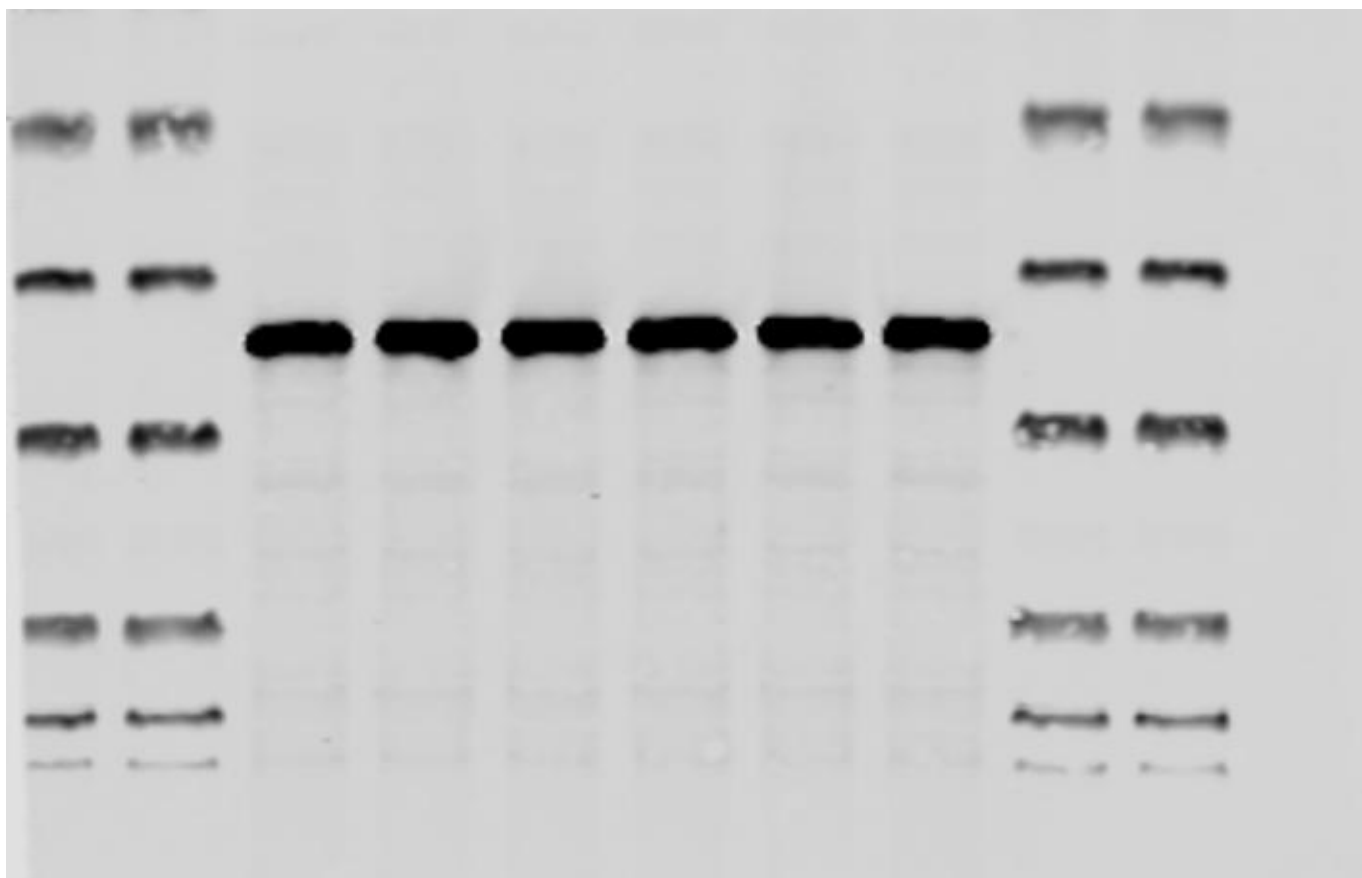

Source Data S7. Original uncropped western blot image for β-actin.

Full membrane image used to detect β-actin as the loading control for the corresponding western blot experiments. The cropped β-actin panel was used for normalization of the indicated protein signals.

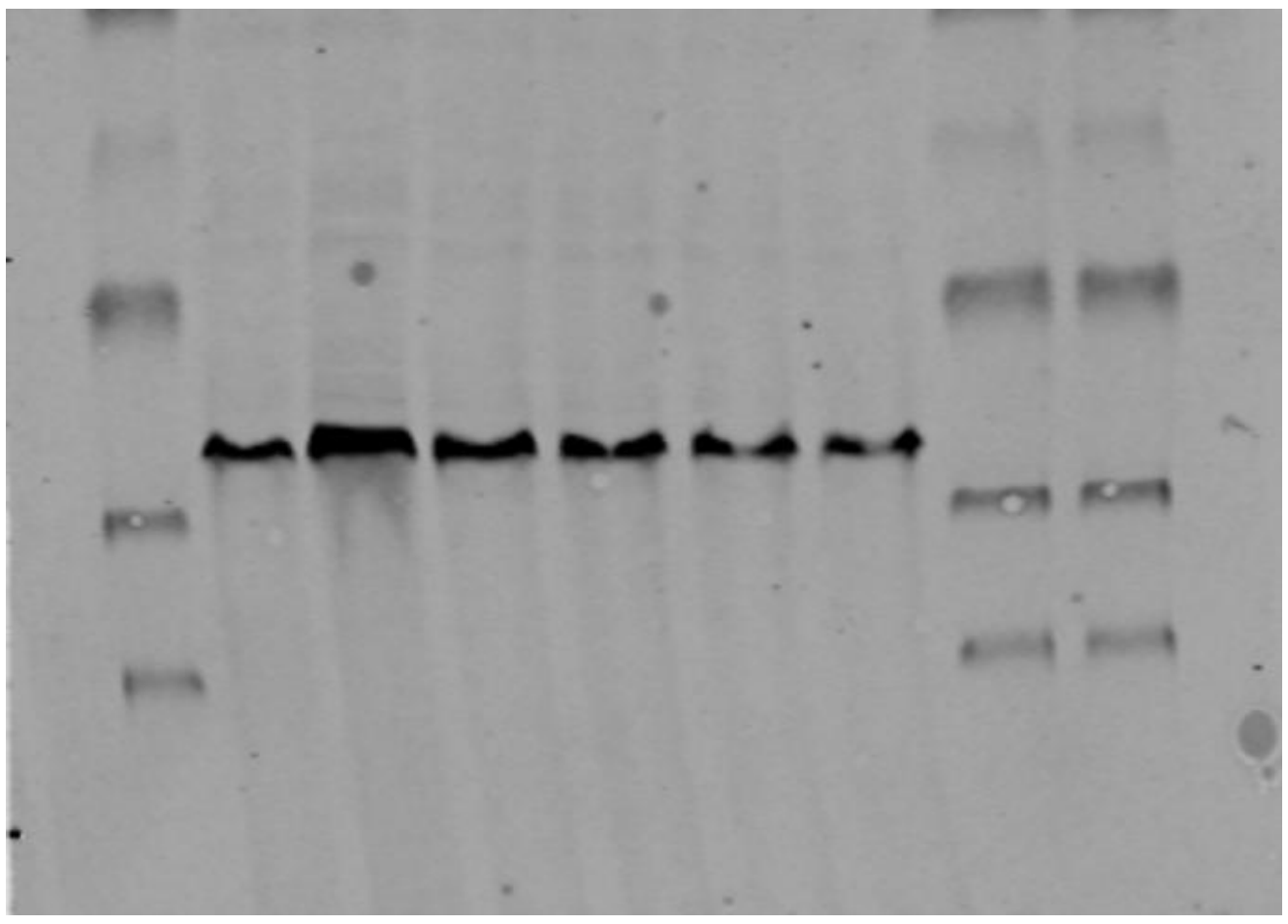

Source Data S8. Original uncropped western blot image for NLRP3.

Full membrane image used to detect NLRP3 in TNF-stimulated cells treated with HPJ or vehicle control. The blot supports assessment of inflammatory pathway modulation and corresponds to the related western blot panels in the main or supplementary figures.

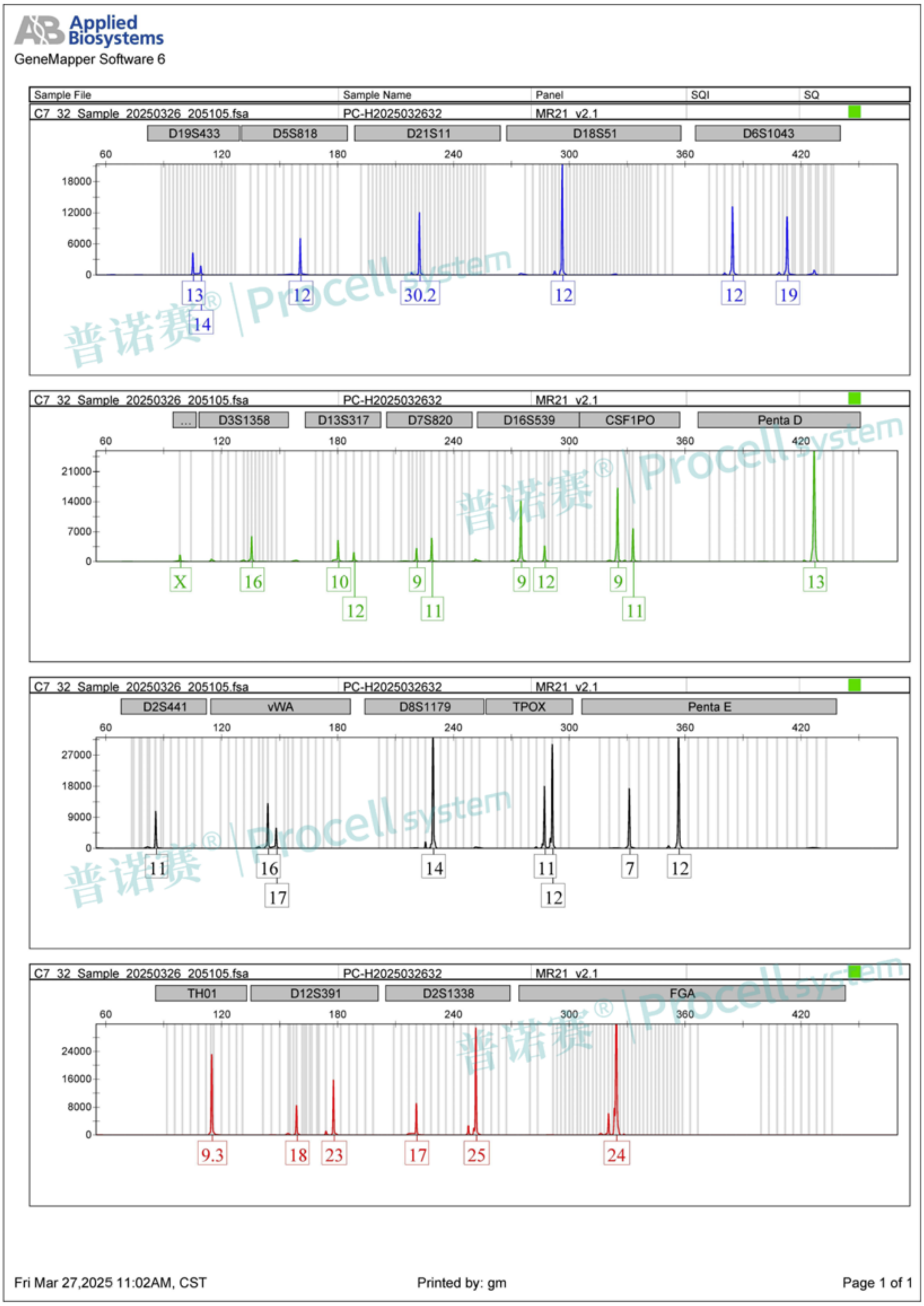

Source Data S9. STR authentication report or source certificate for HaCaT cells. Authentication record for HaCaT cells used in the transcriptomic, RT-qPCR, western blotting, and immunofluorescence experiments.

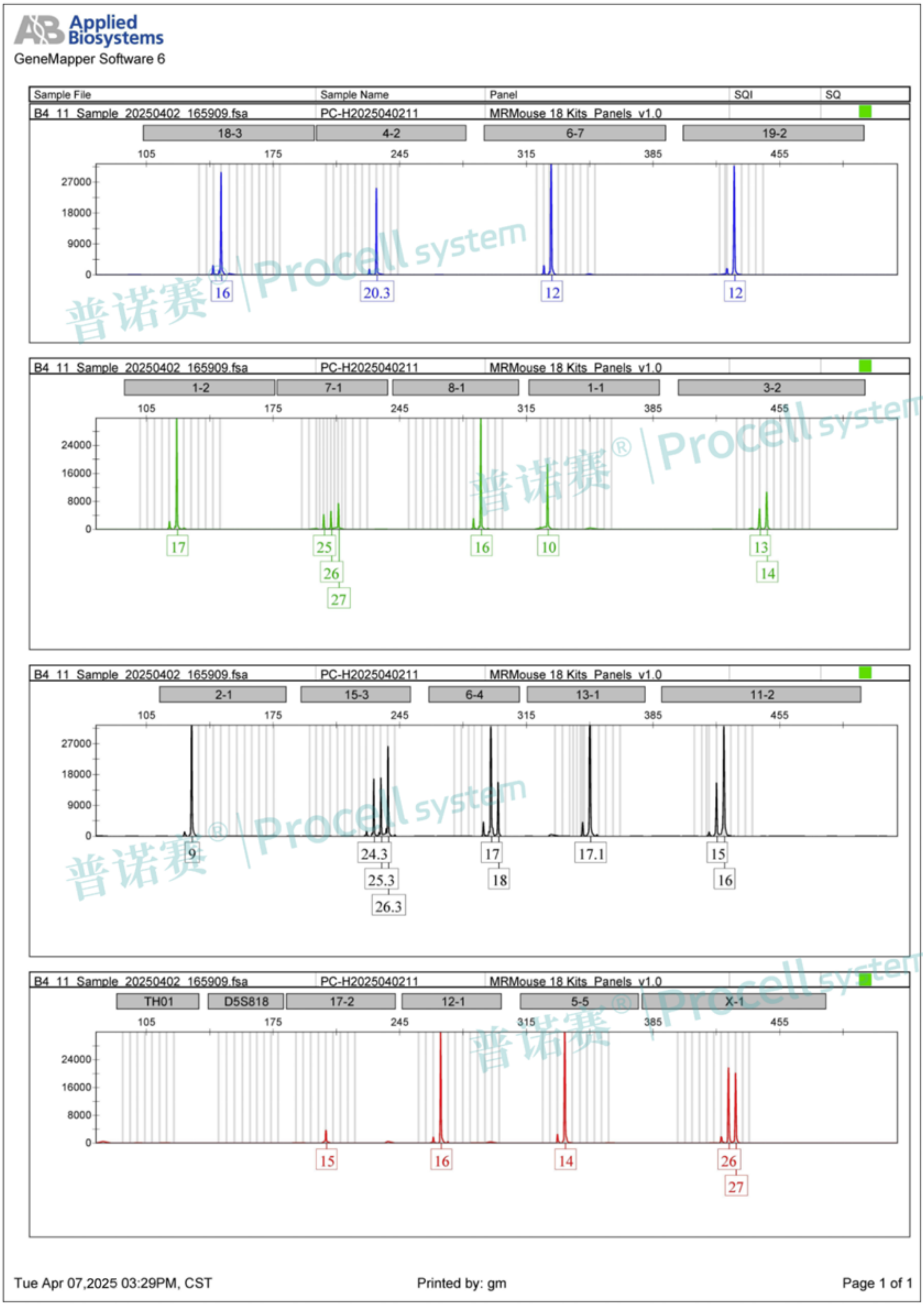

Source Data S10. STR authentication report or source certificate for L929 cells. Authentication record for L929 cells used in the TNF-induced cytotoxicity protection assay.

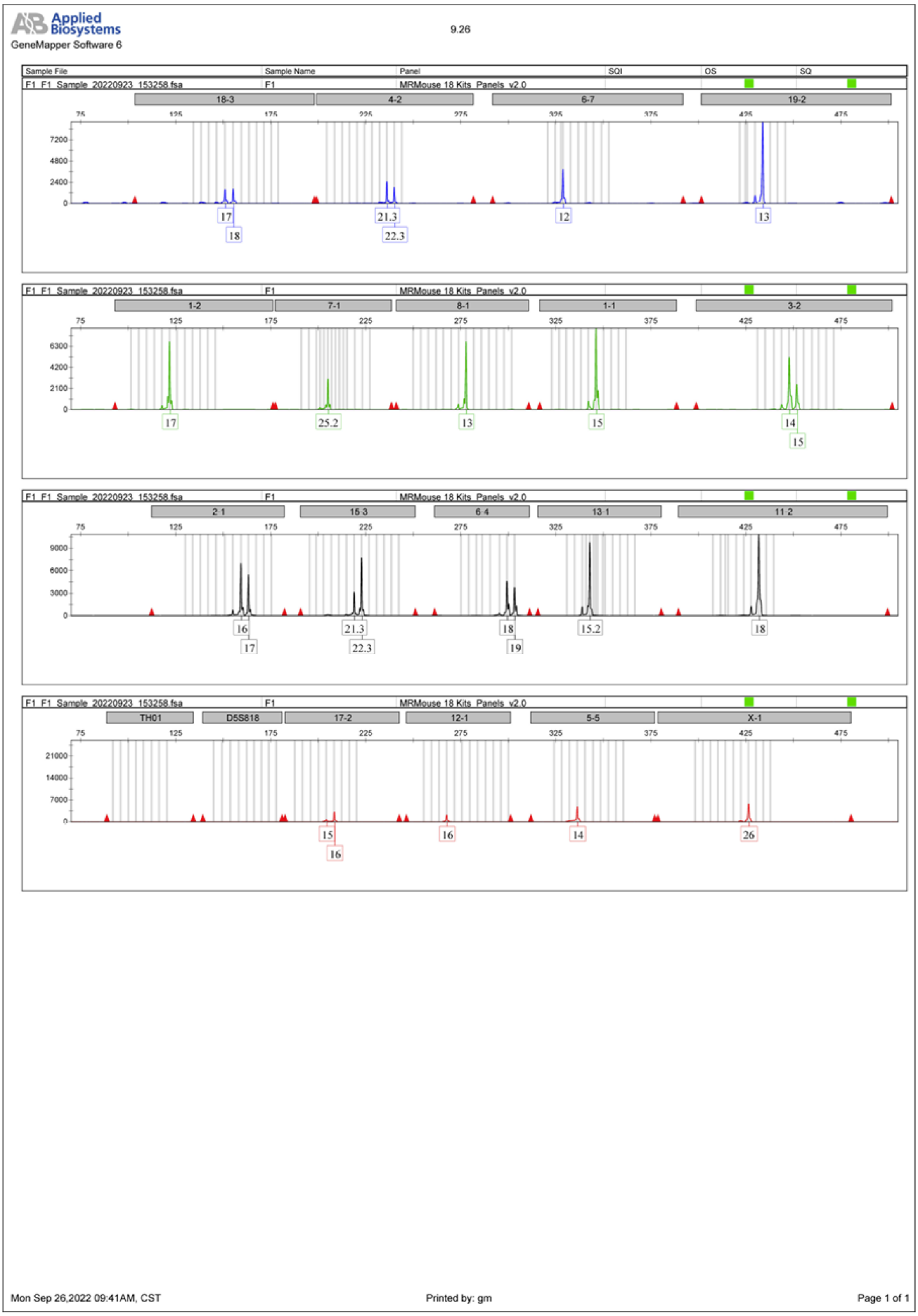

Source Data S11. STR authentication report or source certificate for WEHI164 cells.

Authentication record for WEHI164 cells used in the LT-α-induced cytotoxicity assay.

References

1. Sybyl Software, version X 2.0; Tripos Associates Inc.: St. Louis, MO, **2013**.

2. Neese, F. The ORCA program system, Wiley Interdiscip. Rev.: Comput. Mol. Sci. **2012**, *2*, 73–78.

3. Stephens, P. J.; Harada, N. ECD cotton effect approximated by the Gaussian curve and other methods. Chirality **2010**, *22*, 229–233
